# Psychiatric Disorders Among Fathers in Sweden Before, During, and After Partner Pregnancy

**DOI:** 10.1001/jamanetworkopen.2026.2725

**Published:** 2026-03-23

**Authors:** Nanyan Xiang, Jing Zhou, Yifei Lin, Yihui Yang, Miriam Martini, Bowen Tang, Yufeng Chen, Fotios C. Papadopoulos, Emma Fransson, Alkistis Skalkidou, Jin Huang, Donghao Lu

**Affiliations:** 1 Department of Urology, Health Data Science Laboratory, Innovation Institute for Integration of Medicine and Engineering, Frontiers Science Center for Disease-Related Molecular Network, West China Hospital, Sichuan University, Chengdu, Sichuan, China; 2 Institute of Environmental Medicine, Karolinska Institutet, Stockholm, Sweden; 3 Department of Medical Epidemiology and Biostatistics, Karolinska Institutet, Stockholm, Sweden; 4 Psychiatry, Department of Medical Sciences, Uppsala University, Uppsala, Sweden; 5 Department of Women’s and Children’s Health, Uppsala University, Uppsala, Sweden

## Abstract

**Importance:**

Paternal psychiatric disorders during the perinatal period can affect the health of the entire family; however, these conditions have often been underrecognized, and little is known about their incidence and timing of onset.

**Objective:**

To investigate incidence patterns of new-onset diagnosed psychiatric disorders among men in Sweden before, during, and after a partner’s pregnancy.

**Design, Setting, and Participants:**

This prospective cohort study used linked national register data for all fathers of children born in Sweden between January 1, 2003, and December 31, 2021, with follow-up from 1 year before to 1 year after pregnancy. Data were analyzed from October 1, 2024, to March 31, 2025.

**Exposures:**

The time during pregnancy and 1 year after childbirth (post partum) were considered the risk periods, while 1 year before pregnancy (before conception) was used as the reference period.

**Main Outcomes and Measures:**

Annual and weekly incidence rates (IRs) of clinical diagnoses of any psychiatric disorder and 9 type-specific disorders were calculated and standardized by age and calendar year. Adjusted Poisson regression analysis was used to further estimate incidence rate ratios (IRRs) of psychiatric disorders during and after pregnancy compared with before conception.

**Results:**

This study included 1 915 722 births from 1 096 198 fathers (mean [SD] age at childbirth, 33.8 [6.2] years) in Sweden. IRs of any diagnosed psychiatric disorder were lower during pregnancy (eg, pregnancy week 1: IR, 5.50 [95% CI, 4.69-6.31] per 1000 person-years) and the early postpartum period (eg, postpartum week 1: IR, 5.19 [95% CI, 4.41-5.97] per 1000 person-years) than in the corresponding preconception weeks (eg, preconception week 1: IR, 7.00 [95% CI, 5.97-8.04] per 1000 person-years); they returned to comparable rates later post partum. This pattern was also observed for IRRs of anxiety, alcohol use, and drug use (ie, the use of nonalcohol, nontobacco psychoactive drugs) disorders. IRRs of depression (eg, postpartum weeks 45-49: IRR, 1.30 [95% CI, 1.12-1.52]) and stress-related disorders (eg, postpartum weeks 45-49: IRR, 1.36 [95% CI, 1.15-1.61]), however, showed a notable 30% increase toward the end of the first postpartum year. In contrast, IRRs of diagnosis of tobacco use disorder, attention-deficit/hyperactivity disorder, bipolar disorder, or psychosis remained relatively stable before, during, and after pregnancy.

**Conclusions and Relevance:**

In this nationwide cohort study, fathers in Sweden were less likely to be diagnosed with a psychiatric disorder during a partner’s pregnancy and early post partum than before conception, but IRs returned to comparable levels thereafter. These incidence patterns may reflect transient protection and delayed detection during the transition to fatherhood and support the need for paternal mental health surveillance, particularly for increased depression and stress-related disorders in the late postpartum period.

## Key Points

**Question:**

What are the incidence patterns of paternal psychiatric disorders before, during, and after a partner’s pregnancy?

**Findings:**

In this cohort study of 1 096 198 fathers in Sweden, incidence rates of any diagnosed psychiatric disorder declined during pregnancy and early post partum compared with before conception and then returned to preconception levels later. Incidence rate ratios of anxiety, alcohol use, and drug use disorders followed a similar pattern, whereas those of depression and stress-related disorders increased by more than 30% toward the end of the first postpartum year.

**Meaning:**

These findings suggest that fatherhood may be associated with a temporary reduction in psychiatric disorder diagnoses followed by increased vulnerability later post partum, highlighting the need for sustained paternal mental health surveillance, particularly for depression and stress-related disorders.

## Teaser

This cohort study examines incidence patterns of new-onset diagnosed psychiatric disorders among men in Sweden before, during, and after a partner’s pregnancy.

## Introduction

Parental mental health is central to family functioning and child development, influencing emotional well-being, caregiving, and long-term health outcomes.^1,2,3^ Psychiatric disorders among parents affect not only their own health but also the well-being of their partners and children.^4,5^ Compared with mothers, mental health among fathers has received far less research attention despite evidence suggesting that paternal perinatal depression can increase risk for current and future mental health problems among both mothers and children.^6,7^ Moreover, fathers with psychiatric disorders may experience additional stigmatization and delayed recognition and treatment,^8^ allowing adverse family consequences to persist.

The perinatal period might be particularly vulnerable for paternal mental health. During and after a partner’s pregnancy, fathers experience a core identity shift, often accompanied by positive experiences such as a sense of accomplishment and joyful moments when bonding with their child.^9^ However, this transition may also introduce risk factors for mental health problems among fathers, including deterioration in their relationship with their partner due to reduced opportunities for communication and emotional connection as they transition into fatherhood^10^ and sleep deprivation due to caregiving responsibilities.^11^ The combined effects of these factors make the trajectory of paternal mental health complex across the perinatal period.^12^ Lack of clarity about critical time windows in these trajectories may add challenges in identifying the optimal timing for paternal mental health screening, which has been implemented for mothers in many countries.^13,14,15,16^

Several studies have suggested that the prevalence of paternal psychiatric disorders is higher during the first 6 months post partum compared with males in the general population.^12,17,18^ However, prevalence alone does not capture the timing of new episodes, which is essential to allocate clinical resources and understand potential mechanisms. To our knowledge, only 2 Danish population-based studies have examined the incidence of paternal psychiatric disorders. One study reported an increasing trend over the perinatal period relative to early pregnancy, with postpartum incidence slightly higher than during pregnancy.^19^ Another study found that fathers had higher rates of psychiatric disorders post partum compared with nonfathers.^20^ However, no studies, to our knowledge, have estimated the incidence of paternal psychiatric disorders before pregnancy or statistically compared it with the pregnancy or postpartum periods. Such comparisons could reveal whether the transition to fatherhood itself is accompanied by increased risk of mental health problems. Additionally, research during the perinatal period has primarily focused on depression and anxiety,^12,19,21^ with limited exploration of other types of psychiatric disorders.

To this end, we used data from nationwide health care registers in Sweden to investigate incidence patterns of new-onset clinically diagnosed paternal psychiatric disorders, overall and by specific types, during and after pregnancy compared with before pregnancy. We also examined secular trends in the incidence of paternal psychiatric disorders across the study period.

## Methods

### Study Design

We conducted a nationwide cohort study of all fathers whose child was born between January 1, 2003, and December 31, 2021, in Sweden, using data from national registers. Ethical approval for this study was obtained from the Swedish Ethical Review Authority. In accordance with Swedish legislation, informed consent is not required for studies based solely on register data. This study followed the Strengthening the Reporting of Observational Studies in Epidemiology (STROBE) reporting guideline.

We identified 2 080 206 births during 2003 to 2021 from the Medical Birth Register (MBR), which contains information on 98% of all births in Sweden since 1973.^22^ After we excluded erroneous records (n = 7729) due to reused identifiers or conflicting data (eg, death before conception) and duplicated gestation records (n = 55 989) so that only 1 record was kept for each pregnancy in multiple births, a total of 2 016 488 births remained. These births were linked to their fathers (n = 1 132 593) primarily using the child’s record containing paternal information in the Multi-Generation Register (MGR), which provides familial linkage data for individuals born since 1932.^23^ Paternal linkage was further supplemented using maternal reports of cohabitation with the father in the MBR, combined with cohabitation information from Statistics Sweden. These fathers were identified based on legally recognized paternity in national administrative registers, excluding adoptive relationships.^24^ Paternal records could not be linked for 100 766 births (5.0%). After further excluding fathers with reused personal identity numbers (n = 36 392) or conflicting data (n = 3 died before conception), 1 096 198 fathers and 1 915 722 births were included in the final analysis.

As reported in an earlier study,^25^ the conception date was calculated using the estimated gestational length recorded in the MBR, which was primarily based on routine ultrasound assessments in early to midpregnancy. The antepartum period was defined as the time between conception and the end of pregnancy, the preconception period referred to the year prior to pregnancy, and the postpartum period extended to 1 year after pregnancy. All fathers were followed up from 1 year before pregnancy, immigration, or January 1, 2003, whichever came last, until the first diagnosis of any psychiatric disorder, 1 year after pregnancy, emigration, death, or December 31, 2022, whichever came first. Because some fathers entered follow-up through immigration, were observed from January 1, 2003, or experienced closely spaced consecutive births, 25.1% of childbirths did not contribute a complete 1-year preconception follow-up.

### Ascertainment of Psychiatric Disorders

All diagnoses of psychiatric disorders were identified using the Swedish version of the *International Statistical Classification of Diseases and Related Health Problems, Tenth Revision* (*ICD-10*) codes (F10-F99). Data were obtained from the National Patient Register (NPR), which covers nationwide inpatient psychiatric care since 1973 and specialist outpatient visits since 2001.^26^ In addition to looking at any psychiatric condition, we defined type-specific psychiatric disorders as secondary outcomes, including depression, anxiety, stress-related disorder, alcohol use disorder, tobacco use disorder, drug use disorder (ie, the use of nonalcohol, nontobacco psychoactive drugs), bipolar disorder, and psychosis. We further included attention-deficit/hyperactivity disorder (ADHD). Although ADHD is a neurodevelopmental disorder typically with onset in childhood, many individuals are only diagnosed in adulthood.^27^ For ease of readability, we have grouped ADHD with the psychiatric disorders. The corresponding *ICD-10* codes for each category are detailed in eTable 1 in Supplement 1.

### Covariates

We determined the calendar year and season at the date of pregnancy end, and we calculated paternal age at that time using the father’s date of birth. Information on demographic and socioeconomic characteristics, including country of birth, county of residence, civil status, income, and education level, was obtained from the Total Population Register and the Longitudinal Integration Database for Health Insurance and Labor Market. Additionally, we retrieved any psychiatric diagnosis before the start of the preconception, pregnancy, and postpartum periods from the NPR to account for psychiatric history, while the availability of inpatient records since 1973 and outpatient records since 2001 in the NPR limited the look-back period. The number of children prior to the current pregnancy was further identified through linkage with the MBR and the MGR.

### Statistical Analysis

First, we estimated the annual incidence rate (IR) of any psychiatric disorder, as well as different categories of psychiatric disorders, for 3 periods (before, during, and after pregnancy) from 2003 to 2021. The IR was calculated by dividing the number of newly diagnosed cases by the total accumulated person-years of follow-up within each calendar year, and it was further standardized by age at childbirth. In addition, we calculated weekly age- and year-standardized IRs of psychiatric disorders across the 3 periods.

Next, we used adjusted Poisson regression models to estimate incidence rate ratios (IRRs) of psychiatric disorders at 5-week intervals, comparing the incidence in each interval during pregnancy and post partum with that in the preconception period to observe dynamic changes in incidence patterns over time. Model 1 was adjusted for age and calendar year at childbirth and follow-up week. Model 2 was additionally adjusted for paternal country of birth, region of residence, education and annual income before pregnancy, and season. Model 3 was additionally adjusted for civil status during pregnancy, multiple gestations, number of children, and history of psychiatric disorder. These models did not account for potential clustering arising from one father contributing multiple pregnancies.

Because fathers with a psychiatric history may have a different incidence pattern in the peripartum period, we focused on first-ever psychiatric disorder diagnosis in the type-specific analysis, excluding fathers with a history of any other psychiatric conditions. In addition, because more than half of psychiatric disorders in Sweden are managed within primary care and thus not captured in the NPR,^28^ we estimated IRRs in Stockholm County, where both specialized care and primary care data were available for fathers. IRRs were also estimated after excluding childbirths with incomplete 1-year preconception follow-up. To address potential within-father clustering arising from multiple childbirths, IRR calculations were further restricted to first childbirths only.

The nationwide postpartum depression screening program was implemented for mothers in Sweden in 2010, which may benefit the detection of mental illness among fathers as well. We therefore stratified the analysis by childbirth year (before and after 2010). We also performed analyses stratified on by educational level, country of birth, and number of children to assess whether the IRRs modified across these subgroups.

Data management was performed using SAS, version 9.4 (SAS Institute Inc), and statistical analyses were carried out with Stata, version 17 (StataCorp LLC). Two-tailed *P* < .05 was considered statistically significant. Data were analyzed from October 1, 2024, to March 31, 2025.

## Results

This study included 1 096 198 fathers and 1 915 722 childbirths. The Table summarizes the characteristics of included fathers, all of whom were recorded as male in terms of biological sex in the national registers, and all childbirths. Most fathers (77.5%) were born in Sweden, resided in Central Sweden (61.2%), had completed 10 to 12 years of education (46.1%), and cohabited with their partner (64.6%). The mean (SD) paternal age at childbirth was 33.8 (6.2) years, and 50.1% were first-time fathers.

**Table.** Characteristics of Fathers and Corresponding Pregnancies^a^

Characteristic	Values (N = 1 915 722 births)
**Overall**	
Country of birth	
Sweden	1 484 442 (77.5)
Other	431 280 (22.5)
Psychiatric history	
Yes	191 235 (10.0)
No	1 724 487 (90.0)
**Before pregnancy**	
Annual income, Sk^b^	
Median (IQR)	1696 (1199-2293)
Quartile	
First	467 117 (24.4)
Second	466 576 (24.4)
Third	466 265 (24.3)
Fourth	466 450 (24.3)
Unknown	49 314 (2.6)
Region of residence in Sweden	
South	438 728 (22.9)
Central	1 171 915 (61.2)
North	305 079 (15.9)
Education, y	
<10	223 132 (11.6)
10-12	882 644 (46.1)
≥13	732 313 (38.2)
Unknown	77 633 (4.1)
No. of children	
0	959 243 (50.1)
1	686 405 (35.8)
2	203 115 (10.6)
≥3	66 959 (3.5)
**During pregnancy**	
Paternal civil status	
Cohabitating	1 237 683 (64.6)
Not cohabitating	678 039 (35.4)
Multiple gestations	
Yes	26 956 (1.4)
No	1 888 766 (98.6)
**At childbirth**	
Paternal age, y	
Mean (SD)	33.8 (6.2)
Age group	
<20	7364 (0.4)
20-24	105 326 (5.5)
25-29	417 385 (21.8)
30-34	654 276 (34.2)
35-39	453 221 (23.7)
≥40	278 150 (14.5)
Calendar year	
2003-2005	378 909 (19.8)
2006-2009	404 468 (21.1)
2010-2013	410 190 (21.4)
2014-2017	418 164 (21.8)
2018-2021	303 991 (15.9)
Season	
March to May	507 752 (26.5)
June to August	510 519 (26.6)
September to November	453 848 (23.7)
December to February	443 603 (23.2)
Birthweight, g	
<1500	12 960 (0.7)
1500-<2500	56 628 (3.0)
≥2500	1 843 793 (96.2)
Unknown	2341 (0.1)
Length of gestation, wk	
<32	16 260 (0.8)
32-36	93 465 (4.9)
37-41	1 712 647 (89.4)
≥42	93 350 (4.9)

^a^ Unless indicated otherwise, values are presented as the No. (%) of births.

^b^ To convert Swedish krona to US dollars, multiply by 0.11.

**Figure 1.**
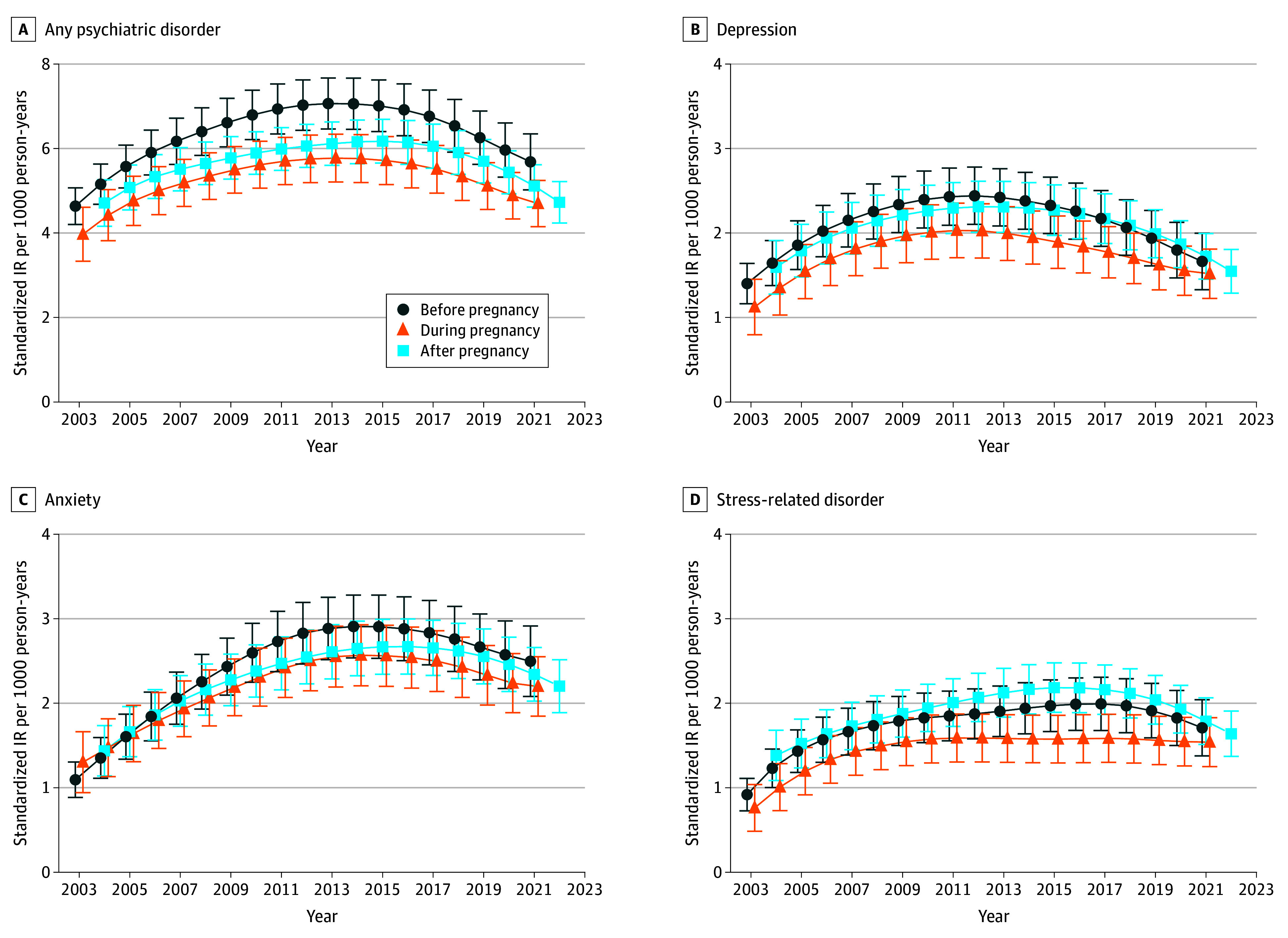
Line Graphs of Standardized Incidence Rates (IRs) of Any and Common Paternal Psychiatric Disorders Throughout Pregnancy, 2003-2022 IRs were standardized by age at childbirth. The smoothed trend of standardized IRs across years used locally weighted scatterplot smoothing. Error bars indicate 95% CIs.

We examined temporal trends in psychiatric disorder incidence from 2003 to 2013. We observed that IRs of any psychiatric disorder increased consistently before, during, and after pregnancy, followed by a decline after 2013 (eg, before conception: IR, 4.64 [95% CI, 4.20-5.07] per 1000 person-years in 2003; 7.06 [95% CI, 6.46-7.67] per 1000 person-years in 2013; and 5.69 [95% CI, 5.02-6.35] per 1000 person-years in 2021). Similar patterns in IRs were observed for depression, anxiety, stress-related disorder, alcohol use disorder, and drug use disorder. In contrast, IRs of tobacco use and bipolar disorder increased gradually before stabilizing, while psychosis remained stable throughout (Figure 1 and eFigure 1 in Supplement 1, with estimated IRs provided in eTable 2 in Supplement 1). IRs of ADHD continued to rise across the study period (2003-2021), with a slower increase after 2013.

**Figure 2.**
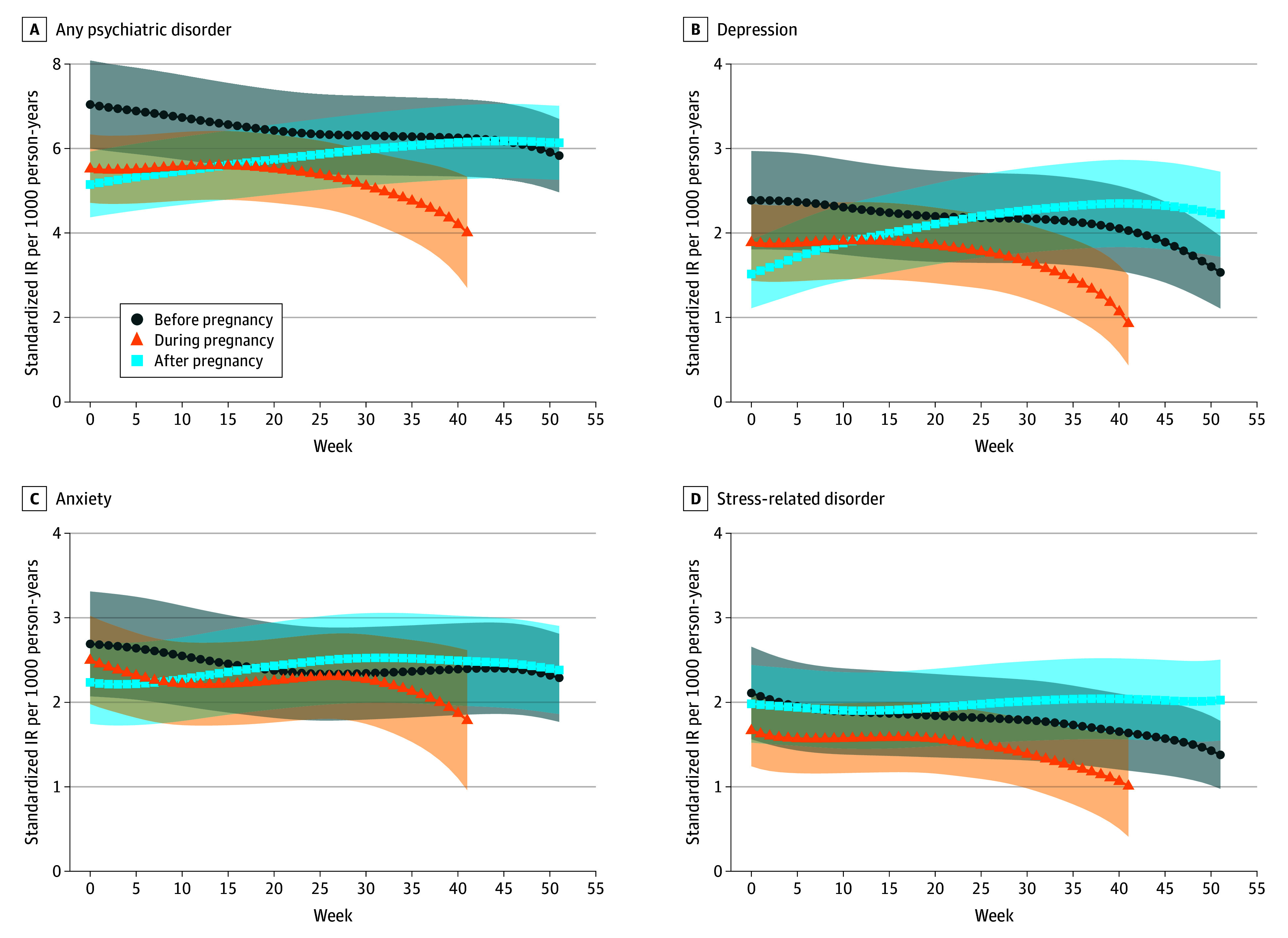
Line Graphs of Standardized Incidence Rates (IRs) of Any and Common Paternal Psychiatric Disorders Throughout Pregnancy by Week IRs were standardized by age and calendar year at childbirth. The smoothed trend of standardized IRs across weeks used locally weighted scatterplot smoothing. Shaded areas indicate 95% CIs.

IRs of any diagnosed psychiatric disorder were lower during pregnancy (eg, pregnancy week 1: IR, 5.50 [95% CI, 4.69-6.31] per 1000 person-years) and the early postpartum period (eg, postpartum week 1: IR, 5.19 [95% CI, 4.41-5.97] per 1000 person-years) than in the corresponding preconception weeks (eg, preconception week 1: IR, 7.00 [95% CI, 5.97-8.04] per 1000 person-years). Across weeks before, during, and after pregnancy, IRs of any paternal psychiatric disorder declined slightly from before conception to early pregnancy and more markedly in later pregnancy, reaching a minimum of 4 per 1000 person-years (pregnancy week 41: IR, 4.01 [95% CI, 2.70-5.33] per 1000 person-years). After childbirth, IRs of any paternal psychiatric disorder increased gradually and returned to preconception levels (eg, postpartum week 45: IR, 6.18 [95% CI, 5.30-7.06] per 1000 person-years). Depression, anxiety, stress-related disorder, alcohol use disorder, and drug use disorder showed similar trends in IRs, with IRs of depression and stress-related disorder slightly exceeding preconception levels toward the end of the first postpartum year. IRs of tobacco use disorder, ADHD, bipolar disorder, and psychosis remained relatively stable, although IRs of ADHD and psychosis showed slight decreases in late pregnancy (Figure 2 and eFigure 2 in Supplement 1, with estimated IRs provided in eTable 3 in Supplement 1).

**Figure 3.**
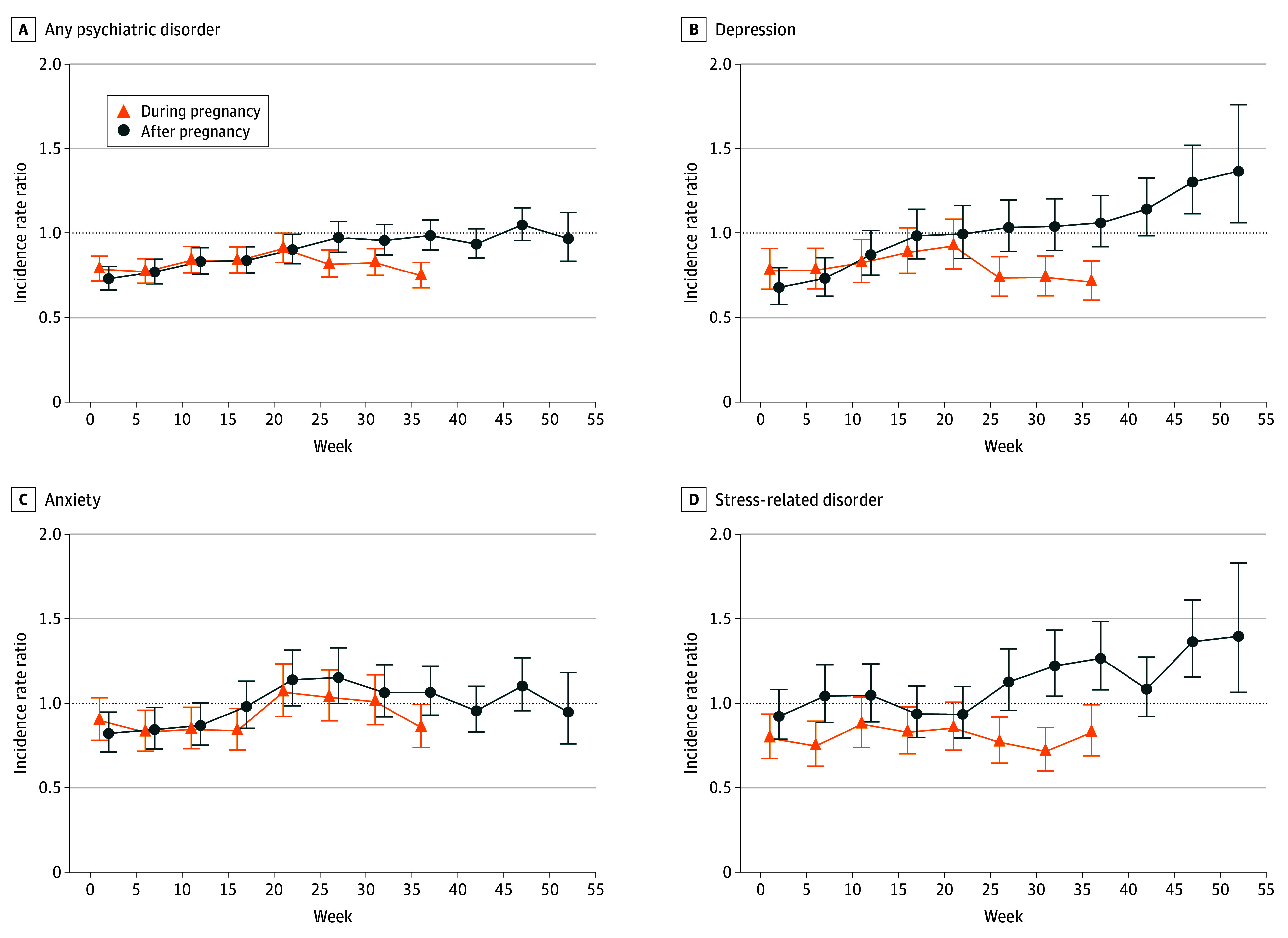
Line Graphs of Incidence Rate Ratios of Any and Common Paternal Psychiatric Disorders During and After Pregnancy Incidence rate ratios were estimated per every 5 weeks by comparing the incidence in each interval during and after pregnancy with that before pregnancy. The model was adjusted for age, calendar year at childbirth and week at follow-up, paternal country of birth, region of residence, education level and annual income before pregnancy, season, civil status during pregnancy, multiple gestations, number of children, and history of psychiatric disorder. Error bars indicate 95% CIs.

Compared with corresponding preconception weeks, IRRs of any paternal psychiatric disorder increased during early pregnancy, approaching unity around gestational week 20 (pregnancy weeks 20-24: IRR, 0.91 [95% CI, 0.83-1.00]) and gradually declined thereafter. In the postpartum period, the IRR of any paternal psychiatric disorder rose from a lower level to a comparable level (eg, postpartum weeks 45-49: IRR, 1.05 [95% CI, 0.95-1.15]). Similar incidence patterns were observed for anxiety, alcohol use disorder, and drug use disorder. IRRs of depression and stress-related disorders were more than 30% higher toward the end of the first postpartum year (postpartum weeks 45-49: IRR, 1.30 [95% CI, 1.12-1.52] for depression and 1.36 [95% CI, 1.15-1.61] for stress-related disorders). IRRs of tobacco use disorder, ADHD, bipolar disorder, and psychosis were generally comparable to the preconception period (Figure 3 and eFigures 3 and 4 in Supplement 1, with estimated IRRs provided in eTables 4 and 5 in Supplement 1).

Sensitivity analyses restricted to individuals without a history of any other psychiatric disorders, to Stockholm County (where both primary and specialist care data were available), to births with a complete 1-year preconception period, or to first births yielded similar results. However, IRRs were slightly higher than in the main analysis (eg, pregnancy weeks 0-4: IRR, 0.79 [95% CI, 0.72-0.86] in the main analysis; IRR, 0.93 [95% CI, 0.83-1.04] in the sensitivity analysis restricting to Stockholm) (eFigures 5-8 in Supplement 1, with estimated IRRs provided in eTables 5-8 in Supplement 1).

In the stratified analysis, fathers with lower educational attainment had higher IRs of any psychiatric disorder before, during, and after pregnancy (eg, postpartum week 1: IR, 10.85 [95% CI, 7.33-14.38] per 1000 person-years for educational level <10 years; IR, 5.09 [95% CI, 3.95-6.23] per 1000 person-years for educational level 10-12 years), while IRRs were similar across strata. Year of childbirth, country of birth, and number of children did not significantly influence either IRs or IRRs (eFigures 9 and 10 in Supplement 1, with estimated IRs and IRRs provided in eTables 9 and 10 in Supplement 1).

## Discussion

In this nationwide cohort study of 1 096 198 fathers and 1 915 722 childbirths in Sweden, IRs of any paternal psychiatric disorder declined from before conception to childbirth and increased gradually post partum. IRs of any paternal psychiatric disorder were lower in the pregnancy and early postpartum periods than in the corresponding preconception weeks, whereas later post partum showed comparable results. Similar incidence patterns were observed for IRRs of anxiety, alcohol use, and drug use disorders, while the IRRs of depression and stress-related disorders showed a notable 30% increase 1 year after childbirth. In contrast, IRRs of being diagnosed with tobacco use disorder, ADHD, bipolar disorder, or psychosis remained relatively stable throughout the perinatal period.

This study is, to our knowledge, the first to report IRs of overall paternal psychiatric disorders before conception. The observed decline in preconception IRs may be explained by a lower likelihood of men with psychiatric disorders to engage in pregnancy planning.^29^ Additionally, motivated by a desire for fatherhood or societal expectations, some fathers deliberately alter their lifestyle and behaviors^30^ or avoid seeking mental health care,^31,32^ leading to decreased rates of diagnosed psychiatric disorders in the year before pregnancy. A previous study reported that with rising infertility rates, more couples had contact with preconception health care services.^33^ Although increased psychiatric symptoms have been documented among prospective mothers,^34^ paternal risk remains poorly understood and not screened in clinical practice. Therefore, future research is warranted to understand the mental health risk among prospective fathers.

In this study, IRs of paternal psychiatric disorders decreased further after conception, remaining lower than before conception. Although IRs increased gradually after childbirth, they stayed below preconception levels during the first 20 weeks post partum and became comparable thereafter. This trend is largely consistent with a previous Danish study based on monthly incidence before and after childbirth.^19^ These findings may indicate a potential mental health benefit associated with transitioning into fatherhood, such as enhanced paternal accomplishment^32^ and strengthened partner relationships.^35^ Alternatively, reduced detection and help-seeking around pregnancy may partly explain the decline, because some fathers may minimize or internalize their own mental health symptoms or decrease help-seeking during their partner’s pregnancy to avoid diverting attention away from maternal needs.^36,37^ Moreover, fathers may face structural barriers within maternity-focused perinatal health care,^38^ with limited tailored information available from health care professionals.^32^ Our study currently cannot distinguish true changes in IRs from reduced detection or help-seeking. Biological mechanisms, including reduced testosterone levels during pregnancy^39^ and increased prolactin after childbirth,^40^ have also been proposed but could not be evaluated within this study.

For depression, a French study constructed long-term trajectories of prevalence during the transition to fatherhood, showing a steady increase from pregnancy until stabilization after childbirth, with rates substantially higher than in nonfathers.^12^ However, that study relied on voluntary participants and self-reported depression rather than clinical diagnosis, and prevalence does not directly inform incident onset. Our study demonstrated a reduction in incident depression from pregnancy through the early postpartum period, followed by an increase over the first year post partum that exceeded preconception levels. This trend may be due to insidious onset^41,42^ and the accumulation of work- and parenting-related stress over time.^43,44^

In this study, the incidence of being diagnosed with stress-related disorders was higher in the late postpartum period compared with corresponding preconception weeks. Although evidence on paternal stress-related disorders during perinatal period remains limited, existing research has indicated that postnatal posttraumatic stress disorder in fathers may persist or worsen without intervention.^45^ These patterns underscore the importance of extended screening and support surveillance efforts for paternal mental health during the postpartum period.

Compared with mothers whose IRs of psychiatric disorders, particularly depression and psychosis, commonly exhibited a pronounced peak in the early postpartum period,^46^ we did not observe any similar peak among fathers. While maternal and paternal depression risks are correlated and their co-occurrence is associated with adverse child outcomes,^47,48^ our findings highlight distinct temporal patterns between parents. This difference is consistent with hypotheses that hormonal fluctuations around childbirth play a role in driving maternal risk of psychiatric disorders.^49^

In addition, our study found that fathers with lower educational attainment consistently exhibited higher IRs of any psychiatric disorders before, during, and after a partner’s pregnancy. Socioeconomic disadvantage, including financial instability and limited health care resources, likely contributes to the mental health burden among this population.^50^ This finding highlights the need for targeted perinatal mental health support and interventions for fathers facing disadvantages to promote health equity.^8^

Regarding secular trends, the observed increase in IRs of paternal psychiatric disorders prior to 2013 may be partly attributable to improved coverage in the NPR over time.^51^ It is also possible that improved mental health destigmatization^52^ and greater access to health care^53^ result in better detection of mental health problems. As such, enhanced early diagnosis and intervention may prevent subclinical symptoms from progressing to diagnosable conditions,^54^ explaining the subsequent decline in IRs in later years. ADHD was the only subtype with a continuous rise in IRs between 2003 and 2021, consistent with trends in the nonperinatal population.^55,56^ Because ADHD is considered a lifetime condition, perinatal diagnoses likely reflect the recognition and identification of preexisting symptoms rather than new onset, possibly driven by increased awareness of adult ADHD.^27^

### Strengths and Limitations

Our study has several strengths. First, we used large-scale, high-quality, prospectively collected data from Swedish national registers covering all identifiable fathers for nearly 2 decades, which enabled investigation of both common and less prevalent psychiatric disorders. Additionally, misclassification of the preconception exposure window was minimized by estimating the date of conception from delivery dates and gestational age.

Some limitations also should be acknowledged. First, paternal information was missed for 100 766 births (5.0%), and whether these fathers differed in incidence of psychiatric disorders remains unknown. Second, the IR estimates may have been affected by left truncation of psychiatric history predating register coverage^57^ and undetected disorders among fathers who did not seek care. Third, the main analyses relied on the NPR, which may capture more severe disorders. However, the sensitivity analysis incorporating primary care data in Stockholm County, accounting for approximately 25% of the study population, yielded comparable IRRs, indicating that this did not materially influence the observed temporal patterns. Fourth, psychiatric history may be underestimated due to incomplete outpatient coverage before 2001; however, results were similar for births occurring from 2010 onward, when history could be more comprehensively ascertained. Fifth, given Sweden’s high-quality, tax-funded health care system, generalizability to settings with different health care structures may be limited. Finally, we did not examine symptom exacerbation among fathers with a prior psychiatric disorder, which should be explored in future research.

## Conclusions

In this nationwide cohort study in Sweden, fathers had a lower incidence of being diagnosed with psychiatric disorders during pregnancy and early post partum, which became comparable to preconception levels later post partum. Notably, the incidence of being diagnosed with depression and stress-related disorders increased toward the end of the first postpartum year. While these patterns may reflect a protective effect associated with the transition to fatherhood, they may also partly arise from delayed detection driven by reduced help-seeking and underrecognition during this period. Our findings highlight the need for ongoing paternal mental health surveillance, particularly for depression and stress-related disorders in the late postpartum period.

## Notes

Supplement 1. **eFigure 1.** Standardized Incidence Rates of Paternal Alcohol Use, Tobacco Use, Drug Use, Attention-Deficit/Hyperactivity, Bipolar, and Psychotic Disorders Before, During, and After Pregnancy, 2003-2022 **eFigure 2.** Standardized Incidence Rates of Paternal Alcohol Use, Tobacco Use, Drug Use, Attention-Deficit/Hyperactivity, Bipolar, and Psychotic Disorders Before, During, and After Pregnancy, by Week **eFigure 3.** Incidence Rate Ratios of Paternal Alcohol Use, Tobacco Use, Drug Use, Attention-Deficit/Hyperactivity, Bipolar, and Psychotic Disorders During and After Pregnancy **eFigure 4.** Incidence Rate Ratios of Any Paternal Psychiatric Disorder and 9 Type-Specific Disorders During and After Pregnancy **eFigure 5.** Incidence Rate Ratios of 9 Type-Specific Paternal Psychiatric Disorders During and After Pregnancy, Excluding Fathers With a History of Any Other Psychiatric Disorder **eFigure 6.** Incidence Rate Ratios of Any Paternal Psychiatric Disorder and 9 Type-Specific Disorders During and After Pregnancy, Restricted to Stockholm Where Both Primary Care and Specialist Care Data Were Available **eFigure 7.** Incidence Rate Ratios of Any Paternal Psychiatric Disorder and 9 Type-Specific Disorders During and After Pregnancy, Restricted to Childbirths With Complete 1-Year Preconception Follow-Up **eFigure 8.** Incidence Rate Ratios of Any Paternal Psychiatric Disorder and 9 Type-Specific Disorders During and After Pregnancy Compared With That Before Pregnancy, Including First Childbirths Only **eFigure 9.** Standardized Incidence Rates of Any Paternal Psychiatric Disorder Before, During, and After Pregnancy, Stratified by Year of Childbirth, Education Level, Country of Birth, and Number of Children **eFigure 10.** Incidence Rate Ratios of Any Paternal Psychiatric Disorder During and After Pregnancy, Stratified by Year of Childbirth, Education Level, Country of Birth, and Number of Children **eTable 1.** Classification of Any Psychiatric Disorder and Type-Specific Psychiatric Disorders **eTable 2.** Standardized Incidence Rates of Any Paternal Psychiatric Disorder and 9 Type-Specific Disorders Before, During, and After Pregnancy, 2003-2022 **eTable 3.** Standardized Incidence Rates of Any Paternal Psychiatric Disorder and 9 Type-Specific Disorders Before, During, and After Pregnancy, by Weeks **eTable 4.** Incidence Rate Ratios of Any Paternal Psychiatric Disorder and 9 Type-Specific Disorders During and After Pregnancy **eTable 5.** Incidence Rate Ratios of 9 Type-Specific Paternal Psychiatric Disorders During and After Pregnancy, Excluding Fathers With a History of Any Other Psychiatric Disorder **eTable 6.** Incidence Rate Ratios of Any Paternal Psychiatric Disorder and 9 Type-Specific Disorders During and After Pregnancy, Restricted to Stockholm Where Both Primary Care and Specialist Care Data Were Available **eTable 7.** Incidence Rate Ratios of Any Paternal Psychiatric Disorder and 9 Type-Specific Disorders During and After Pregnancy, Restricted to Childbirths With Complete 1-Year Preconception Follow-Up **eTable 8.** Incidence Rate Ratios of Any Paternal Psychiatric Disorder and 9 Type-Specific Disorders During and After Pregnancy, Including First Childbirths Only **eTable 9.** Standardized Incidence Rates of Any Paternal Psychiatric Disorder Before, During, and After Pregnancy by Weeks, Stratified by Year of Childbirth, Education Level, Country of Birth, and Number of Children **eTable 10.** Incidence Rate Ratios of Any Paternal Psychiatric Disorder During and After Pregnancy, Stratified by Year of Childbirth, Education Level, Country of Birth, and Number of Children

Supplement 2.
Data Sharing Statement
