# Supplementary material for: Psychiatric Disorders Among Fathers in Sweden Before, During, and After Partner Pregnancy
Source: JAMA Netw Open. 2026 Mar 23;9(3):e262725. doi: 10.1001/jamanetworkopen.2026.2725 (PMC13010218; doi:10.1001/jamanetworkopen.2026.2725)
Supplement: Supplement 1. — eFigure 1. Standardized Incidence Rates of Paternal Alcohol Use, Tobacco Use, Drug Use, Attention-Deficit/Hyperactivity, Bipolar, and Psychotic Disorders Before, During, and After Pregnancy, 2003-2022 eFigure 2. Standardized Incidence Rates of Paternal Alcohol Use, Tobacco Use, Drug Use, Attention-Deficit/Hyperactivity, Bipolar, and Psychotic Disorders Before, During, and After Pregnancy, by Week eFigure 3. Incidence Rate Ratios of Paternal Alcohol Use, Tobacco Use, Drug Use, Attention-Deficit/Hyperactivity, Bipolar, and Psychotic Disorders During and After Pregnancy eFigure 4. Incidence Rate Ratios of Any Paternal Psychiatric Disorder and 9 Type-Specific Disorders During and After Pregnancy eFigure 5. Incidence Rate Ratios of 9 Type-Specific Paternal Psychiatric Disorders During and After Pregnancy, Excluding Fathers With a History of Any Other Psychiatric Disorder eFigure 6. Incidence Rate Ratios of Any Paternal Psychiatric Disorder and 9 Type-Specific Disorders During and After Pregnancy, Restricted to Stockholm Where Both Primary Care and Specialist Care Data Were Available eFigure 7. Incidence Rate Ratios of Any Paternal Psychiatric Disorder and 9 Type-Specific Disorders During and After Pregnancy, Restricted to Childbirths With Complete 1-Year Preconception Follow-Up eFigure 8. Incidence Rate Ratios of Any Paternal Psychiatric Disorder and 9 Type-Specific Disorders During and After Pregnancy Compared With That Before Pregnancy, Including First Childbirths Only eFigure 9. Standardized Incidence Rates of Any Paternal Psychiatric Disorder Before, During, and After Pregnancy, Stratified by Year of Childbirth, Education Level, Country of Birth, and Number of Children eFigure 10. Incidence Rate Ratios of Any Paternal Psychiatric Disorder During and After Pregnancy, Stratified by Year of Childbirth, Education Level, Country of Birth, and Number of Children eTable 1. Classification of Any Psychiatric Disorder and Type-Specific Psychiatric Disorders eTable 2. Standardi [file jamanetwopen-e262725-s001.pdf]

Supplementary Online Content

Xiang N, Zhou J, Lin Y, et al. Psychiatric disorders among fathers in Sweden before, during, and after partner pregnancy. *JAMA Netw Open*. 2026;9(3):e262725. doi:10.1001/jamanetworkopen.2026.2725

eFigure 1. Standardized Incidence Rates of Paternal Alcohol Use, Tobacco Use, Drug Use, Attention-Deficit/Hyperactivity, Bipolar, and Psychotic Disorders Before, During, and After Pregnancy, 2003-2022

eFigure 2. Standardized Incidence Rates of Paternal Alcohol Use, Tobacco Use, Drug Use, Attention-Deficit/Hyperactivity, Bipolar, and Psychotic Disorders Before, During, and After Pregnancy, by Week

eFigure 3. Incidence Rate Ratios of Paternal Alcohol Use, Tobacco Use, Drug Use, Attention-Deficit/Hyperactivity, Bipolar, and Psychotic Disorders During and After Pregnancy

eFigure 4. Incidence Rate Ratios of Any Paternal Psychiatric Disorder and 9 Type-Specific Disorders During and After Pregnancy

eFigure 5. Incidence Rate Ratios of 9 Type-Specific Paternal Psychiatric Disorders During and After Pregnancy, Excluding Fathers With a History of Any Other Psychiatric Disorder

eFigure 6. Incidence Rate Ratios of Any Paternal Psychiatric Disorder and 9 Type-Specific Disorders During and After Pregnancy, Restricted to Stockholm Where Both Primary Care and Specialist Care Data Were Available

eFigure 7. Incidence Rate Ratios of Any Paternal Psychiatric Disorder and 9 Type-Specific Disorders During and After Pregnancy, Restricted to Childbirths With Complete 1-Year Preconception Follow-Up

eFigure 8. Incidence Rate Ratios of Any Paternal Psychiatric Disorder and 9 Type-Specific Disorders During and After Pregnancy Compared With That Before Pregnancy, Including First Childbirths Only

eFigure 9. Standardized Incidence Rates of Any Paternal Psychiatric Disorder Before, During, and After Pregnancy, Stratified by Year of Childbirth, Education Level, Country of Birth, and Number of Children

eFigure 10. Incidence Rate Ratios of Any Paternal Psychiatric Disorder During and After Pregnancy, Stratified by Year of Childbirth, Education Level, Country of Birth, and Number of Children

eTable 1. Classification of Any Psychiatric Disorder and Type-Specific Psychiatric Disorders

© 2026 Xiang N et al. *JAMA Network Open*.

eTable 2. Standardized Incidence Rates of Any Paternal Psychiatric Disorder and 9 Type-Specific Disorders Before, During, and After Pregnancy, 2003-2022

eTable 3. Standardized Incidence Rates of Any Paternal Psychiatric Disorder and 9 Type-Specific Disorders Before, During, and After Pregnancy, by Weeks

eTable 4. Incidence Rate Ratios of Any Paternal Psychiatric Disorder and 9 Type-Specific Disorders During and After Pregnancy

eTable 5. Incidence Rate Ratios of 9 Type-Specific Paternal Psychiatric Disorders During and After Pregnancy, Excluding Fathers With a History of Any Other Psychiatric Disorder

eTable 6. Incidence Rate Ratios of Any Paternal Psychiatric Disorder and 9 Type-Specific Disorders During and After Pregnancy, Restricted to Stockholm Where Both Primary Care and Specialist Care Data Were Available

eTable 7. Incidence Rate Ratios of Any Paternal Psychiatric Disorder and 9 Type-Specific Disorders During and After Pregnancy, Restricted to Childbirths With Complete 1-Year Preconception Follow-Up

eTable 8. Incidence Rate Ratios of Any Paternal Psychiatric Disorder and 9 Type-Specific Disorders During and After Pregnancy, Including First Childbirths Only

eTable 9. Standardized Incidence Rates of Any Paternal Psychiatric Disorder Before, During, and After Pregnancy by Weeks, Stratified by Year of Childbirth, Education Level, Country of Birth, and Number of Children

eTable 10. Incidence Rate Ratios of Any Paternal Psychiatric Disorder During and After Pregnancy, Stratified by Year of Childbirth, Education Level, Country of Birth, and Number of Children

This supplementary material has been provided by the authors to give readers additional information about their work.

© 2026 Xiang N et al. *JAMA Network Open*.

eFigure 1. Standardized Incidence Rates of Paternal Alcohol Use, Tobacco Use, Drug Use, Attention-Deficit/Hyperactivity, Bipolar, and Psychotic Disorders Before, During, and After Pregnancy, 2003-2022

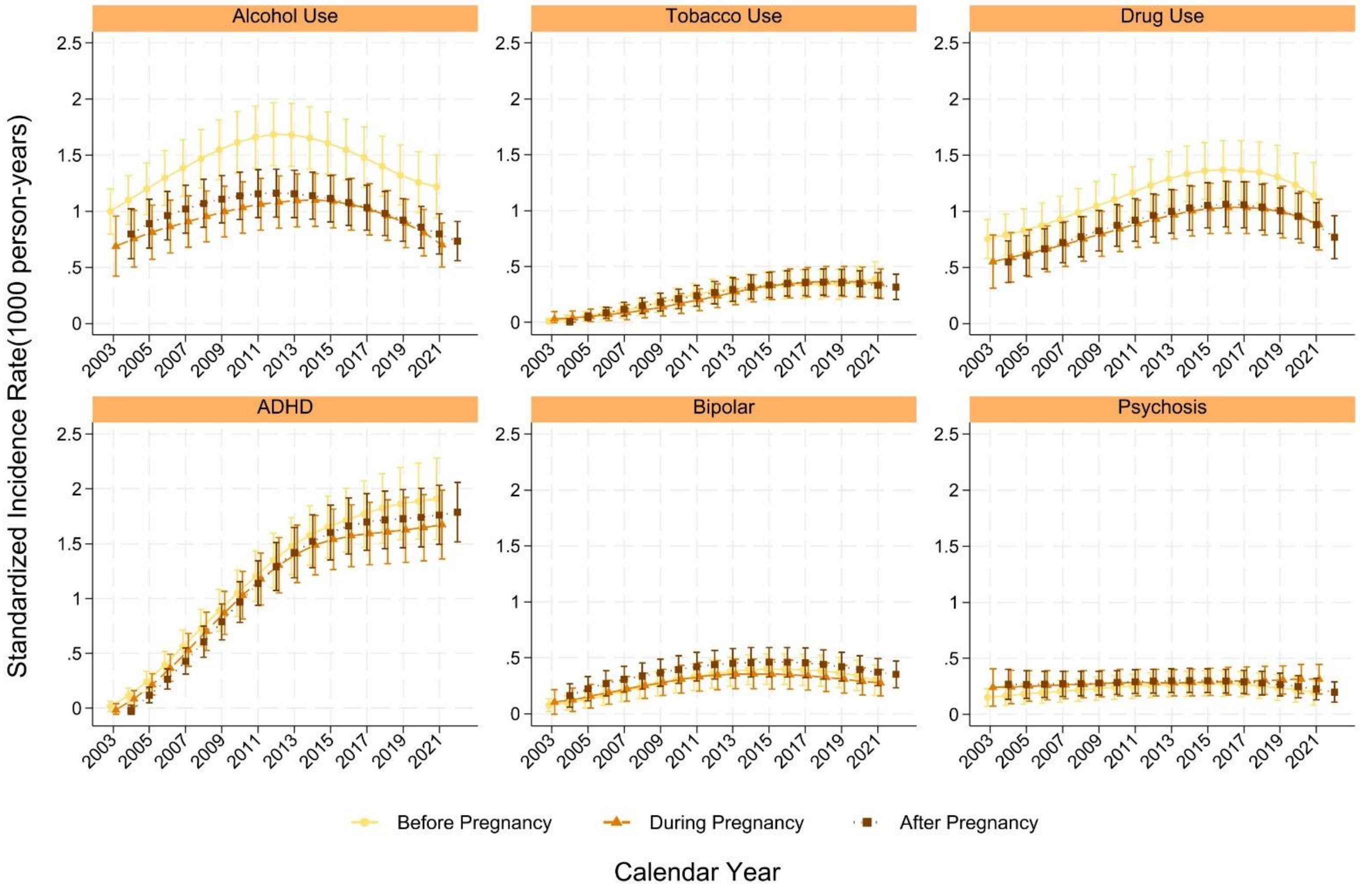

The incidence rate was standardized by age at childbirth. The smoothed trend of standardized incidence rate across years using locally weighted scatterplot smoothing.

© 2026 Xiang N et al. *JAMA Network Open*.

eFigure 2. Standardized Incidence Rates of Paternal Alcohol Use, Tobacco Use, Drug Use, Attention-Deficit/Hyperactivity, Bipolar, and Psychotic Disorders Before, During, and After Pregnancy, by Week

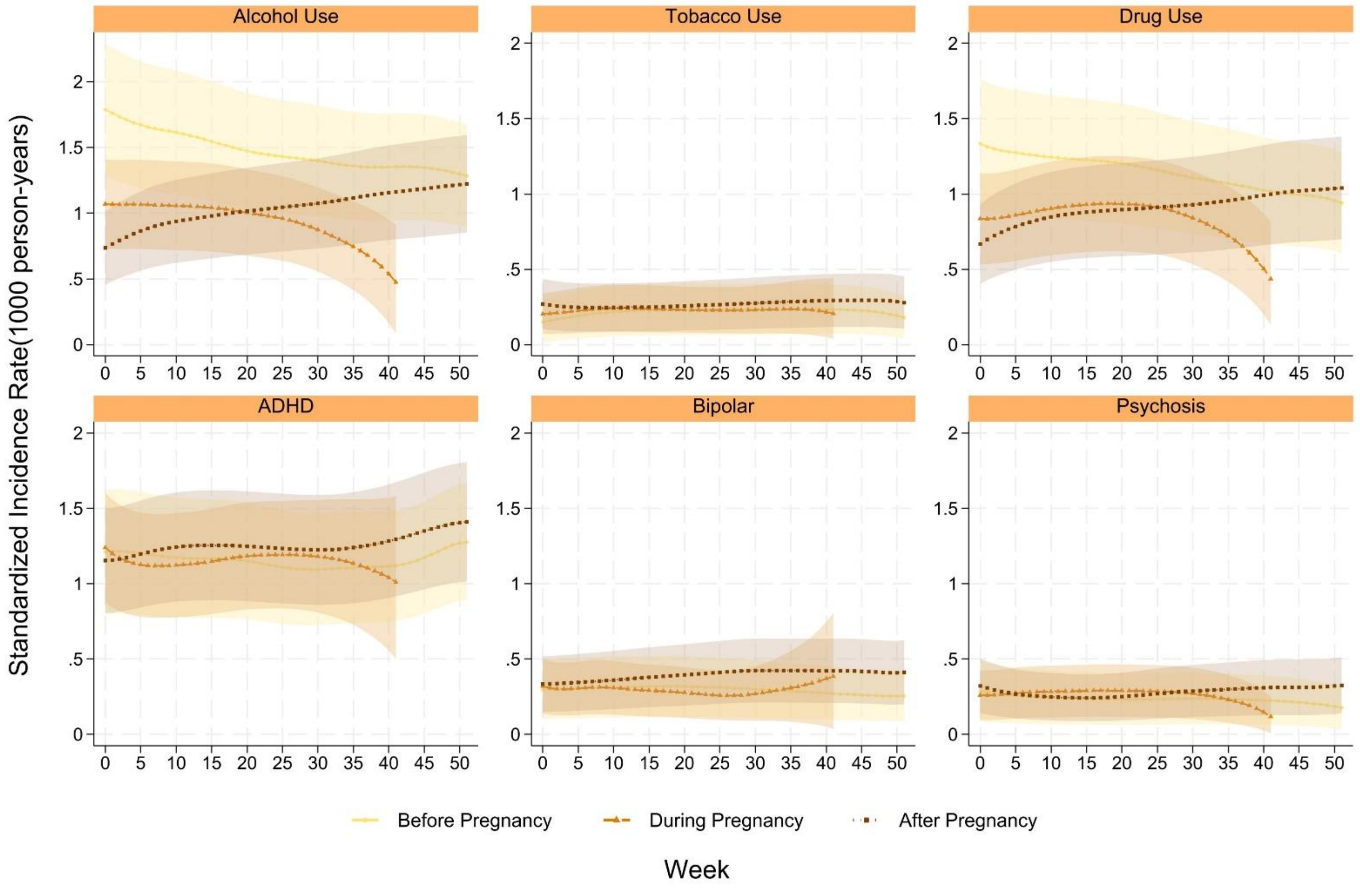

The incidence rate was standardized by age at childbirth. The smoothed trend of standardized incidence rate across years using locally weighted scatterplot smoothing.

© 2026 Xiang N et al. *JAMA Network Open*.

eFigure 3. Incidence Rate Ratios of Paternal Alcohol Use, Tobacco Use, Drug Use, Attention-Deficit/Hyperactivity, Bipolar, and Psychotic Disorders During and After Pregnancy

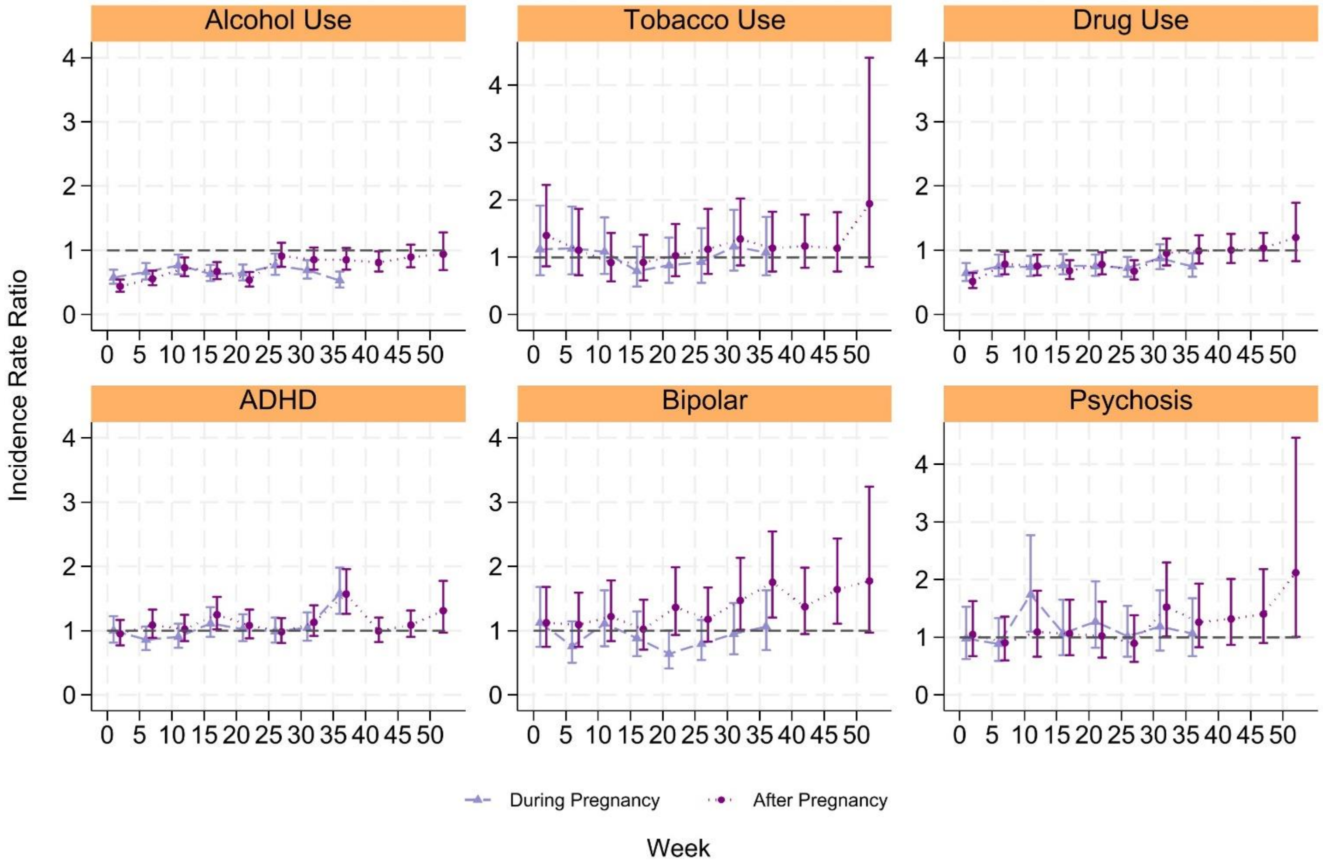

© 2026 Xiang N et al. *JAMA Network Open*.

The incidence rate ratio was estimated by every 5 weeks, by comparing the incidence in each interval during and after pregnancy with that before pregnancy. Model was adjusted for age, calendar year at childbirth and week at follow-up, paternal country of birth, region of residence, education and annual income before pregnancy, season, civil status during pregnancy, multiple gestation, number of children, and history of psychiatric disorder.

© 2026 Xiang N et al. *JAMA Network Open*.

eFigure 4. Incidence Rate Ratios of Any Paternal Psychiatric Disorder and 9 Type-Specific Disorders During and After Pregnancy

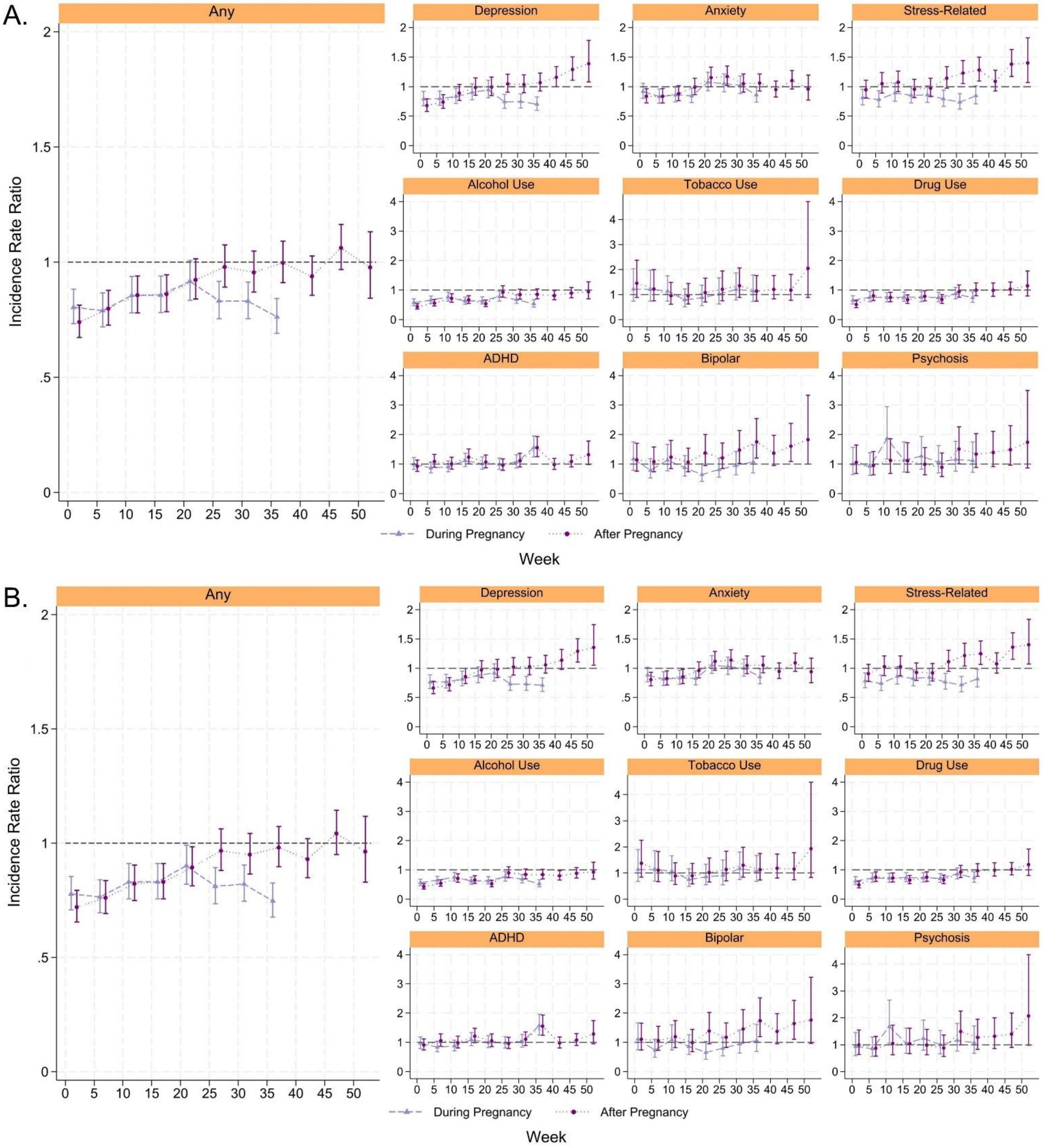

The incidence rate ratio was estimated by every 5 weeks, by comparing the incidence in each interval during and after pregnancy with that before pregnancy. A. Model was adjusted for age, calendar year at childbirth and week at follow-up. B. Model was adjusted for age, calendar year at childbirth and week at follow-up, paternal country of birth, region of residence, education and annual income before pregnancy, and season. The X-axis represents weeks within each period: weeks during pregnancy (gestational weeks), and weeks after pregnancy (counting from childbirth).

© 2026 Xiang N et al. *JAMA Network Open*.

eFigure 5. Incidence Rate Ratios of 9 Type-Specific Paternal Psychiatric Disorders During and After Pregnancy, Excluding Fathers With a History of Any Other Psychiatric Disorder

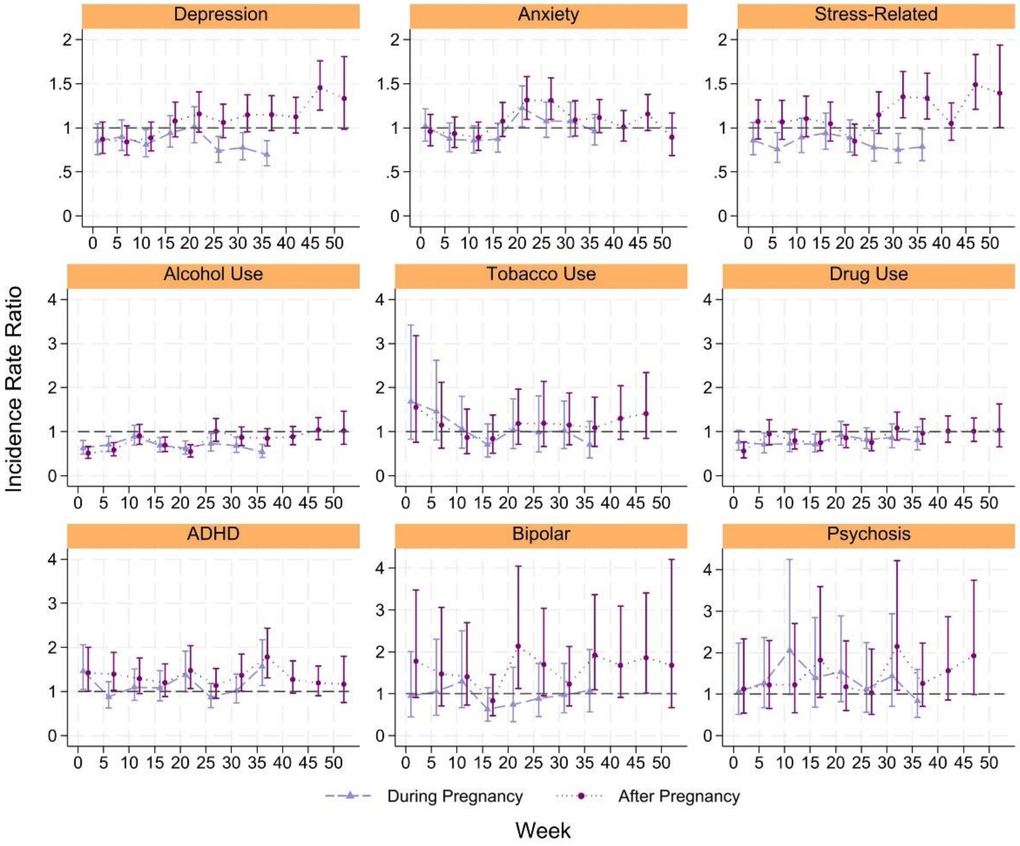

The incidence rate ratio was estimated by every 5 weeks, by comparing the incidence in each interval during and after pregnancy with that before pregnancy. Model was adjusted for age, calendar year at childbirth and week at follow-up, country of birth, region of residence and education before pregnancy, season at childbirth, income before pregnancy, civil status during pregnancy, multiple gestation, number of children, and history of psychiatric disorders. The X-axis represents weeks within each period: weeks during pregnancy (gestational weeks), and weeks after pregnancy (counting from childbirth).

© 2026 Xiang N et al. *JAMA Network Open*.

eFigure 6. Incidence Rate Ratios of Any Paternal Psychiatric Disorder and 9 Type-Specific Disorders During and After Pregnancy, Restricted to Stockholm Where Both Primary Care and Specialist Care Data Were Available

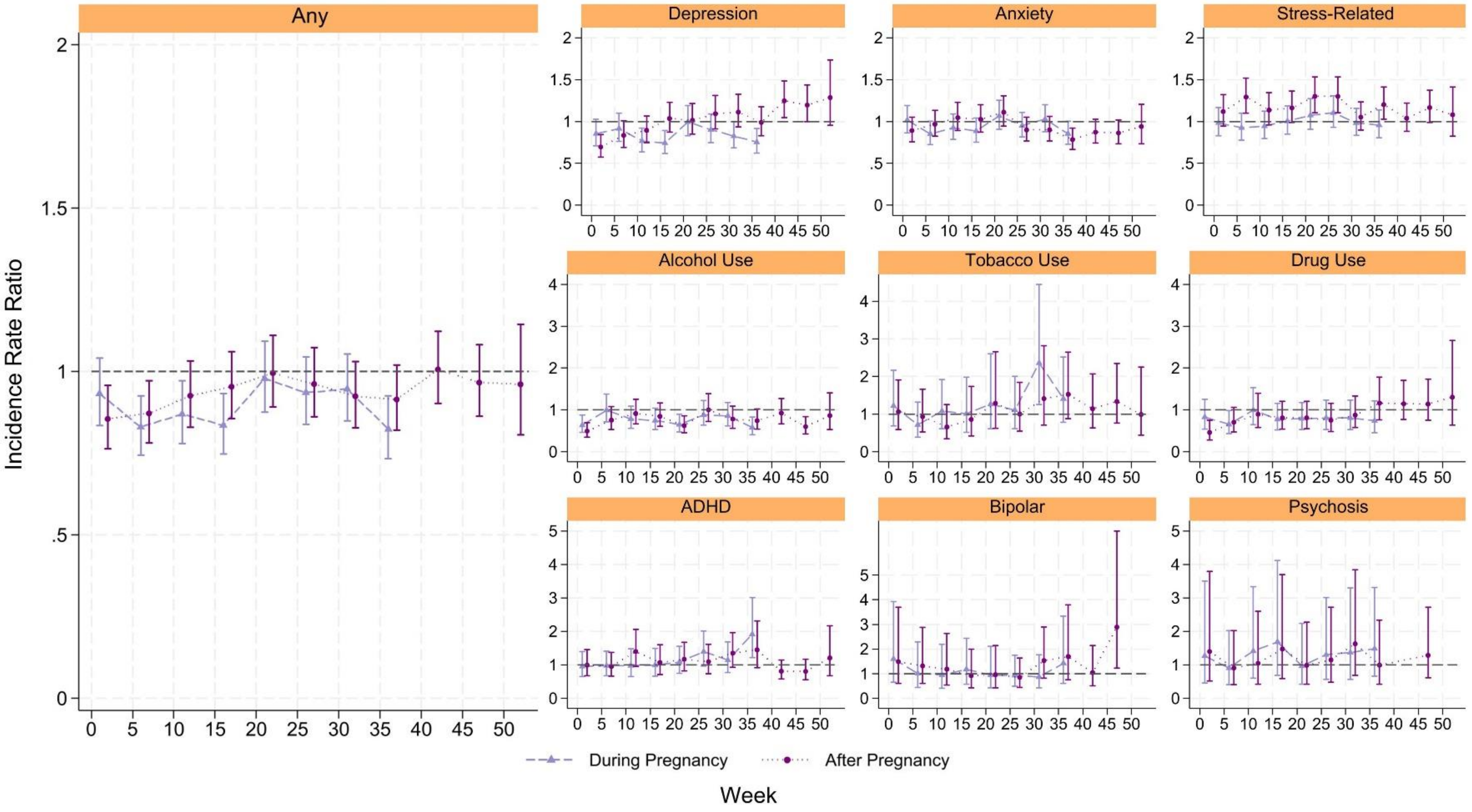

The incidence rate ratio was estimated by every 5 weeks, by comparing the incidence in each interval during and after pregnancy with that before pregnancy. Model was adjusted for age, calendar year at childbirth and week at follow-up, country of birth, region of residence and education before pregnancy, season at childbirth, income before pregnancy, civil status during pregnancy, multiple gestation, number of children, and history of psychiatric disorders. The X-axis represents weeks within each period: weeks during pregnancy (gestational weeks), and weeks after pregnancy (counting from childbirth).

© 2026 Xiang N et al. *JAMA Network Open*.

eFigure 7. Incidence Rate Ratios of Any Paternal Psychiatric Disorder and 9 Type-Specific Disorders During and After Pregnancy, Restricted to Childbirths With Complete 1-Year Preconception Follow-Up

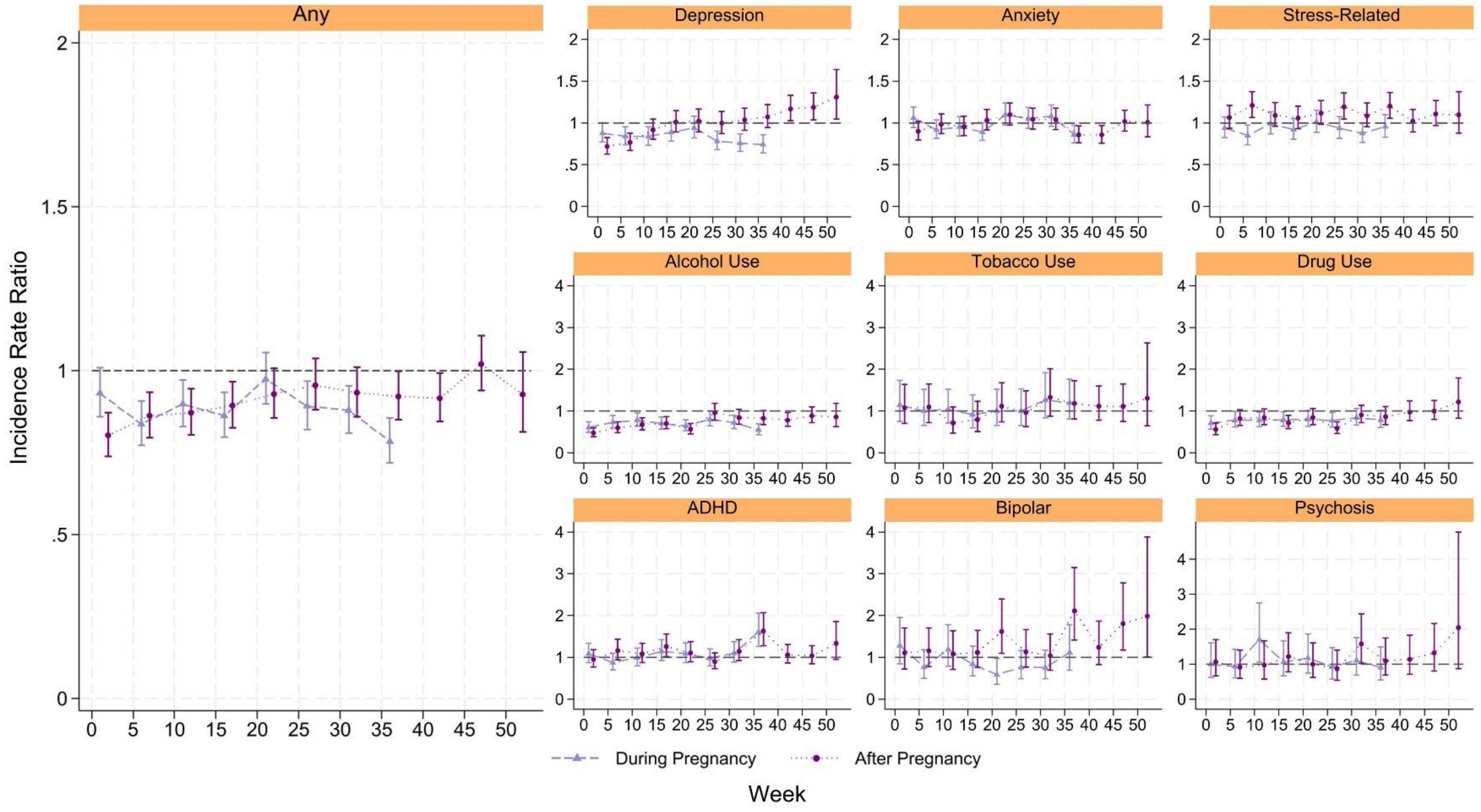

The incidence rate ratio was estimated by every 5 weeks, by comparing the incidence in each interval during and after pregnancy with that before pregnancy. Model was adjusted for age, calendar year at childbirth and week at follow-up, country of birth, region of residence and education before pregnancy, season at childbirth, income before pregnancy, civil status during pregnancy, multiple gestation, number of children, and history of psychiatric disorders. The X-axis represents weeks within each period: weeks during pregnancy (gestational weeks), and weeks after pregnancy (counting from childbirth).

© 2026 Xiang N et al. *JAMA Network Open*.

eFigure 8. Incidence Rate Ratios of Any Paternal Psychiatric Disorder and 9 Type-Specific Disorders During and After Pregnancy Compared With That Before Pregnancy, Including First Childbirths Only

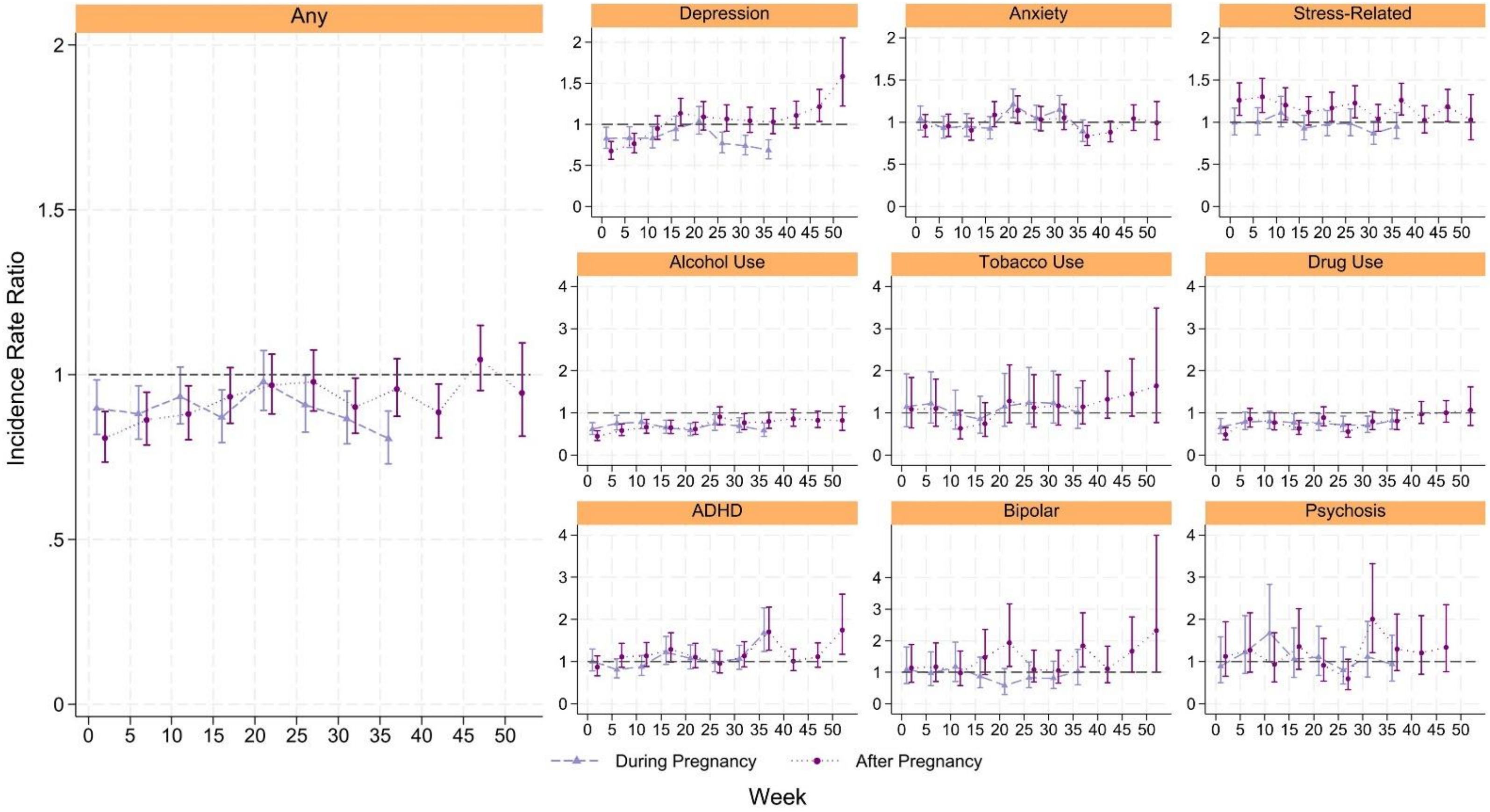

The incidence rate ratio was estimated by every 5 weeks, by comparing the incidence in each interval during and after pregnancy with that before pregnancy. Model was adjusted for age, calendar year at childbirth and week at follow-up, country of birth, region of residence and education before pregnancy, season at childbirth, income before pregnancy, civil status during pregnancy, multiple gestation, number of children, and history of psychiatric disorders. The X-axis represents weeks within each period: weeks during pregnancy (gestational weeks), and weeks after pregnancy (counting from childbirth).

© 2026 Xiang N et al. *JAMA Network Open*.

eFigure 9. Standardized Incidence Rates of Any Paternal Psychiatric Disorder Before, During, and After Pregnancy, Stratified by Year of Childbirth, Education Level, Country of Birth, and Number of Children

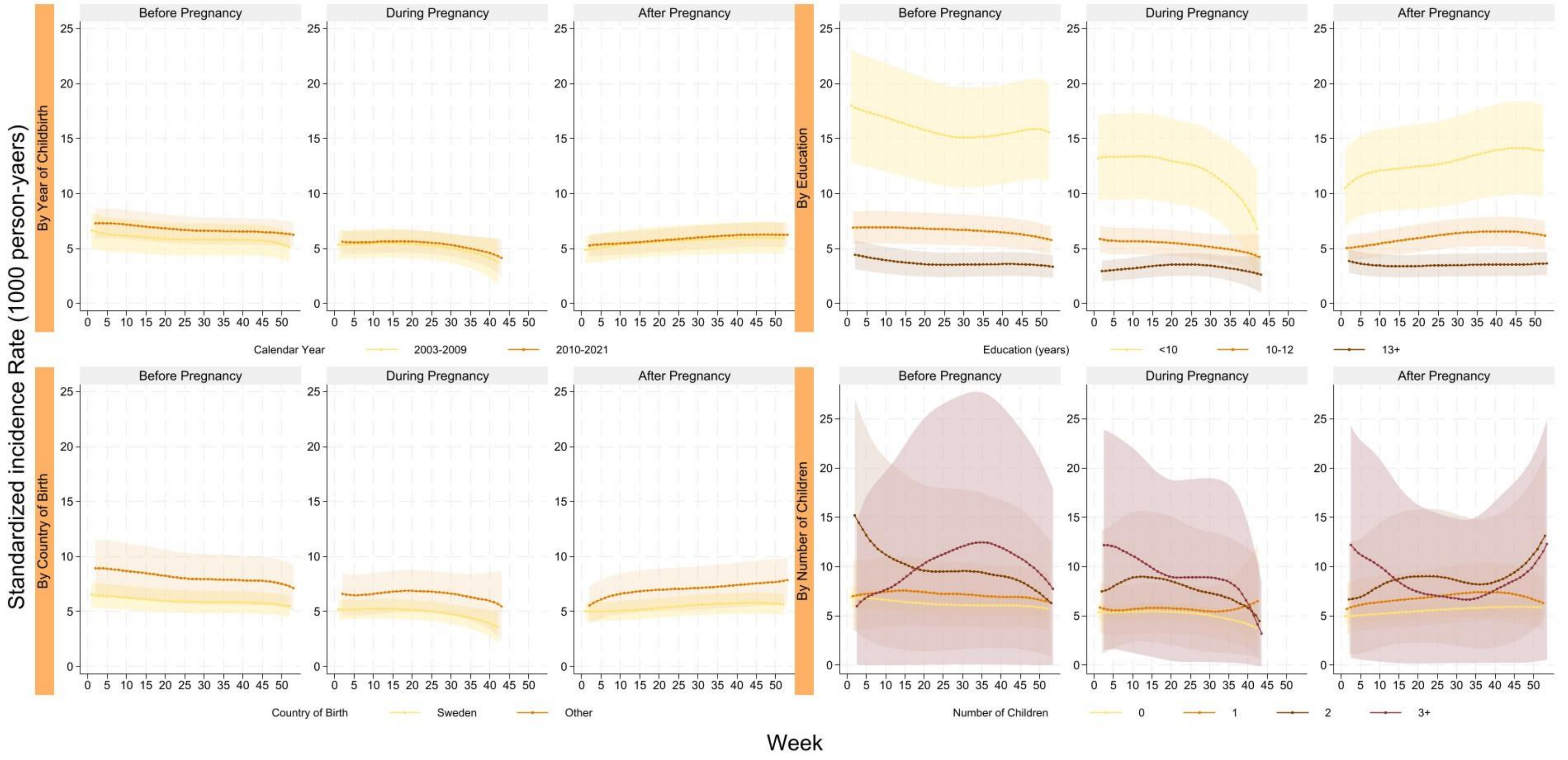

The incidence rate was standardized by age and calendar year at childbirth. The smoothed trend of standardized incidence rate across weeks using locally weighted scatterplot smoothing.

© 2026 Xiang N et al. *JAMA Network Open*.

eFigure 10. Incidence Rate Ratios of Any Paternal Psychiatric Disorder During and After Pregnancy, Stratified by Year of Childbirth, Education Level, Country of Birth, and Number of Children

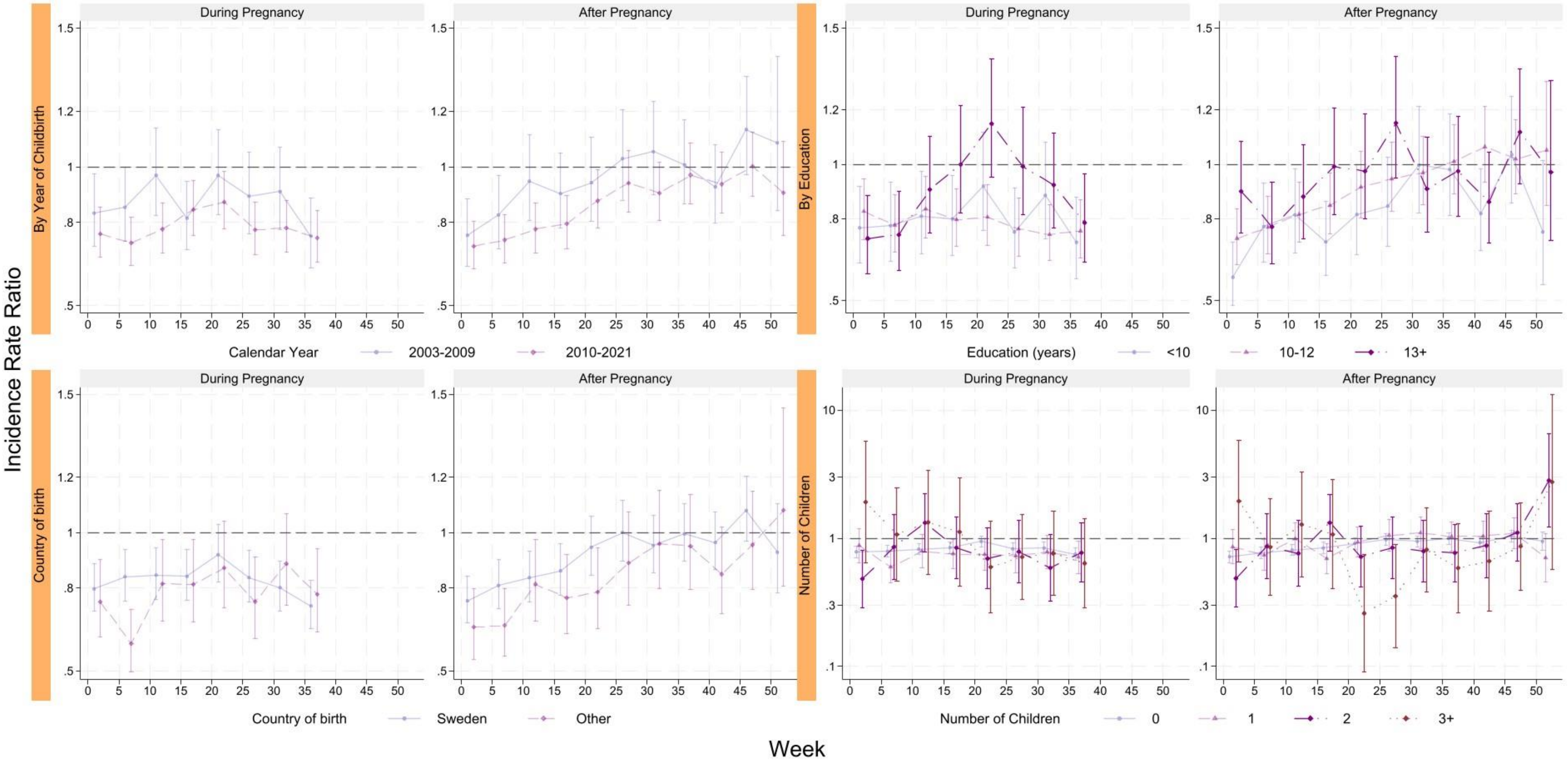

The incidence rate ratio was estimated by every 5 weeks, by comparing the incidence in each interval during and after pregnancy with that before pregnancy. Model was adjusted for age, calendar year at childbirth and week at follow-up, country of birth, region of residence and education before pregnancy, season at childbirth, income before pregnancy, civil status during pregnancy, multiple gestation, number of children, and history of psychiatric disorders.

© 2026 Xiang N et al. *JAMA Network Open*.

eTable 1. Classification of Any Psychiatric Disorder and Type-Specific Psychiatric Disorders

	**ICD 10**
Any psychiatric disorder	F10-F99, except F50, F60, F61, F62 and F69
Depressive disorder	F32-F39, F53.0
Anxiety disorder	F40-F41
Stress-related disorder	F43
Alcohol use disorders	F10
Tobacco use disorder	F17
Drug use disorders	F11- F19, except F17
ADHD	F90
Bipolar disorder	F30-F31
Psychosis	F20-F25, F28-F29

ADHD, attention deficit hyperactivity disorder; ICD, International Classification of Diseases

© 2026 Xiang N et al. *JAMA Network Open*.

eTable 2. Standardized Incidence Rates of Any Paternal Psychiatric Disorder and 9 Type-Specific Disorders Before, During, and After Pregnancy, 2003-2022

Psychiatric disorders	Phase	Year	Standardized incidence rate per 1000 person-years (95%CI)
Any psychiatric disorder	Before pregnancy	2003	4.64(4.20 to 5.07)
Any psychiatric disorder	Before pregnancy	2004	5.16(4.68 to 5.63)
Any psychiatric disorder	Before pregnancy	2005	5.57(5.07 to 6.08)
Any psychiatric disorder	Before pregnancy	2006	5.91(5.38 to 6.44)
Any psychiatric disorder	Before pregnancy	2007	6.17(5.62 to 6.72)
Any psychiatric disorder	Before pregnancy	2008	6.40(5.84 to 6.96)
Any psychiatric disorder	Before pregnancy	2009	6.61(6.04 to 7.19)
Any psychiatric disorder	Before pregnancy	2010	6.80(6.21 to 7.38)
Any psychiatric disorder	Before pregnancy	2011	6.94(6.34 to 7.53)
Any psychiatric disorder	Before pregnancy	2012	7.03(6.43 to 7.63)
Any psychiatric disorder	Before pregnancy	2013	7.06(6.46 to 7.67)
Any psychiatric disorder	Before pregnancy	2014	7.06(6.45 to 7.67)
Any psychiatric disorder	Before pregnancy	2015	7.01(6.40 to 7.62)
Any psychiatric disorder	Before pregnancy	2016	6.92(6.30 to 7.53)
Any psychiatric disorder	Before pregnancy	2017	6.76(6.14 to 7.38)
Any psychiatric disorder	Before pregnancy	2018	6.54(5.91 to 7.16)
Any psychiatric disorder	Before pregnancy	2019	6.26(5.63 to 6.89)
Any psychiatric disorder	Before pregnancy	2020	5.96(5.32 to 6.61)
Any psychiatric disorder	Before pregnancy	2021	5.69(5.02 to 6.35)
Any psychiatric disorder	During pregnancy	2003	3.97(3.33 to 4.61)
Any psychiatric disorder	During pregnancy	2004	4.42(3.82 to 5.03)
Any psychiatric disorder	During pregnancy	2005	4.76(4.18 to 5.34)
Any psychiatric disorder	During pregnancy	2006	5.00(4.44 to 5.57)
Any psychiatric disorder	During pregnancy	2007	5.19(4.63 to 5.74)
Any psychiatric disorder	During pregnancy	2008	5.35(4.80 to 5.90)
Any psychiatric disorder	During pregnancy	2009	5.50(4.94 to 6.05)
Any psychiatric disorder	During pregnancy	2010	5.62(5.06 to 6.17)
Any psychiatric disorder	During pregnancy	2011	5.70(5.14 to 6.26)
Any psychiatric disorder	During pregnancy	2012	5.76(5.19 to 6.32)
Any psychiatric disorder	During pregnancy	2013	5.77(5.21 to 6.34)
Any psychiatric disorder	During pregnancy	2014	5.76(5.19 to 6.33)
Any psychiatric disorder	During pregnancy	2015	5.72(5.15 to 6.28)
Any psychiatric disorder	During pregnancy	2016	5.63(5.07 to 6.20)
Any psychiatric disorder	During pregnancy	2017	5.51(4.94 to 6.07)
Any psychiatric disorder	During pregnancy	2018	5.33(4.77 to 5.89)
Any psychiatric disorder	During pregnancy	2019	5.11(4.56 to 5.67)
Any psychiatric disorder	During pregnancy	2020	4.88(4.33 to 5.43)
Any psychiatric disorder	During pregnancy	2021	4.70(4.15 to 5.25)
Any psychiatric disorder	After pregnancy	2004	4.71(4.16 to 5.26)

© 2026 Xiang N et al. *JAMA Network Open*.

Any psychiatric disorder	After pregnancy	2005	5.08(4.55 to 5.61)
Any psychiatric disorder	After pregnancy	2006	5.34(4.81 to 5.86)
Any psychiatric disorder	After pregnancy	2007	5.51(5.00 to 6.02)
Any psychiatric disorder	After pregnancy	2008	5.65(5.14 to 6.15)
Any psychiatric disorder	After pregnancy	2009	5.78(5.28 to 6.28)
Any psychiatric disorder	After pregnancy	2010	5.89(5.39 to 6.40)
Any psychiatric disorder	After pregnancy	2011	5.99(5.48 to 6.49)
Any psychiatric disorder	After pregnancy	2012	6.06(5.55 to 6.57)
Any psychiatric disorder	After pregnancy	2013	6.12(5.60 to 6.63)
Any psychiatric disorder	After pregnancy	2014	6.16(5.64 to 6.67)
Any psychiatric disorder	After pregnancy	2015	6.17(5.66 to 6.69)
Any psychiatric disorder	After pregnancy	2016	6.14(5.62 to 6.66)
Any psychiatric disorder	After pregnancy	2017	6.06(5.54 to 6.58)
Any psychiatric disorder	After pregnancy	2018	5.91(5.39 to 6.42)
Any psychiatric disorder	After pregnancy	2019	5.70(5.19 to 6.21)
Any psychiatric disorder	After pregnancy	2020	5.44(4.93 to 5.95)
Any psychiatric disorder	After pregnancy	2021	5.11(4.61 to 5.62)
Any psychiatric disorder	After pregnancy	2022	4.73(4.24 to 5.22)
Depression	Before pregnancy	2003	1.40(1.16 to 1.64)
Depression	Before pregnancy	2004	1.64(1.38 to 1.91)
Depression	Before pregnancy	2005	1.86(1.57 to 2.14)
Depression	Before pregnancy	2006	2.02(1.72 to 2.33)
Depression	Before pregnancy	2007	2.15(1.84 to 2.47)
Depression	Before pregnancy	2008	2.25(1.93 to 2.58)
Depression	Before pregnancy	2009	2.34(2.00 to 2.67)
Depression	Before pregnancy	2010	2.40(2.06 to 2.73)
Depression	Before pregnancy	2011	2.43(2.09 to 2.77)
Depression	Before pregnancy	2012	2.44(2.10 to 2.78)
Depression	Before pregnancy	2013	2.42(2.08 to 2.76)
Depression	Before pregnancy	2014	2.38(2.05 to 2.72)
Depression	Before pregnancy	2015	2.33(1.99 to 2.66)
Depression	Before pregnancy	2016	2.26(1.93 to 2.59)
Depression	Before pregnancy	2017	2.17(1.84 to 2.50)
Depression	Before pregnancy	2018	2.07(1.74 to 2.39)
Depression	Before pregnancy	2019	1.94(1.61 to 2.27)
Depression	Before pregnancy	2020	1.80(1.47 to 2.13)
Depression	Before pregnancy	2021	1.66(1.33 to 2.00)
Depression	During pregnancy	2003	1.12(0.80 to 1.45)
Depression	During pregnancy	2004	1.35(1.03 to 1.67)
Depression	During pregnancy	2005	1.54(1.22 to 1.86)
Depression	During pregnancy	2006	1.70(1.38 to 2.02)
Depression	During pregnancy	2007	1.81(1.49 to 2.13)
Depression	During pregnancy	2008	1.90(1.58 to 2.22)
Depression	During pregnancy	2009	1.97(1.65 to 2.29)
Depression	During pregnancy	2010	2.01(1.69 to 2.33)

© 2026 Xiang N et al. *JAMA Network Open*.

Depression	During pregnancy	2011	2.03(1.71 to 2.35)
Depression	During pregnancy	2012	2.02(1.70 to 2.35)
Depression	During pregnancy	2013	1.99(1.67 to 2.31)
Depression	During pregnancy	2014	1.95(1.63 to 2.26)
Depression	During pregnancy	2015	1.89(1.58 to 2.20)
Depression	During pregnancy	2016	1.83(1.53 to 2.14)
Depression	During pregnancy	2017	1.77(1.47 to 2.08)
Depression	During pregnancy	2018	1.70(1.40 to 2.00)
Depression	During pregnancy	2019	1.62(1.33 to 1.92)
Depression	During pregnancy	2020	1.55(1.26 to 1.85)
Depression	During pregnancy	2021	1.52(1.23 to 1.81)
Depression	After pregnancy	2004	1.59(1.28 to 1.91)
Depression	After pregnancy	2005	1.79(1.48 to 2.10)
Depression	After pregnancy	2006	1.94(1.63 to 2.25)
Depression	After pregnancy	2007	2.06(1.75 to 2.36)
Depression	After pregnancy	2008	2.15(1.84 to 2.45)
Depression	After pregnancy	2009	2.21(1.91 to 2.51)
Depression	After pregnancy	2010	2.26(1.96 to 2.56)
Depression	After pregnancy	2011	2.30(1.99 to 2.60)
Depression	After pregnancy	2012	2.31(2.01 to 2.61)
Depression	After pregnancy	2013	2.31(2.01 to 2.61)
Depression	After pregnancy	2014	2.30(1.99 to 2.60)
Depression	After pregnancy	2015	2.27(1.97 to 2.57)
Depression	After pregnancy	2016	2.23(1.93 to 2.53)
Depression	After pregnancy	2017	2.17(1.88 to 2.46)
Depression	After pregnancy	2018	2.09(1.80 to 2.38)
Depression	After pregnancy	2019	1.99(1.71 to 2.28)
Depression	After pregnancy	2020	1.87(1.59 to 2.15)
Depression	After pregnancy	2021	1.72(1.45 to 1.99)
Depression	After pregnancy	2022	1.55(1.29 to 1.81)
Anxiety	Before pregnancy	2003	1.09(0.88 to 1.30)
Anxiety	Before pregnancy	2004	1.35(1.11 to 1.59)
Anxiety	Before pregnancy	2005	1.60(1.34 to 1.87)
Anxiety	Before pregnancy	2006	1.84(1.55 to 2.13)
Anxiety	Before pregnancy	2007	2.06(1.75 to 2.37)
Anxiety	Before pregnancy	2008	2.25(1.93 to 2.58)
Anxiety	Before pregnancy	2009	2.43(2.10 to 2.77)
Anxiety	Before pregnancy	2010	2.60(2.25 to 2.95)
Anxiety	Before pregnancy	2011	2.73(2.37 to 3.09)
Anxiety	Before pregnancy	2012	2.83(2.46 to 3.19)
Anxiety	Before pregnancy	2013	2.88(2.52 to 3.25)
Anxiety	Before pregnancy	2014	2.91(2.54 to 3.28)
Anxiety	Before pregnancy	2015	2.91(2.53 to 3.28)
Anxiety	Before pregnancy	2016	2.88(2.50 to 3.26)
Anxiety	Before pregnancy	2017	2.83(2.45 to 3.22)

© 2026 Xiang N et al. *JAMA Network Open*.

Anxiety	Before pregnancy	2018	2.76(2.38 to 3.15)
Anxiety	Before pregnancy	2019	2.67(2.28 to 3.06)
Anxiety	Before pregnancy	2020	2.57(2.17 to 2.97)
Anxiety	Before pregnancy	2021	2.50(2.08 to 2.91)
Anxiety	During pregnancy	2003	1.30(0.94 to 1.66)
Anxiety	During pregnancy	2004	1.47(1.13 to 1.82)
Anxiety	During pregnancy	2005	1.64(1.31 to 1.98)
Anxiety	During pregnancy	2006	1.80(1.46 to 2.13)
Anxiety	During pregnancy	2007	1.93(1.60 to 2.26)
Anxiety	During pregnancy	2008	2.06(1.73 to 2.39)
Anxiety	During pregnancy	2009	2.19(1.85 to 2.52)
Anxiety	During pregnancy	2010	2.31(1.97 to 2.65)
Anxiety	During pregnancy	2011	2.42(2.07 to 2.77)
Anxiety	During pregnancy	2012	2.50(2.15 to 2.86)
Anxiety	During pregnancy	2013	2.55(2.19 to 2.91)
Anxiety	During pregnancy	2014	2.57(2.21 to 2.93)
Anxiety	During pregnancy	2015	2.56(2.20 to 2.92)
Anxiety	During pregnancy	2016	2.54(2.18 to 2.90)
Anxiety	During pregnancy	2017	2.50(2.14 to 2.86)
Anxiety	During pregnancy	2018	2.43(2.07 to 2.78)
Anxiety	During pregnancy	2019	2.33(1.98 to 2.68)
Anxiety	During pregnancy	2020	2.24(1.89 to 2.59)
Anxiety	During pregnancy	2021	2.20(1.85 to 2.55)
Anxiety	After pregnancy	2004	1.44(1.14 to 1.74)
Anxiety	After pregnancy	2005	1.66(1.37 to 1.96)
Anxiety	After pregnancy	2006	1.86(1.56 to 2.16)
Anxiety	After pregnancy	2007	2.03(1.73 to 2.33)
Anxiety	After pregnancy	2008	2.16(1.86 to 2.46)
Anxiety	After pregnancy	2009	2.28(1.97 to 2.58)
Anxiety	After pregnancy	2010	2.38(2.07 to 2.69)
Anxiety	After pregnancy	2011	2.47(2.16 to 2.78)
Anxiety	After pregnancy	2012	2.55(2.23 to 2.87)
Anxiety	After pregnancy	2013	2.61(2.29 to 2.93)
Anxiety	After pregnancy	2014	2.65(2.32 to 2.97)
Anxiety	After pregnancy	2015	2.67(2.34 to 2.99)
Anxiety	After pregnancy	2016	2.67(2.34 to 3.00)
Anxiety	After pregnancy	2017	2.66(2.33 to 2.98)
Anxiety	After pregnancy	2018	2.62(2.29 to 2.94)
Anxiety	After pregnancy	2019	2.55(2.23 to 2.88)
Anxiety	After pregnancy	2020	2.46(2.14 to 2.78)
Anxiety	After pregnancy	2021	2.34(2.02 to 2.66)
Anxiety	After pregnancy	2022	2.20(1.89 to 2.51)
Stress-related disorder	Before pregnancy	2003	0.92(0.73 to 1.11)
Stress-related disorder	Before pregnancy	2004	1.23(1.00 to 1.46)
Stress-related disorder	Before pregnancy	2005	1.43(1.18 to 1.68)

© 2026 Xiang N et al. *JAMA Network Open*.

Stress-related disorder	Before pregnancy	2006	1.57(1.30 to 1.83)
Stress-related disorder	Before pregnancy	2007	1.66(1.39 to 1.94)
Stress-related disorder	Before pregnancy	2008	1.73(1.45 to 2.02)
Stress-related disorder	Before pregnancy	2009	1.79(1.50 to 2.08)
Stress-related disorder	Before pregnancy	2010	1.83(1.53 to 2.12)
Stress-related disorder	Before pregnancy	2011	1.85(1.55 to 2.14)
Stress-related disorder	Before pregnancy	2012	1.87(1.58 to 2.17)
Stress-related disorder	Before pregnancy	2013	1.90(1.61 to 2.20)
Stress-related disorder	Before pregnancy	2014	1.94(1.64 to 2.24)
Stress-related disorder	Before pregnancy	2015	1.97(1.66 to 2.28)
Stress-related disorder	Before pregnancy	2016	1.99(1.68 to 2.30)
Stress-related disorder	Before pregnancy	2017	1.99(1.68 to 2.31)
Stress-related disorder	Before pregnancy	2018	1.97(1.65 to 2.29)
Stress-related disorder	Before pregnancy	2019	1.91(1.59 to 2.23)
Stress-related disorder	Before pregnancy	2020	1.83(1.50 to 2.15)
Stress-related disorder	Before pregnancy	2021	1.71(1.38 to 2.04)
Stress-related disorder	During pregnancy	2003	0.76(0.48 to 1.04)
Stress-related disorder	During pregnancy	2004	1.01(0.73 to 1.28)
Stress-related disorder	During pregnancy	2005	1.19(0.91 to 1.47)
Stress-related disorder	During pregnancy	2006	1.33(1.05 to 1.62)
Stress-related disorder	During pregnancy	2007	1.43(1.15 to 1.71)
Stress-related disorder	During pregnancy	2008	1.49(1.21 to 1.78)
Stress-related disorder	During pregnancy	2009	1.54(1.26 to 1.83)
Stress-related disorder	During pregnancy	2010	1.58(1.29 to 1.86)
Stress-related disorder	During pregnancy	2011	1.59(1.30 to 1.87)
Stress-related disorder	During pregnancy	2012	1.59(1.30 to 1.87)
Stress-related disorder	During pregnancy	2013	1.58(1.30 to 1.86)
Stress-related disorder	During pregnancy	2014	1.58(1.29 to 1.86)
Stress-related disorder	During pregnancy	2015	1.58(1.29 to 1.86)
Stress-related disorder	During pregnancy	2016	1.58(1.30 to 1.86)
Stress-related disorder	During pregnancy	2017	1.58(1.30 to 1.87)
Stress-related disorder	During pregnancy	2018	1.58(1.29 to 1.86)
Stress-related disorder	During pregnancy	2019	1.56(1.27 to 1.84)
Stress-related disorder	During pregnancy	2020	1.54(1.26 to 1.83)
Stress-related disorder	During pregnancy	2021	1.54(1.25 to 1.83)
Stress-related disorder	After pregnancy	2004	1.38(1.09 to 1.68)
Stress-related disorder	After pregnancy	2005	1.52(1.23 to 1.81)
Stress-related disorder	After pregnancy	2006	1.64(1.35 to 1.92)
Stress-related disorder	After pregnancy	2007	1.73(1.45 to 2.01)
Stress-related disorder	After pregnancy	2008	1.81(1.53 to 2.09)
Stress-related disorder	After pregnancy	2009	1.88(1.60 to 2.16)
Stress-related disorder	After pregnancy	2010	1.94(1.66 to 2.22)
Stress-related disorder	After pregnancy	2011	2.01(1.73 to 2.29)
Stress-related disorder	After pregnancy	2012	2.07(1.78 to 2.35)
Stress-related disorder	After pregnancy	2013	2.12(1.83 to 2.41)

© 2026 Xiang N et al. *JAMA Network Open*.

Stress-related disorder	After pregnancy	2014	2.16(1.87 to 2.46)
Stress-related disorder	After pregnancy	2015	2.18(1.89 to 2.48)
Stress-related disorder	After pregnancy	2016	2.18(1.89 to 2.48)
Stress-related disorder	After pregnancy	2017	2.16(1.87 to 2.45)
Stress-related disorder	After pregnancy	2018	2.12(1.82 to 2.41)
Stress-related disorder	After pregnancy	2019	2.04(1.75 to 2.33)
Stress-related disorder	After pregnancy	2020	1.93(1.65 to 2.21)
Stress-related disorder	After pregnancy	2021	1.79(1.51 to 2.06)
Stress-related disorder	After pregnancy	2022	1.64(1.37 to 1.91)
Alcohol use disorder	Before pregnancy	2003	1.00(0.80 to 1.20)
Alcohol use disorder	Before pregnancy	2004	1.10(0.88 to 1.32)
Alcohol use disorder	Before pregnancy	2005	1.20(0.97 to 1.43)
Alcohol use disorder	Before pregnancy	2006	1.30(1.05 to 1.54)
Alcohol use disorder	Before pregnancy	2007	1.39(1.13 to 1.64)
Alcohol use disorder	Before pregnancy	2008	1.47(1.21 to 1.73)
Alcohol use disorder	Before pregnancy	2009	1.55(1.28 to 1.82)
Alcohol use disorder	Before pregnancy	2010	1.61(1.34 to 1.89)
Alcohol use disorder	Before pregnancy	2011	1.66(1.38 to 1.94)
Alcohol use disorder	Before pregnancy	2012	1.68(1.40 to 1.96)
Alcohol use disorder	Before pregnancy	2013	1.68(1.40 to 1.96)
Alcohol use disorder	Before pregnancy	2014	1.65(1.38 to 1.93)
Alcohol use disorder	Before pregnancy	2015	1.61(1.33 to 1.88)
Alcohol use disorder	Before pregnancy	2016	1.55(1.28 to 1.82)
Alcohol use disorder	Before pregnancy	2017	1.48(1.21 to 1.75)
Alcohol use disorder	Before pregnancy	2018	1.40(1.13 to 1.67)
Alcohol use disorder	Before pregnancy	2019	1.32(1.05 to 1.59)
Alcohol use disorder	Before pregnancy	2020	1.26(0.99 to 1.53)
Alcohol use disorder	Before pregnancy	2021	1.22(0.94 to 1.50)
Alcohol use disorder	During pregnancy	2003	0.69(0.42 to 0.96)
Alcohol use disorder	During pregnancy	2004	0.76(0.50 to 1.01)
Alcohol use disorder	During pregnancy	2005	0.81(0.57 to 1.06)
Alcohol use disorder	During pregnancy	2006	0.86(0.63 to 1.10)
Alcohol use disorder	During pregnancy	2007	0.91(0.68 to 1.14)
Alcohol use disorder	During pregnancy	2008	0.95(0.73 to 1.18)
Alcohol use disorder	During pregnancy	2009	1.00(0.77 to 1.22)
Alcohol use disorder	During pregnancy	2010	1.03(0.80 to 1.26)
Alcohol use disorder	During pregnancy	2011	1.06(0.83 to 1.29)
Alcohol use disorder	During pregnancy	2012	1.08(0.85 to 1.32)
Alcohol use disorder	During pregnancy	2013	1.10(0.86 to 1.33)
Alcohol use disorder	During pregnancy	2014	1.10(0.86 to 1.33)
Alcohol use disorder	During pregnancy	2015	1.09(0.85 to 1.32)
Alcohol use disorder	During pregnancy	2016	1.06(0.83 to 1.29)
Alcohol use disorder	During pregnancy	2017	1.02(0.79 to 1.24)
Alcohol use disorder	During pregnancy	2018	0.96(0.74 to 1.18)
Alcohol use disorder	During pregnancy	2019	0.89(0.68 to 1.11)

© 2026 Xiang N et al. *JAMA Network Open*.

Alcohol use disorder	During pregnancy	2020	0.81(0.60 to 1.02)
Alcohol use disorder	During pregnancy	2021	0.70(0.50 to 0.90)
Alcohol use disorder	After pregnancy	2004	0.80(0.58 to 1.02)
Alcohol use disorder	After pregnancy	2005	0.89(0.67 to 1.11)
Alcohol use disorder	After pregnancy	2006	0.96(0.75 to 1.18)
Alcohol use disorder	After pregnancy	2007	1.02(0.81 to 1.23)
Alcohol use disorder	After pregnancy	2008	1.07(0.86 to 1.28)
Alcohol use disorder	After pregnancy	2009	1.11(0.90 to 1.32)
Alcohol use disorder	After pregnancy	2010	1.14(0.92 to 1.35)
Alcohol use disorder	After pregnancy	2011	1.16(0.94 to 1.37)
Alcohol use disorder	After pregnancy	2012	1.16(0.95 to 1.38)
Alcohol use disorder	After pregnancy	2013	1.16(0.94 to 1.37)
Alcohol use disorder	After pregnancy	2014	1.14(0.93 to 1.35)
Alcohol use disorder	After pregnancy	2015	1.11(0.91 to 1.32)
Alcohol use disorder	After pregnancy	2016	1.08(0.87 to 1.28)
Alcohol use disorder	After pregnancy	2017	1.03(0.83 to 1.23)
Alcohol use disorder	After pregnancy	2018	0.98(0.78 to 1.18)
Alcohol use disorder	After pregnancy	2019	0.92(0.73 to 1.11)
Alcohol use disorder	After pregnancy	2020	0.86(0.67 to 1.04)
Alcohol use disorder	After pregnancy	2021	0.80(0.62 to 0.98)
Alcohol use disorder	After pregnancy	2022	0.73(0.56 to 0.91)
Tobacco use disorder	Before pregnancy	2003	0.01(-0.01 to 0.04)
Tobacco use disorder	Before pregnancy	2004	0.03(-0.00 to 0.07)
Tobacco use disorder	Before pregnancy	2005	0.05(0.01 to 0.10)
Tobacco use disorder	Before pregnancy	2006	0.08(0.03 to 0.14)
Tobacco use disorder	Before pregnancy	2007	0.11(0.04 to 0.17)
Tobacco use disorder	Before pregnancy	2008	0.14(0.06 to 0.21)
Tobacco use disorder	Before pregnancy	2009	0.17(0.09 to 0.26)
Tobacco use disorder	Before pregnancy	2010	0.21(0.12 to 0.31)
Tobacco use disorder	Before pregnancy	2011	0.25(0.14 to 0.35)
Tobacco use disorder	Before pregnancy	2012	0.28(0.17 to 0.39)
Tobacco use disorder	Before pregnancy	2013	0.30(0.18 to 0.41)
Tobacco use disorder	Before pregnancy	2014	0.31(0.20 to 0.43)
Tobacco use disorder	Before pregnancy	2015	0.33(0.20 to 0.45)
Tobacco use disorder	Before pregnancy	2016	0.34(0.21 to 0.46)
Tobacco use disorder	Before pregnancy	2017	0.34(0.21 to 0.47)
Tobacco use disorder	Before pregnancy	2018	0.34(0.21 to 0.47)
Tobacco use disorder	Before pregnancy	2019	0.34(0.21 to 0.48)
Tobacco use disorder	Before pregnancy	2020	0.36(0.21 to 0.50)
Tobacco use disorder	Before pregnancy	2021	0.39(0.23 to 0.54)
Tobacco use disorder	During pregnancy	2003	0.03(-0.00 to 0.10)
Tobacco use disorder	During pregnancy	2004	0.04(-0.00 to 0.10)
Tobacco use disorder	During pregnancy	2005	0.05(0.01 to 0.12)
Tobacco use disorder	During pregnancy	2006	0.07(0.01 to 0.13)
Tobacco use disorder	During pregnancy	2007	0.09(0.03 to 0.16)

© 2026 Xiang N et al. *JAMA Network Open*.

Tobacco use disorder	During pregnancy	2008	0.11(0.04 to 0.19)
Tobacco use disorder	During pregnancy	2009	0.14(0.06 to 0.22)
Tobacco use disorder	During pregnancy	2010	0.17(0.08 to 0.26)
Tobacco use disorder	During pregnancy	2011	0.20(0.11 to 0.30)
Tobacco use disorder	During pregnancy	2012	0.24(0.13 to 0.34)
Tobacco use disorder	During pregnancy	2013	0.27(0.16 to 0.39)
Tobacco use disorder	During pregnancy	2014	0.30(0.18 to 0.42)
Tobacco use disorder	During pregnancy	2015	0.33(0.20 to 0.45)
Tobacco use disorder	During pregnancy	2016	0.35(0.22 to 0.48)
Tobacco use disorder	During pregnancy	2017	0.36(0.23 to 0.49)
Tobacco use disorder	During pregnancy	2018	0.37(0.23 to 0.50)
Tobacco use disorder	During pregnancy	2019	0.37(0.23 to 0.50)
Tobacco use disorder	During pregnancy	2020	0.36(0.23 to 0.49)
Tobacco use disorder	During pregnancy	2021	0.35(0.21 to 0.48)
Tobacco use disorder	After pregnancy	2004	0.01(-0.00 to 0.02)
Tobacco use disorder	After pregnancy	2005	0.05(0.02 to 0.08)
Tobacco use disorder	After pregnancy	2006	0.09(0.04 to 0.13)
Tobacco use disorder	After pregnancy	2007	0.12(0.06 to 0.18)
Tobacco use disorder	After pregnancy	2008	0.15(0.08 to 0.22)
Tobacco use disorder	After pregnancy	2009	0.18(0.10 to 0.26)
Tobacco use disorder	After pregnancy	2010	0.21(0.12 to 0.30)
Tobacco use disorder	After pregnancy	2011	0.24(0.14 to 0.33)
Tobacco use disorder	After pregnancy	2012	0.27(0.17 to 0.37)
Tobacco use disorder	After pregnancy	2013	0.29(0.19 to 0.40)
Tobacco use disorder	After pregnancy	2014	0.32(0.21 to 0.43)
Tobacco use disorder	After pregnancy	2015	0.33(0.22 to 0.45)
Tobacco use disorder	After pregnancy	2016	0.35(0.23 to 0.46)
Tobacco use disorder	After pregnancy	2017	0.36(0.24 to 0.47)
Tobacco use disorder	After pregnancy	2018	0.36(0.24 to 0.48)
Tobacco use disorder	After pregnancy	2019	0.36(0.24 to 0.47)
Tobacco use disorder	After pregnancy	2020	0.35(0.23 to 0.46)
Tobacco use disorder	After pregnancy	2021	0.33(0.22 to 0.45)
Tobacco use disorder	After pregnancy	2022	0.32(0.20 to 0.43)
Drug use disorder	Before pregnancy	2003	0.75(0.58 to 0.93)
Drug use disorder	Before pregnancy	2004	0.79(0.60 to 0.97)
Drug use disorder	Before pregnancy	2005	0.83(0.64 to 1.02)
Drug use disorder	Before pregnancy	2006	0.87(0.67 to 1.07)
Drug use disorder	Before pregnancy	2007	0.93(0.72 to 1.13)
Drug use disorder	Before pregnancy	2008	0.99(0.78 to 1.20)
Drug use disorder	Before pregnancy	2009	1.05(0.83 to 1.27)
Drug use disorder	Before pregnancy	2010	1.10(0.88 to 1.33)
Drug use disorder	Before pregnancy	2011	1.17(0.94 to 1.40)
Drug use disorder	Before pregnancy	2012	1.23(0.99 to 1.47)
Drug use disorder	Before pregnancy	2013	1.29(1.05 to 1.53)
Drug use disorder	Before pregnancy	2014	1.33(1.08 to 1.58)

© 2026 Xiang N et al. *JAMA Network Open*.

Drug use disorder	Before pregnancy	2015	1.36(1.11 to 1.61)
Drug use disorder	Before pregnancy	2016	1.37(1.11 to 1.63)
Drug use disorder	Before pregnancy	2017	1.36(1.10 to 1.63)
Drug use disorder	Before pregnancy	2018	1.35(1.08 to 1.62)
Drug use disorder	Before pregnancy	2019	1.31(1.03 to 1.58)
Drug use disorder	Before pregnancy	2020	1.24(0.95 to 1.52)
Drug use disorder	Before pregnancy	2021	1.14(0.85 to 1.44)
Drug use disorder	During pregnancy	2003	0.55(0.31 to 0.79)
Drug use disorder	During pregnancy	2004	0.59(0.37 to 0.81)
Drug use disorder	During pregnancy	2005	0.63(0.42 to 0.84)
Drug use disorder	During pregnancy	2006	0.66(0.46 to 0.87)
Drug use disorder	During pregnancy	2007	0.71(0.51 to 0.91)
Drug use disorder	During pregnancy	2008	0.75(0.55 to 0.95)
Drug use disorder	During pregnancy	2009	0.80(0.60 to 1.00)
Drug use disorder	During pregnancy	2010	0.84(0.64 to 1.05)
Drug use disorder	During pregnancy	2011	0.89(0.68 to 1.10)
Drug use disorder	During pregnancy	2012	0.93(0.72 to 1.14)
Drug use disorder	During pregnancy	2013	0.97(0.75 to 1.19)
Drug use disorder	During pregnancy	2014	1.00(0.78 to 1.22)
Drug use disorder	During pregnancy	2015	1.03(0.80 to 1.25)
Drug use disorder	During pregnancy	2016	1.03(0.81 to 1.26)
Drug use disorder	During pregnancy	2017	1.03(0.80 to 1.26)
Drug use disorder	During pregnancy	2018	1.02(0.79 to 1.25)
Drug use disorder	During pregnancy	2019	0.99(0.76 to 1.23)
Drug use disorder	During pregnancy	2020	0.95(0.72 to 1.18)
Drug use disorder	During pregnancy	2021	0.88(0.65 to 1.11)
Drug use disorder	After pregnancy	2004	0.55(0.36 to 0.74)
Drug use disorder	After pregnancy	2005	0.60(0.42 to 0.78)
Drug use disorder	After pregnancy	2006	0.67(0.49 to 0.84)
Drug use disorder	After pregnancy	2007	0.72(0.54 to 0.90)
Drug use disorder	After pregnancy	2008	0.77(0.59 to 0.95)
Drug use disorder	After pregnancy	2009	0.83(0.64 to 1.01)
Drug use disorder	After pregnancy	2010	0.88(0.69 to 1.06)
Drug use disorder	After pregnancy	2011	0.92(0.73 to 1.11)
Drug use disorder	After pregnancy	2012	0.96(0.77 to 1.16)
Drug use disorder	After pregnancy	2013	1.00(0.80 to 1.19)
Drug use disorder	After pregnancy	2014	1.03(0.83 to 1.23)
Drug use disorder	After pregnancy	2015	1.05(0.85 to 1.25)
Drug use disorder	After pregnancy	2016	1.06(0.86 to 1.27)
Drug use disorder	After pregnancy	2017	1.06(0.85 to 1.26)
Drug use disorder	After pregnancy	2018	1.04(0.83 to 1.24)
Drug use disorder	After pregnancy	2019	1.00(0.80 to 1.21)
Drug use disorder	After pregnancy	2020	0.96(0.75 to 1.16)
Drug use disorder	After pregnancy	2021	0.88(0.68 to 1.08)
Drug use disorder	After pregnancy	2022	0.77(0.58 to 0.96)

© 2026 Xiang N et al. *JAMA Network Open*.

ADHD	Before pregnancy	2003	0.02(-0.03 to 0.07)
ADHD	Before pregnancy	2004	0.11(0.04 to 0.18)
ADHD	Before pregnancy	2005	0.24(0.14 to 0.34)
ADHD	Before pregnancy	2006	0.40(0.27 to 0.52)
ADHD	Before pregnancy	2007	0.56(0.42 to 0.71)
ADHD	Before pregnancy	2008	0.73(0.56 to 0.90)
ADHD	Before pregnancy	2009	0.89(0.70 to 1.08)
ADHD	Before pregnancy	2010	1.05(0.84 to 1.26)
ADHD	Before pregnancy	2011	1.20(0.97 to 1.43)
ADHD	Before pregnancy	2012	1.35(1.10 to 1.60)
ADHD	Before pregnancy	2013	1.48(1.22 to 1.74)
ADHD	Before pregnancy	2014	1.58(1.31 to 1.85)
ADHD	Before pregnancy	2015	1.65(1.37 to 1.93)
ADHD	Before pregnancy	2016	1.71(1.42 to 2.00)
ADHD	Before pregnancy	2017	1.77(1.47 to 2.07)
ADHD	Before pregnancy	2018	1.82(1.51 to 2.14)
ADHD	Before pregnancy	2019	1.86(1.53 to 2.19)
ADHD	Before pregnancy	2020	1.89(1.54 to 2.23)
ADHD	Before pregnancy	2021	1.91(1.54 to 2.28)
ADHD	During pregnancy	2003	-0.02(-0.05 to 0.04)
ADHD	During pregnancy	2004	0.08(0.02 to 0.16)
ADHD	During pregnancy	2005	0.21(0.12 to 0.32)
ADHD	During pregnancy	2006	0.37(0.24 to 0.49)
ADHD	During pregnancy	2007	0.53(0.38 to 0.68)
ADHD	During pregnancy	2008	0.70(0.53 to 0.88)
ADHD	During pregnancy	2009	0.87(0.67 to 1.07)
ADHD	During pregnancy	2010	1.03(0.81 to 1.25)
ADHD	During pregnancy	2011	1.18(0.94 to 1.41)
ADHD	During pregnancy	2012	1.30(1.05 to 1.55)
ADHD	During pregnancy	2013	1.41(1.15 to 1.67)
ADHD	During pregnancy	2014	1.49(1.22 to 1.76)
ADHD	During pregnancy	2015	1.54(1.26 to 1.82)
ADHD	During pregnancy	2016	1.57(1.29 to 1.85)
ADHD	During pregnancy	2017	1.59(1.31 to 1.88)
ADHD	During pregnancy	2018	1.61(1.32 to 1.90)
ADHD	During pregnancy	2019	1.63(1.33 to 1.92)
ADHD	During pregnancy	2020	1.65(1.34 to 1.95)
ADHD	During pregnancy	2021	1.67(1.36 to 1.99)
ADHD	After pregnancy	2004	-0.02(-0.06 to 0.03)
ADHD	After pregnancy	2005	0.12(0.05 to 0.19)
ADHD	After pregnancy	2006	0.26(0.17 to 0.36)
ADHD	After pregnancy	2007	0.43(0.31 to 0.55)
ADHD	After pregnancy	2008	0.60(0.46 to 0.75)
ADHD	After pregnancy	2009	0.79(0.62 to 0.95)
ADHD	After pregnancy	2010	0.97(0.78 to 1.15)

© 2026 Xiang N et al. *JAMA Network Open*.

ADHD	After pregnancy	2011	1.14(0.94 to 1.34)
ADHD	After pregnancy	2012	1.29(1.07 to 1.51)
ADHD	After pregnancy	2013	1.42(1.19 to 1.65)
ADHD	After pregnancy	2014	1.52(1.28 to 1.76)
ADHD	After pregnancy	2015	1.60(1.35 to 1.85)
ADHD	After pregnancy	2016	1.66(1.41 to 1.92)
ADHD	After pregnancy	2017	1.70(1.44 to 1.96)
ADHD	After pregnancy	2018	1.72(1.46 to 1.98)
ADHD	After pregnancy	2019	1.73(1.46 to 1.99)
ADHD	After pregnancy	2020	1.74(1.47 to 2.01)
ADHD	After pregnancy	2021	1.76(1.49 to 2.03)
ADHD	After pregnancy	2022	1.79(1.51 to 2.06)
Bipolar disorder	Before pregnancy	2003	0.08(0.02 to 0.13)
Bipolar disorder	Before pregnancy	2004	0.10(0.03 to 0.16)
Bipolar disorder	Before pregnancy	2005	0.13(0.05 to 0.20)
Bipolar disorder	Before pregnancy	2006	0.16(0.08 to 0.24)
Bipolar disorder	Before pregnancy	2007	0.20(0.11 to 0.29)
Bipolar disorder	Before pregnancy	2008	0.23(0.13 to 0.33)
Bipolar disorder	Before pregnancy	2009	0.27(0.16 to 0.38)
Bipolar disorder	Before pregnancy	2010	0.30(0.18 to 0.41)
Bipolar disorder	Before pregnancy	2011	0.33(0.21 to 0.45)
Bipolar disorder	Before pregnancy	2012	0.35(0.23 to 0.48)
Bipolar disorder	Before pregnancy	2013	0.38(0.24 to 0.51)
Bipolar disorder	Before pregnancy	2014	0.39(0.26 to 0.53)
Bipolar disorder	Before pregnancy	2015	0.40(0.26 to 0.53)
Bipolar disorder	Before pregnancy	2016	0.40(0.26 to 0.53)
Bipolar disorder	Before pregnancy	2017	0.39(0.26 to 0.53)
Bipolar disorder	Before pregnancy	2018	0.38(0.25 to 0.52)
Bipolar disorder	Before pregnancy	2019	0.37(0.23 to 0.51)
Bipolar disorder	Before pregnancy	2020	0.34(0.21 to 0.48)
Bipolar disorder	Before pregnancy	2021	0.30(0.16 to 0.43)
Bipolar disorder	During pregnancy	2003	0.10(-0.01 to 0.21)
Bipolar disorder	During pregnancy	2004	0.12(0.02 to 0.23)
Bipolar disorder	During pregnancy	2005	0.16(0.05 to 0.26)
Bipolar disorder	During pregnancy	2006	0.19(0.08 to 0.30)
Bipolar disorder	During pregnancy	2007	0.22(0.11 to 0.33)
Bipolar disorder	During pregnancy	2008	0.25(0.14 to 0.36)
Bipolar disorder	During pregnancy	2009	0.28(0.16 to 0.40)
Bipolar disorder	During pregnancy	2010	0.31(0.18 to 0.43)
Bipolar disorder	During pregnancy	2011	0.33(0.20 to 0.46)
Bipolar disorder	During pregnancy	2012	0.35(0.22 to 0.48)
Bipolar disorder	During pregnancy	2013	0.35(0.22 to 0.49)
Bipolar disorder	During pregnancy	2014	0.36(0.22 to 0.49)
Bipolar disorder	During pregnancy	2015	0.35(0.22 to 0.49)
Bipolar disorder	During pregnancy	2016	0.35(0.22 to 0.48)

© 2026 Xiang N et al. *JAMA Network Open*.

Bipolar disorder	During pregnancy	2017	0.34(0.21 to 0.47)
Bipolar disorder	During pregnancy	2018	0.33(0.20 to 0.45)
Bipolar disorder	During pregnancy	2019	0.31(0.18 to 0.43)
Bipolar disorder	During pregnancy	2020	0.29(0.17 to 0.41)
Bipolar disorder	During pregnancy	2021	0.28(0.16 to 0.39)
Bipolar disorder	After pregnancy	2004	0.16(0.06 to 0.27)
Bipolar disorder	After pregnancy	2005	0.22(0.11 to 0.33)
Bipolar disorder	After pregnancy	2006	0.27(0.16 to 0.38)
Bipolar disorder	After pregnancy	2007	0.31(0.19 to 0.42)
Bipolar disorder	After pregnancy	2008	0.34(0.22 to 0.45)
Bipolar disorder	After pregnancy	2009	0.37(0.25 to 0.49)
Bipolar disorder	After pregnancy	2010	0.40(0.27 to 0.52)
Bipolar disorder	After pregnancy	2011	0.42(0.29 to 0.55)
Bipolar disorder	After pregnancy	2012	0.44(0.31 to 0.57)
Bipolar disorder	After pregnancy	2013	0.45(0.32 to 0.58)
Bipolar disorder	After pregnancy	2014	0.46(0.33 to 0.59)
Bipolar disorder	After pregnancy	2015	0.46(0.33 to 0.59)
Bipolar disorder	After pregnancy	2016	0.46(0.33 to 0.59)
Bipolar disorder	After pregnancy	2017	0.45(0.32 to 0.59)
Bipolar disorder	After pregnancy	2018	0.44(0.31 to 0.57)
Bipolar disorder	After pregnancy	2019	0.42(0.29 to 0.55)
Bipolar disorder	After pregnancy	2020	0.40(0.27 to 0.52)
Bipolar disorder	After pregnancy	2021	0.37(0.25 to 0.49)
Bipolar disorder	After pregnancy	2022	0.35(0.23 to 0.47)
Psychosis	Before pregnancy	2003	0.15(0.07 to 0.23)
Psychosis	Before pregnancy	2004	0.17(0.08 to 0.25)
Psychosis	Before pregnancy	2005	0.18(0.09 to 0.27)
Psychosis	Before pregnancy	2006	0.19(0.10 to 0.29)
Psychosis	Before pregnancy	2007	0.21(0.11 to 0.30)
Psychosis	Before pregnancy	2008	0.22(0.12 to 0.32)
Psychosis	Before pregnancy	2009	0.23(0.13 to 0.33)
Psychosis	Before pregnancy	2010	0.24(0.14 to 0.35)
Psychosis	Before pregnancy	2011	0.25(0.14 to 0.36)
Psychosis	Before pregnancy	2012	0.26(0.15 to 0.37)
Psychosis	Before pregnancy	2013	0.27(0.16 to 0.38)
Psychosis	Before pregnancy	2014	0.27(0.16 to 0.39)
Psychosis	Before pregnancy	2015	0.28(0.16 to 0.39)
Psychosis	Before pregnancy	2016	0.28(0.16 to 0.39)
Psychosis	Before pregnancy	2017	0.27(0.15 to 0.38)
Psychosis	Before pregnancy	2018	0.26(0.14 to 0.37)
Psychosis	Before pregnancy	2019	0.24(0.13 to 0.35)
Psychosis	Before pregnancy	2020	0.22(0.11 to 0.34)
Psychosis	Before pregnancy	2021	0.20(0.08 to 0.31)
Psychosis	During pregnancy	2003	0.24(0.07 to 0.41)
Psychosis	During pregnancy	2004	0.24(0.10 to 0.39)

© 2026 Xiang N et al. *JAMA Network Open*.

Psychosis	During pregnancy	2005	0.25(0.12 to 0.39)
Psychosis	During pregnancy	2006	0.26(0.13 to 0.39)
Psychosis	During pregnancy	2007	0.26(0.14 to 0.39)
Psychosis	During pregnancy	2008	0.27(0.15 to 0.39)
Psychosis	During pregnancy	2009	0.28(0.16 to 0.39)
Psychosis	During pregnancy	2010	0.28(0.16 to 0.40)
Psychosis	During pregnancy	2011	0.29(0.17 to 0.40)
Psychosis	During pregnancy	2012	0.28(0.16 to 0.40)
Psychosis	During pregnancy	2013	0.28(0.16 to 0.40)
Psychosis	During pregnancy	2014	0.28(0.17 to 0.40)
Psychosis	During pregnancy	2015	0.29(0.17 to 0.41)
Psychosis	During pregnancy	2016	0.30(0.18 to 0.42)
Psychosis	During pregnancy	2017	0.30(0.18 to 0.42)
Psychosis	During pregnancy	2018	0.30(0.18 to 0.43)
Psychosis	During pregnancy	2019	0.31(0.18 to 0.43)
Psychosis	During pregnancy	2020	0.32(0.19 to 0.45)
Psychosis	During pregnancy	2021	0.31(0.18 to 0.45)
Psychosis	After pregnancy	2004	0.27(0.14 to 0.40)
Psychosis	After pregnancy	2005	0.27(0.14 to 0.39)
Psychosis	After pregnancy	2006	0.27(0.15 to 0.39)
Psychosis	After pregnancy	2007	0.27(0.16 to 0.39)
Psychosis	After pregnancy	2008	0.28(0.17 to 0.38)
Psychosis	After pregnancy	2009	0.28(0.17 to 0.39)
Psychosis	After pregnancy	2010	0.29(0.18 to 0.39)
Psychosis	After pregnancy	2011	0.29(0.19 to 0.40)
Psychosis	After pregnancy	2012	0.30(0.19 to 0.40)
Psychosis	After pregnancy	2013	0.30(0.19 to 0.41)
Psychosis	After pregnancy	2014	0.30(0.20 to 0.41)
Psychosis	After pregnancy	2015	0.30(0.19 to 0.41)
Psychosis	After pregnancy	2016	0.30(0.19 to 0.40)
Psychosis	After pregnancy	2017	0.29(0.18 to 0.40)
Psychosis	After pregnancy	2018	0.28(0.18 to 0.38)
Psychosis	After pregnancy	2019	0.27(0.16 to 0.37)
Psychosis	After pregnancy	2020	0.25(0.15 to 0.35)
Psychosis	After pregnancy	2021	0.23(0.13 to 0.32)
Psychosis	After pregnancy	2022	0.20(0.11 to 0.29)

*ADHD, attention deficit hyperactivity disorder.

*Incidence rate was standardized by age at childbirth.

© 2026 Xiang N et al. *JAMA Network Open*.

eTable 3. Standardized Incidence Rates of Any Paternal Psychiatric Disorder and 9 Type-Specific Disorders Before, During, and After Pregnancy, by Weeks

Psychiatric disorders	Phase	Week	Standardized incidence rate per 1000 person-years (95%CI)
**Any psychiatric disorder**	Before pregnancy	1	7.00(5.97 to 8.04)
**Any psychiatric disorder**	Before pregnancy	2	6.97(5.94 to 8.00)
**Any psychiatric disorder**	Before pregnancy	3	6.94(5.91 to 7.97)
**Any psychiatric disorder**	Before pregnancy	4	6.91(5.89 to 7.94)
**Any psychiatric disorder**	Before pregnancy	5	6.89(5.87 to 7.91)
**Any psychiatric disorder**	Before pregnancy	6	6.86(5.84 to 7.88)
**Any psychiatric disorder**	Before pregnancy	7	6.83(5.82 to 7.84)
**Any psychiatric disorder**	Before pregnancy	8	6.80(5.79 to 7.81)
**Any psychiatric disorder**	Before pregnancy	9	6.77(5.76 to 7.77)
**Any psychiatric disorder**	Before pregnancy	10	6.73(5.73 to 7.73)
**Any psychiatric disorder**	Before pregnancy	11	6.70(5.70 to 7.70)
**Any psychiatric disorder**	Before pregnancy	12	6.66(5.67 to 7.66)
**Any psychiatric disorder**	Before pregnancy	13	6.63(5.64 to 7.62)
**Any psychiatric disorder**	Before pregnancy	14	6.60(5.61 to 7.58)
**Any psychiatric disorder**	Before pregnancy	15	6.57(5.59 to 7.55)
**Any psychiatric disorder**	Before pregnancy	16	6.54(5.56 to 7.51)
**Any psychiatric disorder**	Before pregnancy	17	6.51(5.53 to 7.48)
**Any psychiatric disorder**	Before pregnancy	18	6.48(5.51 to 7.45)
**Any psychiatric disorder**	Before pregnancy	19	6.45(5.49 to 7.42)
**Any psychiatric disorder**	Before pregnancy	20	6.43(5.46 to 7.39)
**Any psychiatric disorder**	Before pregnancy	21	6.40(5.45 to 7.36)
**Any psychiatric disorder**	Before pregnancy	22	6.38(5.43 to 7.34)
**Any psychiatric disorder**	Before pregnancy	23	6.36(5.41 to 7.32)
**Any psychiatric disorder**	Before pregnancy	24	6.35(5.40 to 7.30)
**Any psychiatric disorder**	Before pregnancy	25	6.34(5.39 to 7.28)
**Any psychiatric disorder**	Before pregnancy	26	6.33(5.38 to 7.27)
**Any psychiatric disorder**	Before pregnancy	27	6.32(5.38 to 7.26)
**Any psychiatric disorder**	Before pregnancy	28	6.31(5.37 to 7.25)
**Any psychiatric disorder**	Before pregnancy	29	6.31(5.37 to 7.25)
**Any psychiatric disorder**	Before pregnancy	30	6.30(5.37 to 7.24)
**Any psychiatric disorder**	Before pregnancy	31	6.30(5.37 to 7.23)
**Any psychiatric disorder**	Before pregnancy	32	6.29(5.36 to 7.23)
**Any psychiatric disorder**	Before pregnancy	33	6.29(5.36 to 7.22)
**Any psychiatric disorder**	Before pregnancy	34	6.28(5.36 to 7.21)
**Any psychiatric disorder**	Before pregnancy	35	6.28(5.35 to 7.21)
**Any psychiatric disorder**	Before pregnancy	36	6.28(5.35 to 7.20)
**Any psychiatric disorder**	Before pregnancy	37	6.27(5.35 to 7.19)
**Any psychiatric disorder**	Before pregnancy	38	6.27(5.35 to 7.19)
**Any psychiatric disorder**	Before pregnancy	39	6.26(5.34 to 7.18)

© 2026 Xiang N et al. *JAMA Network Open*.

**Any psychiatric disorder**	Before pregnancy	40	6.25(5.34 to 7.17)
**Any psychiatric disorder**	Before pregnancy	41	6.24(5.33 to 7.16)
**Any psychiatric disorder**	Before pregnancy	42	6.23(5.32 to 7.14)
**Any psychiatric disorder**	Before pregnancy	43	6.22(5.31 to 7.12)
**Any psychiatric disorder**	Before pregnancy	44	6.20(5.29 to 7.10)
**Any psychiatric disorder**	Before pregnancy	45	6.17(5.27 to 7.07)
**Any psychiatric disorder**	Before pregnancy	46	6.14(5.24 to 7.03)
**Any psychiatric disorder**	Before pregnancy	47	6.10(5.20 to 6.99)
**Any psychiatric disorder**	Before pregnancy	48	6.04(5.16 to 6.93)
**Any psychiatric disorder**	Before pregnancy	49	5.98(5.10 to 6.86)
**Any psychiatric disorder**	Before pregnancy	50	5.91(5.04 to 6.79)
**Any psychiatric disorder**	Before pregnancy	51	5.83(4.96 to 6.70)
**Any psychiatric disorder**	During pregnancy	1	5.50(4.69 to 6.31)
**Any psychiatric disorder**	During pregnancy	2	5.49(4.69 to 6.30)
**Any psychiatric disorder**	During pregnancy	3	5.49(4.69 to 6.30)
**Any psychiatric disorder**	During pregnancy	4	5.50(4.69 to 6.31)
**Any psychiatric disorder**	During pregnancy	5	5.51(4.70 to 6.31)
**Any psychiatric disorder**	During pregnancy	6	5.52(4.71 to 6.33)
**Any psychiatric disorder**	During pregnancy	7	5.53(4.72 to 6.34)
**Any psychiatric disorder**	During pregnancy	8	5.55(4.74 to 6.36)
**Any psychiatric disorder**	During pregnancy	9	5.56(4.75 to 6.37)
**Any psychiatric disorder**	During pregnancy	10	5.58(4.76 to 6.39)
**Any psychiatric disorder**	During pregnancy	11	5.59(4.77 to 6.40)
**Any psychiatric disorder**	During pregnancy	12	5.60(4.78 to 6.41)
**Any psychiatric disorder**	During pregnancy	13	5.60(4.79 to 6.41)
**Any psychiatric disorder**	During pregnancy	14	5.60(4.79 to 6.41)
**Any psychiatric disorder**	During pregnancy	15	5.60(4.78 to 6.41)
**Any psychiatric disorder**	During pregnancy	16	5.59(4.78 to 6.40)
**Any psychiatric disorder**	During pregnancy	17	5.58(4.76 to 6.39)
**Any psychiatric disorder**	During pregnancy	18	5.56(4.75 to 6.37)
**Any psychiatric disorder**	During pregnancy	19	5.54(4.73 to 6.35)
**Any psychiatric disorder**	During pregnancy	20	5.52(4.71 to 6.33)
**Any psychiatric disorder**	During pregnancy	21	5.50(4.69 to 6.30)
**Any psychiatric disorder**	During pregnancy	22	5.47(4.67 to 6.28)
**Any psychiatric disorder**	During pregnancy	23	5.44(4.64 to 6.25)
**Any psychiatric disorder**	During pregnancy	24	5.42(4.61 to 6.22)
**Any psychiatric disorder**	During pregnancy	25	5.38(4.58 to 6.18)
**Any psychiatric disorder**	During pregnancy	26	5.34(4.54 to 6.14)
**Any psychiatric disorder**	During pregnancy	27	5.30(4.49 to 6.10)
**Any psychiatric disorder**	During pregnancy	28	5.24(4.43 to 6.05)
**Any psychiatric disorder**	During pregnancy	29	5.18(4.37 to 6.00)
**Any psychiatric disorder**	During pregnancy	30	5.12(4.29 to 5.95)
**Any psychiatric disorder**	During pregnancy	31	5.05(4.21 to 5.89)
**Any psychiatric disorder**	During pregnancy	32	4.98(4.12 to 5.84)
**Any psychiatric disorder**	During pregnancy	33	4.91(4.02 to 5.80)

© 2026 Xiang N et al. *JAMA Network Open*.

**Any psychiatric disorder**	During pregnancy	34	4.84(3.93 to 5.75)
**Any psychiatric disorder**	During pregnancy	35	4.76(3.82 to 5.71)
**Any psychiatric disorder**	During pregnancy	36	4.68(3.70 to 5.67)
**Any psychiatric disorder**	During pregnancy	37	4.59(3.56 to 5.62)
**Any psychiatric disorder**	During pregnancy	38	4.49(3.40 to 5.57)
**Any psychiatric disorder**	During pregnancy	39	4.36(3.22 to 5.50)
**Any psychiatric disorder**	During pregnancy	40	4.20(2.98 to 5.42)
**Any psychiatric disorder**	During pregnancy	41	4.01(2.70 to 5.33)
**Any psychiatric disorder**	After pregnancy	1	5.19(4.41 to 5.97)
**Any psychiatric disorder**	After pregnancy	2	5.22(4.44 to 6.01)
**Any psychiatric disorder**	After pregnancy	3	5.26(4.47 to 6.04)
**Any psychiatric disorder**	After pregnancy	4	5.29(4.50 to 6.08)
**Any psychiatric disorder**	After pregnancy	5	5.32(4.53 to 6.11)
**Any psychiatric disorder**	After pregnancy	6	5.35(4.56 to 6.15)
**Any psychiatric disorder**	After pregnancy	7	5.38(4.59 to 6.18)
**Any psychiatric disorder**	After pregnancy	8	5.41(4.62 to 6.21)
**Any psychiatric disorder**	After pregnancy	9	5.44(4.64 to 6.24)
**Any psychiatric disorder**	After pregnancy	10	5.47(4.67 to 6.27)
**Any psychiatric disorder**	After pregnancy	11	5.50(4.69 to 6.30)
**Any psychiatric disorder**	After pregnancy	12	5.53(4.72 to 6.33)
**Any psychiatric disorder**	After pregnancy	13	5.55(4.74 to 6.36)
**Any psychiatric disorder**	After pregnancy	14	5.58(4.77 to 6.39)
**Any psychiatric disorder**	After pregnancy	15	5.61(4.79 to 6.42)
**Any psychiatric disorder**	After pregnancy	16	5.64(4.82 to 6.45)
**Any psychiatric disorder**	After pregnancy	17	5.66(4.84 to 6.48)
**Any psychiatric disorder**	After pregnancy	18	5.69(4.86 to 6.51)
**Any psychiatric disorder**	After pregnancy	19	5.71(4.89 to 6.54)
**Any psychiatric disorder**	After pregnancy	20	5.74(4.91 to 6.57)
**Any psychiatric disorder**	After pregnancy	21	5.76(4.93 to 6.59)
**Any psychiatric disorder**	After pregnancy	22	5.79(4.96 to 6.62)
**Any psychiatric disorder**	After pregnancy	23	5.81(4.98 to 6.65)
**Any psychiatric disorder**	After pregnancy	24	5.84(5.00 to 6.68)
**Any psychiatric disorder**	After pregnancy	25	5.86(5.02 to 6.70)
**Any psychiatric disorder**	After pregnancy	26	5.89(5.04 to 6.73)
**Any psychiatric disorder**	After pregnancy	27	5.91(5.07 to 6.76)
**Any psychiatric disorder**	After pregnancy	28	5.93(5.09 to 6.78)
**Any psychiatric disorder**	After pregnancy	29	5.96(5.10 to 6.81)
**Any psychiatric disorder**	After pregnancy	30	5.98(5.12 to 6.83)
**Any psychiatric disorder**	After pregnancy	31	6.00(5.14 to 6.85)
**Any psychiatric disorder**	After pregnancy	32	6.02(5.16 to 6.87)
**Any psychiatric disorder**	After pregnancy	33	6.03(5.17 to 6.89)
**Any psychiatric disorder**	After pregnancy	34	6.05(5.19 to 6.91)
**Any psychiatric disorder**	After pregnancy	35	6.07(5.21 to 6.93)
**Any psychiatric disorder**	After pregnancy	36	6.09(5.22 to 6.95)
**Any psychiatric disorder**	After pregnancy	37	6.10(5.23 to 6.97)

© 2026 Xiang N et al. *JAMA Network Open*.

**Any psychiatric disorder**	After pregnancy	38	6.12(5.25 to 6.99)
**Any psychiatric disorder**	After pregnancy	39	6.13(5.26 to 7.00)
**Any psychiatric disorder**	After pregnancy	40	6.15(5.27 to 7.02)
**Any psychiatric disorder**	After pregnancy	41	6.16(5.28 to 7.03)
**Any psychiatric disorder**	After pregnancy	42	6.16(5.29 to 7.04)
**Any psychiatric disorder**	After pregnancy	43	6.17(5.29 to 7.05)
**Any psychiatric disorder**	After pregnancy	44	6.18(5.30 to 7.05)
**Any psychiatric disorder**	After pregnancy	45	6.18(5.30 to 7.06)
**Any psychiatric disorder**	After pregnancy	46	6.18(5.30 to 7.05)
**Any psychiatric disorder**	After pregnancy	47	6.17(5.29 to 7.05)
**Any psychiatric disorder**	After pregnancy	48	6.16(5.28 to 7.04)
**Any psychiatric disorder**	After pregnancy	49	6.15(5.27 to 7.03)
**Any psychiatric disorder**	After pregnancy	50	6.14(5.27 to 7.02)
**Any psychiatric disorder**	After pregnancy	51	6.13(5.26 to 7.01)
**Depression**	Before pregnancy	1	2.39(1.81 to 2.97)
**Depression**	Before pregnancy	2	2.38(1.80 to 2.96)
**Depression**	Before pregnancy	3	2.38(1.80 to 2.96)
**Depression**	Before pregnancy	4	2.38(1.80 to 2.95)
**Depression**	Before pregnancy	5	2.37(1.79 to 2.94)
**Depression**	Before pregnancy	6	2.36(1.79 to 2.93)
**Depression**	Before pregnancy	7	2.35(1.78 to 2.92)
**Depression**	Before pregnancy	8	2.33(1.77 to 2.90)
**Depression**	Before pregnancy	9	2.32(1.75 to 2.88)
**Depression**	Before pregnancy	10	2.30(1.74 to 2.87)
**Depression**	Before pregnancy	11	2.29(1.73 to 2.85)
**Depression**	Before pregnancy	12	2.28(1.72 to 2.83)
**Depression**	Before pregnancy	13	2.26(1.71 to 2.82)
**Depression**	Before pregnancy	14	2.25(1.70 to 2.80)
**Depression**	Before pregnancy	15	2.24(1.69 to 2.79)
**Depression**	Before pregnancy	16	2.23(1.68 to 2.78)
**Depression**	Before pregnancy	17	2.22(1.67 to 2.76)
**Depression**	Before pregnancy	18	2.21(1.67 to 2.75)
**Depression**	Before pregnancy	19	2.20(1.66 to 2.74)
**Depression**	Before pregnancy	20	2.20(1.66 to 2.74)
**Depression**	Before pregnancy	21	2.19(1.65 to 2.73)
**Depression**	Before pregnancy	22	2.19(1.65 to 2.72)
**Depression**	Before pregnancy	23	2.18(1.65 to 2.72)
**Depression**	Before pregnancy	24	2.18(1.65 to 2.71)
**Depression**	Before pregnancy	25	2.18(1.65 to 2.71)
**Depression**	Before pregnancy	26	2.18(1.65 to 2.71)
**Depression**	Before pregnancy	27	2.17(1.64 to 2.70)
**Depression**	Before pregnancy	28	2.17(1.64 to 2.70)
**Depression**	Before pregnancy	29	2.17(1.64 to 2.70)
**Depression**	Before pregnancy	30	2.17(1.64 to 2.70)
**Depression**	Before pregnancy	31	2.17(1.64 to 2.69)

**Depression**	Before pregnancy	32	2.16(1.64 to 2.68)
**Depression**	Before pregnancy	33	2.15(1.63 to 2.67)
**Depression**	Before pregnancy	34	2.14(1.62 to 2.66)
**Depression**	Before pregnancy	35	2.13(1.62 to 2.65)
**Depression**	Before pregnancy	36	2.12(1.61 to 2.64)
**Depression**	Before pregnancy	37	2.11(1.60 to 2.62)
**Depression**	Before pregnancy	38	2.09(1.58 to 2.60)
**Depression**	Before pregnancy	39	2.07(1.57 to 2.58)
**Depression**	Before pregnancy	40	2.05(1.55 to 2.56)
**Depression**	Before pregnancy	41	2.03(1.53 to 2.53)
**Depression**	Before pregnancy	42	2.00(1.51 to 2.50)
**Depression**	Before pregnancy	43	1.97(1.48 to 2.46)
**Depression**	Before pregnancy	44	1.93(1.45 to 2.42)
**Depression**	Before pregnancy	45	1.89(1.41 to 2.37)
**Depression**	Before pregnancy	46	1.84(1.37 to 2.31)
**Depression**	Before pregnancy	47	1.79(1.33 to 2.25)
**Depression**	Before pregnancy	48	1.73(1.27 to 2.19)
**Depression**	Before pregnancy	49	1.67(1.22 to 2.11)
**Depression**	Before pregnancy	50	1.60(1.16 to 2.04)
**Depression**	Before pregnancy	51	1.53(1.11 to 1.96)
**Depression**	During pregnancy	1	1.88(1.43 to 2.33)
**Depression**	During pregnancy	2	1.87(1.42 to 2.33)
**Depression**	During pregnancy	3	1.87(1.42 to 2.33)
**Depression**	During pregnancy	4	1.88(1.42 to 2.33)
**Depression**	During pregnancy	5	1.88(1.43 to 2.33)
**Depression**	During pregnancy	6	1.89(1.43 to 2.34)
**Depression**	During pregnancy	7	1.89(1.44 to 2.35)
**Depression**	During pregnancy	8	1.90(1.45 to 2.35)
**Depression**	During pregnancy	9	1.90(1.45 to 2.36)
**Depression**	During pregnancy	10	1.91(1.45 to 2.36)
**Depression**	During pregnancy	11	1.91(1.45 to 2.36)
**Depression**	During pregnancy	12	1.91(1.45 to 2.36)
**Depression**	During pregnancy	13	1.91(1.45 to 2.36)
**Depression**	During pregnancy	14	1.91(1.45 to 2.36)
**Depression**	During pregnancy	15	1.90(1.45 to 2.35)
**Depression**	During pregnancy	16	1.89(1.44 to 2.35)
**Depression**	During pregnancy	17	1.89(1.43 to 2.34)
**Depression**	During pregnancy	18	1.88(1.43 to 2.33)
**Depression**	During pregnancy	19	1.86(1.42 to 2.31)
**Depression**	During pregnancy	20	1.85(1.40 to 2.30)
**Depression**	During pregnancy	21	1.84(1.39 to 2.28)
**Depression**	During pregnancy	22	1.83(1.38 to 2.27)
**Depression**	During pregnancy	23	1.81(1.37 to 2.25)
**Depression**	During pregnancy	24	1.80(1.36 to 2.24)
**Depression**	During pregnancy	25	1.78(1.34 to 2.22)

© 2026 Xiang N et al. *JAMA Network Open*.

**Depression**	During pregnancy	26	1.77(1.33 to 2.20)
**Depression**	During pregnancy	27	1.74(1.31 to 2.18)
**Depression**	During pregnancy	28	1.72(1.28 to 2.16)
**Depression**	During pregnancy	29	1.69(1.25 to 2.13)
**Depression**	During pregnancy	30	1.66(1.22 to 2.10)
**Depression**	During pregnancy	31	1.62(1.18 to 2.07)
**Depression**	During pregnancy	32	1.58(1.14 to 2.03)
**Depression**	During pregnancy	33	1.54(1.10 to 2.00)
**Depression**	During pregnancy	34	1.50(1.05 to 1.96)
**Depression**	During pregnancy	35	1.45(1.00 to 1.92)
**Depression**	During pregnancy	36	1.40(0.94 to 1.88)
**Depression**	During pregnancy	37	1.34(0.87 to 1.83)
**Depression**	During pregnancy	38	1.27(0.80 to 1.78)
**Depression**	During pregnancy	39	1.18(0.70 to 1.71)
**Depression**	During pregnancy	40	1.07(0.58 to 1.62)
**Depression**	During pregnancy	41	0.93(0.43 to 1.50)
**Depression**	After pregnancy	1	1.55(1.14 to 1.96)
**Depression**	After pregnancy	2	1.59(1.18 to 2.01)
**Depression**	After pregnancy	3	1.63(1.22 to 2.05)
**Depression**	After pregnancy	4	1.68(1.25 to 2.10)
**Depression**	After pregnancy	5	1.72(1.29 to 2.14)
**Depression**	After pregnancy	6	1.75(1.32 to 2.19)
**Depression**	After pregnancy	7	1.79(1.35 to 2.23)
**Depression**	After pregnancy	8	1.82(1.38 to 2.27)
**Depression**	After pregnancy	9	1.85(1.41 to 2.30)
**Depression**	After pregnancy	10	1.88(1.43 to 2.33)
**Depression**	After pregnancy	11	1.91(1.45 to 2.36)
**Depression**	After pregnancy	12	1.93(1.47 to 2.39)
**Depression**	After pregnancy	13	1.95(1.49 to 2.41)
**Depression**	After pregnancy	14	1.97(1.51 to 2.44)
**Depression**	After pregnancy	15	2.00(1.53 to 2.46)
**Depression**	After pregnancy	16	2.02(1.55 to 2.49)
**Depression**	After pregnancy	17	2.04(1.57 to 2.51)
**Depression**	After pregnancy	18	2.06(1.59 to 2.54)
**Depression**	After pregnancy	19	2.08(1.61 to 2.56)
**Depression**	After pregnancy	20	2.11(1.63 to 2.58)
**Depression**	After pregnancy	21	2.13(1.65 to 2.61)
**Depression**	After pregnancy	22	2.15(1.66 to 2.63)
**Depression**	After pregnancy	23	2.17(1.68 to 2.65)
**Depression**	After pregnancy	24	2.18(1.69 to 2.67)
**Depression**	After pregnancy	25	2.20(1.71 to 2.69)
**Depression**	After pregnancy	26	2.22(1.72 to 2.71)
**Depression**	After pregnancy	27	2.23(1.74 to 2.73)
**Depression**	After pregnancy	28	2.24(1.75 to 2.74)
**Depression**	After pregnancy	29	2.26(1.76 to 2.76)

© 2026 Xiang N et al. *JAMA Network Open*.

**Depression**	After pregnancy	30	2.27(1.77 to 2.77)
**Depression**	After pregnancy	31	2.28(1.78 to 2.78)
**Depression**	After pregnancy	32	2.29(1.79 to 2.80)
**Depression**	After pregnancy	33	2.30(1.79 to 2.81)
**Depression**	After pregnancy	34	2.31(1.80 to 2.82)
**Depression**	After pregnancy	35	2.32(1.81 to 2.83)
**Depression**	After pregnancy	36	2.33(1.82 to 2.84)
**Depression**	After pregnancy	37	2.34(1.82 to 2.85)
**Depression**	After pregnancy	38	2.34(1.83 to 2.86)
**Depression**	After pregnancy	39	2.35(1.83 to 2.86)
**Depression**	After pregnancy	40	2.35(1.83 to 2.86)
**Depression**	After pregnancy	41	2.35(1.83 to 2.86)
**Depression**	After pregnancy	42	2.35(1.83 to 2.86)
**Depression**	After pregnancy	43	2.34(1.82 to 2.86)
**Depression**	After pregnancy	44	2.33(1.82 to 2.85)
**Depression**	After pregnancy	45	2.32(1.81 to 2.84)
**Depression**	After pregnancy	46	2.31(1.80 to 2.82)
**Depression**	After pregnancy	47	2.30(1.78 to 2.81)
**Depression**	After pregnancy	48	2.28(1.77 to 2.79)
**Depression**	After pregnancy	49	2.26(1.75 to 2.77)
**Depression**	After pregnancy	50	2.24(1.74 to 2.75)
**Depression**	After pregnancy	51	2.22(1.72 to 2.72)
**Anxiety**	Before pregnancy	1	2.68(2.06 to 3.30)
**Anxiety**	Before pregnancy	2	2.67(2.06 to 3.29)
**Anxiety**	Before pregnancy	3	2.66(2.05 to 3.28)
**Anxiety**	Before pregnancy	4	2.65(2.04 to 3.26)
**Anxiety**	Before pregnancy	5	2.64(2.03 to 3.25)
**Anxiety**	Before pregnancy	6	2.62(2.02 to 3.23)
**Anxiety**	Before pregnancy	7	2.61(2.00 to 3.21)
**Anxiety**	Before pregnancy	8	2.59(1.99 to 3.19)
**Anxiety**	Before pregnancy	9	2.57(1.97 to 3.17)
**Anxiety**	Before pregnancy	10	2.55(1.96 to 3.14)
**Anxiety**	Before pregnancy	11	2.53(1.94 to 3.12)
**Anxiety**	Before pregnancy	12	2.51(1.92 to 3.10)
**Anxiety**	Before pregnancy	13	2.49(1.91 to 3.07)
**Anxiety**	Before pregnancy	14	2.47(1.89 to 3.05)
**Anxiety**	Before pregnancy	15	2.45(1.88 to 3.03)
**Anxiety**	Before pregnancy	16	2.44(1.86 to 3.01)
**Anxiety**	Before pregnancy	17	2.42(1.85 to 2.99)
**Anxiety**	Before pregnancy	18	2.41(1.84 to 2.97)
**Anxiety**	Before pregnancy	19	2.39(1.83 to 2.96)
**Anxiety**	Before pregnancy	20	2.38(1.82 to 2.94)
**Anxiety**	Before pregnancy	21	2.37(1.81 to 2.92)
**Anxiety**	Before pregnancy	22	2.35(1.80 to 2.91)
**Anxiety**	Before pregnancy	23	2.35(1.79 to 2.90)

© 2026 Xiang N et al. *JAMA Network Open*.

**Anxiety**	Before pregnancy	24	2.34(1.79 to 2.89)
**Anxiety**	Before pregnancy	25	2.34(1.78 to 2.89)
**Anxiety**	Before pregnancy	26	2.33(1.78 to 2.88)
**Anxiety**	Before pregnancy	27	2.33(1.78 to 2.88)
**Anxiety**	Before pregnancy	28	2.34(1.79 to 2.88)
**Anxiety**	Before pregnancy	29	2.34(1.79 to 2.89)
**Anxiety**	Before pregnancy	30	2.34(1.79 to 2.89)
**Anxiety**	Before pregnancy	31	2.35(1.80 to 2.89)
**Anxiety**	Before pregnancy	32	2.35(1.80 to 2.90)
**Anxiety**	Before pregnancy	33	2.36(1.81 to 2.90)
**Anxiety**	Before pregnancy	34	2.36(1.82 to 2.91)
**Anxiety**	Before pregnancy	35	2.37(1.82 to 2.91)
**Anxiety**	Before pregnancy	36	2.37(1.83 to 2.92)
**Anxiety**	Before pregnancy	37	2.38(1.83 to 2.92)
**Anxiety**	Before pregnancy	38	2.38(1.84 to 2.93)
**Anxiety**	Before pregnancy	39	2.39(1.84 to 2.93)
**Anxiety**	Before pregnancy	40	2.39(1.85 to 2.93)
**Anxiety**	Before pregnancy	41	2.40(1.85 to 2.94)
**Anxiety**	Before pregnancy	42	2.40(1.86 to 2.94)
**Anxiety**	Before pregnancy	43	2.40(1.86 to 2.94)
**Anxiety**	Before pregnancy	44	2.40(1.86 to 2.94)
**Anxiety**	Before pregnancy	45	2.40(1.86 to 2.94)
**Anxiety**	Before pregnancy	46	2.39(1.86 to 2.93)
**Anxiety**	Before pregnancy	47	2.38(1.85 to 2.92)
**Anxiety**	Before pregnancy	48	2.37(1.83 to 2.90)
**Anxiety**	Before pregnancy	49	2.35(1.82 to 2.87)
**Anxiety**	Before pregnancy	50	2.32(1.79 to 2.84)
**Anxiety**	Before pregnancy	51	2.29(1.77 to 2.81)
**Anxiety**	During pregnancy	1	2.46(1.94 to 2.97)
**Anxiety**	During pregnancy	2	2.42(1.91 to 2.93)
**Anxiety**	During pregnancy	3	2.39(1.88 to 2.89)
**Anxiety**	During pregnancy	4	2.35(1.85 to 2.86)
**Anxiety**	During pregnancy	5	2.32(1.82 to 2.82)
**Anxiety**	During pregnancy	6	2.29(1.79 to 2.79)
**Anxiety**	During pregnancy	7	2.26(1.77 to 2.76)
**Anxiety**	During pregnancy	8	2.24(1.75 to 2.74)
**Anxiety**	During pregnancy	9	2.23(1.74 to 2.72)
**Anxiety**	During pregnancy	10	2.22(1.73 to 2.71)
**Anxiety**	During pregnancy	11	2.22(1.72 to 2.71)
**Anxiety**	During pregnancy	12	2.21(1.72 to 2.70)
**Anxiety**	During pregnancy	13	2.21(1.72 to 2.70)
**Anxiety**	During pregnancy	14	2.22(1.73 to 2.71)
**Anxiety**	During pregnancy	15	2.22(1.73 to 2.71)
**Anxiety**	During pregnancy	16	2.22(1.73 to 2.72)
**Anxiety**	During pregnancy	17	2.23(1.74 to 2.72)

© 2026 Xiang N et al. *JAMA Network Open*.

**Anxiety**	During pregnancy	18	2.24(1.74 to 2.73)
**Anxiety**	During pregnancy	19	2.25(1.75 to 2.74)
**Anxiety**	During pregnancy	20	2.26(1.76 to 2.75)
**Anxiety**	During pregnancy	21	2.27(1.77 to 2.76)
**Anxiety**	During pregnancy	22	2.28(1.78 to 2.77)
**Anxiety**	During pregnancy	23	2.29(1.79 to 2.79)
**Anxiety**	During pregnancy	24	2.30(1.80 to 2.80)
**Anxiety**	During pregnancy	25	2.30(1.80 to 2.80)
**Anxiety**	During pregnancy	26	2.31(1.80 to 2.81)
**Anxiety**	During pregnancy	27	2.31(1.80 to 2.81)
**Anxiety**	During pregnancy	28	2.30(1.79 to 2.81)
**Anxiety**	During pregnancy	29	2.29(1.77 to 2.81)
**Anxiety**	During pregnancy	30	2.27(1.74 to 2.80)
**Anxiety**	During pregnancy	31	2.25(1.71 to 2.79)
**Anxiety**	During pregnancy	32	2.23(1.67 to 2.78)
**Anxiety**	During pregnancy	33	2.20(1.63 to 2.76)
**Anxiety**	During pregnancy	34	2.17(1.58 to 2.75)
**Anxiety**	During pregnancy	35	2.13(1.53 to 2.74)
**Anxiety**	During pregnancy	36	2.09(1.47 to 2.72)
**Anxiety**	During pregnancy	37	2.05(1.40 to 2.71)
**Anxiety**	During pregnancy	38	2.00(1.31 to 2.69)
**Anxiety**	During pregnancy	39	1.94(1.22 to 2.67)
**Anxiety**	During pregnancy	40	1.87(1.10 to 2.64)
**Anxiety**	During pregnancy	41	1.79(0.96 to 2.62)
**Anxiety**	After pregnancy	1	2.22(1.73 to 2.71)
**Anxiety**	After pregnancy	2	2.21(1.73 to 2.70)
**Anxiety**	After pregnancy	3	2.21(1.72 to 2.70)
**Anxiety**	After pregnancy	4	2.21(1.73 to 2.70)
**Anxiety**	After pregnancy	5	2.22(1.73 to 2.71)
**Anxiety**	After pregnancy	6	2.23(1.74 to 2.72)
**Anxiety**	After pregnancy	7	2.23(1.74 to 2.73)
**Anxiety**	After pregnancy	8	2.25(1.75 to 2.74)
**Anxiety**	After pregnancy	9	2.26(1.77 to 2.75)
**Anxiety**	After pregnancy	10	2.27(1.78 to 2.77)
**Anxiety**	After pregnancy	11	2.29(1.79 to 2.79)
**Anxiety**	After pregnancy	12	2.31(1.81 to 2.81)
**Anxiety**	After pregnancy	13	2.33(1.82 to 2.83)
**Anxiety**	After pregnancy	14	2.34(1.84 to 2.85)
**Anxiety**	After pregnancy	15	2.36(1.85 to 2.87)
**Anxiety**	After pregnancy	16	2.38(1.87 to 2.88)
**Anxiety**	After pregnancy	17	2.39(1.88 to 2.90)
**Anxiety**	After pregnancy	18	2.40(1.89 to 2.92)
**Anxiety**	After pregnancy	19	2.42(1.90 to 2.93)
**Anxiety**	After pregnancy	20	2.43(1.92 to 2.95)
**Anxiety**	After pregnancy	21	2.44(1.93 to 2.96)

© 2026 Xiang N et al. *JAMA Network Open*.

**Anxiety**	After pregnancy	22	2.46(1.94 to 2.98)
**Anxiety**	After pregnancy	23	2.47(1.95 to 2.99)
**Anxiety**	After pregnancy	24	2.48(1.96 to 3.00)
**Anxiety**	After pregnancy	25	2.49(1.97 to 3.02)
**Anxiety**	After pregnancy	26	2.50(1.98 to 3.03)
**Anxiety**	After pregnancy	27	2.51(1.98 to 3.04)
**Anxiety**	After pregnancy	28	2.52(1.99 to 3.04)
**Anxiety**	After pregnancy	29	2.52(1.99 to 3.05)
**Anxiety**	After pregnancy	30	2.52(1.99 to 3.05)
**Anxiety**	After pregnancy	31	2.53(2.00 to 3.06)
**Anxiety**	After pregnancy	32	2.53(2.00 to 3.06)
**Anxiety**	After pregnancy	33	2.53(1.99 to 3.06)
**Anxiety**	After pregnancy	34	2.52(1.99 to 3.06)
**Anxiety**	After pregnancy	35	2.52(1.99 to 3.05)
**Anxiety**	After pregnancy	36	2.51(1.98 to 3.05)
**Anxiety**	After pregnancy	37	2.51(1.98 to 3.04)
**Anxiety**	After pregnancy	38	2.50(1.97 to 3.04)
**Anxiety**	After pregnancy	39	2.50(1.97 to 3.03)
**Anxiety**	After pregnancy	40	2.49(1.96 to 3.02)
**Anxiety**	After pregnancy	41	2.49(1.96 to 3.02)
**Anxiety**	After pregnancy	42	2.48(1.95 to 3.01)
**Anxiety**	After pregnancy	43	2.48(1.95 to 3.01)
**Anxiety**	After pregnancy	44	2.47(1.94 to 3.00)
**Anxiety**	After pregnancy	45	2.47(1.93 to 3.00)
**Anxiety**	After pregnancy	46	2.46(1.93 to 2.99)
**Anxiety**	After pregnancy	47	2.45(1.92 to 2.97)
**Anxiety**	After pregnancy	48	2.43(1.90 to 2.96)
**Anxiety**	After pregnancy	49	2.41(1.89 to 2.94)
**Anxiety**	After pregnancy	50	2.40(1.88 to 2.92)
**Anxiety**	After pregnancy	51	2.38(1.86 to 2.90)
**Stress-related disorder**	Before pregnancy	1	2.07(1.53 to 2.61)
**Stress-related disorder**	Before pregnancy	2	2.03(1.50 to 2.57)
**Stress-related disorder**	Before pregnancy	3	2.00(1.47 to 2.53)
**Stress-related disorder**	Before pregnancy	4	1.97(1.45 to 2.50)
**Stress-related disorder**	Before pregnancy	5	1.95(1.43 to 2.48)
**Stress-related disorder**	Before pregnancy	6	1.93(1.41 to 2.45)
**Stress-related disorder**	Before pregnancy	7	1.92(1.40 to 2.44)
**Stress-related disorder**	Before pregnancy	8	1.91(1.39 to 2.42)
**Stress-related disorder**	Before pregnancy	9	1.90(1.39 to 2.41)
**Stress-related disorder**	Before pregnancy	10	1.89(1.38 to 2.40)
**Stress-related disorder**	Before pregnancy	11	1.89(1.38 to 2.39)
**Stress-related disorder**	Before pregnancy	12	1.88(1.38 to 2.39)
**Stress-related disorder**	Before pregnancy	13	1.88(1.37 to 2.38)
**Stress-related disorder**	Before pregnancy	14	1.87(1.37 to 2.38)
**Stress-related disorder**	Before pregnancy	15	1.87(1.37 to 2.37)

© 2026 Xiang N et al. *JAMA Network Open*.

**Stress-related disorder**	Before pregnancy	16	1.86(1.36 to 2.36)
**Stress-related disorder**	Before pregnancy	17	1.86(1.36 to 2.36)
**Stress-related disorder**	Before pregnancy	18	1.85(1.36 to 2.35)
**Stress-related disorder**	Before pregnancy	19	1.85(1.35 to 2.34)
**Stress-related disorder**	Before pregnancy	20	1.84(1.35 to 2.33)
**Stress-related disorder**	Before pregnancy	21	1.84(1.34 to 2.33)
**Stress-related disorder**	Before pregnancy	22	1.83(1.34 to 2.32)
**Stress-related disorder**	Before pregnancy	23	1.83(1.34 to 2.31)
**Stress-related disorder**	Before pregnancy	24	1.82(1.33 to 2.31)
**Stress-related disorder**	Before pregnancy	25	1.82(1.33 to 2.30)
**Stress-related disorder**	Before pregnancy	26	1.81(1.33 to 2.29)
**Stress-related disorder**	Before pregnancy	27	1.81(1.32 to 2.29)
**Stress-related disorder**	Before pregnancy	28	1.80(1.32 to 2.28)
**Stress-related disorder**	Before pregnancy	29	1.80(1.32 to 2.27)
**Stress-related disorder**	Before pregnancy	30	1.79(1.31 to 2.27)
**Stress-related disorder**	Before pregnancy	31	1.78(1.31 to 2.26)
**Stress-related disorder**	Before pregnancy	32	1.77(1.30 to 2.24)
**Stress-related disorder**	Before pregnancy	33	1.76(1.29 to 2.23)
**Stress-related disorder**	Before pregnancy	34	1.75(1.28 to 2.21)
**Stress-related disorder**	Before pregnancy	35	1.73(1.27 to 2.20)
**Stress-related disorder**	Before pregnancy	36	1.72(1.25 to 2.18)
**Stress-related disorder**	Before pregnancy	37	1.70(1.24 to 2.16)
**Stress-related disorder**	Before pregnancy	38	1.68(1.23 to 2.14)
**Stress-related disorder**	Before pregnancy	39	1.67(1.22 to 2.12)
**Stress-related disorder**	Before pregnancy	40	1.65(1.20 to 2.10)
**Stress-related disorder**	Before pregnancy	41	1.64(1.19 to 2.08)
**Stress-related disorder**	Before pregnancy	42	1.62(1.18 to 2.06)
**Stress-related disorder**	Before pregnancy	43	1.61(1.17 to 2.05)
**Stress-related disorder**	Before pregnancy	44	1.59(1.15 to 2.03)
**Stress-related disorder**	Before pregnancy	45	1.57(1.14 to 2.00)
**Stress-related disorder**	Before pregnancy	46	1.55(1.12 to 1.98)
**Stress-related disorder**	Before pregnancy	47	1.53(1.10 to 1.95)
**Stress-related disorder**	Before pregnancy	48	1.50(1.08 to 1.92)
**Stress-related disorder**	Before pregnancy	49	1.47(1.05 to 1.88)
**Stress-related disorder**	Before pregnancy	50	1.43(1.02 to 1.84)
**Stress-related disorder**	Before pregnancy	51	1.38(0.98 to 1.78)
**Stress-related disorder**	During pregnancy	1	1.63(1.21 to 2.05)
**Stress-related disorder**	During pregnancy	2	1.60(1.19 to 2.02)
**Stress-related disorder**	During pregnancy	3	1.59(1.17 to 2.00)
**Stress-related disorder**	During pregnancy	4	1.58(1.17 to 1.99)
**Stress-related disorder**	During pregnancy	5	1.57(1.16 to 1.99)
**Stress-related disorder**	During pregnancy	6	1.57(1.16 to 1.98)
**Stress-related disorder**	During pregnancy	7	1.57(1.16 to 1.98)
**Stress-related disorder**	During pregnancy	8	1.57(1.16 to 1.98)
**Stress-related disorder**	During pregnancy	9	1.57(1.16 to 1.98)

© 2026 Xiang N et al. *JAMA Network Open*.

**Stress-related disorder**	During pregnancy	10	1.57(1.16 to 1.99)
**Stress-related disorder**	During pregnancy	11	1.58(1.16 to 1.99)
**Stress-related disorder**	During pregnancy	12	1.58(1.17 to 1.99)
**Stress-related disorder**	During pregnancy	13	1.58(1.17 to 2.00)
**Stress-related disorder**	During pregnancy	14	1.58(1.17 to 2.00)
**Stress-related disorder**	During pregnancy	15	1.59(1.17 to 2.00)
**Stress-related disorder**	During pregnancy	16	1.59(1.17 to 2.00)
**Stress-related disorder**	During pregnancy	17	1.59(1.17 to 2.00)
**Stress-related disorder**	During pregnancy	18	1.58(1.17 to 2.00)
**Stress-related disorder**	During pregnancy	19	1.58(1.16 to 1.99)
**Stress-related disorder**	During pregnancy	20	1.57(1.16 to 1.98)
**Stress-related disorder**	During pregnancy	21	1.56(1.15 to 1.96)
**Stress-related disorder**	During pregnancy	22	1.54(1.13 to 1.95)
**Stress-related disorder**	During pregnancy	23	1.53(1.12 to 1.93)
**Stress-related disorder**	During pregnancy	24	1.51(1.11 to 1.91)
**Stress-related disorder**	During pregnancy	25	1.50(1.09 to 1.90)
**Stress-related disorder**	During pregnancy	26	1.48(1.08 to 1.88)
**Stress-related disorder**	During pregnancy	27	1.46(1.06 to 1.86)
**Stress-related disorder**	During pregnancy	28	1.44(1.04 to 1.84)
**Stress-related disorder**	During pregnancy	29	1.42(1.01 to 1.82)
**Stress-related disorder**	During pregnancy	30	1.39(0.98 to 1.80)
**Stress-related disorder**	During pregnancy	31	1.36(0.95 to 1.77)
**Stress-related disorder**	During pregnancy	32	1.33(0.91 to 1.75)
**Stress-related disorder**	During pregnancy	33	1.30(0.88 to 1.73)
**Stress-related disorder**	During pregnancy	34	1.27(0.84 to 1.71)
**Stress-related disorder**	During pregnancy	35	1.24(0.79 to 1.69)
**Stress-related disorder**	During pregnancy	36	1.21(0.75 to 1.68)
**Stress-related disorder**	During pregnancy	37	1.18(0.70 to 1.66)
**Stress-related disorder**	During pregnancy	38	1.15(0.64 to 1.65)
**Stress-related disorder**	During pregnancy	39	1.11(0.58 to 1.64)
**Stress-related disorder**	During pregnancy	40	1.07(0.50 to 1.63)
**Stress-related disorder**	During pregnancy	41	1.01(0.41 to 1.61)
**Stress-related disorder**	After pregnancy	1	1.97(1.51 to 2.43)
**Stress-related disorder**	After pregnancy	2	1.96(1.51 to 2.42)
**Stress-related disorder**	After pregnancy	3	1.96(1.50 to 2.41)
**Stress-related disorder**	After pregnancy	4	1.95(1.49 to 2.40)
**Stress-related disorder**	After pregnancy	5	1.94(1.48 to 2.39)
**Stress-related disorder**	After pregnancy	6	1.93(1.48 to 2.39)
**Stress-related disorder**	After pregnancy	7	1.92(1.47 to 2.38)
**Stress-related disorder**	After pregnancy	8	1.91(1.46 to 2.37)
**Stress-related disorder**	After pregnancy	9	1.91(1.46 to 2.36)
**Stress-related disorder**	After pregnancy	10	1.90(1.45 to 2.36)
**Stress-related disorder**	After pregnancy	11	1.90(1.45 to 2.35)
**Stress-related disorder**	After pregnancy	12	1.90(1.45 to 2.35)
**Stress-related disorder**	After pregnancy	13	1.90(1.45 to 2.35)

© 2026 Xiang N et al. *JAMA Network Open*.

**Stress-related disorder**	After pregnancy	14	1.90(1.45 to 2.36)
**Stress-related disorder**	After pregnancy	15	1.91(1.45 to 2.36)
**Stress-related disorder**	After pregnancy	16	1.91(1.46 to 2.37)
**Stress-related disorder**	After pregnancy	17	1.92(1.46 to 2.37)
**Stress-related disorder**	After pregnancy	18	1.92(1.47 to 2.38)
**Stress-related disorder**	After pregnancy	19	1.93(1.47 to 2.39)
**Stress-related disorder**	After pregnancy	20	1.94(1.48 to 2.40)
**Stress-related disorder**	After pregnancy	21	1.95(1.49 to 2.41)
**Stress-related disorder**	After pregnancy	22	1.95(1.49 to 2.41)
**Stress-related disorder**	After pregnancy	23	1.96(1.50 to 2.42)
**Stress-related disorder**	After pregnancy	24	1.97(1.51 to 2.43)
**Stress-related disorder**	After pregnancy	25	1.98(1.51 to 2.44)
**Stress-related disorder**	After pregnancy	26	1.99(1.52 to 2.45)
**Stress-related disorder**	After pregnancy	27	2.00(1.53 to 2.46)
**Stress-related disorder**	After pregnancy	28	2.00(1.53 to 2.47)
**Stress-related disorder**	After pregnancy	29	2.01(1.54 to 2.48)
**Stress-related disorder**	After pregnancy	30	2.01(1.54 to 2.49)
**Stress-related disorder**	After pregnancy	31	2.02(1.55 to 2.49)
**Stress-related disorder**	After pregnancy	32	2.02(1.55 to 2.50)
**Stress-related disorder**	After pregnancy	33	2.03(1.55 to 2.50)
**Stress-related disorder**	After pregnancy	34	2.03(1.56 to 2.51)
**Stress-related disorder**	After pregnancy	35	2.04(1.56 to 2.51)
**Stress-related disorder**	After pregnancy	36	2.04(1.56 to 2.52)
**Stress-related disorder**	After pregnancy	37	2.04(1.56 to 2.52)
**Stress-related disorder**	After pregnancy	38	2.04(1.56 to 2.52)
**Stress-related disorder**	After pregnancy	39	2.04(1.56 to 2.52)
**Stress-related disorder**	After pregnancy	40	2.04(1.56 to 2.52)
**Stress-related disorder**	After pregnancy	41	2.04(1.56 to 2.52)
**Stress-related disorder**	After pregnancy	42	2.03(1.56 to 2.51)
**Stress-related disorder**	After pregnancy	43	2.03(1.55 to 2.51)
**Stress-related disorder**	After pregnancy	44	2.03(1.55 to 2.51)
**Stress-related disorder**	After pregnancy	45	2.02(1.54 to 2.50)
**Stress-related disorder**	After pregnancy	46	2.02(1.54 to 2.49)
**Stress-related disorder**	After pregnancy	47	2.01(1.53 to 2.49)
**Stress-related disorder**	After pregnancy	48	2.01(1.53 to 2.49)
**Stress-related disorder**	After pregnancy	49	2.01(1.53 to 2.49)
**Stress-related disorder**	After pregnancy	50	2.02(1.54 to 2.49)
**Stress-related disorder**	After pregnancy	51	2.03(1.55 to 2.50)
**Alcohol use disorder**	Before pregnancy	1	1.76(1.26 to 2.26)
**Alcohol use disorder**	Before pregnancy	2	1.73(1.24 to 2.23)
**Alcohol use disorder**	Before pregnancy	3	1.71(1.22 to 2.20)
**Alcohol use disorder**	Before pregnancy	4	1.69(1.20 to 2.18)
**Alcohol use disorder**	Before pregnancy	5	1.67(1.19 to 2.15)
**Alcohol use disorder**	Before pregnancy	6	1.66(1.18 to 2.14)
**Alcohol use disorder**	Before pregnancy	7	1.64(1.17 to 2.12)

© 2026 Xiang N et al. *JAMA Network Open*.

**Alcohol use disorder**	Before pregnancy	8	1.63(1.16 to 2.11)
**Alcohol use disorder**	Before pregnancy	9	1.62(1.15 to 2.10)
**Alcohol use disorder**	Before pregnancy	10	1.61(1.14 to 2.08)
**Alcohol use disorder**	Before pregnancy	11	1.60(1.14 to 2.07)
**Alcohol use disorder**	Before pregnancy	12	1.59(1.13 to 2.05)
**Alcohol use disorder**	Before pregnancy	13	1.58(1.12 to 2.04)
**Alcohol use disorder**	Before pregnancy	14	1.56(1.10 to 2.02)
**Alcohol use disorder**	Before pregnancy	15	1.55(1.09 to 2.00)
**Alcohol use disorder**	Before pregnancy	16	1.53(1.08 to 1.98)
**Alcohol use disorder**	Before pregnancy	17	1.52(1.07 to 1.96)
**Alcohol use disorder**	Before pregnancy	18	1.50(1.05 to 1.95)
**Alcohol use disorder**	Before pregnancy	19	1.49(1.04 to 1.93)
**Alcohol use disorder**	Before pregnancy	20	1.47(1.03 to 1.91)
**Alcohol use disorder**	Before pregnancy	21	1.46(1.03 to 1.90)
**Alcohol use disorder**	Before pregnancy	22	1.45(1.02 to 1.89)
**Alcohol use disorder**	Before pregnancy	23	1.44(1.01 to 1.88)
**Alcohol use disorder**	Before pregnancy	24	1.44(1.01 to 1.87)
**Alcohol use disorder**	Before pregnancy	25	1.43(1.00 to 1.86)
**Alcohol use disorder**	Before pregnancy	26	1.42(1.00 to 1.85)
**Alcohol use disorder**	Before pregnancy	27	1.42(0.99 to 1.85)
**Alcohol use disorder**	Before pregnancy	28	1.41(0.99 to 1.84)
**Alcohol use disorder**	Before pregnancy	29	1.41(0.98 to 1.83)
**Alcohol use disorder**	Before pregnancy	30	1.40(0.98 to 1.82)
**Alcohol use disorder**	Before pregnancy	31	1.39(0.97 to 1.81)
**Alcohol use disorder**	Before pregnancy	32	1.38(0.96 to 1.80)
**Alcohol use disorder**	Before pregnancy	33	1.37(0.96 to 1.79)
**Alcohol use disorder**	Before pregnancy	34	1.37(0.95 to 1.78)
**Alcohol use disorder**	Before pregnancy	35	1.36(0.95 to 1.77)
**Alcohol use disorder**	Before pregnancy	36	1.36(0.94 to 1.77)
**Alcohol use disorder**	Before pregnancy	37	1.35(0.94 to 1.76)
**Alcohol use disorder**	Before pregnancy	38	1.35(0.94 to 1.76)
**Alcohol use disorder**	Before pregnancy	39	1.35(0.94 to 1.76)
**Alcohol use disorder**	Before pregnancy	40	1.35(0.94 to 1.76)
**Alcohol use disorder**	Before pregnancy	41	1.35(0.95 to 1.76)
**Alcohol use disorder**	Before pregnancy	42	1.35(0.95 to 1.76)
**Alcohol use disorder**	Before pregnancy	43	1.35(0.95 to 1.76)
**Alcohol use disorder**	Before pregnancy	44	1.35(0.95 to 1.76)
**Alcohol use disorder**	Before pregnancy	45	1.35(0.95 to 1.75)
**Alcohol use disorder**	Before pregnancy	46	1.34(0.94 to 1.74)
**Alcohol use disorder**	Before pregnancy	47	1.33(0.93 to 1.73)
**Alcohol use disorder**	Before pregnancy	48	1.32(0.93 to 1.72)
**Alcohol use disorder**	Before pregnancy	49	1.31(0.92 to 1.71)
**Alcohol use disorder**	Before pregnancy	50	1.30(0.91 to 1.69)
**Alcohol use disorder**	Before pregnancy	51	1.29(0.90 to 1.67)
**Alcohol use disorder**	During pregnancy	1	1.07(0.73 to 1.40)

© 2026 Xiang N et al. *JAMA Network Open*.

**Alcohol use disorder**	During pregnancy	2	1.07(0.73 to 1.40)
**Alcohol use disorder**	During pregnancy	3	1.07(0.73 to 1.41)
**Alcohol use disorder**	During pregnancy	4	1.07(0.73 to 1.41)
**Alcohol use disorder**	During pregnancy	5	1.07(0.73 to 1.40)
**Alcohol use disorder**	During pregnancy	6	1.06(0.73 to 1.40)
**Alcohol use disorder**	During pregnancy	7	1.06(0.72 to 1.40)
**Alcohol use disorder**	During pregnancy	8	1.06(0.72 to 1.40)
**Alcohol use disorder**	During pregnancy	9	1.06(0.72 to 1.40)
**Alcohol use disorder**	During pregnancy	10	1.06(0.72 to 1.39)
**Alcohol use disorder**	During pregnancy	11	1.06(0.72 to 1.39)
**Alcohol use disorder**	During pregnancy	12	1.05(0.72 to 1.39)
**Alcohol use disorder**	During pregnancy	13	1.05(0.71 to 1.39)
**Alcohol use disorder**	During pregnancy	14	1.05(0.71 to 1.38)
**Alcohol use disorder**	During pregnancy	15	1.04(0.71 to 1.38)
**Alcohol use disorder**	During pregnancy	16	1.04(0.70 to 1.37)
**Alcohol use disorder**	During pregnancy	17	1.03(0.70 to 1.36)
**Alcohol use disorder**	During pregnancy	18	1.02(0.69 to 1.35)
**Alcohol use disorder**	During pregnancy	19	1.01(0.68 to 1.34)
**Alcohol use disorder**	During pregnancy	20	1.01(0.68 to 1.33)
**Alcohol use disorder**	During pregnancy	21	1.00(0.67 to 1.32)
**Alcohol use disorder**	During pregnancy	22	0.99(0.66 to 1.31)
**Alcohol use disorder**	During pregnancy	23	0.98(0.65 to 1.30)
**Alcohol use disorder**	During pregnancy	24	0.97(0.65 to 1.29)
**Alcohol use disorder**	During pregnancy	25	0.96(0.64 to 1.28)
**Alcohol use disorder**	During pregnancy	26	0.95(0.63 to 1.27)
**Alcohol use disorder**	During pregnancy	27	0.93(0.61 to 1.25)
**Alcohol use disorder**	During pregnancy	28	0.91(0.60 to 1.23)
**Alcohol use disorder**	During pregnancy	29	0.89(0.58 to 1.21)
**Alcohol use disorder**	During pregnancy	30	0.87(0.56 to 1.19)
**Alcohol use disorder**	During pregnancy	31	0.85(0.53 to 1.17)
**Alcohol use disorder**	During pregnancy	32	0.83(0.51 to 1.15)
**Alcohol use disorder**	During pregnancy	33	0.80(0.48 to 1.13)
**Alcohol use disorder**	During pregnancy	34	0.77(0.45 to 1.11)
**Alcohol use disorder**	During pregnancy	35	0.75(0.42 to 1.09)
**Alcohol use disorder**	During pregnancy	36	0.71(0.38 to 1.07)
**Alcohol use disorder**	During pregnancy	37	0.68(0.34 to 1.04)
**Alcohol use disorder**	During pregnancy	38	0.64(0.29 to 1.02)
**Alcohol use disorder**	During pregnancy	39	0.59(0.23 to 0.99)
**Alcohol use disorder**	During pregnancy	40	0.54(0.16 to 0.95)
**Alcohol use disorder**	During pregnancy	41	0.47(0.08 to 0.91)
**Alcohol use disorder**	After pregnancy	1	0.77(0.48 to 1.05)
**Alcohol use disorder**	After pregnancy	2	0.79(0.50 to 1.08)
**Alcohol use disorder**	After pregnancy	3	0.82(0.53 to 1.11)
**Alcohol use disorder**	After pregnancy	4	0.84(0.55 to 1.14)
**Alcohol use disorder**	After pregnancy	5	0.86(0.56 to 1.17)

© 2026 Xiang N et al. *JAMA Network Open*.

**Alcohol use disorder**	After pregnancy	6	0.88(0.58 to 1.19)
**Alcohol use disorder**	After pregnancy	7	0.90(0.59 to 1.21)
**Alcohol use disorder**	After pregnancy	8	0.92(0.60 to 1.23)
**Alcohol use disorder**	After pregnancy	9	0.93(0.61 to 1.24)
**Alcohol use disorder**	After pregnancy	10	0.94(0.62 to 1.25)
**Alcohol use disorder**	After pregnancy	11	0.95(0.63 to 1.26)
**Alcohol use disorder**	After pregnancy	12	0.96(0.64 to 1.27)
**Alcohol use disorder**	After pregnancy	13	0.96(0.64 to 1.28)
**Alcohol use disorder**	After pregnancy	14	0.97(0.65 to 1.29)
**Alcohol use disorder**	After pregnancy	15	0.98(0.66 to 1.30)
**Alcohol use disorder**	After pregnancy	16	0.99(0.66 to 1.31)
**Alcohol use disorder**	After pregnancy	17	1.00(0.67 to 1.32)
**Alcohol use disorder**	After pregnancy	18	1.00(0.67 to 1.33)
**Alcohol use disorder**	After pregnancy	19	1.01(0.68 to 1.34)
**Alcohol use disorder**	After pregnancy	20	1.02(0.68 to 1.35)
**Alcohol use disorder**	After pregnancy	21	1.02(0.69 to 1.35)
**Alcohol use disorder**	After pregnancy	22	1.03(0.69 to 1.36)
**Alcohol use disorder**	After pregnancy	23	1.03(0.70 to 1.37)
**Alcohol use disorder**	After pregnancy	24	1.04(0.70 to 1.38)
**Alcohol use disorder**	After pregnancy	25	1.05(0.71 to 1.38)
**Alcohol use disorder**	After pregnancy	26	1.05(0.71 to 1.39)
**Alcohol use disorder**	After pregnancy	27	1.06(0.72 to 1.40)
**Alcohol use disorder**	After pregnancy	28	1.06(0.72 to 1.40)
**Alcohol use disorder**	After pregnancy	29	1.07(0.73 to 1.41)
**Alcohol use disorder**	After pregnancy	30	1.07(0.73 to 1.42)
**Alcohol use disorder**	After pregnancy	31	1.08(0.74 to 1.43)
**Alcohol use disorder**	After pregnancy	32	1.09(0.74 to 1.44)
**Alcohol use disorder**	After pregnancy	33	1.10(0.75 to 1.45)
**Alcohol use disorder**	After pregnancy	34	1.11(0.76 to 1.46)
**Alcohol use disorder**	After pregnancy	35	1.12(0.76 to 1.47)
**Alcohol use disorder**	After pregnancy	36	1.12(0.77 to 1.48)
**Alcohol use disorder**	After pregnancy	37	1.13(0.78 to 1.49)
**Alcohol use disorder**	After pregnancy	38	1.14(0.78 to 1.50)
**Alcohol use disorder**	After pregnancy	39	1.15(0.79 to 1.51)
**Alcohol use disorder**	After pregnancy	40	1.16(0.80 to 1.52)
**Alcohol use disorder**	After pregnancy	41	1.16(0.80 to 1.52)
**Alcohol use disorder**	After pregnancy	42	1.17(0.81 to 1.53)
**Alcohol use disorder**	After pregnancy	43	1.17(0.81 to 1.54)
**Alcohol use disorder**	After pregnancy	44	1.18(0.82 to 1.54)
**Alcohol use disorder**	After pregnancy	45	1.19(0.82 to 1.55)
**Alcohol use disorder**	After pregnancy	46	1.19(0.83 to 1.56)
**Alcohol use disorder**	After pregnancy	47	1.20(0.83 to 1.57)
**Alcohol use disorder**	After pregnancy	48	1.21(0.84 to 1.57)
**Alcohol use disorder**	After pregnancy	49	1.21(0.84 to 1.58)
**Alcohol use disorder**	After pregnancy	50	1.22(0.85 to 1.59)

© 2026 Xiang N et al. *JAMA Network Open*.

**Alcohol use disorder**	After pregnancy	51	1.22(0.85 to 1.59)
**Tobacco use disorder**	Before pregnancy	1	0.16(0.02 to 0.31)
**Tobacco use disorder**	Before pregnancy	2	0.17(0.03 to 0.32)
**Tobacco use disorder**	Before pregnancy	3	0.18(0.03 to 0.33)
**Tobacco use disorder**	Before pregnancy	4	0.18(0.04 to 0.34)
**Tobacco use disorder**	Before pregnancy	5	0.19(0.04 to 0.35)
**Tobacco use disorder**	Before pregnancy	6	0.20(0.04 to 0.36)
**Tobacco use disorder**	Before pregnancy	7	0.20(0.05 to 0.37)
**Tobacco use disorder**	Before pregnancy	8	0.21(0.05 to 0.37)
**Tobacco use disorder**	Before pregnancy	9	0.21(0.05 to 0.38)
**Tobacco use disorder**	Before pregnancy	10	0.22(0.05 to 0.39)
**Tobacco use disorder**	Before pregnancy	11	0.22(0.06 to 0.39)
**Tobacco use disorder**	Before pregnancy	12	0.22(0.06 to 0.39)
**Tobacco use disorder**	Before pregnancy	13	0.23(0.06 to 0.40)
**Tobacco use disorder**	Before pregnancy	14	0.23(0.06 to 0.40)
**Tobacco use disorder**	Before pregnancy	15	0.23(0.06 to 0.40)
**Tobacco use disorder**	Before pregnancy	16	0.23(0.06 to 0.40)
**Tobacco use disorder**	Before pregnancy	17	0.23(0.06 to 0.40)
**Tobacco use disorder**	Before pregnancy	18	0.23(0.06 to 0.41)
**Tobacco use disorder**	Before pregnancy	19	0.23(0.06 to 0.41)
**Tobacco use disorder**	Before pregnancy	20	0.23(0.06 to 0.41)
**Tobacco use disorder**	Before pregnancy	21	0.23(0.06 to 0.41)
**Tobacco use disorder**	Before pregnancy	22	0.23(0.06 to 0.41)
**Tobacco use disorder**	Before pregnancy	23	0.23(0.06 to 0.40)
**Tobacco use disorder**	Before pregnancy	24	0.23(0.06 to 0.40)
**Tobacco use disorder**	Before pregnancy	25	0.23(0.06 to 0.40)
**Tobacco use disorder**	Before pregnancy	26	0.23(0.06 to 0.40)
**Tobacco use disorder**	Before pregnancy	27	0.23(0.06 to 0.40)
**Tobacco use disorder**	Before pregnancy	28	0.23(0.07 to 0.40)
**Tobacco use disorder**	Before pregnancy	29	0.23(0.07 to 0.40)
**Tobacco use disorder**	Before pregnancy	30	0.23(0.07 to 0.40)
**Tobacco use disorder**	Before pregnancy	31	0.23(0.07 to 0.40)
**Tobacco use disorder**	Before pregnancy	32	0.23(0.07 to 0.40)
**Tobacco use disorder**	Before pregnancy	33	0.23(0.07 to 0.40)
**Tobacco use disorder**	Before pregnancy	34	0.23(0.07 to 0.40)
**Tobacco use disorder**	Before pregnancy	35	0.23(0.07 to 0.40)
**Tobacco use disorder**	Before pregnancy	36	0.23(0.07 to 0.40)
**Tobacco use disorder**	Before pregnancy	37	0.23(0.07 to 0.40)
**Tobacco use disorder**	Before pregnancy	38	0.23(0.07 to 0.40)
**Tobacco use disorder**	Before pregnancy	39	0.23(0.07 to 0.40)
**Tobacco use disorder**	Before pregnancy	40	0.23(0.07 to 0.40)
**Tobacco use disorder**	Before pregnancy	41	0.23(0.07 to 0.40)
**Tobacco use disorder**	Before pregnancy	42	0.23(0.07 to 0.40)
**Tobacco use disorder**	Before pregnancy	43	0.23(0.07 to 0.39)
**Tobacco use disorder**	Before pregnancy	44	0.23(0.07 to 0.39)

© 2026 Xiang N et al. *JAMA Network Open*.

**Tobacco use disorder**	Before pregnancy	45	0.23(0.07 to 0.39)
**Tobacco use disorder**	Before pregnancy	46	0.22(0.07 to 0.38)
**Tobacco use disorder**	Before pregnancy	47	0.22(0.06 to 0.37)
**Tobacco use disorder**	Before pregnancy	48	0.21(0.06 to 0.36)
**Tobacco use disorder**	Before pregnancy	49	0.20(0.05 to 0.35)
**Tobacco use disorder**	Before pregnancy	50	0.19(0.05 to 0.34)
**Tobacco use disorder**	Before pregnancy	51	0.18(0.04 to 0.32)
**Tobacco use disorder**	During pregnancy	1	0.21(0.07 to 0.35)
**Tobacco use disorder**	During pregnancy	2	0.21(0.07 to 0.35)
**Tobacco use disorder**	During pregnancy	3	0.22(0.07 to 0.36)
**Tobacco use disorder**	During pregnancy	4	0.22(0.08 to 0.37)
**Tobacco use disorder**	During pregnancy	5	0.23(0.08 to 0.38)
**Tobacco use disorder**	During pregnancy	6	0.23(0.08 to 0.38)
**Tobacco use disorder**	During pregnancy	7	0.23(0.08 to 0.39)
**Tobacco use disorder**	During pregnancy	8	0.24(0.08 to 0.39)
**Tobacco use disorder**	During pregnancy	9	0.24(0.08 to 0.40)
**Tobacco use disorder**	During pregnancy	10	0.24(0.09 to 0.40)
**Tobacco use disorder**	During pregnancy	11	0.24(0.09 to 0.40)
**Tobacco use disorder**	During pregnancy	12	0.24(0.08 to 0.40)
**Tobacco use disorder**	During pregnancy	13	0.24(0.08 to 0.40)
**Tobacco use disorder**	During pregnancy	14	0.24(0.08 to 0.40)
**Tobacco use disorder**	During pregnancy	15	0.24(0.08 to 0.39)
**Tobacco use disorder**	During pregnancy	16	0.24(0.08 to 0.39)
**Tobacco use disorder**	During pregnancy	17	0.23(0.08 to 0.39)
**Tobacco use disorder**	During pregnancy	18	0.23(0.08 to 0.39)
**Tobacco use disorder**	During pregnancy	19	0.23(0.08 to 0.39)
**Tobacco use disorder**	During pregnancy	20	0.23(0.08 to 0.38)
**Tobacco use disorder**	During pregnancy	21	0.23(0.08 to 0.38)
**Tobacco use disorder**	During pregnancy	22	0.23(0.08 to 0.38)
**Tobacco use disorder**	During pregnancy	23	0.23(0.08 to 0.38)
**Tobacco use disorder**	During pregnancy	24	0.23(0.08 to 0.38)
**Tobacco use disorder**	During pregnancy	25	0.23(0.08 to 0.38)
**Tobacco use disorder**	During pregnancy	26	0.23(0.08 to 0.38)
**Tobacco use disorder**	During pregnancy	27	0.23(0.07 to 0.38)
**Tobacco use disorder**	During pregnancy	28	0.23(0.07 to 0.38)
**Tobacco use disorder**	During pregnancy	29	0.23(0.07 to 0.39)
**Tobacco use disorder**	During pregnancy	30	0.23(0.07 to 0.39)
**Tobacco use disorder**	During pregnancy	31	0.23(0.07 to 0.40)
**Tobacco use disorder**	During pregnancy	32	0.23(0.07 to 0.40)
**Tobacco use disorder**	During pregnancy	33	0.24(0.07 to 0.41)
**Tobacco use disorder**	During pregnancy	34	0.24(0.07 to 0.41)
**Tobacco use disorder**	During pregnancy	35	0.24(0.07 to 0.42)
**Tobacco use disorder**	During pregnancy	36	0.24(0.07 to 0.43)
**Tobacco use disorder**	During pregnancy	37	0.23(0.07 to 0.43)
**Tobacco use disorder**	During pregnancy	38	0.23(0.06 to 0.43)

© 2026 Xiang N et al. *JAMA Network Open*.

**Tobacco use disorder**	During pregnancy	39	0.22(0.06 to 0.44)
**Tobacco use disorder**	During pregnancy	40	0.22(0.05 to 0.44)
**Tobacco use disorder**	During pregnancy	41	0.21(0.04 to 0.44)
**Tobacco use disorder**	After pregnancy	1	0.26(0.10 to 0.43)
**Tobacco use disorder**	After pregnancy	2	0.26(0.09 to 0.42)
**Tobacco use disorder**	After pregnancy	3	0.25(0.09 to 0.41)
**Tobacco use disorder**	After pregnancy	4	0.25(0.09 to 0.41)
**Tobacco use disorder**	After pregnancy	5	0.25(0.09 to 0.41)
**Tobacco use disorder**	After pregnancy	6	0.25(0.09 to 0.41)
**Tobacco use disorder**	After pregnancy	7	0.25(0.09 to 0.41)
**Tobacco use disorder**	After pregnancy	8	0.25(0.09 to 0.41)
**Tobacco use disorder**	After pregnancy	9	0.25(0.09 to 0.41)
**Tobacco use disorder**	After pregnancy	10	0.25(0.09 to 0.41)
**Tobacco use disorder**	After pregnancy	11	0.25(0.09 to 0.41)
**Tobacco use disorder**	After pregnancy	12	0.25(0.09 to 0.41)
**Tobacco use disorder**	After pregnancy	13	0.25(0.09 to 0.41)
**Tobacco use disorder**	After pregnancy	14	0.25(0.09 to 0.41)
**Tobacco use disorder**	After pregnancy	15	0.25(0.09 to 0.41)
**Tobacco use disorder**	After pregnancy	16	0.25(0.09 to 0.41)
**Tobacco use disorder**	After pregnancy	17	0.25(0.09 to 0.41)
**Tobacco use disorder**	After pregnancy	18	0.25(0.09 to 0.42)
**Tobacco use disorder**	After pregnancy	19	0.26(0.09 to 0.42)
**Tobacco use disorder**	After pregnancy	20	0.26(0.09 to 0.42)
**Tobacco use disorder**	After pregnancy	21	0.26(0.09 to 0.42)
**Tobacco use disorder**	After pregnancy	22	0.26(0.10 to 0.43)
**Tobacco use disorder**	After pregnancy	23	0.26(0.10 to 0.43)
**Tobacco use disorder**	After pregnancy	24	0.26(0.10 to 0.43)
**Tobacco use disorder**	After pregnancy	25	0.27(0.10 to 0.43)
**Tobacco use disorder**	After pregnancy	26	0.27(0.10 to 0.43)
**Tobacco use disorder**	After pregnancy	27	0.27(0.10 to 0.44)
**Tobacco use disorder**	After pregnancy	28	0.27(0.10 to 0.44)
**Tobacco use disorder**	After pregnancy	29	0.27(0.10 to 0.44)
**Tobacco use disorder**	After pregnancy	30	0.28(0.11 to 0.45)
**Tobacco use disorder**	After pregnancy	31	0.28(0.11 to 0.45)
**Tobacco use disorder**	After pregnancy	32	0.28(0.11 to 0.45)
**Tobacco use disorder**	After pregnancy	33	0.28(0.11 to 0.45)
**Tobacco use disorder**	After pregnancy	34	0.28(0.11 to 0.46)
**Tobacco use disorder**	After pregnancy	35	0.28(0.11 to 0.46)
**Tobacco use disorder**	After pregnancy	36	0.29(0.11 to 0.46)
**Tobacco use disorder**	After pregnancy	37	0.29(0.11 to 0.46)
**Tobacco use disorder**	After pregnancy	38	0.29(0.11 to 0.46)
**Tobacco use disorder**	After pregnancy	39	0.29(0.11 to 0.47)
**Tobacco use disorder**	After pregnancy	40	0.29(0.12 to 0.47)
**Tobacco use disorder**	After pregnancy	41	0.29(0.12 to 0.47)
**Tobacco use disorder**	After pregnancy	42	0.29(0.12 to 0.47)

© 2026 Xiang N et al. *JAMA Network Open*.

**Tobacco use disorder**	After pregnancy	43	0.29(0.12 to 0.47)
**Tobacco use disorder**	After pregnancy	44	0.29(0.12 to 0.47)
**Tobacco use disorder**	After pregnancy	45	0.29(0.12 to 0.47)
**Tobacco use disorder**	After pregnancy	46	0.29(0.12 to 0.47)
**Tobacco use disorder**	After pregnancy	47	0.29(0.12 to 0.47)
**Tobacco use disorder**	After pregnancy	48	0.29(0.12 to 0.47)
**Tobacco use disorder**	After pregnancy	49	0.29(0.11 to 0.47)
**Tobacco use disorder**	After pregnancy	50	0.29(0.11 to 0.46)
**Tobacco use disorder**	After pregnancy	51	0.28(0.11 to 0.45)
**Drug use disorder**	Before pregnancy	1	1.31(0.89 to 1.74)
**Drug use disorder**	Before pregnancy	2	1.30(0.88 to 1.72)
**Drug use disorder**	Before pregnancy	3	1.29(0.87 to 1.71)
**Drug use disorder**	Before pregnancy	4	1.28(0.86 to 1.70)
**Drug use disorder**	Before pregnancy	5	1.27(0.86 to 1.69)
**Drug use disorder**	Before pregnancy	6	1.27(0.85 to 1.68)
**Drug use disorder**	Before pregnancy	7	1.26(0.85 to 1.67)
**Drug use disorder**	Before pregnancy	8	1.26(0.84 to 1.67)
**Drug use disorder**	Before pregnancy	9	1.25(0.84 to 1.66)
**Drug use disorder**	Before pregnancy	10	1.24(0.84 to 1.65)
**Drug use disorder**	Before pregnancy	11	1.24(0.83 to 1.65)
**Drug use disorder**	Before pregnancy	12	1.24(0.83 to 1.64)
**Drug use disorder**	Before pregnancy	13	1.23(0.83 to 1.64)
**Drug use disorder**	Before pregnancy	14	1.23(0.83 to 1.63)
**Drug use disorder**	Before pregnancy	15	1.23(0.82 to 1.63)
**Drug use disorder**	Before pregnancy	16	1.22(0.82 to 1.62)
**Drug use disorder**	Before pregnancy	17	1.22(0.82 to 1.62)
**Drug use disorder**	Before pregnancy	18	1.21(0.82 to 1.61)
**Drug use disorder**	Before pregnancy	19	1.21(0.81 to 1.61)
**Drug use disorder**	Before pregnancy	20	1.20(0.81 to 1.60)
**Drug use disorder**	Before pregnancy	21	1.20(0.80 to 1.59)
**Drug use disorder**	Before pregnancy	22	1.19(0.80 to 1.58)
**Drug use disorder**	Before pregnancy	23	1.18(0.79 to 1.57)
**Drug use disorder**	Before pregnancy	24	1.17(0.78 to 1.56)
**Drug use disorder**	Before pregnancy	25	1.16(0.77 to 1.54)
**Drug use disorder**	Before pregnancy	26	1.15(0.77 to 1.53)
**Drug use disorder**	Before pregnancy	27	1.14(0.76 to 1.52)
**Drug use disorder**	Before pregnancy	28	1.13(0.75 to 1.50)
**Drug use disorder**	Before pregnancy	29	1.12(0.74 to 1.49)
**Drug use disorder**	Before pregnancy	30	1.11(0.74 to 1.48)
**Drug use disorder**	Before pregnancy	31	1.10(0.73 to 1.47)
**Drug use disorder**	Before pregnancy	32	1.09(0.72 to 1.46)
**Drug use disorder**	Before pregnancy	33	1.09(0.72 to 1.45)
**Drug use disorder**	Before pregnancy	34	1.08(0.71 to 1.44)
**Drug use disorder**	Before pregnancy	35	1.07(0.71 to 1.43)
**Drug use disorder**	Before pregnancy	36	1.06(0.70 to 1.42)

© 2026 Xiang N et al. *JAMA Network Open*.

**Drug use disorder**	Before pregnancy	37	1.05(0.69 to 1.41)
**Drug use disorder**	Before pregnancy	38	1.04(0.69 to 1.40)
**Drug use disorder**	Before pregnancy	39	1.03(0.68 to 1.39)
**Drug use disorder**	Before pregnancy	40	1.02(0.67 to 1.38)
**Drug use disorder**	Before pregnancy	41	1.02(0.67 to 1.37)
**Drug use disorder**	Before pregnancy	42	1.01(0.66 to 1.36)
**Drug use disorder**	Before pregnancy	43	1.00(0.66 to 1.35)
**Drug use disorder**	Before pregnancy	44	1.00(0.65 to 1.34)
**Drug use disorder**	Before pregnancy	45	1.00(0.65 to 1.34)
**Drug use disorder**	Before pregnancy	46	0.99(0.65 to 1.33)
**Drug use disorder**	Before pregnancy	47	0.99(0.64 to 1.33)
**Drug use disorder**	Before pregnancy	48	0.98(0.64 to 1.32)
**Drug use disorder**	Before pregnancy	49	0.97(0.63 to 1.31)
**Drug use disorder**	Before pregnancy	50	0.96(0.62 to 1.29)
**Drug use disorder**	Before pregnancy	51	0.94(0.61 to 1.27)
**Drug use disorder**	During pregnancy	1	0.83(0.54 to 1.13)
**Drug use disorder**	During pregnancy	2	0.84(0.54 to 1.14)
**Drug use disorder**	During pregnancy	3	0.84(0.54 to 1.14)
**Drug use disorder**	During pregnancy	4	0.85(0.55 to 1.15)
**Drug use disorder**	During pregnancy	5	0.86(0.56 to 1.16)
**Drug use disorder**	During pregnancy	6	0.87(0.56 to 1.17)
**Drug use disorder**	During pregnancy	7	0.88(0.57 to 1.18)
**Drug use disorder**	During pregnancy	8	0.89(0.58 to 1.20)
**Drug use disorder**	During pregnancy	9	0.90(0.59 to 1.21)
**Drug use disorder**	During pregnancy	10	0.90(0.59 to 1.21)
**Drug use disorder**	During pregnancy	11	0.91(0.60 to 1.22)
**Drug use disorder**	During pregnancy	12	0.92(0.60 to 1.23)
**Drug use disorder**	During pregnancy	13	0.92(0.61 to 1.24)
**Drug use disorder**	During pregnancy	14	0.93(0.61 to 1.24)
**Drug use disorder**	During pregnancy	15	0.93(0.62 to 1.25)
**Drug use disorder**	During pregnancy	16	0.93(0.62 to 1.25)
**Drug use disorder**	During pregnancy	17	0.94(0.62 to 1.25)
**Drug use disorder**	During pregnancy	18	0.94(0.62 to 1.25)
**Drug use disorder**	During pregnancy	19	0.94(0.62 to 1.25)
**Drug use disorder**	During pregnancy	20	0.93(0.62 to 1.25)
**Drug use disorder**	During pregnancy	21	0.93(0.62 to 1.25)
**Drug use disorder**	During pregnancy	22	0.93(0.62 to 1.24)
**Drug use disorder**	During pregnancy	23	0.93(0.61 to 1.24)
**Drug use disorder**	During pregnancy	24	0.92(0.61 to 1.23)
**Drug use disorder**	During pregnancy	25	0.91(0.60 to 1.22)
**Drug use disorder**	During pregnancy	26	0.90(0.59 to 1.21)
**Drug use disorder**	During pregnancy	27	0.89(0.58 to 1.20)
**Drug use disorder**	During pregnancy	28	0.88(0.57 to 1.19)
**Drug use disorder**	During pregnancy	29	0.86(0.55 to 1.17)
**Drug use disorder**	During pregnancy	30	0.84(0.53 to 1.15)

© 2026 Xiang N et al. *JAMA Network Open*.

**Drug use disorder**	During pregnancy	31	0.82(0.51 to 1.13)
**Drug use disorder**	During pregnancy	32	0.80(0.49 to 1.12)
**Drug use disorder**	During pregnancy	33	0.78(0.47 to 1.10)
**Drug use disorder**	During pregnancy	34	0.75(0.45 to 1.07)
**Drug use disorder**	During pregnancy	35	0.72(0.42 to 1.05)
**Drug use disorder**	During pregnancy	36	0.69(0.39 to 1.02)
**Drug use disorder**	During pregnancy	37	0.65(0.35 to 0.99)
**Drug use disorder**	During pregnancy	38	0.61(0.31 to 0.95)
**Drug use disorder**	During pregnancy	39	0.56(0.26 to 0.91)
**Drug use disorder**	During pregnancy	40	0.50(0.20 to 0.87)
**Drug use disorder**	During pregnancy	41	0.44(0.13 to 0.81)
**Drug use disorder**	After pregnancy	1	0.70(0.43 to 0.97)
**Drug use disorder**	After pregnancy	2	0.72(0.45 to 1.00)
**Drug use disorder**	After pregnancy	3	0.75(0.47 to 1.03)
**Drug use disorder**	After pregnancy	4	0.77(0.48 to 1.05)
**Drug use disorder**	After pregnancy	5	0.78(0.50 to 1.07)
**Drug use disorder**	After pregnancy	6	0.80(0.51 to 1.09)
**Drug use disorder**	After pregnancy	7	0.81(0.52 to 1.11)
**Drug use disorder**	After pregnancy	8	0.83(0.53 to 1.12)
**Drug use disorder**	After pregnancy	9	0.84(0.54 to 1.13)
**Drug use disorder**	After pregnancy	10	0.85(0.55 to 1.15)
**Drug use disorder**	After pregnancy	11	0.86(0.56 to 1.16)
**Drug use disorder**	After pregnancy	12	0.86(0.56 to 1.17)
**Drug use disorder**	After pregnancy	13	0.87(0.57 to 1.17)
**Drug use disorder**	After pregnancy	14	0.87(0.57 to 1.18)
**Drug use disorder**	After pregnancy	15	0.88(0.57 to 1.18)
**Drug use disorder**	After pregnancy	16	0.88(0.58 to 1.19)
**Drug use disorder**	After pregnancy	17	0.89(0.58 to 1.19)
**Drug use disorder**	After pregnancy	18	0.89(0.58 to 1.20)
**Drug use disorder**	After pregnancy	19	0.89(0.59 to 1.20)
**Drug use disorder**	After pregnancy	20	0.90(0.59 to 1.20)
**Drug use disorder**	After pregnancy	21	0.90(0.59 to 1.21)
**Drug use disorder**	After pregnancy	22	0.90(0.59 to 1.21)
**Drug use disorder**	After pregnancy	23	0.90(0.59 to 1.21)
**Drug use disorder**	After pregnancy	24	0.91(0.60 to 1.22)
**Drug use disorder**	After pregnancy	25	0.91(0.60 to 1.22)
**Drug use disorder**	After pregnancy	26	0.91(0.60 to 1.23)
**Drug use disorder**	After pregnancy	27	0.92(0.60 to 1.23)
**Drug use disorder**	After pregnancy	28	0.92(0.61 to 1.24)
**Drug use disorder**	After pregnancy	29	0.93(0.61 to 1.24)
**Drug use disorder**	After pregnancy	30	0.93(0.61 to 1.25)
**Drug use disorder**	After pregnancy	31	0.93(0.62 to 1.25)
**Drug use disorder**	After pregnancy	32	0.94(0.62 to 1.26)
**Drug use disorder**	After pregnancy	33	0.94(0.62 to 1.27)
**Drug use disorder**	After pregnancy	34	0.95(0.63 to 1.27)

© 2026 Xiang N et al. *JAMA Network Open*.

**Drug use disorder**	After pregnancy	35	0.96(0.63 to 1.28)
**Drug use disorder**	After pregnancy	36	0.96(0.64 to 1.29)
**Drug use disorder**	After pregnancy	37	0.97(0.64 to 1.30)
**Drug use disorder**	After pregnancy	38	0.98(0.65 to 1.31)
**Drug use disorder**	After pregnancy	39	0.98(0.65 to 1.31)
**Drug use disorder**	After pregnancy	40	0.99(0.66 to 1.32)
**Drug use disorder**	After pregnancy	41	1.00(0.67 to 1.33)
**Drug use disorder**	After pregnancy	42	1.01(0.67 to 1.34)
**Drug use disorder**	After pregnancy	43	1.01(0.68 to 1.35)
**Drug use disorder**	After pregnancy	44	1.02(0.68 to 1.35)
**Drug use disorder**	After pregnancy	45	1.02(0.68 to 1.36)
**Drug use disorder**	After pregnancy	46	1.02(0.68 to 1.36)
**Drug use disorder**	After pregnancy	47	1.03(0.69 to 1.37)
**Drug use disorder**	After pregnancy	48	1.03(0.69 to 1.37)
**Drug use disorder**	After pregnancy	49	1.03(0.69 to 1.37)
**Drug use disorder**	After pregnancy	50	1.04(0.70 to 1.38)
**Drug use disorder**	After pregnancy	51	1.04(0.70 to 1.38)
**ADHD**	Before pregnancy	1	1.21(0.80 to 1.63)
**ADHD**	Before pregnancy	2	1.21(0.80 to 1.63)
**ADHD**	Before pregnancy	3	1.21(0.80 to 1.62)
**ADHD**	Before pregnancy	4	1.21(0.80 to 1.62)
**ADHD**	Before pregnancy	5	1.20(0.80 to 1.61)
**ADHD**	Before pregnancy	6	1.20(0.79 to 1.60)
**ADHD**	Before pregnancy	7	1.19(0.79 to 1.59)
**ADHD**	Before pregnancy	8	1.18(0.78 to 1.58)
**ADHD**	Before pregnancy	9	1.18(0.78 to 1.58)
**ADHD**	Before pregnancy	10	1.17(0.77 to 1.57)
**ADHD**	Before pregnancy	11	1.17(0.77 to 1.57)
**ADHD**	Before pregnancy	12	1.17(0.77 to 1.56)
**ADHD**	Before pregnancy	13	1.17(0.77 to 1.56)
**ADHD**	Before pregnancy	14	1.17(0.77 to 1.56)
**ADHD**	Before pregnancy	15	1.17(0.77 to 1.56)
**ADHD**	Before pregnancy	16	1.17(0.77 to 1.56)
**ADHD**	Before pregnancy	17	1.16(0.77 to 1.56)
**ADHD**	Before pregnancy	18	1.16(0.77 to 1.55)
**ADHD**	Before pregnancy	19	1.16(0.77 to 1.55)
**ADHD**	Before pregnancy	20	1.15(0.76 to 1.54)
**ADHD**	Before pregnancy	21	1.14(0.76 to 1.53)
**ADHD**	Before pregnancy	22	1.14(0.75 to 1.52)
**ADHD**	Before pregnancy	23	1.13(0.75 to 1.51)
**ADHD**	Before pregnancy	24	1.12(0.74 to 1.50)
**ADHD**	Before pregnancy	25	1.11(0.73 to 1.49)
**ADHD**	Before pregnancy	26	1.10(0.73 to 1.48)
**ADHD**	Before pregnancy	27	1.10(0.73 to 1.47)
**ADHD**	Before pregnancy	28	1.10(0.72 to 1.47)

© 2026 Xiang N et al. *JAMA Network Open*.

**ADHD**	Before pregnancy	29	1.09(0.72 to 1.46)
**ADHD**	Before pregnancy	30	1.10(0.73 to 1.46)
**ADHD**	Before pregnancy	31	1.10(0.73 to 1.47)
**ADHD**	Before pregnancy	32	1.10(0.73 to 1.47)
**ADHD**	Before pregnancy	33	1.10(0.73 to 1.47)
**ADHD**	Before pregnancy	34	1.10(0.74 to 1.47)
**ADHD**	Before pregnancy	35	1.11(0.74 to 1.47)
**ADHD**	Before pregnancy	36	1.11(0.74 to 1.48)
**ADHD**	Before pregnancy	37	1.11(0.74 to 1.48)
**ADHD**	Before pregnancy	38	1.11(0.75 to 1.48)
**ADHD**	Before pregnancy	39	1.11(0.75 to 1.48)
**ADHD**	Before pregnancy	40	1.12(0.75 to 1.48)
**ADHD**	Before pregnancy	41	1.12(0.76 to 1.49)
**ADHD**	Before pregnancy	42	1.13(0.76 to 1.49)
**ADHD**	Before pregnancy	43	1.14(0.77 to 1.51)
**ADHD**	Before pregnancy	44	1.15(0.79 to 1.52)
**ADHD**	Before pregnancy	45	1.17(0.80 to 1.54)
**ADHD**	Before pregnancy	46	1.19(0.82 to 1.57)
**ADHD**	Before pregnancy	47	1.22(0.84 to 1.59)
**ADHD**	Before pregnancy	48	1.24(0.86 to 1.62)
**ADHD**	Before pregnancy	49	1.26(0.87 to 1.64)
**ADHD**	Before pregnancy	50	1.27(0.89 to 1.66)
**ADHD**	Before pregnancy	51	1.28(0.89 to 1.66)
**ADHD**	During pregnancy	1	1.20(0.84 to 1.56)
**ADHD**	During pregnancy	2	1.17(0.82 to 1.52)
**ADHD**	During pregnancy	3	1.15(0.80 to 1.50)
**ADHD**	During pregnancy	4	1.14(0.79 to 1.48)
**ADHD**	During pregnancy	5	1.13(0.78 to 1.47)
**ADHD**	During pregnancy	6	1.12(0.78 to 1.47)
**ADHD**	During pregnancy	7	1.12(0.77 to 1.46)
**ADHD**	During pregnancy	8	1.12(0.77 to 1.46)
**ADHD**	During pregnancy	9	1.12(0.78 to 1.47)
**ADHD**	During pregnancy	10	1.12(0.78 to 1.47)
**ADHD**	During pregnancy	11	1.13(0.78 to 1.47)
**ADHD**	During pregnancy	12	1.13(0.78 to 1.48)
**ADHD**	During pregnancy	13	1.14(0.79 to 1.48)
**ADHD**	During pregnancy	14	1.14(0.79 to 1.49)
**ADHD**	During pregnancy	15	1.15(0.80 to 1.50)
**ADHD**	During pregnancy	16	1.16(0.81 to 1.51)
**ADHD**	During pregnancy	17	1.17(0.81 to 1.52)
**ADHD**	During pregnancy	18	1.17(0.82 to 1.53)
**ADHD**	During pregnancy	19	1.18(0.83 to 1.53)
**ADHD**	During pregnancy	20	1.18(0.83 to 1.54)
**ADHD**	During pregnancy	21	1.19(0.83 to 1.54)
**ADHD**	During pregnancy	22	1.19(0.83 to 1.55)

© 2026 Xiang N et al. *JAMA Network Open*.

**ADHD**	During pregnancy	23	1.19(0.84 to 1.55)
**ADHD**	During pregnancy	24	1.19(0.84 to 1.55)
**ADHD**	During pregnancy	25	1.19(0.84 to 1.55)
**ADHD**	During pregnancy	26	1.19(0.83 to 1.55)
**ADHD**	During pregnancy	27	1.19(0.83 to 1.55)
**ADHD**	During pregnancy	28	1.19(0.83 to 1.55)
**ADHD**	During pregnancy	29	1.19(0.82 to 1.56)
**ADHD**	During pregnancy	30	1.18(0.81 to 1.56)
**ADHD**	During pregnancy	31	1.18(0.80 to 1.56)
**ADHD**	During pregnancy	32	1.17(0.78 to 1.56)
**ADHD**	During pregnancy	33	1.16(0.77 to 1.56)
**ADHD**	During pregnancy	34	1.15(0.75 to 1.56)
**ADHD**	During pregnancy	35	1.14(0.73 to 1.56)
**ADHD**	During pregnancy	36	1.12(0.70 to 1.56)
**ADHD**	During pregnancy	37	1.10(0.67 to 1.56)
**ADHD**	During pregnancy	38	1.09(0.64 to 1.56)
**ADHD**	During pregnancy	39	1.06(0.60 to 1.57)
**ADHD**	During pregnancy	40	1.04(0.56 to 1.57)
**ADHD**	During pregnancy	41	1.01(0.50 to 1.58)
**ADHD**	After pregnancy	1	1.16(0.81 to 1.50)
**ADHD**	After pregnancy	2	1.16(0.81 to 1.51)
**ADHD**	After pregnancy	3	1.17(0.82 to 1.52)
**ADHD**	After pregnancy	4	1.19(0.83 to 1.54)
**ADHD**	After pregnancy	5	1.20(0.84 to 1.55)
**ADHD**	After pregnancy	6	1.21(0.85 to 1.57)
**ADHD**	After pregnancy	7	1.22(0.86 to 1.58)
**ADHD**	After pregnancy	8	1.23(0.87 to 1.59)
**ADHD**	After pregnancy	9	1.24(0.88 to 1.60)
**ADHD**	After pregnancy	10	1.24(0.88 to 1.61)
**ADHD**	After pregnancy	11	1.25(0.89 to 1.61)
**ADHD**	After pregnancy	12	1.25(0.89 to 1.62)
**ADHD**	After pregnancy	13	1.25(0.89 to 1.62)
**ADHD**	After pregnancy	14	1.26(0.89 to 1.62)
**ADHD**	After pregnancy	15	1.26(0.89 to 1.62)
**ADHD**	After pregnancy	16	1.26(0.89 to 1.62)
**ADHD**	After pregnancy	17	1.25(0.89 to 1.62)
**ADHD**	After pregnancy	18	1.25(0.89 to 1.62)
**ADHD**	After pregnancy	19	1.25(0.89 to 1.62)
**ADHD**	After pregnancy	20	1.25(0.88 to 1.61)
**ADHD**	After pregnancy	21	1.25(0.88 to 1.61)
**ADHD**	After pregnancy	22	1.24(0.88 to 1.61)
**ADHD**	After pregnancy	23	1.24(0.88 to 1.60)
**ADHD**	After pregnancy	24	1.24(0.87 to 1.60)
**ADHD**	After pregnancy	25	1.23(0.87 to 1.60)
**ADHD**	After pregnancy	26	1.23(0.87 to 1.60)

© 2026 Xiang N et al. *JAMA Network Open*.

**ADHD**	After pregnancy	27	1.23(0.87 to 1.59)
**ADHD**	After pregnancy	28	1.23(0.86 to 1.59)
**ADHD**	After pregnancy	29	1.23(0.86 to 1.59)
**ADHD**	After pregnancy	30	1.22(0.86 to 1.59)
**ADHD**	After pregnancy	31	1.23(0.86 to 1.59)
**ADHD**	After pregnancy	32	1.23(0.86 to 1.59)
**ADHD**	After pregnancy	33	1.23(0.86 to 1.60)
**ADHD**	After pregnancy	34	1.23(0.87 to 1.60)
**ADHD**	After pregnancy	35	1.24(0.87 to 1.61)
**ADHD**	After pregnancy	36	1.25(0.88 to 1.62)
**ADHD**	After pregnancy	37	1.25(0.88 to 1.63)
**ADHD**	After pregnancy	38	1.26(0.89 to 1.64)
**ADHD**	After pregnancy	39	1.27(0.90 to 1.65)
**ADHD**	After pregnancy	40	1.28(0.91 to 1.66)
**ADHD**	After pregnancy	41	1.30(0.92 to 1.67)
**ADHD**	After pregnancy	42	1.31(0.93 to 1.69)
**ADHD**	After pregnancy	43	1.32(0.94 to 1.70)
**ADHD**	After pregnancy	44	1.34(0.95 to 1.72)
**ADHD**	After pregnancy	45	1.35(0.96 to 1.74)
**ADHD**	After pregnancy	46	1.36(0.97 to 1.75)
**ADHD**	After pregnancy	47	1.38(0.99 to 1.77)
**ADHD**	After pregnancy	48	1.39(1.00 to 1.78)
**ADHD**	After pregnancy	49	1.40(1.00 to 1.79)
**ADHD**	After pregnancy	50	1.41(1.01 to 1.80)
**ADHD**	After pregnancy	51	1.41(1.02 to 1.81)
**Bipolar disorder**	Before pregnancy	1	0.30(0.10 to 0.50)
**Bipolar disorder**	Before pregnancy	2	0.30(0.10 to 0.50)
**Bipolar disorder**	Before pregnancy	3	0.30(0.10 to 0.50)
**Bipolar disorder**	Before pregnancy	4	0.30(0.11 to 0.51)
**Bipolar disorder**	Before pregnancy	5	0.31(0.11 to 0.51)
**Bipolar disorder**	Before pregnancy	6	0.31(0.11 to 0.51)
**Bipolar disorder**	Before pregnancy	7	0.31(0.11 to 0.51)
**Bipolar disorder**	Before pregnancy	8	0.31(0.11 to 0.51)
**Bipolar disorder**	Before pregnancy	9	0.31(0.11 to 0.52)
**Bipolar disorder**	Before pregnancy	10	0.31(0.11 to 0.52)
**Bipolar disorder**	Before pregnancy	11	0.31(0.11 to 0.52)
**Bipolar disorder**	Before pregnancy	12	0.32(0.11 to 0.52)
**Bipolar disorder**	Before pregnancy	13	0.32(0.11 to 0.52)
**Bipolar disorder**	Before pregnancy	14	0.32(0.11 to 0.52)
**Bipolar disorder**	Before pregnancy	15	0.32(0.12 to 0.52)
**Bipolar disorder**	Before pregnancy	16	0.32(0.12 to 0.52)
**Bipolar disorder**	Before pregnancy	17	0.32(0.12 to 0.52)
**Bipolar disorder**	Before pregnancy	18	0.32(0.12 to 0.52)
**Bipolar disorder**	Before pregnancy	19	0.32(0.12 to 0.52)
**Bipolar disorder**	Before pregnancy	20	0.32(0.12 to 0.52)

© 2026 Xiang N et al. *JAMA Network Open*.

**Bipolar disorder**	Before pregnancy	21	0.32(0.12 to 0.52)
**Bipolar disorder**	Before pregnancy	22	0.32(0.12 to 0.52)
**Bipolar disorder**	Before pregnancy	23	0.31(0.12 to 0.51)
**Bipolar disorder**	Before pregnancy	24	0.31(0.11 to 0.51)
**Bipolar disorder**	Before pregnancy	25	0.31(0.11 to 0.51)
**Bipolar disorder**	Before pregnancy	26	0.31(0.11 to 0.51)
**Bipolar disorder**	Before pregnancy	27	0.31(0.11 to 0.50)
**Bipolar disorder**	Before pregnancy	28	0.31(0.11 to 0.50)
**Bipolar disorder**	Before pregnancy	29	0.30(0.11 to 0.50)
**Bipolar disorder**	Before pregnancy	30	0.30(0.11 to 0.49)
**Bipolar disorder**	Before pregnancy	31	0.30(0.11 to 0.49)
**Bipolar disorder**	Before pregnancy	32	0.30(0.11 to 0.48)
**Bipolar disorder**	Before pregnancy	33	0.29(0.11 to 0.48)
**Bipolar disorder**	Before pregnancy	34	0.29(0.10 to 0.48)
**Bipolar disorder**	Before pregnancy	35	0.29(0.10 to 0.47)
**Bipolar disorder**	Before pregnancy	36	0.28(0.10 to 0.47)
**Bipolar disorder**	Before pregnancy	37	0.28(0.10 to 0.46)
**Bipolar disorder**	Before pregnancy	38	0.28(0.10 to 0.46)
**Bipolar disorder**	Before pregnancy	39	0.28(0.10 to 0.46)
**Bipolar disorder**	Before pregnancy	40	0.27(0.10 to 0.45)
**Bipolar disorder**	Before pregnancy	41	0.27(0.10 to 0.45)
**Bipolar disorder**	Before pregnancy	42	0.27(0.09 to 0.44)
**Bipolar disorder**	Before pregnancy	43	0.27(0.09 to 0.44)
**Bipolar disorder**	Before pregnancy	44	0.26(0.09 to 0.44)
**Bipolar disorder**	Before pregnancy	45	0.26(0.09 to 0.43)
**Bipolar disorder**	Before pregnancy	46	0.26(0.09 to 0.43)
**Bipolar disorder**	Before pregnancy	47	0.26(0.09 to 0.43)
**Bipolar disorder**	Before pregnancy	48	0.26(0.09 to 0.42)
**Bipolar disorder**	Before pregnancy	49	0.25(0.09 to 0.42)
**Bipolar disorder**	Before pregnancy	50	0.25(0.09 to 0.42)
**Bipolar disorder**	Before pregnancy	51	0.25(0.09 to 0.42)
**Bipolar disorder**	During pregnancy	1	0.31(0.13 to 0.49)
**Bipolar disorder**	During pregnancy	2	0.30(0.13 to 0.48)
**Bipolar disorder**	During pregnancy	3	0.30(0.13 to 0.48)
**Bipolar disorder**	During pregnancy	4	0.30(0.13 to 0.48)
**Bipolar disorder**	During pregnancy	5	0.31(0.13 to 0.48)
**Bipolar disorder**	During pregnancy	6	0.31(0.13 to 0.49)
**Bipolar disorder**	During pregnancy	7	0.31(0.13 to 0.49)
**Bipolar disorder**	During pregnancy	8	0.31(0.13 to 0.49)
**Bipolar disorder**	During pregnancy	9	0.31(0.13 to 0.49)
**Bipolar disorder**	During pregnancy	10	0.31(0.13 to 0.49)
**Bipolar disorder**	During pregnancy	11	0.31(0.13 to 0.48)
**Bipolar disorder**	During pregnancy	12	0.30(0.13 to 0.48)
**Bipolar disorder**	During pregnancy	13	0.30(0.12 to 0.47)
**Bipolar disorder**	During pregnancy	14	0.29(0.12 to 0.47)

© 2026 Xiang N et al. *JAMA Network Open*.

**Bipolar disorder**	During pregnancy	15	0.29(0.12 to 0.46)
**Bipolar disorder**	During pregnancy	16	0.29(0.12 to 0.46)
**Bipolar disorder**	During pregnancy	17	0.29(0.12 to 0.46)
**Bipolar disorder**	During pregnancy	18	0.28(0.11 to 0.45)
**Bipolar disorder**	During pregnancy	19	0.28(0.11 to 0.45)
**Bipolar disorder**	During pregnancy	20	0.28(0.11 to 0.45)
**Bipolar disorder**	During pregnancy	21	0.27(0.11 to 0.44)
**Bipolar disorder**	During pregnancy	22	0.27(0.10 to 0.43)
**Bipolar disorder**	During pregnancy	23	0.27(0.10 to 0.43)
**Bipolar disorder**	During pregnancy	24	0.26(0.10 to 0.43)
**Bipolar disorder**	During pregnancy	25	0.26(0.10 to 0.42)
**Bipolar disorder**	During pregnancy	26	0.26(0.09 to 0.42)
**Bipolar disorder**	During pregnancy	27	0.26(0.09 to 0.42)
**Bipolar disorder**	During pregnancy	28	0.26(0.09 to 0.43)
**Bipolar disorder**	During pregnancy	29	0.26(0.09 to 0.44)
**Bipolar disorder**	During pregnancy	30	0.27(0.09 to 0.45)
**Bipolar disorder**	During pregnancy	31	0.28(0.09 to 0.47)
**Bipolar disorder**	During pregnancy	32	0.28(0.09 to 0.49)
**Bipolar disorder**	During pregnancy	33	0.29(0.09 to 0.51)
**Bipolar disorder**	During pregnancy	34	0.30(0.09 to 0.53)
**Bipolar disorder**	During pregnancy	35	0.31(0.08 to 0.55)
**Bipolar disorder**	During pregnancy	36	0.32(0.08 to 0.58)
**Bipolar disorder**	During pregnancy	37	0.33(0.07 to 0.61)
**Bipolar disorder**	During pregnancy	38	0.34(0.07 to 0.65)
**Bipolar disorder**	During pregnancy	39	0.35(0.06 to 0.69)
**Bipolar disorder**	During pregnancy	40	0.37(0.05 to 0.75)
**Bipolar disorder**	During pregnancy	41	0.39(0.04 to 0.81)
**Bipolar disorder**	After pregnancy	1	0.34(0.15 to 0.52)
**Bipolar disorder**	After pregnancy	2	0.34(0.15 to 0.52)
**Bipolar disorder**	After pregnancy	3	0.34(0.15 to 0.53)
**Bipolar disorder**	After pregnancy	4	0.34(0.16 to 0.53)
**Bipolar disorder**	After pregnancy	5	0.35(0.16 to 0.53)
**Bipolar disorder**	After pregnancy	6	0.35(0.16 to 0.54)
**Bipolar disorder**	After pregnancy	7	0.35(0.16 to 0.54)
**Bipolar disorder**	After pregnancy	8	0.35(0.16 to 0.55)
**Bipolar disorder**	After pregnancy	9	0.36(0.17 to 0.55)
**Bipolar disorder**	After pregnancy	10	0.36(0.17 to 0.55)
**Bipolar disorder**	After pregnancy	11	0.36(0.17 to 0.56)
**Bipolar disorder**	After pregnancy	12	0.37(0.17 to 0.56)
**Bipolar disorder**	After pregnancy	13	0.37(0.18 to 0.57)
**Bipolar disorder**	After pregnancy	14	0.38(0.18 to 0.57)
**Bipolar disorder**	After pregnancy	15	0.38(0.18 to 0.58)
**Bipolar disorder**	After pregnancy	16	0.38(0.18 to 0.58)
**Bipolar disorder**	After pregnancy	17	0.39(0.18 to 0.59)
**Bipolar disorder**	After pregnancy	18	0.39(0.19 to 0.59)

© 2026 Xiang N et al. *JAMA Network Open*.

**Bipolar disorder**	After pregnancy	19	0.39(0.19 to 0.59)
**Bipolar disorder**	After pregnancy	20	0.39(0.19 to 0.60)
**Bipolar disorder**	After pregnancy	21	0.40(0.19 to 0.60)
**Bipolar disorder**	After pregnancy	22	0.40(0.20 to 0.61)
**Bipolar disorder**	After pregnancy	23	0.40(0.20 to 0.61)
**Bipolar disorder**	After pregnancy	24	0.41(0.20 to 0.62)
**Bipolar disorder**	After pregnancy	25	0.41(0.20 to 0.62)
**Bipolar disorder**	After pregnancy	26	0.42(0.21 to 0.62)
**Bipolar disorder**	After pregnancy	27	0.42(0.21 to 0.63)
**Bipolar disorder**	After pregnancy	28	0.42(0.21 to 0.63)
**Bipolar disorder**	After pregnancy	29	0.42(0.21 to 0.63)
**Bipolar disorder**	After pregnancy	30	0.42(0.21 to 0.64)
**Bipolar disorder**	After pregnancy	31	0.42(0.21 to 0.64)
**Bipolar disorder**	After pregnancy	32	0.42(0.21 to 0.64)
**Bipolar disorder**	After pregnancy	33	0.42(0.21 to 0.64)
**Bipolar disorder**	After pregnancy	34	0.42(0.21 to 0.64)
**Bipolar disorder**	After pregnancy	35	0.42(0.21 to 0.64)
**Bipolar disorder**	After pregnancy	36	0.42(0.21 to 0.64)
**Bipolar disorder**	After pregnancy	37	0.42(0.21 to 0.64)
**Bipolar disorder**	After pregnancy	38	0.42(0.21 to 0.64)
**Bipolar disorder**	After pregnancy	39	0.42(0.21 to 0.64)
**Bipolar disorder**	After pregnancy	40	0.42(0.21 to 0.64)
**Bipolar disorder**	After pregnancy	41	0.42(0.21 to 0.64)
**Bipolar disorder**	After pregnancy	42	0.42(0.21 to 0.64)
**Bipolar disorder**	After pregnancy	43	0.42(0.21 to 0.64)
**Bipolar disorder**	After pregnancy	44	0.42(0.21 to 0.63)
**Bipolar disorder**	After pregnancy	45	0.42(0.21 to 0.63)
**Bipolar disorder**	After pregnancy	46	0.42(0.20 to 0.63)
**Bipolar disorder**	After pregnancy	47	0.41(0.20 to 0.63)
**Bipolar disorder**	After pregnancy	48	0.41(0.20 to 0.62)
**Bipolar disorder**	After pregnancy	49	0.41(0.20 to 0.62)
**Bipolar disorder**	After pregnancy	50	0.41(0.20 to 0.62)
**Bipolar disorder**	After pregnancy	51	0.41(0.20 to 0.62)
**Psychosis**	Before pregnancy	1	0.28(0.08 to 0.48)
**Psychosis**	Before pregnancy	2	0.27(0.08 to 0.47)
**Psychosis**	Before pregnancy	3	0.27(0.08 to 0.46)
**Psychosis**	Before pregnancy	4	0.26(0.08 to 0.45)
**Psychosis**	Before pregnancy	5	0.26(0.07 to 0.44)
**Psychosis**	Before pregnancy	6	0.25(0.07 to 0.44)
**Psychosis**	Before pregnancy	7	0.25(0.07 to 0.43)
**Psychosis**	Before pregnancy	8	0.25(0.07 to 0.43)
**Psychosis**	Before pregnancy	9	0.25(0.07 to 0.43)
**Psychosis**	Before pregnancy	10	0.24(0.07 to 0.42)
**Psychosis**	Before pregnancy	11	0.24(0.07 to 0.42)
**Psychosis**	Before pregnancy	12	0.24(0.07 to 0.42)

© 2026 Xiang N et al. *JAMA Network Open*.

**Psychosis**	Before pregnancy	13	0.24(0.07 to 0.42)
**Psychosis**	Before pregnancy	14	0.24(0.07 to 0.41)
**Psychosis**	Before pregnancy	15	0.24(0.06 to 0.41)
**Psychosis**	Before pregnancy	16	0.24(0.06 to 0.41)
**Psychosis**	Before pregnancy	17	0.24(0.06 to 0.41)
**Psychosis**	Before pregnancy	18	0.23(0.06 to 0.41)
**Psychosis**	Before pregnancy	19	0.23(0.06 to 0.41)
**Psychosis**	Before pregnancy	20	0.23(0.06 to 0.40)
**Psychosis**	Before pregnancy	21	0.23(0.06 to 0.40)
**Psychosis**	Before pregnancy	22	0.23(0.06 to 0.40)
**Psychosis**	Before pregnancy	23	0.23(0.06 to 0.40)
**Psychosis**	Before pregnancy	24	0.23(0.06 to 0.40)
**Psychosis**	Before pregnancy	25	0.23(0.06 to 0.40)
**Psychosis**	Before pregnancy	26	0.24(0.07 to 0.41)
**Psychosis**	Before pregnancy	27	0.24(0.07 to 0.41)
**Psychosis**	Before pregnancy	28	0.24(0.07 to 0.41)
**Psychosis**	Before pregnancy	29	0.24(0.07 to 0.41)
**Psychosis**	Before pregnancy	30	0.24(0.07 to 0.41)
**Psychosis**	Before pregnancy	31	0.24(0.07 to 0.41)
**Psychosis**	Before pregnancy	32	0.24(0.07 to 0.41)
**Psychosis**	Before pregnancy	33	0.24(0.07 to 0.41)
**Psychosis**	Before pregnancy	34	0.24(0.07 to 0.40)
**Psychosis**	Before pregnancy	35	0.23(0.07 to 0.40)
**Psychosis**	Before pregnancy	36	0.23(0.07 to 0.40)
**Psychosis**	Before pregnancy	37	0.23(0.07 to 0.40)
**Psychosis**	Before pregnancy	38	0.23(0.07 to 0.39)
**Psychosis**	Before pregnancy	39	0.23(0.06 to 0.39)
**Psychosis**	Before pregnancy	40	0.23(0.06 to 0.39)
**Psychosis**	Before pregnancy	41	0.22(0.06 to 0.39)
**Psychosis**	Before pregnancy	42	0.22(0.06 to 0.38)
**Psychosis**	Before pregnancy	43	0.22(0.06 to 0.38)
**Psychosis**	Before pregnancy	44	0.22(0.06 to 0.38)
**Psychosis**	Before pregnancy	45	0.21(0.06 to 0.37)
**Psychosis**	Before pregnancy	46	0.21(0.05 to 0.36)
**Psychosis**	Before pregnancy	47	0.20(0.05 to 0.36)
**Psychosis**	Before pregnancy	48	0.20(0.05 to 0.35)
**Psychosis**	Before pregnancy	49	0.19(0.04 to 0.34)
**Psychosis**	Before pregnancy	50	0.18(0.04 to 0.33)
**Psychosis**	Before pregnancy	51	0.18(0.03 to 0.32)
**Psychosis**	During pregnancy	1	0.26(0.09 to 0.42)
**Psychosis**	During pregnancy	2	0.26(0.10 to 0.43)
**Psychosis**	During pregnancy	3	0.26(0.10 to 0.43)
**Psychosis**	During pregnancy	4	0.27(0.10 to 0.44)
**Psychosis**	During pregnancy	5	0.27(0.10 to 0.44)
**Psychosis**	During pregnancy	6	0.28(0.11 to 0.45)

© 2026 Xiang N et al. *JAMA Network Open*.

**Psychosis**	During pregnancy	7	0.28(0.11 to 0.45)
**Psychosis**	During pregnancy	8	0.28(0.11 to 0.45)
**Psychosis**	During pregnancy	9	0.28(0.11 to 0.46)
**Psychosis**	During pregnancy	10	0.28(0.11 to 0.46)
**Psychosis**	During pregnancy	11	0.29(0.11 to 0.46)
**Psychosis**	During pregnancy	12	0.29(0.11 to 0.46)
**Psychosis**	During pregnancy	13	0.29(0.11 to 0.46)
**Psychosis**	During pregnancy	14	0.29(0.12 to 0.46)
**Psychosis**	During pregnancy	15	0.29(0.12 to 0.46)
**Psychosis**	During pregnancy	16	0.29(0.12 to 0.46)
**Psychosis**	During pregnancy	17	0.29(0.12 to 0.47)
**Psychosis**	During pregnancy	18	0.29(0.12 to 0.47)
**Psychosis**	During pregnancy	19	0.29(0.12 to 0.47)
**Psychosis**	During pregnancy	20	0.29(0.12 to 0.46)
**Psychosis**	During pregnancy	21	0.29(0.12 to 0.46)
**Psychosis**	During pregnancy	22	0.29(0.12 to 0.46)
**Psychosis**	During pregnancy	23	0.29(0.11 to 0.46)
**Psychosis**	During pregnancy	24	0.29(0.11 to 0.46)
**Psychosis**	During pregnancy	25	0.29(0.11 to 0.46)
**Psychosis**	During pregnancy	26	0.28(0.11 to 0.46)
**Psychosis**	During pregnancy	27	0.28(0.11 to 0.46)
**Psychosis**	During pregnancy	28	0.28(0.11 to 0.45)
**Psychosis**	During pregnancy	29	0.28(0.11 to 0.45)
**Psychosis**	During pregnancy	30	0.27(0.10 to 0.44)
**Psychosis**	During pregnancy	31	0.27(0.10 to 0.44)
**Psychosis**	During pregnancy	32	0.26(0.09 to 0.43)
**Psychosis**	During pregnancy	33	0.25(0.09 to 0.42)
**Psychosis**	During pregnancy	34	0.24(0.08 to 0.41)
**Psychosis**	During pregnancy	35	0.23(0.08 to 0.39)
**Psychosis**	During pregnancy	36	0.22(0.07 to 0.38)
**Psychosis**	During pregnancy	37	0.21(0.06 to 0.36)
**Psychosis**	During pregnancy	38	0.19(0.05 to 0.34)
**Psychosis**	During pregnancy	39	0.17(0.04 to 0.32)
**Psychosis**	During pregnancy	40	0.15(0.03 to 0.29)
**Psychosis**	During pregnancy	41	0.12(0.01 to 0.25)
**Psychosis**	After pregnancy	1	0.31(0.13 to 0.48)
**Psychosis**	After pregnancy	2	0.30(0.12 to 0.47)
**Psychosis**	After pregnancy	3	0.28(0.12 to 0.45)
**Psychosis**	After pregnancy	4	0.28(0.11 to 0.44)
**Psychosis**	After pregnancy	5	0.27(0.11 to 0.43)
**Psychosis**	After pregnancy	6	0.26(0.10 to 0.42)
**Psychosis**	After pregnancy	7	0.26(0.10 to 0.42)
**Psychosis**	After pregnancy	8	0.25(0.10 to 0.41)
**Psychosis**	After pregnancy	9	0.25(0.09 to 0.41)
**Psychosis**	After pregnancy	10	0.25(0.09 to 0.41)

© 2026 Xiang N et al. *JAMA Network Open*.

**Psychosis**	After pregnancy	11	0.25(0.09 to 0.40)
**Psychosis**	After pregnancy	12	0.25(0.09 to 0.40)
**Psychosis**	After pregnancy	13	0.24(0.09 to 0.40)
**Psychosis**	After pregnancy	14	0.24(0.09 to 0.40)
**Psychosis**	After pregnancy	15	0.24(0.09 to 0.40)
**Psychosis**	After pregnancy	16	0.24(0.09 to 0.40)
**Psychosis**	After pregnancy	17	0.24(0.09 to 0.40)
**Psychosis**	After pregnancy	18	0.25(0.09 to 0.40)
**Psychosis**	After pregnancy	19	0.25(0.09 to 0.41)
**Psychosis**	After pregnancy	20	0.25(0.09 to 0.41)
**Psychosis**	After pregnancy	21	0.25(0.09 to 0.41)
**Psychosis**	After pregnancy	22	0.26(0.10 to 0.42)
**Psychosis**	After pregnancy	23	0.26(0.10 to 0.43)
**Psychosis**	After pregnancy	24	0.27(0.10 to 0.43)
**Psychosis**	After pregnancy	25	0.27(0.10 to 0.44)
**Psychosis**	After pregnancy	26	0.27(0.11 to 0.44)
**Psychosis**	After pregnancy	27	0.28(0.11 to 0.45)
**Psychosis**	After pregnancy	28	0.28(0.11 to 0.45)
**Psychosis**	After pregnancy	29	0.29(0.11 to 0.46)
**Psychosis**	After pregnancy	30	0.29(0.11 to 0.46)
**Psychosis**	After pregnancy	31	0.29(0.12 to 0.46)
**Psychosis**	After pregnancy	32	0.29(0.12 to 0.47)
**Psychosis**	After pregnancy	33	0.29(0.12 to 0.47)
**Psychosis**	After pregnancy	34	0.30(0.12 to 0.47)
**Psychosis**	After pregnancy	35	0.30(0.12 to 0.48)
**Psychosis**	After pregnancy	36	0.30(0.12 to 0.48)
**Psychosis**	After pregnancy	37	0.30(0.12 to 0.48)
**Psychosis**	After pregnancy	38	0.31(0.13 to 0.49)
**Psychosis**	After pregnancy	39	0.31(0.13 to 0.49)
**Psychosis**	After pregnancy	40	0.31(0.13 to 0.49)
**Psychosis**	After pregnancy	41	0.31(0.13 to 0.49)
**Psychosis**	After pregnancy	42	0.31(0.13 to 0.50)
**Psychosis**	After pregnancy	43	0.31(0.13 to 0.50)
**Psychosis**	After pregnancy	44	0.31(0.13 to 0.50)
**Psychosis**	After pregnancy	45	0.31(0.13 to 0.50)
**Psychosis**	After pregnancy	46	0.31(0.13 to 0.50)
**Psychosis**	After pregnancy	47	0.31(0.13 to 0.50)
**Psychosis**	After pregnancy	48	0.32(0.13 to 0.50)
**Psychosis**	After pregnancy	49	0.32(0.13 to 0.50)
**Psychosis**	After pregnancy	50	0.32(0.14 to 0.51)
**Psychosis**	After pregnancy	51	0.33(0.14 to 0.51)

*ADHD, attention deficit hyperactivity disorder.

*Incidence rate was standardized by age, calendar year at childbirth and week at follow-up.

© 2026 Xiang N et al. *JAMA Network Open*.

eTable 4. Incidence Rate Ratios of Any Paternal Psychiatric Disorder and 9 Type-Specific Disorders During and After Pregnancy

Psychiatric disorders	Phase	Weeks	Incidence rate differences per 1000 person-years		
			Model 1	Model 2	Model 3
Any psychiatric disorder	During pregnancy	0-4	0.80(0.73 to 0.88)	0.78(0.71 to 0.85)	0.79(0.72 to 0.86)
Any psychiatric disorder	During pregnancy	5-9	0.79(0.72 to 0.87)	0.77(0.70 to 0.84)	0.77(0.70 to 0.85)
Any psychiatric disorder	During pregnancy	10-14	0.86(0.78 to 0.94)	0.83(0.76 to 0.91)	0.84(0.76 to 0.92)
Any psychiatric disorder	During pregnancy	15-19	0.86(0.78 to 0.94)	0.83(0.76 to 0.91)	0.84(0.76 to 0.92)
Any psychiatric disorder	During pregnancy	20-24	0.92(0.83 to 1.01)	0.90(0.82 to 0.99)	0.91(0.83 to 1.00)
Any psychiatric disorder	During pregnancy	25-29	0.83(0.76 to 0.92)	0.81(0.74 to 0.89)	0.81(0.74 to 0.90)
Any psychiatric disorder	During pregnancy	30-34	0.83(0.76 to 0.91)	0.82(0.75 to 0.90)	0.82(0.75 to 0.91)
Any psychiatric disorder	During pregnancy	35-39	0.76(0.69 to 0.84)	0.75(0.68 to 0.83)	0.75(0.68 to 0.83)
Any psychiatric disorder	After pregnancy	0-4	0.74(0.67 to 0.81)	0.72(0.65 to 0.79)	0.73(0.66 to 0.80)
Any psychiatric disorder	After pregnancy	5-9	0.80(0.73 to 0.88)	0.76(0.69 to 0.84)	0.77(0.70 to 0.85)
Any psychiatric disorder	After pregnancy	10-14	0.86(0.78 to 0.94)	0.82(0.75 to 0.90)	0.83(0.76 to 0.91)
Any psychiatric disorder	After pregnancy	15-19	0.86(0.79 to 0.94)	0.83(0.76 to 0.91)	0.84(0.76 to 0.92)
Any psychiatric disorder	After pregnancy	20-24	0.92(0.84 to 1.02)	0.89(0.81 to 0.98)	0.90(0.82 to 0.99)
Any psychiatric disorder	After pregnancy	25-29	0.98(0.89 to 1.08)	0.97(0.88 to 1.06)	0.97(0.89 to 1.07)
Any psychiatric disorder	After pregnancy	30-34	0.96(0.87 to 1.05)	0.95(0.87 to 1.04)	0.96(0.87 to 1.05)
Any psychiatric disorder	After pregnancy	35-39	1.00(0.91 to 1.09)	0.98(0.90 to 1.07)	0.98(0.90 to 1.08)
Any psychiatric disorder	After pregnancy	40-44	0.94(0.86 to 1.03)	0.93(0.85 to 1.02)	0.93(0.85 to 1.02)
Any psychiatric disorder	After pregnancy	45-49	1.06(0.97 to 1.16)	1.04(0.95 to 1.14)	1.05(0.95 to 1.15)
Any psychiatric disorder	After pregnancy	50-51	0.98(0.84 to 1.13)	0.96(0.83 to 1.12)	0.97(0.83 to 1.12)
Depression	During pregnancy	0-4	0.79(0.68 to 0.92)	0.76(0.65 to 0.89)	0.78(0.67 to 0.91)
Depression	During pregnancy	5-9	0.80(0.68 to 0.93)	0.77(0.66 to 0.90)	0.78(0.67 to 0.91)
Depression	During pregnancy	10-14	0.83(0.71 to 0.97)	0.81(0.70 to 0.95)	0.82(0.71 to 0.96)
Depression	During pregnancy	15-19	0.90(0.78 to 1.05)	0.88(0.75 to 1.02)	0.88(0.76 to 1.03)
Depression	During pregnancy	20-24	0.95(0.81 to 1.11)	0.92(0.78 to 1.08)	0.92(0.79 to 1.08)
Depression	During pregnancy	25-29	0.74(0.63 to 0.87)	0.73(0.62 to 0.85)	0.73(0.63 to 0.86)
Depression	During pregnancy	30-34	0.75(0.64 to 0.88)	0.73(0.62 to 0.86)	0.74(0.63 to 0.86)
Depression	During pregnancy	35-39	0.70(0.60 to 0.83)	0.71(0.60 to 0.84)	0.71(0.60 to 0.83)
Depression	After pregnancy	0-4	0.68(0.58 to 0.80)	0.66(0.56 to 0.78)	0.68(0.58 to 0.80)
Depression	After pregnancy	5-9	0.74(0.63 to 0.86)	0.72(0.61 to 0.84)	0.73(0.63 to 0.85)
Depression	After pregnancy	10-14	0.89(0.77 to 1.04)	0.86(0.74 to 1.00)	0.87(0.75 to 1.01)
Depression	After pregnancy	15-19	0.99(0.85 to 1.15)	0.97(0.84 to 1.13)	0.98(0.85 to 1.14)
Depression	After pregnancy	20-24	0.99(0.85 to 1.16)	0.98(0.84 to 1.15)	0.99(0.85 to 1.16)
Depression	After pregnancy	25-29	1.05(0.90 to 1.21)	1.02(0.88 to 1.18)	1.03(0.89 to 1.20)
Depression	After pregnancy	30-34	1.04(0.90 to 1.20)	1.03(0.89 to 1.19)	1.04(0.90 to 1.20)
Depression	After pregnancy	35-39	1.07(0.93 to 1.23)	1.06(0.92 to 1.22)	1.06(0.92 to 1.22)
Depression	After pregnancy	40-44	1.16(1.00 to 1.34)	1.14(0.98 to 1.32)	1.14(0.98 to 1.32)
Depression	After pregnancy	45-49	1.29(1.11 to 1.50)	1.29(1.11 to 1.51)	1.30(1.12 to 1.52)
Depression	After pregnancy	50-51	1.39(1.08 to 1.78)	1.35(1.05 to 1.74)	1.37(1.06 to 1.76)
Anxiety	During pregnancy	0-4	0.92(0.80 to 1.06)	0.88(0.77 to 1.02)	0.90(0.78 to 1.03)
Anxiety	During pregnancy	5-9	0.83(0.72 to 0.96)	0.81(0.70 to 0.94)	0.83(0.72 to 0.96)

© 2026 Xiang N et al. *JAMA Network Open*.

Anxiety	During pregnancy	10-14	0.86(0.74 to 0.99)	0.83(0.72 to 0.96)	0.85(0.73 to 0.98)
Anxiety	During pregnancy	15-19	0.84(0.73 to 0.97)	0.82(0.71 to 0.95)	0.84(0.72 to 0.97)
Anxiety	During pregnancy	20-24	1.08(0.93 to 1.24)	1.05(0.91 to 1.22)	1.07(0.92 to 1.23)
Anxiety	During pregnancy	25-29	1.05(0.91 to 1.21)	1.03(0.89 to 1.19)	1.04(0.90 to 1.20)
Anxiety	During pregnancy	30-34	1.02(0.88 to 1.18)	1.00(0.87 to 1.16)	1.01(0.87 to 1.17)
Anxiety	During pregnancy	35-39	0.86(0.74 to 0.99)	0.85(0.73 to 0.99)	0.86(0.74 to 0.99)
Anxiety	After pregnancy	0-4	0.84(0.73 to 0.96)	0.80(0.70 to 0.93)	0.82(0.71 to 0.95)
Anxiety	After pregnancy	5-9	0.84(0.72 to 0.97)	0.82(0.71 to 0.95)	0.84(0.73 to 0.98)
Anxiety	After pregnancy	10-14	0.88(0.76 to 1.02)	0.85(0.74 to 0.99)	0.87(0.75 to 1.00)
Anxiety	After pregnancy	15-19	0.99(0.86 to 1.14)	0.96(0.83 to 1.11)	0.98(0.85 to 1.13)
Anxiety	After pregnancy	20-24	1.15(1.00 to 1.33)	1.12(0.97 to 1.29)	1.14(0.99 to 1.31)
Anxiety	After pregnancy	25-29	1.17(1.02 to 1.35)	1.14(0.99 to 1.31)	1.15(1.00 to 1.33)
Anxiety	After pregnancy	30-34	1.05(0.91 to 1.21)	1.05(0.91 to 1.21)	1.06(0.92 to 1.23)
Anxiety	After pregnancy	35-39	1.06(0.93 to 1.21)	1.05(0.92 to 1.21)	1.06(0.93 to 1.22)
Anxiety	After pregnancy	40-44	0.95(0.83 to 1.09)	0.95(0.82 to 1.09)	0.96(0.83 to 1.10)
Anxiety	After pregnancy	45-49	1.10(0.96 to 1.27)	1.09(0.95 to 1.26)	1.10(0.96 to 1.27)
Anxiety	After pregnancy	50-51	0.96(0.77 to 1.19)	0.94(0.75 to 1.17)	0.95(0.76 to 1.18)
Stress-related disorder	During pregnancy	0-4	0.81(0.69 to 0.96)	0.78(0.66 to 0.92)	0.79(0.67 to 0.94)
Stress-related disorder	During pregnancy	5-9	0.78(0.65 to 0.93)	0.74(0.62 to 0.88)	0.75(0.63 to 0.89)
Stress-related disorder	During pregnancy	10-14	0.89(0.75 to 1.06)	0.86(0.73 to 1.02)	0.88(0.74 to 1.04)
Stress-related disorder	During pregnancy	15-19	0.85(0.72 to 1.01)	0.82(0.70 to 0.97)	0.83(0.70 to 0.98)
Stress-related disorder	During pregnancy	20-24	0.86(0.73 to 1.02)	0.84(0.71 to 0.99)	0.85(0.72 to 1.01)
Stress-related disorder	During pregnancy	25-29	0.79(0.67 to 0.94)	0.76(0.64 to 0.91)	0.77(0.65 to 0.92)
Stress-related disorder	During pregnancy	30-34	0.74(0.62 to 0.88)	0.72(0.60 to 0.86)	0.72(0.60 to 0.86)
Stress-related disorder	During pregnancy	35-39	0.85(0.71 to 1.01)	0.82(0.68 to 0.98)	0.83(0.69 to 0.99)
Stress-related disorder	After pregnancy	0-4	0.95(0.81 to 1.11)	0.91(0.77 to 1.06)	0.92(0.79 to 1.08)
Stress-related disorder	After pregnancy	5-9	1.05(0.89 to 1.24)	1.03(0.87 to 1.21)	1.04(0.88 to 1.23)
Stress-related disorder	After pregnancy	10-14	1.08(0.92 to 1.26)	1.03(0.87 to 1.21)	1.05(0.89 to 1.23)
Stress-related disorder	After pregnancy	15-19	0.96(0.81 to 1.12)	0.93(0.79 to 1.09)	0.94(0.80 to 1.10)
Stress-related disorder	After pregnancy	20-24	0.97(0.83 to 1.14)	0.92(0.78 to 1.08)	0.93(0.79 to 1.10)
Stress-related disorder	After pregnancy	25-29	1.14(0.97 to 1.34)	1.11(0.95 to 1.30)	1.13(0.96 to 1.32)
Stress-related disorder	After pregnancy	30-34	1.23(1.05 to 1.44)	1.22(1.04 to 1.43)	1.22(1.04 to 1.43)
Stress-related disorder	After pregnancy	35-39	1.28(1.10 to 1.50)	1.25(1.07 to 1.46)	1.27(1.08 to 1.48)
Stress-related disorder	After pregnancy	40-44	1.09(0.93 to 1.28)	1.08(0.92 to 1.26)	1.08(0.92 to 1.27)
Stress-related disorder	After pregnancy	45-49	1.38(1.17 to 1.63)	1.36(1.15 to 1.61)	1.36(1.15 to 1.61)
Stress-related disorder	After pregnancy	50-51	1.40(1.07 to 1.83)	1.40(1.07 to 1.84)	1.40(1.06 to 1.83)
Alcohol use disorder	During pregnancy	0-4	0.58(0.48 to 0.70)	0.57(0.47 to 0.69)	0.58(0.48 to 0.70)
Alcohol use disorder	During pregnancy	5-9	0.66(0.55 to 0.80)	0.65(0.53 to 0.78)	0.66(0.55 to 0.80)
Alcohol use disorder	During pregnancy	10-14	0.77(0.63 to 0.93)	0.75(0.62 to 0.91)	0.77(0.63 to 0.93)
Alcohol use disorder	During pregnancy	15-19	0.63(0.52 to 0.77)	0.62(0.51 to 0.76)	0.63(0.52 to 0.77)
Alcohol use disorder	During pregnancy	20-24	0.64(0.53 to 0.77)	0.63(0.52 to 0.77)	0.64(0.53 to 0.78)
Alcohol use disorder	During pregnancy	25-29	0.78(0.63 to 0.97)	0.76(0.61 to 0.94)	0.76(0.62 to 0.95)
Alcohol use disorder	During pregnancy	30-34	0.68(0.55 to 0.83)	0.68(0.56 to 0.84)	0.69(0.56 to 0.85)
Alcohol use disorder	During pregnancy	35-39	0.54(0.43 to 0.68)	0.53(0.42 to 0.67)	0.53(0.42 to 0.67)
Alcohol use disorder	After pregnancy	0-4	0.44(0.36 to 0.54)	0.43(0.35 to 0.53)	0.44(0.36 to 0.54)

© 2026 Xiang N et al. *JAMA Network Open*.

Alcohol use disorder	After pregnancy	5-9	0.56(0.46 to 0.69)	0.54(0.44 to 0.66)	0.56(0.45 to 0.68)
Alcohol use disorder	After pregnancy	10-14	0.72(0.59 to 0.88)	0.71(0.58 to 0.87)	0.73(0.60 to 0.89)
Alcohol use disorder	After pregnancy	15-19	0.67(0.55 to 0.81)	0.66(0.54 to 0.80)	0.67(0.55 to 0.81)
Alcohol use disorder	After pregnancy	20-24	0.54(0.44 to 0.66)	0.53(0.43 to 0.65)	0.54(0.44 to 0.66)
Alcohol use disorder	After pregnancy	25-29	0.93(0.76 to 1.15)	0.90(0.73 to 1.11)	0.91(0.74 to 1.12)
Alcohol use disorder	After pregnancy	30-34	0.85(0.70 to 1.03)	0.84(0.69 to 1.03)	0.85(0.70 to 1.04)
Alcohol use disorder	After pregnancy	35-39	0.86(0.71 to 1.04)	0.84(0.70 to 1.02)	0.85(0.70 to 1.03)
Alcohol use disorder	After pregnancy	40-44	0.81(0.67 to 0.99)	0.80(0.66 to 0.97)	0.81(0.67 to 0.98)
Alcohol use disorder	After pregnancy	45-49	0.90(0.74 to 1.09)	0.88(0.73 to 1.08)	0.90(0.74 to 1.09)
Alcohol use disorder	After pregnancy	50-51	0.95(0.70 to 1.28)	0.93(0.69 to 1.26)	0.94(0.69 to 1.27)
Tobacco use disorder	During pregnancy	0-4	1.22(0.73 to 2.03)	1.14(0.68 to 1.90)	1.14(0.68 to 1.90)
Tobacco use disorder	During pregnancy	5-9	1.21(0.74 to 1.97)	1.14(0.69 to 1.86)	1.15(0.70 to 1.88)
Tobacco use disorder	During pregnancy	10-14	1.14(0.74 to 1.75)	1.08(0.70 to 1.66)	1.10(0.71 to 1.69)
Tobacco use disorder	During pregnancy	15-19	0.79(0.51 to 1.23)	0.75(0.48 to 1.17)	0.76(0.49 to 1.18)
Tobacco use disorder	During pregnancy	20-24	0.88(0.56 to 1.37)	0.86(0.55 to 1.34)	0.86(0.55 to 1.34)
Tobacco use disorder	During pregnancy	25-29	1.03(0.63 to 1.67)	0.91(0.56 to 1.51)	0.91(0.55 to 1.50)
Tobacco use disorder	During pregnancy	30-34	1.21(0.79 to 1.87)	1.17(0.76 to 1.81)	1.18(0.77 to 1.83)
Tobacco use disorder	During pregnancy	35-39	1.14(0.73 to 1.79)	1.07(0.68 to 1.68)	1.08(0.69 to 1.70)
Tobacco use disorder	After pregnancy	0-4	1.45(0.89 to 2.38)	1.38(0.84 to 2.25)	1.38(0.84 to 2.26)
Tobacco use disorder	After pregnancy	5-9	1.23(0.75 to 2.00)	1.11(0.67 to 1.82)	1.12(0.68 to 1.84)
Tobacco use disorder	After pregnancy	10-14	0.96(0.61 to 1.49)	0.89(0.57 to 1.40)	0.91(0.58 to 1.42)
Tobacco use disorder	After pregnancy	15-19	0.95(0.62 to 1.45)	0.90(0.59 to 1.37)	0.91(0.59 to 1.39)
Tobacco use disorder	After pregnancy	20-24	1.08(0.71 to 1.66)	1.02(0.66 to 1.57)	1.03(0.67 to 1.58)
Tobacco use disorder	After pregnancy	25-29	1.21(0.76 to 1.95)	1.14(0.71 to 1.84)	1.14(0.71 to 1.84)
Tobacco use disorder	After pregnancy	30-34	1.35(0.89 to 2.07)	1.30(0.85 to 1.99)	1.32(0.86 to 2.02)
Tobacco use disorder	After pregnancy	35-39	1.14(0.74 to 1.76)	1.13(0.73 to 1.75)	1.16(0.75 to 1.79)
Tobacco use disorder	After pregnancy	40-44	1.21(0.83 to 1.76)	1.19(0.81 to 1.73)	1.19(0.82 to 1.74)
Tobacco use disorder	After pregnancy	45-49	1.18(0.77 to 1.81)	1.16(0.75 to 1.79)	1.15(0.75 to 1.78)
Tobacco use disorder	After pregnancy	50-51	2.05(0.89 to 4.72)	1.93(0.83 to 4.48)	1.93(0.83 to 4.48)
Drug use disorder	During pregnancy	0-4	0.65(0.53 to 0.81)	0.62(0.50 to 0.77)	0.65(0.52 to 0.80)
Drug use disorder	During pregnancy	5-9	0.76(0.61 to 0.95)	0.72(0.58 to 0.90)	0.75(0.60 to 0.93)
Drug use disorder	During pregnancy	10-14	0.74(0.60 to 0.92)	0.72(0.58 to 0.88)	0.74(0.60 to 0.91)
Drug use disorder	During pregnancy	15-19	0.76(0.62 to 0.94)	0.74(0.60 to 0.91)	0.77(0.62 to 0.94)
Drug use disorder	During pregnancy	20-24	0.76(0.61 to 0.94)	0.73(0.59 to 0.91)	0.75(0.61 to 0.93)
Drug use disorder	During pregnancy	25-29	0.75(0.60 to 0.92)	0.71(0.57 to 0.88)	0.73(0.59 to 0.90)
Drug use disorder	During pregnancy	30-34	0.87(0.70 to 1.09)	0.86(0.69 to 1.07)	0.88(0.70 to 1.09)
Drug use disorder	During pregnancy	35-39	0.74(0.58 to 0.95)	0.73(0.58 to 0.94)	0.75(0.59 to 0.95)
Drug use disorder	After pregnancy	0-4	0.51(0.41 to 0.65)	0.49(0.39 to 0.62)	0.52(0.41 to 0.65)
Drug use disorder	After pregnancy	5-9	0.81(0.65 to 1.00)	0.75(0.60 to 0.94)	0.78(0.63 to 0.98)
Drug use disorder	After pregnancy	10-14	0.75(0.61 to 0.93)	0.73(0.59 to 0.90)	0.75(0.61 to 0.93)
Drug use disorder	After pregnancy	15-19	0.67(0.55 to 0.83)	0.65(0.53 to 0.81)	0.68(0.55 to 0.84)
Drug use disorder	After pregnancy	20-24	0.79(0.64 to 0.98)	0.75(0.61 to 0.93)	0.78(0.63 to 0.97)
Drug use disorder	After pregnancy	25-29	0.68(0.55 to 0.85)	0.66(0.53 to 0.82)	0.68(0.54 to 0.84)
Drug use disorder	After pregnancy	30-34	0.95(0.76 to 1.18)	0.93(0.75 to 1.15)	0.95(0.77 to 1.19)
Drug use disorder	After pregnancy	35-39	1.00(0.81 to 1.24)	0.98(0.78 to 1.21)	0.99(0.80 to 1.23)

© 2026 Xiang N et al. *JAMA Network Open*.

Drug use disorder	After pregnancy	40-44	0.99(0.80 to 1.24)	0.99(0.80 to 1.24)	1.01(0.80 to 1.26)
Drug use disorder	After pregnancy	45-49	1.03(0.84 to 1.27)	1.02(0.83 to 1.25)	1.03(0.84 to 1.27)
Drug use disorder	After pregnancy	50-51	1.15(0.80 to 1.65)	1.18(0.81 to 1.71)	1.20(0.83 to 1.74)
ADHD	During pregnancy	0-4	1.00(0.81 to 1.22)	0.96(0.79 to 1.18)	1.00(0.82 to 1.23)
ADHD	During pregnancy	5-9	0.86(0.70 to 1.07)	0.84(0.68 to 1.03)	0.86(0.70 to 1.06)
ADHD	During pregnancy	10-14	0.90(0.74 to 1.11)	0.88(0.71 to 1.08)	0.90(0.73 to 1.10)
ADHD	During pregnancy	15-19	1.12(0.91 to 1.37)	1.09(0.89 to 1.33)	1.11(0.91 to 1.36)
ADHD	During pregnancy	20-24	1.02(0.83 to 1.24)	1.00(0.82 to 1.23)	1.02(0.84 to 1.25)
ADHD	During pregnancy	25-29	0.99(0.82 to 1.20)	0.98(0.81 to 1.19)	0.99(0.82 to 1.20)
ADHD	During pregnancy	30-34	1.07(0.87 to 1.32)	1.03(0.83 to 1.27)	1.04(0.84 to 1.29)
ADHD	During pregnancy	35-39	1.56(1.25 to 1.95)	1.57(1.25 to 1.97)	1.58(1.26 to 1.98)
ADHD	After pregnancy	0-4	0.93(0.75 to 1.14)	0.91(0.74 to 1.12)	0.95(0.77 to 1.17)
ADHD	After pregnancy	5-9	1.09(0.89 to 1.33)	1.06(0.86 to 1.29)	1.09(0.89 to 1.33)
ADHD	After pregnancy	10-14	1.01(0.83 to 1.23)	0.99(0.81 to 1.21)	1.02(0.84 to 1.25)
ADHD	After pregnancy	15-19	1.24(1.01 to 1.51)	1.21(0.99 to 1.48)	1.25(1.02 to 1.53)
ADHD	After pregnancy	20-24	1.07(0.88 to 1.31)	1.05(0.86 to 1.29)	1.08(0.88 to 1.32)
ADHD	After pregnancy	25-29	0.97(0.79 to 1.17)	0.96(0.79 to 1.17)	0.98(0.81 to 1.20)
ADHD	After pregnancy	30-34	1.11(0.90 to 1.37)	1.11(0.90 to 1.36)	1.13(0.92 to 1.39)
ADHD	After pregnancy	35-39	1.56(1.25 to 1.94)	1.55(1.25 to 1.94)	1.57(1.26 to 1.96)
ADHD	After pregnancy	40-44	0.99(0.82 to 1.19)	0.98(0.81 to 1.19)	0.99(0.82 to 1.20)
ADHD	After pregnancy	45-49	1.09(0.90 to 1.31)	1.08(0.89 to 1.30)	1.09(0.90 to 1.31)
ADHD	After pregnancy	50-51	1.32(0.98 to 1.78)	1.29(0.95 to 1.74)	1.31(0.97 to 1.77)
Bipolar disorder	During pregnancy	0-4	1.17(0.79 to 1.75)	1.11(0.74 to 1.66)	1.12(0.75 to 1.68)
Bipolar disorder	During pregnancy	5-9	0.79(0.53 to 1.18)	0.73(0.49 to 1.11)	0.76(0.50 to 1.14)
Bipolar disorder	During pregnancy	10-14	1.13(0.77 to 1.67)	1.09(0.74 to 1.60)	1.11(0.75 to 1.63)
Bipolar disorder	During pregnancy	15-19	0.88(0.60 to 1.29)	0.86(0.59 to 1.26)	0.88(0.60 to 1.30)
Bipolar disorder	During pregnancy	20-24	0.65(0.41 to 1.01)	0.66(0.42 to 1.03)	0.64(0.41 to 1.00)
Bipolar disorder	During pregnancy	25-29	0.81(0.55 to 1.19)	0.80(0.54 to 1.17)	0.79(0.54 to 1.17)
Bipolar disorder	During pregnancy	30-34	0.95(0.63 to 1.43)	0.95(0.63 to 1.42)	0.95(0.63 to 1.43)
Bipolar disorder	During pregnancy	35-39	1.08(0.71 to 1.64)	1.06(0.69 to 1.62)	1.06(0.70 to 1.63)
Bipolar disorder	After pregnancy	0-4	1.14(0.76 to 1.71)	1.11(0.74 to 1.66)	1.12(0.75 to 1.68)
Bipolar disorder	After pregnancy	5-9	1.08(0.74 to 1.58)	1.06(0.73 to 1.55)	1.09(0.75 to 1.59)
Bipolar disorder	After pregnancy	10-14	1.24(0.85 to 1.80)	1.19(0.82 to 1.74)	1.22(0.84 to 1.78)
Bipolar disorder	After pregnancy	15-19	1.07(0.74 to 1.54)	1.00(0.69 to 1.44)	1.02(0.71 to 1.48)
Bipolar disorder	After pregnancy	20-24	1.38(0.95 to 2.00)	1.39(0.95 to 2.02)	1.36(0.93 to 1.99)
Bipolar disorder	After pregnancy	25-29	1.21(0.85 to 1.71)	1.17(0.82 to 1.67)	1.18(0.83 to 1.67)
Bipolar disorder	After pregnancy	30-34	1.48(1.02 to 2.14)	1.46(1.00 to 2.12)	1.47(1.01 to 2.14)
Bipolar disorder	After pregnancy	35-39	1.75(1.21 to 2.54)	1.74(1.20 to 2.52)	1.75(1.21 to 2.54)
Bipolar disorder	After pregnancy	40-44	1.37(0.95 to 1.98)	1.38(0.95 to 1.98)	1.37(0.95 to 1.98)
Bipolar disorder	After pregnancy	45-49	1.61(1.09 to 2.38)	1.64(1.11 to 2.44)	1.64(1.10 to 2.43)
Bipolar disorder	After pregnancy	50-51	1.83(1.00 to 3.33)	1.76(0.96 to 3.23)	1.77(0.97 to 3.24)
Psychosis	During pregnancy	0-4	1.00(0.64 to 1.57)	0.93(0.60 to 1.46)	0.98(0.62 to 1.53)
Psychosis	During pregnancy	5-9	0.92(0.62 to 1.39)	0.85(0.57 to 1.29)	0.88(0.59 to 1.33)
Psychosis	During pregnancy	10-14	1.86(1.18 to 2.95)	1.68(1.06 to 2.66)	1.74(1.10 to 2.77)
Psychosis	During pregnancy	15-19	1.13(0.74 to 1.75)	1.05(0.68 to 1.63)	1.07(0.69 to 1.65)

© 2026 Xiang N et al. *JAMA Network Open*.

Psychosis	During pregnancy	20-24	1.27(0.83 to 1.94)	1.24(0.80 to 1.92)	1.27(0.82 to 1.97)
Psychosis	During pregnancy	25-29	1.07(0.70 to 1.63)	1.00(0.66 to 1.53)	1.01(0.66 to 1.54)
Psychosis	During pregnancy	30-34	1.16(0.76 to 1.77)	1.17(0.76 to 1.79)	1.18(0.77 to 1.82)
Psychosis	During pregnancy	35-39	1.12(0.72 to 1.75)	1.08(0.68 to 1.69)	1.06(0.67 to 1.67)
Psychosis	After pregnancy	0-4	1.06(0.68 to 1.64)	1.00(0.64 to 1.55)	1.05(0.67 to 1.63)
Psychosis	After pregnancy	5-9	0.95(0.64 to 1.42)	0.87(0.58 to 1.31)	0.90(0.60 to 1.36)
Psychosis	After pregnancy	10-14	1.12(0.68 to 1.86)	1.05(0.63 to 1.73)	1.09(0.66 to 1.81)
Psychosis	After pregnancy	15-19	1.11(0.72 to 1.72)	1.04(0.67 to 1.61)	1.06(0.68 to 1.65)
Psychosis	After pregnancy	20-24	0.99(0.63 to 1.56)	0.99(0.62 to 1.57)	1.02(0.64 to 1.62)
Psychosis	After pregnancy	25-29	0.89(0.57 to 1.38)	0.88(0.57 to 1.36)	0.89(0.58 to 1.39)
Psychosis	After pregnancy	30-34	1.51(1.01 to 2.27)	1.49(0.99 to 2.25)	1.52(1.01 to 2.30)
Psychosis	After pregnancy	35-39	1.34(0.88 to 2.03)	1.27(0.83 to 1.95)	1.26(0.82 to 1.93)
Psychosis	After pregnancy	40-44	1.39(0.92 to 2.11)	1.32(0.86 to 2.01)	1.32(0.86 to 2.01)
Psychosis	After pregnancy	45-49	1.49(0.96 to 2.31)	1.40(0.90 to 2.18)	1.40(0.90 to 2.18)
Psychosis	After pregnancy	50-51	1.74(0.87 to 3.50)	2.07(0.99 to 4.35)	2.12(1.01 to 4.46)

*ADHD, attention deficit hyperactivity disorder.

* The incidence rate ratio was estimated by every 5 weeks, by comparing the incidence in each interval during and after pregnancy with that before pregnancy. Model 1 was adjusted for age and calendar year at childbirth and week at follow-up. Model 2 was additionally adjusted for country of birth, region of residence and education before pregnancy, season at childbirth, and income before pregnancy. Model 3 was additionally adjusted for civil status during pregnancy, multiple gestation, number of children, and history of psychiatric disorders.

© 2026 Xiang N et al. *JAMA Network Open*.

eTable 5. Incidence Rate Ratios of 9 Type-Specific Paternal Psychiatric Disorders During and After Pregnancy, Excluding Fathers With a History of Any Other Psychiatric Disorder

Psychiatric disorders	Phase	Weeks	Incidence rate differences per 1000 person-years		
			Model 1	Model 2	Model 3
**Depression**	During pregnancy	0-4	0.86(0.70 to 1.06)	0.85(0.69 to 1.04)	0.85(0.69 to 1.05)
**Depression**	During pregnancy	5-9	0.93(0.77 to 1.12)	0.89(0.74 to 1.08)	0.90(0.74 to 1.09)
**Depression**	During pregnancy	10-14	0.82(0.68 to 0.99)	0.80(0.66 to 0.97)	0.81(0.67 to 0.98)
**Depression**	During pregnancy	15-19	0.97(0.80 to 1.17)	0.94(0.78 to 1.14)	0.94(0.78 to 1.14)
**Depression**	During pregnancy	20-24	1.05(0.86 to 1.28)	1.01(0.83 to 1.23)	1.01(0.83 to 1.24)
**Depression**	During pregnancy	25-29	0.75(0.62 to 0.91)	0.74(0.61 to 0.90)	0.74(0.61 to 0.90)
**Depression**	During pregnancy	30-34	0.79(0.65 to 0.96)	0.77(0.63 to 0.94)	0.78(0.64 to 0.95)
**Depression**	During pregnancy	35-39	0.69(0.56 to 0.84)	0.70(0.57 to 0.86)	0.70(0.57 to 0.85)
**Depression**	After pregnancy	0-4	0.88(0.72 to 1.08)	0.86(0.70 to 1.06)	0.87(0.71 to 1.07)
**Depression**	After pregnancy	5-9	0.86(0.71 to 1.05)	0.84(0.69 to 1.02)	0.84(0.69 to 1.02)
**Depression**	After pregnancy	10-14	0.91(0.76 to 1.10)	0.88(0.73 to 1.05)	0.89(0.74 to 1.07)
**Depression**	After pregnancy	15-19	1.09(0.91 to 1.31)	1.08(0.90 to 1.29)	1.08(0.90 to 1.29)
**Depression**	After pregnancy	20-24	1.17(0.96 to 1.42)	1.15(0.95 to 1.40)	1.16(0.95 to 1.41)
**Depression**	After pregnancy	25-29	1.08(0.90 to 1.29)	1.06(0.88 to 1.27)	1.06(0.89 to 1.27)
**Depression**	After pregnancy	30-34	1.15(0.96 to 1.38)	1.14(0.95 to 1.36)	1.15(0.96 to 1.38)
**Depression**	After pregnancy	35-39	1.16(0.98 to 1.38)	1.15(0.97 to 1.37)	1.15(0.97 to 1.37)
**Depression**	After pregnancy	40-44	1.15(0.96 to 1.37)	1.12(0.94 to 1.34)	1.12(0.94 to 1.34)
**Depression**	After pregnancy	45-49	1.45(1.20 to 1.75)	1.45(1.20 to 1.75)	1.45(1.20 to 1.76)
**Depression**	After pregnancy	50-51	1.37(1.02 to 1.85)	1.33(0.98 to 1.80)	1.33(0.98 to 1.80)
**Anxiety**	During pregnancy	0-4	1.05(0.88 to 1.26)	1.01(0.84 to 1.21)	1.02(0.85 to 1.22)
**Anxiety**	During pregnancy	5-9	0.89(0.74 to 1.07)	0.87(0.72 to 1.05)	0.88(0.73 to 1.05)
**Anxiety**	During pregnancy	10-14	0.88(0.74 to 1.06)	0.85(0.71 to 1.02)	0.85(0.71 to 1.02)
**Anxiety**	During pregnancy	15-19	0.89(0.74 to 1.06)	0.87(0.72 to 1.05)	0.87(0.72 to 1.05)
**Anxiety**	During pregnancy	20-24	1.25(1.04 to 1.51)	1.22(1.01 to 1.47)	1.22(1.01 to 1.47)
**Anxiety**	During pregnancy	25-29	1.10(0.91 to 1.32)	1.07(0.89 to 1.29)	1.07(0.89 to 1.29)
**Anxiety**	During pregnancy	30-34	1.09(0.92 to 1.31)	1.07(0.90 to 1.28)	1.08(0.90 to 1.29)
**Anxiety**	During pregnancy	35-39	0.97(0.81 to 1.16)	0.96(0.80 to 1.15)	0.96(0.80 to 1.15)
**Anxiety**	After pregnancy	0-4	0.99(0.82 to 1.18)	0.95(0.79 to 1.14)	0.96(0.80 to 1.15)
**Anxiety**	After pregnancy	5-9	0.94(0.78 to 1.13)	0.93(0.77 to 1.11)	0.93(0.78 to 1.12)
**Anxiety**	After pregnancy	10-14	0.93(0.78 to 1.11)	0.89(0.75 to 1.06)	0.89(0.74 to 1.06)
**Anxiety**	After pregnancy	15-19	1.11(0.93 to 1.33)	1.07(0.90 to 1.28)	1.08(0.90 to 1.28)
**Anxiety**	After pregnancy	20-24	1.35(1.12 to 1.62)	1.31(1.09 to 1.58)	1.31(1.09 to 1.58)
**Anxiety**	After pregnancy	25-29	1.35(1.13 to 1.61)	1.31(1.09 to 1.56)	1.31(1.10 to 1.57)
**Anxiety**	After pregnancy	30-34	1.08(0.91 to 1.30)	1.08(0.90 to 1.30)	1.09(0.91 to 1.30)
**Anxiety**	After pregnancy	35-39	1.12(0.95 to 1.32)	1.11(0.94 to 1.31)	1.12(0.94 to 1.32)
**Anxiety**	After pregnancy	40-44	1.01(0.85 to 1.20)	1.01(0.85 to 1.20)	1.01(0.85 to 1.20)
**Anxiety**	After pregnancy	45-49	1.17(0.98 to 1.39)	1.15(0.97 to 1.37)	1.16(0.97 to 1.38)
**Anxiety**	After pregnancy	50-51	0.92(0.71 to 1.19)	0.89(0.68 to 1.16)	0.89(0.69 to 1.17)
**Stress-related disorder**	During pregnancy	0-4	0.89(0.72 to 1.11)	0.86(0.69 to 1.06)	0.86(0.69 to 1.06)
**Stress-related disorder**	During pregnancy	5-9	0.81(0.65 to 1.01)	0.76(0.61 to 0.94)	0.76(0.61 to 0.95)

© 2026 Xiang N et al. *JAMA Network Open*.

**Stress-related disorder**	During pregnancy	10-14	0.92(0.75 to 1.15)	0.89(0.72 to 1.11)	0.89(0.72 to 1.11)
**Stress-related disorder**	During pregnancy	15-19	0.98(0.79 to 1.21)	0.94(0.76 to 1.17)	0.94(0.76 to 1.17)
**Stress-related disorder**	During pregnancy	20-24	0.91(0.74 to 1.11)	0.89(0.72 to 1.09)	0.89(0.72 to 1.09)
**Stress-related disorder**	During pregnancy	25-29	0.81(0.65 to 1.01)	0.78(0.62 to 0.97)	0.78(0.62 to 0.97)
**Stress-related disorder**	During pregnancy	30-34	0.78(0.63 to 0.97)	0.75(0.60 to 0.94)	0.75(0.60 to 0.94)
**Stress-related disorder**	During pregnancy	35-39	0.82(0.66 to 1.03)	0.79(0.63 to 0.99)	0.79(0.63 to 0.99)
**Stress-related disorder**	After pregnancy	0-4	1.13(0.92 to 1.38)	1.07(0.87 to 1.31)	1.07(0.87 to 1.32)
**Stress-related disorder**	After pregnancy	5-9	1.09(0.89 to 1.34)	1.07(0.87 to 1.31)	1.07(0.87 to 1.31)
**Stress-related disorder**	After pregnancy	10-14	1.17(0.95 to 1.43)	1.10(0.90 to 1.36)	1.10(0.90 to 1.36)
**Stress-related disorder**	After pregnancy	15-19	1.09(0.88 to 1.34)	1.05(0.85 to 1.29)	1.05(0.85 to 1.29)
**Stress-related disorder**	After pregnancy	20-24	0.92(0.75 to 1.13)	0.85(0.69 to 1.04)	0.85(0.69 to 1.04)
**Stress-related disorder**	After pregnancy	25-29	1.18(0.97 to 1.44)	1.15(0.94 to 1.40)	1.15(0.94 to 1.41)
**Stress-related disorder**	After pregnancy	30-34	1.36(1.12 to 1.65)	1.35(1.11 to 1.64)	1.35(1.11 to 1.64)
**Stress-related disorder**	After pregnancy	35-39	1.38(1.14 to 1.67)	1.33(1.10 to 1.62)	1.34(1.10 to 1.62)
**Stress-related disorder**	After pregnancy	40-44	1.06(0.87 to 1.30)	1.05(0.86 to 1.29)	1.05(0.86 to 1.28)
**Stress-related disorder**	After pregnancy	45-49	1.52(1.24 to 1.86)	1.49(1.21 to 1.83)	1.49(1.21 to 1.83)
**Stress-related disorder**	After pregnancy	50-51	1.40(1.02 to 1.93)	1.41(1.01 to 1.96)	1.39(1.00 to 1.94)
**Alcohol use disorder**	During pregnancy	0-4	0.63(0.49 to 0.80)	0.62(0.48 to 0.79)	0.63(0.49 to 0.80)
**Alcohol use disorder**	During pregnancy	5-9	0.71(0.56 to 0.90)	0.70(0.55 to 0.89)	0.71(0.55 to 0.90)
**Alcohol use disorder**	During pregnancy	10-14	0.89(0.70 to 1.14)	0.88(0.69 to 1.13)	0.89(0.70 to 1.15)
**Alcohol use disorder**	During pregnancy	15-19	0.69(0.55 to 0.87)	0.68(0.54 to 0.86)	0.69(0.54 to 0.87)
**Alcohol use disorder**	During pregnancy	20-24	0.62(0.49 to 0.78)	0.62(0.49 to 0.78)	0.62(0.49 to 0.79)
**Alcohol use disorder**	During pregnancy	25-29	0.77(0.59 to 1.00)	0.74(0.56 to 0.97)	0.74(0.56 to 0.97)
**Alcohol use disorder**	During pregnancy	30-34	0.67(0.52 to 0.86)	0.68(0.52 to 0.88)	0.68(0.53 to 0.88)
**Alcohol use disorder**	During pregnancy	35-39	0.56(0.43 to 0.73)	0.54(0.41 to 0.71)	0.54(0.41 to 0.71)
**Alcohol use disorder**	After pregnancy	0-4	0.52(0.40 to 0.67)	0.51(0.39 to 0.66)	0.52(0.40 to 0.67)
**Alcohol use disorder**	After pregnancy	5-9	0.60(0.47 to 0.78)	0.58(0.45 to 0.75)	0.59(0.45 to 0.76)
**Alcohol use disorder**	After pregnancy	10-14	0.91(0.71 to 1.17)	0.90(0.70 to 1.15)	0.91(0.71 to 1.17)
**Alcohol use disorder**	After pregnancy	15-19	0.71(0.56 to 0.89)	0.69(0.54 to 0.87)	0.69(0.55 to 0.88)
**Alcohol use disorder**	After pregnancy	20-24	0.55(0.43 to 0.71)	0.54(0.42 to 0.70)	0.55(0.43 to 0.70)
**Alcohol use disorder**	After pregnancy	25-29	1.05(0.82 to 1.35)	1.01(0.78 to 1.30)	1.01(0.78 to 1.30)
**Alcohol use disorder**	After pregnancy	30-34	0.87(0.69 to 1.11)	0.87(0.68 to 1.11)	0.87(0.69 to 1.11)
**Alcohol use disorder**	After pregnancy	35-39	0.87(0.69 to 1.09)	0.85(0.67 to 1.07)	0.85(0.67 to 1.07)
**Alcohol use disorder**	After pregnancy	40-44	0.90(0.71 to 1.13)	0.88(0.70 to 1.11)	0.89(0.70 to 1.12)
**Alcohol use disorder**	After pregnancy	45-49	1.05(0.83 to 1.34)	1.03(0.81 to 1.31)	1.04(0.82 to 1.32)
**Alcohol use disorder**	After pregnancy	50-51	1.01(0.71 to 1.44)	1.02(0.71 to 1.46)	1.02(0.72 to 1.46)
**Tobacco use disorder**	During pregnancy	0-4	1.85(0.92 to 3.73)	1.70(0.84 to 3.44)	1.69(0.83 to 3.42)
**Tobacco use disorder**	During pregnancy	5-9	1.53(0.86 to 2.74)	1.44(0.80 to 2.59)	1.46(0.81 to 2.62)
**Tobacco use disorder**	During pregnancy	10-14	1.11(0.66 to 1.87)	1.04(0.62 to 1.77)	1.06(0.63 to 1.80)
**Tobacco use disorder**	During pregnancy	15-19	0.74(0.45 to 1.23)	0.71(0.42 to 1.18)	0.71(0.42 to 1.18)
**Tobacco use disorder**	During pregnancy	20-24	1.07(0.63 to 1.79)	1.04(0.62 to 1.75)	1.04(0.62 to 1.75)
**Tobacco use disorder**	During pregnancy	25-29	1.13(0.63 to 2.05)	0.98(0.53 to 1.80)	0.99(0.54 to 1.81)
**Tobacco use disorder**	During pregnancy	30-34	1.06(0.64 to 1.74)	1.02(0.62 to 1.68)	1.03(0.62 to 1.69)
**Tobacco use disorder**	During pregnancy	35-39	0.78(0.45 to 1.35)	0.70(0.40 to 1.23)	0.71(0.40 to 1.24)
**Tobacco use disorder**	After pregnancy	0-4	1.62(0.79 to 3.31)	1.57(0.77 to 3.20)	1.56(0.76 to 3.18)

© 2026 Xiang N et al. *JAMA Network Open*.

**Tobacco use disorder**	After pregnancy	5-9	1.29(0.71 to 2.35)	1.14(0.62 to 2.10)	1.15(0.62 to 2.12)
**Tobacco use disorder**	After pregnancy	10-14	0.94(0.55 to 1.61)	0.86(0.50 to 1.49)	0.87(0.50 to 1.51)
**Tobacco use disorder**	After pregnancy	15-19	0.89(0.55 to 1.45)	0.84(0.51 to 1.37)	0.84(0.52 to 1.38)
**Tobacco use disorder**	After pregnancy	20-24	1.27(0.77 to 2.10)	1.18(0.71 to 1.97)	1.19(0.71 to 1.97)
**Tobacco use disorder**	After pregnancy	25-29	1.28(0.72 to 2.28)	1.18(0.65 to 2.12)	1.19(0.66 to 2.14)
**Tobacco use disorder**	After pregnancy	30-34	1.20(0.74 to 1.95)	1.14(0.70 to 1.86)	1.15(0.70 to 1.88)
**Tobacco use disorder**	After pregnancy	35-39	1.07(0.66 to 1.75)	1.07(0.65 to 1.75)	1.09(0.66 to 1.78)
**Tobacco use disorder**	After pregnancy	40-44	1.32(0.84 to 2.07)	1.29(0.82 to 2.03)	1.30(0.83 to 2.05)
**Tobacco use disorder**	After pregnancy	45-49	1.43(0.87 to 2.35)	1.41(0.85 to 2.34)	1.41(0.85 to 2.35)
**Drug use disorder**	During pregnancy	0-4	0.79(0.59 to 1.05)	0.74(0.56 to 0.99)	0.78(0.58 to 1.04)
**Drug use disorder**	During pregnancy	5-9	0.74(0.54 to 1.00)	0.69(0.51 to 0.95)	0.71(0.52 to 0.97)
**Drug use disorder**	During pregnancy	10-14	0.74(0.56 to 0.98)	0.71(0.54 to 0.94)	0.73(0.55 to 0.97)
**Drug use disorder**	During pregnancy	15-19	0.72(0.55 to 0.96)	0.70(0.53 to 0.92)	0.71(0.54 to 0.94)
**Drug use disorder**	During pregnancy	20-24	0.95(0.71 to 1.27)	0.91(0.68 to 1.21)	0.92(0.69 to 1.24)
**Drug use disorder**	During pregnancy	25-29	0.86(0.65 to 1.13)	0.81(0.61 to 1.07)	0.82(0.62 to 1.09)
**Drug use disorder**	During pregnancy	30-34	0.87(0.64 to 1.18)	0.85(0.63 to 1.15)	0.87(0.64 to 1.17)
**Drug use disorder**	During pregnancy	35-39	0.81(0.59 to 1.11)	0.79(0.58 to 1.09)	0.81(0.59 to 1.11)
**Drug use disorder**	After pregnancy	0-4	0.56(0.41 to 0.76)	0.54(0.39 to 0.74)	0.56(0.41 to 0.77)
**Drug use disorder**	After pregnancy	5-9	1.01(0.76 to 1.35)	0.92(0.68 to 1.23)	0.95(0.71 to 1.27)
**Drug use disorder**	After pregnancy	10-14	0.81(0.61 to 1.06)	0.78(0.59 to 1.03)	0.80(0.61 to 1.05)
**Drug use disorder**	After pregnancy	15-19	0.76(0.58 to 1.01)	0.73(0.55 to 0.96)	0.75(0.57 to 0.99)
**Drug use disorder**	After pregnancy	20-24	0.91(0.68 to 1.22)	0.84(0.63 to 1.13)	0.86(0.64 to 1.16)
**Drug use disorder**	After pregnancy	25-29	0.77(0.58 to 1.02)	0.74(0.55 to 0.99)	0.75(0.56 to 1.00)
**Drug use disorder**	After pregnancy	30-34	1.09(0.82 to 1.45)	1.06(0.80 to 1.42)	1.08(0.81 to 1.45)
**Drug use disorder**	After pregnancy	35-39	1.00(0.75 to 1.33)	0.96(0.71 to 1.28)	0.97(0.72 to 1.29)
**Drug use disorder**	After pregnancy	40-44	1.01(0.76 to 1.34)	1.01(0.76 to 1.35)	1.02(0.76 to 1.36)
**Drug use disorder**	After pregnancy	45-49	1.02(0.79 to 1.32)	1.00(0.77 to 1.29)	1.01(0.78 to 1.31)
**Drug use disorder**	After pregnancy	50-51	0.99(0.63 to 1.54)	1.02(0.64 to 1.62)	1.03(0.65 to 1.63)
**ADHD**	During pregnancy	0-4	1.48(1.06 to 2.07)	1.44(1.03 to 2.02)	1.47(1.05 to 2.06)
**ADHD**	During pregnancy	5-9	0.90(0.65 to 1.26)	0.88(0.63 to 1.22)	0.88(0.63 to 1.22)
**ADHD**	During pregnancy	10-14	1.12(0.82 to 1.54)	1.08(0.78 to 1.49)	1.10(0.80 to 1.51)
**ADHD**	During pregnancy	15-19	1.12(0.83 to 1.53)	1.08(0.79 to 1.47)	1.08(0.79 to 1.48)
**ADHD**	During pregnancy	20-24	1.37(0.99 to 1.90)	1.36(0.98 to 1.89)	1.38(0.99 to 1.92)
**ADHD**	During pregnancy	25-29	0.88(0.65 to 1.20)	0.87(0.64 to 1.19)	0.87(0.64 to 1.18)
**ADHD**	During pregnancy	30-34	1.06(0.77 to 1.45)	1.01(0.74 to 1.39)	1.02(0.74 to 1.40)
**ADHD**	During pregnancy	35-39	1.57(1.14 to 2.17)	1.57(1.13 to 2.17)	1.57(1.13 to 2.18)
**ADHD**	After pregnancy	0-4	1.40(1.00 to 1.97)	1.40(0.99 to 1.96)	1.42(1.01 to 2.00)
**ADHD**	After pregnancy	5-9	1.45(1.07 to 1.95)	1.39(1.03 to 1.89)	1.39(1.03 to 1.89)
**ADHD**	After pregnancy	10-14	1.28(0.94 to 1.74)	1.27(0.93 to 1.73)	1.29(0.95 to 1.76)
**ADHD**	After pregnancy	15-19	1.23(0.91 to 1.67)	1.19(0.88 to 1.62)	1.20(0.88 to 1.63)
**ADHD**	After pregnancy	20-24	1.50(1.09 to 2.07)	1.46(1.06 to 2.02)	1.48(1.07 to 2.05)
**ADHD**	After pregnancy	25-29	1.12(0.84 to 1.50)	1.14(0.85 to 1.53)	1.14(0.85 to 1.52)
**ADHD**	After pregnancy	30-34	1.35(1.00 to 1.82)	1.36(1.01 to 1.83)	1.37(1.02 to 1.85)
**ADHD**	After pregnancy	35-39	1.80(1.32 to 2.46)	1.78(1.30 to 2.42)	1.78(1.31 to 2.44)
**ADHD**	After pregnancy	40-44	1.28(0.96 to 1.69)	1.27(0.95 to 1.68)	1.28(0.96 to 1.69)

© 2026 Xiang N et al. *JAMA Network Open*.

**ADHD**	After pregnancy	45-49	1.23(0.93 to 1.61)	1.20(0.91 to 1.58)	1.20(0.91 to 1.58)
**ADHD**	After pregnancy	50-51	1.22(0.80 to 1.86)	1.15(0.75 to 1.78)	1.17(0.76 to 1.80)
**Bipolar disorder**	During pregnancy	0-4	1.00(0.47 to 2.11)	0.95(0.45 to 2.01)	0.95(0.45 to 2.01)
**Bipolar disorder**	During pregnancy	5-9	1.07(0.49 to 2.33)	1.04(0.48 to 2.26)	1.05(0.48 to 2.30)
**Bipolar disorder**	During pregnancy	10-14	1.40(0.73 to 2.68)	1.30(0.67 to 2.51)	1.29(0.67 to 2.50)
**Bipolar disorder**	During pregnancy	15-19	0.63(0.35 to 1.16)	0.63(0.34 to 1.14)	0.63(0.34 to 1.15)
**Bipolar disorder**	During pregnancy	20-24	0.70(0.32 to 1.52)	0.76(0.35 to 1.66)	0.74(0.34 to 1.63)
**Bipolar disorder**	During pregnancy	25-29	0.93(0.48 to 1.78)	0.88(0.45 to 1.71)	0.89(0.46 to 1.72)
**Bipolar disorder**	During pregnancy	30-34	0.97(0.55 to 1.72)	0.97(0.55 to 1.72)	0.97(0.55 to 1.72)
**Bipolar disorder**	During pregnancy	35-39	1.13(0.60 to 2.14)	1.09(0.57 to 2.08)	1.08(0.56 to 2.06)
**Bipolar disorder**	After pregnancy	0-4	1.85(0.94 to 3.61)	1.77(0.91 to 3.47)	1.78(0.91 to 3.48)
**Bipolar disorder**	After pregnancy	5-9	1.48(0.71 to 3.07)	1.45(0.70 to 3.01)	1.47(0.71 to 3.06)
**Bipolar disorder**	After pregnancy	10-14	1.50(0.79 to 2.86)	1.41(0.74 to 2.70)	1.41(0.73 to 2.69)
**Bipolar disorder**	After pregnancy	15-19	0.93(0.54 to 1.61)	0.83(0.47 to 1.45)	0.83(0.48 to 1.46)
**Bipolar disorder**	After pregnancy	20-24	2.06(1.11 to 3.83)	2.17(1.15 to 4.12)	2.13(1.13 to 4.05)
**Bipolar disorder**	After pregnancy	25-29	1.73(0.97 to 3.09)	1.69(0.94 to 3.02)	1.70(0.95 to 3.04)
**Bipolar disorder**	After pregnancy	30-34	1.26(0.73 to 2.17)	1.22(0.71 to 2.11)	1.23(0.71 to 2.13)
**Bipolar disorder**	After pregnancy	35-39	1.95(1.12 to 3.41)	1.92(1.10 to 3.37)	1.92(1.10 to 3.36)
**Bipolar disorder**	After pregnancy	40-44	1.68(0.91 to 3.10)	1.69(0.92 to 3.11)	1.68(0.91 to 3.09)
**Bipolar disorder**	After pregnancy	45-49	1.76(0.97 to 3.17)	1.87(1.02 to 3.41)	1.86(1.02 to 3.40)
**Bipolar disorder**	After pregnancy	50-51	1.84(0.74 to 4.56)	1.71(0.68 to 4.29)	1.68(0.67 to 4.21)
**Psychosis**	During pregnancy	0-4	1.15(0.55 to 2.39)	1.03(0.49 to 2.17)	1.06(0.51 to 2.23)
**Psychosis**	During pregnancy	5-9	1.38(0.75 to 2.56)	1.24(0.66 to 2.31)	1.27(0.68 to 2.37)
**Psychosis**	During pregnancy	10-14	2.31(1.12 to 4.73)	2.02(0.98 to 4.17)	2.06(0.99 to 4.25)
**Psychosis**	During pregnancy	15-19	1.55(0.77 to 3.12)	1.39(0.68 to 2.83)	1.40(0.69 to 2.84)
**Psychosis**	During pregnancy	20-24	1.60(0.87 to 2.92)	1.51(0.81 to 2.83)	1.54(0.82 to 2.88)
**Psychosis**	During pregnancy	25-29	1.20(0.60 to 2.37)	1.13(0.57 to 2.25)	1.12(0.56 to 2.24)
**Psychosis**	During pregnancy	30-34	1.33(0.66 to 2.67)	1.43(0.70 to 2.92)	1.43(0.70 to 2.94)
**Psychosis**	During pregnancy	35-39	0.93(0.50 to 1.73)	0.86(0.45 to 1.64)	0.84(0.44 to 1.60)
**Psychosis**	After pregnancy	0-4	1.13(0.55 to 2.35)	1.09(0.53 to 2.27)	1.12(0.54 to 2.33)
**Psychosis**	After pregnancy	5-9	1.37(0.74 to 2.53)	1.19(0.63 to 2.23)	1.22(0.65 to 2.29)
**Psychosis**	After pregnancy	10-14	1.34(0.61 to 2.93)	1.20(0.55 to 2.65)	1.23(0.56 to 2.70)
**Psychosis**	After pregnancy	15-19	1.96(1.00 to 3.84)	1.81(0.92 to 3.57)	1.82(0.92 to 3.59)
**Psychosis**	After pregnancy	20-24	1.19(0.63 to 2.26)	1.15(0.59 to 2.24)	1.18(0.61 to 2.28)
**Psychosis**	After pregnancy	25-29	1.03(0.51 to 2.09)	1.03(0.51 to 2.10)	1.03(0.51 to 2.09)
**Psychosis**	After pregnancy	30-34	2.10(1.10 to 4.01)	2.13(1.09 to 4.18)	2.15(1.10 to 4.21)
**Psychosis**	After pregnancy	35-39	1.38(0.80 to 2.40)	1.28(0.72 to 2.27)	1.26(0.71 to 2.23)
**Psychosis**	After pregnancy	40-44	1.71(0.95 to 3.09)	1.56(0.85 to 2.85)	1.57(0.86 to 2.86)
**Psychosis**	After pregnancy	45-49	2.15(1.12 to 4.13)	1.93(0.99 to 3.76)	1.92(0.99 to 3.74)

*ADHD, attention deficit hyperactivity disorder.

* The incidence rate ratio was estimated by every 5 weeks, by comparing the incidence in each interval during and after pregnancy with that before pregnancy. Model 1 was adjusted for age and calendar year at childbirth and week at follow-up. Model 2 was additionally adjusted for country of birth, region of residence and education before pregnancy, season at childbirth, and income before pregnancy. Model 3 was additionally adjusted for civil status during pregnancy, multiple gestation, number of children, and history of

© 2026 Xiang N et al. *JAMA Network Open*.

psychiatric disorders.

© 2026 Xiang N et al. *JAMA Network Open*.

eTable 6. Incidence Rate Ratios of Any Paternal Psychiatric Disorder and 9 Type-Specific Disorders During and After Pregnancy, Restricted to Stockholm Where Both Primary Care and Specialist Care Data Were Available

Psychiatric disorders	Phase	Weeks	Incidence rate differences per 1000 person-years		
			Model 1	Model 2	Model 3
Any psychiatric disorder	During pregnancy	0-4	0.94(0.84 to 1.05)	0.93(0.83 to 1.04)	0.93(0.83 to 1.04)
Any psychiatric disorder	During pregnancy	5-9	0.84(0.76 to 0.94)	0.83(0.74 to 0.92)	0.83(0.74 to 0.93)
Any psychiatric disorder	During pregnancy	10-14	0.88(0.79 to 0.99)	0.87(0.78 to 0.97)	0.87(0.78 to 0.97)
Any psychiatric disorder	During pregnancy	15-19	0.85(0.77 to 0.95)	0.83(0.75 to 0.93)	0.84(0.75 to 0.93)
Any psychiatric disorder	During pregnancy	20-24	0.99(0.89 to 1.10)	0.98(0.87 to 1.09)	0.98(0.88 to 1.09)
Any psychiatric disorder	During pregnancy	25-29	0.94(0.85 to 1.05)	0.93(0.83 to 1.04)	0.94(0.84 to 1.04)
Any psychiatric disorder	During pregnancy	30-34	0.96(0.86 to 1.07)	0.94(0.85 to 1.05)	0.95(0.85 to 1.05)
Any psychiatric disorder	During pregnancy	35-39	0.82(0.73 to 0.92)	0.82(0.73 to 0.92)	0.82(0.73 to 0.93)
Any psychiatric disorder	After pregnancy	0-4	0.86(0.77 to 0.96)	0.85(0.76 to 0.95)	0.86(0.76 to 0.96)
Any psychiatric disorder	After pregnancy	5-9	0.88(0.79 to 0.98)	0.87(0.78 to 0.97)	0.87(0.78 to 0.97)
Any psychiatric disorder	After pregnancy	10-14	0.94(0.84 to 1.04)	0.92(0.83 to 1.03)	0.93(0.83 to 1.03)
Any psychiatric disorder	After pregnancy	15-19	0.97(0.88 to 1.08)	0.95(0.85 to 1.06)	0.95(0.86 to 1.06)
Any psychiatric disorder	After pregnancy	20-24	1.02(0.92 to 1.14)	0.99(0.89 to 1.11)	1.00(0.89 to 1.11)
Any psychiatric disorder	After pregnancy	25-29	0.97(0.87 to 1.08)	0.96(0.86 to 1.07)	0.96(0.86 to 1.07)
Any psychiatric disorder	After pregnancy	30-34	0.93(0.84 to 1.04)	0.92(0.83 to 1.03)	0.92(0.83 to 1.03)
Any psychiatric disorder	After pregnancy	35-39	0.92(0.83 to 1.03)	0.91(0.82 to 1.02)	0.91(0.82 to 1.02)
Any psychiatric disorder	After pregnancy	40-44	1.01(0.91 to 1.13)	1.00(0.90 to 1.12)	1.01(0.90 to 1.12)
Any psychiatric disorder	After pregnancy	45-49	0.97(0.87 to 1.09)	0.96(0.86 to 1.08)	0.97(0.86 to 1.08)
Any psychiatric disorder	After pregnancy	50-51	0.98(0.83 to 1.17)	0.96(0.81 to 1.15)	0.96(0.81 to 1.15)
Depression	During pregnancy	0-4	0.87(0.73 to 1.05)	0.85(0.71 to 1.02)	0.86(0.71 to 1.03)
Depression	During pregnancy	5-9	0.92(0.77 to 1.10)	0.91(0.75 to 1.09)	0.91(0.76 to 1.10)
Depression	During pregnancy	10-14	0.78(0.65 to 0.94)	0.76(0.63 to 0.91)	0.77(0.64 to 0.92)
Depression	During pregnancy	15-19	0.75(0.62 to 0.90)	0.74(0.62 to 0.89)	0.74(0.62 to 0.89)
Depression	During pregnancy	20-24	1.00(0.83 to 1.19)	0.99(0.83 to 1.19)	1.00(0.83 to 1.19)
Depression	During pregnancy	25-29	0.92(0.76 to 1.11)	0.90(0.74 to 1.09)	0.90(0.75 to 1.09)
Depression	During pregnancy	30-34	0.84(0.70 to 1.02)	0.83(0.69 to 1.00)	0.83(0.69 to 1.00)
Depression	During pregnancy	35-39	0.74(0.61 to 0.90)	0.75(0.62 to 0.91)	0.75(0.62 to 0.91)
Depression	After pregnancy	0-4	0.70(0.58 to 0.85)	0.69(0.57 to 0.84)	0.70(0.57 to 0.84)
Depression	After pregnancy	5-9	0.84(0.70 to 1.01)	0.83(0.68 to 1.00)	0.83(0.69 to 1.01)
Depression	After pregnancy	10-14	0.90(0.75 to 1.07)	0.88(0.74 to 1.05)	0.89(0.75 to 1.07)
Depression	After pregnancy	15-19	1.04(0.88 to 1.23)	1.03(0.87 to 1.22)	1.04(0.87 to 1.23)
Depression	After pregnancy	20-24	1.02(0.85 to 1.22)	1.01(0.84 to 1.21)	1.01(0.85 to 1.21)
Depression	After pregnancy	25-29	1.11(0.93 to 1.33)	1.09(0.91 to 1.31)	1.09(0.91 to 1.31)
Depression	After pregnancy	30-34	1.11(0.93 to 1.32)	1.11(0.93 to 1.32)	1.11(0.93 to 1.33)
Depression	After pregnancy	35-39	0.98(0.82 to 1.17)	0.98(0.82 to 1.17)	0.99(0.83 to 1.18)
Depression	After pregnancy	40-44	1.26(1.06 to 1.50)	1.24(1.04 to 1.48)	1.25(1.05 to 1.49)
Depression	After pregnancy	45-49	1.18(0.99 to 1.42)	1.19(0.99 to 1.43)	1.20(1.00 to 1.44)
Depression	After pregnancy	50-51	1.26(0.94 to 1.70)	1.28(0.95 to 1.73)	1.29(0.95 to 1.73)
Anxiety	During pregnancy	0-4	1.02(0.87 to 1.19)	1.00(0.86 to 1.18)	1.02(0.87 to 1.19)

© 2026 Xiang N et al. *JAMA Network Open*.

Anxiety	During pregnancy	5-9	0.87(0.74 to 1.02)	0.84(0.72 to 0.99)	0.85(0.72 to 1.00)
Anxiety	During pregnancy	10-14	0.93(0.79 to 1.09)	0.92(0.78 to 1.09)	0.93(0.79 to 1.09)
Anxiety	During pregnancy	15-19	0.91(0.77 to 1.07)	0.88(0.75 to 1.04)	0.89(0.75 to 1.04)
Anxiety	During pregnancy	20-24	1.08(0.92 to 1.27)	1.06(0.90 to 1.25)	1.07(0.91 to 1.25)
Anxiety	During pregnancy	25-29	0.95(0.82 to 1.11)	0.94(0.81 to 1.10)	0.95(0.82 to 1.11)
Anxiety	During pregnancy	30-34	1.03(0.88 to 1.20)	1.02(0.87 to 1.19)	1.03(0.88 to 1.20)
Anxiety	During pregnancy	35-39	0.85(0.73 to 1.00)	0.85(0.72 to 1.00)	0.85(0.73 to 1.00)
Anxiety	After pregnancy	0-4	0.88(0.75 to 1.04)	0.88(0.75 to 1.04)	0.89(0.76 to 1.05)
Anxiety	After pregnancy	5-9	0.97(0.83 to 1.14)	0.96(0.82 to 1.12)	0.97(0.82 to 1.13)
Anxiety	After pregnancy	10-14	1.05(0.90 to 1.23)	1.04(0.89 to 1.22)	1.05(0.89 to 1.23)
Anxiety	After pregnancy	15-19	1.03(0.88 to 1.21)	1.02(0.87 to 1.19)	1.03(0.88 to 1.20)
Anxiety	After pregnancy	20-24	1.12(0.96 to 1.32)	1.10(0.94 to 1.30)	1.11(0.95 to 1.31)
Anxiety	After pregnancy	25-29	0.91(0.78 to 1.06)	0.89(0.76 to 1.04)	0.90(0.77 to 1.05)
Anxiety	After pregnancy	30-34	0.91(0.77 to 1.07)	0.89(0.76 to 1.05)	0.90(0.77 to 1.06)
Anxiety	After pregnancy	35-39	0.79(0.67 to 0.93)	0.78(0.66 to 0.91)	0.78(0.67 to 0.92)
Anxiety	After pregnancy	40-44	0.87(0.74 to 1.02)	0.87(0.74 to 1.02)	0.87(0.74 to 1.03)
Anxiety	After pregnancy	45-49	0.87(0.73 to 1.02)	0.86(0.73 to 1.01)	0.86(0.73 to 1.02)
Anxiety	After pregnancy	50-51	0.93(0.73 to 1.19)	0.94(0.73 to 1.20)	0.94(0.73 to 1.20)
Stress-related disorder	During pregnancy	0-4	0.98(0.83 to 1.16)	0.98(0.83 to 1.16)	0.98(0.83 to 1.17)
Stress-related disorder	During pregnancy	5-9	0.94(0.79 to 1.11)	0.92(0.78 to 1.10)	0.92(0.78 to 1.10)
Stress-related disorder	During pregnancy	10-14	0.95(0.80 to 1.13)	0.94(0.79 to 1.12)	0.95(0.79 to 1.12)
Stress-related disorder	During pregnancy	15-19	1.01(0.85 to 1.19)	1.00(0.85 to 1.18)	1.00(0.85 to 1.18)
Stress-related disorder	During pregnancy	20-24	1.06(0.89 to 1.25)	1.07(0.90 to 1.26)	1.07(0.91 to 1.27)
Stress-related disorder	During pregnancy	25-29	1.10(0.93 to 1.31)	1.10(0.93 to 1.30)	1.10(0.93 to 1.31)
Stress-related disorder	During pregnancy	30-34	0.99(0.84 to 1.17)	0.98(0.84 to 1.16)	0.98(0.83 to 1.16)
Stress-related disorder	During pregnancy	35-39	0.95(0.80 to 1.13)	0.95(0.80 to 1.13)	0.96(0.80 to 1.14)
Stress-related disorder	After pregnancy	0-4	1.12(0.95 to 1.32)	1.11(0.94 to 1.31)	1.12(0.95 to 1.32)
Stress-related disorder	After pregnancy	5-9	1.29(1.10 to 1.52)	1.29(1.10 to 1.52)	1.29(1.10 to 1.52)
Stress-related disorder	After pregnancy	10-14	1.14(0.96 to 1.34)	1.13(0.95 to 1.33)	1.14(0.96 to 1.34)
Stress-related disorder	After pregnancy	15-19	1.17(1.00 to 1.38)	1.16(0.99 to 1.36)	1.16(0.99 to 1.37)
Stress-related disorder	After pregnancy	20-24	1.31(1.11 to 1.54)	1.29(1.10 to 1.52)	1.30(1.10 to 1.53)
Stress-related disorder	After pregnancy	25-29	1.30(1.10 to 1.53)	1.29(1.10 to 1.52)	1.30(1.10 to 1.53)
Stress-related disorder	After pregnancy	30-34	1.06(0.90 to 1.24)	1.05(0.90 to 1.24)	1.05(0.90 to 1.24)
Stress-related disorder	After pregnancy	35-39	1.20(1.03 to 1.41)	1.20(1.02 to 1.40)	1.20(1.02 to 1.41)
Stress-related disorder	After pregnancy	40-44	1.04(0.89 to 1.22)	1.04(0.88 to 1.22)	1.04(0.88 to 1.22)
Stress-related disorder	After pregnancy	45-49	1.17(0.99 to 1.38)	1.16(0.98 to 1.37)	1.17(0.99 to 1.37)
Stress-related disorder	After pregnancy	50-51	1.10(0.84 to 1.44)	1.08(0.83 to 1.42)	1.08(0.83 to 1.41)
Alcohol use disorder	During Pregnancy	0-4	0.63(0.46 to 0.87)	0.63(0.46 to 0.86)	0.63(0.46 to 0.87)
Alcohol use disorder	During Pregnancy	5-9	0.98(0.70 to 1.36)	0.97(0.70 to 1.36)	0.99(0.71 to 1.38)
Alcohol use disorder	During Pregnancy	10-14	0.79(0.57 to 1.10)	0.78(0.56 to 1.08)	0.78(0.56 to 1.09)
Alcohol use disorder	During Pregnancy	15-19	0.75(0.54 to 1.05)	0.74(0.53 to 1.04)	0.75(0.53 to 1.04)
Alcohol use disorder	During Pregnancy	20-24	0.66(0.48 to 0.90)	0.64(0.47 to 0.88)	0.65(0.47 to 0.89)
Alcohol use disorder	During Pregnancy	25-29	0.88(0.63 to 1.23)	0.87(0.63 to 1.21)	0.88(0.63 to 1.23)
Alcohol use disorder	During Pregnancy	30-34	0.86(0.62 to 1.18)	0.85(0.62 to 1.18)	0.85(0.62 to 1.18)
Alcohol use disorder	During Pregnancy	35-39	0.56(0.39 to 0.81)	0.57(0.40 to 0.82)	0.58(0.40 to 0.83)

© 2026 Xiang N et al. *JAMA Network Open*.

Alcohol use disorder	After Pregnancy	0-4	0.48(0.34 to 0.68)	0.49(0.35 to 0.69)	0.49(0.35 to 0.70)
Alcohol use disorder	After Pregnancy	5-9	0.73(0.52 to 1.05)	0.74(0.52 to 1.05)	0.76(0.53 to 1.08)
Alcohol use disorder	After Pregnancy	10-14	0.92(0.67 to 1.26)	0.90(0.65 to 1.24)	0.91(0.66 to 1.26)
Alcohol use disorder	After Pregnancy	15-19	0.84(0.61 to 1.16)	0.84(0.61 to 1.16)	0.85(0.61 to 1.17)
Alcohol use disorder	After Pregnancy	20-24	0.64(0.47 to 0.87)	0.61(0.45 to 0.85)	0.62(0.45 to 0.86)
Alcohol use disorder	After Pregnancy	25-29	1.04(0.76 to 1.43)	0.99(0.72 to 1.36)	1.00(0.73 to 1.38)
Alcohol use disorder	After Pregnancy	30-34	0.78(0.56 to 1.08)	0.78(0.56 to 1.08)	0.78(0.56 to 1.09)
Alcohol use disorder	After Pregnancy	35-39	0.74(0.53 to 1.02)	0.73(0.53 to 1.01)	0.74(0.54 to 1.02)
Alcohol use disorder	After Pregnancy	40-44	0.92(0.67 to 1.27)	0.92(0.66 to 1.26)	0.92(0.67 to 1.28)
Alcohol use disorder	After Pregnancy	45-49	0.61(0.44 to 0.85)	0.59(0.42 to 0.83)	0.60(0.43 to 0.84)
Alcohol use disorder	After Pregnancy	50-51	0.91(0.56 to 1.46)	0.85(0.52 to 1.39)	0.86(0.53 to 1.40)
Tobacco use disorder	During Pregnancy	0-4	1.21(0.68 to 2.14)	1.21(0.68 to 2.14)	1.22(0.69 to 2.16)
Tobacco use disorder	During Pregnancy	5-9	0.77(0.42 to 1.41)	0.72(0.39 to 1.33)	0.72(0.39 to 1.32)
Tobacco use disorder	During Pregnancy	10-14	1.13(0.64 to 2.00)	1.07(0.61 to 1.91)	1.08(0.61 to 1.92)
Tobacco use disorder	During Pregnancy	15-19	1.01(0.51 to 1.99)	1.01(0.51 to 1.98)	1.01(0.51 to 1.99)
Tobacco use disorder	During Pregnancy	20-24	1.29(0.63 to 2.66)	1.28(0.62 to 2.64)	1.26(0.61 to 2.61)
Tobacco use disorder	During Pregnancy	25-29	1.18(0.66 to 2.12)	1.13(0.62 to 2.04)	1.11(0.61 to 2.00)
Tobacco use disorder	During Pregnancy	30-34	2.35(1.25 to 4.43)	2.36(1.25 to 4.44)	2.36(1.25 to 4.45)
Tobacco use disorder	During Pregnancy	35-39	1.39(0.78 to 2.49)	1.39(0.78 to 2.48)	1.41(0.79 to 2.52)
Tobacco use disorder	After Pregnancy	0-4	1.04(0.58 to 1.87)	1.05(0.58 to 1.89)	1.06(0.59 to 1.91)
Tobacco use disorder	After Pregnancy	5-9	0.95(0.53 to 1.68)	0.94(0.53 to 1.66)	0.93(0.52 to 1.66)
Tobacco use disorder	After Pregnancy	10-14	0.70(0.37 to 1.32)	0.65(0.34 to 1.25)	0.66(0.35 to 1.26)
Tobacco use disorder	After Pregnancy	15-19	0.85(0.42 to 1.72)	0.86(0.42 to 1.73)	0.86(0.42 to 1.74)
Tobacco use disorder	After Pregnancy	20-24	1.30(0.63 to 2.67)	1.31(0.63 to 2.69)	1.29(0.62 to 2.65)
Tobacco use disorder	After Pregnancy	25-29	1.06(0.58 to 1.93)	1.02(0.56 to 1.87)	1.00(0.55 to 1.84)
Tobacco use disorder	After Pregnancy	30-34	1.39(0.70 to 2.79)	1.41(0.71 to 2.81)	1.41(0.71 to 2.82)
Tobacco use disorder	After Pregnancy	35-39	1.56(0.90 to 2.71)	1.52(0.88 to 2.64)	1.53(0.88 to 2.65)
Tobacco use disorder	After Pregnancy	40-44	1.16(0.64 to 2.09)	1.13(0.62 to 2.04)	1.14(0.63 to 2.07)
Tobacco use disorder	After Pregnancy	45-49	1.37(0.79 to 2.40)	1.34(0.76 to 2.35)	1.34(0.76 to 2.35)
Tobacco use disorder	After Pregnancy	50-51	0.99(0.44 to 2.24)	0.99(0.44 to 2.25)	0.99(0.44 to 2.25)
Drug use disorder	During Pregnancy	0-4	0.84(0.55 to 1.27)	0.80(0.53 to 1.22)	0.83(0.54 to 1.26)
Drug use disorder	During Pregnancy	5-9	0.67(0.44 to 1.01)	0.64(0.42 to 0.96)	0.65(0.43 to 0.99)
Drug use disorder	During Pregnancy	10-14	0.99(0.64 to 1.53)	0.98(0.63 to 1.50)	1.00(0.65 to 1.54)
Drug use disorder	During Pregnancy	15-19	0.76(0.51 to 1.14)	0.76(0.51 to 1.13)	0.78(0.52 to 1.16)
Drug use disorder	During Pregnancy	20-24	0.82(0.56 to 1.22)	0.78(0.52 to 1.15)	0.79(0.53 to 1.18)
Drug use disorder	During Pregnancy	25-29	0.78(0.51 to 1.19)	0.79(0.52 to 1.21)	0.81(0.53 to 1.23)
Drug use disorder	During Pregnancy	30-34	0.80(0.52 to 1.22)	0.80(0.52 to 1.22)	0.81(0.53 to 1.24)
Drug use disorder	During Pregnancy	35-39	0.72(0.44 to 1.18)	0.73(0.44 to 1.20)	0.74(0.45 to 1.22)
Drug use disorder	After Pregnancy	0-4	0.48(0.30 to 0.78)	0.45(0.27 to 0.73)	0.46(0.28 to 0.75)
Drug use disorder	After Pregnancy	5-9	0.71(0.47 to 1.06)	0.69(0.46 to 1.03)	0.70(0.47 to 1.06)
Drug use disorder	After Pregnancy	10-14	0.88(0.56 to 1.37)	0.87(0.56 to 1.36)	0.90(0.57 to 1.40)
Drug use disorder	After Pregnancy	15-19	0.81(0.55 to 1.21)	0.78(0.52 to 1.17)	0.81(0.54 to 1.21)
Drug use disorder	After Pregnancy	20-24	0.85(0.57 to 1.25)	0.79(0.53 to 1.18)	0.81(0.55 to 1.21)
Drug use disorder	After Pregnancy	25-29	0.77(0.51 to 1.18)	0.73(0.48 to 1.13)	0.75(0.49 to 1.16)
Drug use disorder	After Pregnancy	30-34	0.86(0.56 to 1.30)	0.86(0.56 to 1.31)	0.87(0.57 to 1.33)

© 2026 Xiang N et al. *JAMA Network Open*.

Drug use disorder	After Pregnancy	35-39	1.17(0.77 to 1.79)	1.16(0.76 to 1.77)	1.16(0.76 to 1.78)
Drug use disorder	After Pregnancy	40-44	1.10(0.74 to 1.63)	1.13(0.76 to 1.68)	1.15(0.77 to 1.70)
Drug use disorder	After Pregnancy	45-49	1.14(0.75 to 1.73)	1.12(0.74 to 1.70)	1.14(0.75 to 1.73)
Drug use disorder	After Pregnancy	50-51	1.42(0.71 to 2.86)	1.28(0.63 to 2.62)	1.30(0.64 to 2.66)
ADHD	During Pregnancy	0-4	1.00(0.69 to 1.46)	0.94(0.64 to 1.38)	0.95(0.65 to 1.40)
ADHD	During Pregnancy	5-9	0.98(0.68 to 1.41)	0.96(0.67 to 1.39)	0.97(0.67 to 1.41)
ADHD	During Pregnancy	10-14	0.97(0.64 to 1.47)	0.96(0.64 to 1.45)	0.98(0.65 to 1.48)
ADHD	During Pregnancy	15-19	0.99(0.66 to 1.48)	0.97(0.65 to 1.46)	0.99(0.66 to 1.49)
ADHD	During Pregnancy	20-24	1.09(0.75 to 1.56)	1.08(0.75 to 1.55)	1.08(0.75 to 1.55)
ADHD	During Pregnancy	25-29	1.40(0.97 to 2.03)	1.39(0.96 to 2.01)	1.39(0.96 to 2.01)
ADHD	During Pregnancy	30-34	1.20(0.82 to 1.76)	1.14(0.77 to 1.68)	1.14(0.78 to 1.68)
ADHD	During Pregnancy	35-39	1.89(1.20 to 2.97)	1.91(1.21 to 3.01)	1.91(1.21 to 3.01)
ADHD	After Pregnancy	0-4	1.01(0.69 to 1.47)	0.97(0.66 to 1.42)	0.99(0.68 to 1.45)
ADHD	After Pregnancy	5-9	0.96(0.66 to 1.38)	0.93(0.65 to 1.35)	0.95(0.66 to 1.37)
ADHD	After Pregnancy	10-14	1.38(0.94 to 2.03)	1.36(0.93 to 2.00)	1.40(0.95 to 2.05)
ADHD	After Pregnancy	15-19	1.05(0.70 to 1.56)	1.04(0.70 to 1.56)	1.07(0.71 to 1.59)
ADHD	After Pregnancy	20-24	1.17(0.82 to 1.68)	1.16(0.81 to 1.66)	1.17(0.82 to 1.67)
ADHD	After Pregnancy	25-29	1.09(0.74 to 1.60)	1.08(0.73 to 1.60)	1.09(0.74 to 1.61)
ADHD	After Pregnancy	30-34	1.35(0.93 to 1.97)	1.34(0.92 to 1.95)	1.35(0.93 to 1.97)
ADHD	After Pregnancy	35-39	1.46(0.92 to 2.31)	1.44(0.90 to 2.29)	1.45(0.91 to 2.31)
ADHD	After Pregnancy	40-44	0.80(0.57 to 1.14)	0.81(0.57 to 1.14)	0.81(0.57 to 1.14)
ADHD	After Pregnancy	45-49	0.78(0.54 to 1.14)	0.80(0.55 to 1.16)	0.80(0.55 to 1.17)
ADHD	After Pregnancy	50-51	1.27(0.72 to 2.26)	1.18(0.66 to 2.12)	1.21(0.67 to 2.16)
Bipolar disorder	During Pregnancy	0-4	1.62(0.66 to 3.99)	1.60(0.65 to 3.92)	1.60(0.65 to 3.92)
Bipolar disorder	During Pregnancy	5-9	1.00(0.44 to 2.29)	0.99(0.43 to 2.26)	1.01(0.44 to 2.29)
Bipolar disorder	During Pregnancy	10-14	0.94(0.41 to 2.18)	0.93(0.40 to 2.16)	0.95(0.41 to 2.19)
Bipolar disorder	During Pregnancy	15-19	1.19(0.58 to 2.48)	1.18(0.57 to 2.45)	1.17(0.56 to 2.43)
Bipolar disorder	During Pregnancy	20-24	0.88(0.40 to 1.93)	0.97(0.43 to 2.15)	0.94(0.42 to 2.11)
Bipolar disorder	During Pregnancy	25-29	0.93(0.49 to 1.75)	0.93(0.49 to 1.75)	0.93(0.49 to 1.75)
Bipolar disorder	During Pregnancy	30-34	0.85(0.42 to 1.75)	0.86(0.42 to 1.75)	0.86(0.42 to 1.77)
Bipolar disorder	During Pregnancy	35-39	1.41(0.60 to 3.31)	1.43(0.61 to 3.35)	1.42(0.61 to 3.33)
Bipolar disorder	After Pregnancy	0-4	1.50(0.60 to 3.71)	1.49(0.60 to 3.69)	1.49(0.60 to 3.69)
Bipolar disorder	After Pregnancy	5-9	1.30(0.60 to 2.84)	1.29(0.59 to 2.83)	1.32(0.60 to 2.87)
Bipolar disorder	After Pregnancy	10-14	1.25(0.57 to 2.75)	1.17(0.52 to 2.60)	1.19(0.53 to 2.64)
Bipolar disorder	After Pregnancy	15-19	1.06(0.50 to 2.24)	0.92(0.43 to 2.00)	0.92(0.42 to 1.99)
Bipolar disorder	After Pregnancy	20-24	0.89(0.40 to 1.94)	0.98(0.44 to 2.18)	0.96(0.43 to 2.14)
Bipolar disorder	After Pregnancy	25-29	0.84(0.44 to 1.62)	0.85(0.44 to 1.63)	0.85(0.44 to 1.63)
Bipolar disorder	After Pregnancy	30-34	1.50(0.79 to 2.83)	1.51(0.80 to 2.85)	1.53(0.81 to 2.89)
Bipolar disorder	After Pregnancy	35-39	1.68(0.75 to 3.77)	1.69(0.75 to 3.80)	1.69(0.75 to 3.80)
Bipolar disorder	After Pregnancy	40-44	1.06(0.52 to 2.16)	1.06(0.52 to 2.18)	1.04(0.51 to 2.13)
Bipolar disorder	After Pregnancy	45-49	2.52(1.12 to 5.69)	2.88(1.23 to 6.78)	2.89(1.23 to 6.79)
Psychosis	During Pregnancy	0-4	1.41(0.52 to 3.82)	1.26(0.46 to 3.47)	1.27(0.46 to 3.50)
Psychosis	During Pregnancy	5-9	0.93(0.42 to 2.09)	0.91(0.41 to 2.04)	0.90(0.40 to 2.02)
Psychosis	During Pregnancy	10-14	1.48(0.63 to 3.49)	1.41(0.60 to 3.34)	1.41(0.60 to 3.34)
Psychosis	During Pregnancy	15-19	1.95(0.81 to 4.69)	1.65(0.67 to 4.06)	1.68(0.68 to 4.12)

© 2026 Xiang N et al. *JAMA Network Open*.

Psychosis	During Pregnancy	20-24	0.98(0.42 to 2.27)	0.96(0.41 to 2.22)	0.96(0.42 to 2.23)
Psychosis	During Pregnancy	25-29	1.29(0.56 to 2.99)	1.29(0.56 to 2.98)	1.30(0.56 to 3.01)
Psychosis	During Pregnancy	30-34	1.40(0.58 to 3.38)	1.39(0.58 to 3.36)	1.37(0.57 to 3.30)
Psychosis	During Pregnancy	35-39	1.46(0.68 to 3.16)	1.49(0.67 to 3.33)	1.48(0.66 to 3.31)
Psychosis	After Pregnancy	0-4	1.39(0.51 to 3.76)	1.38(0.51 to 3.75)	1.40(0.52 to 3.79)
Psychosis	After Pregnancy	5-9	0.92(0.41 to 2.06)	0.91(0.41 to 2.04)	0.91(0.40 to 2.02)
Psychosis	After Pregnancy	10-14	1.08(0.43 to 2.67)	1.04(0.42 to 2.59)	1.04(0.42 to 2.60)
Psychosis	After Pregnancy	15-19	1.60(0.65 to 3.97)	1.45(0.58 to 3.63)	1.47(0.59 to 3.70)
Psychosis	After Pregnancy	20-24	0.99(0.43 to 2.28)	0.97(0.42 to 2.26)	0.98(0.43 to 2.28)
Psychosis	After Pregnancy	25-29	1.12(0.47 to 2.66)	1.13(0.48 to 2.68)	1.15(0.48 to 2.72)
Psychosis	After Pregnancy	30-34	1.75(0.75 to 4.10)	1.65(0.70 to 3.89)	1.63(0.69 to 3.84)
Psychosis	After Pregnancy	35-39	1.06(0.48 to 2.37)	0.99(0.42 to 2.33)	0.99(0.42 to 2.33)
Psychosis	After Pregnancy	45-49	1.36(0.65 to 2.85)	1.29(0.61 to 2.73)	1.28(0.61 to 2.71)

*ADHD, attention deficit hyperactivity disorder.

* The incidence rate ratio was estimated by every 5 weeks, by comparing the incidence in each interval during and after pregnancy with that before pregnancy. Model 1 was adjusted for age and calendar year at childbirth and week at follow-up. Model 2 was additionally adjusted for country of birth, region of residence and education before pregnancy, season at childbirth, and income before pregnancy. Model 3 was additionally adjusted for civil status during pregnancy, multiple gestation, number of children, and history of psychiatric disorders.

© 2026 Xiang N et al. *JAMA Network Open*.

eTable 7. Incidence Rate Ratios of Any Paternal Psychiatric Disorder and 9 Type-Specific Disorders During and After Pregnancy, Restricted to Childbirths With Complete 1-Year Preconception Follow-Up

Psychiatric disorders	Phase	Weeks	Incidence rate differences per 1000 person-years		
			Model 1	Model 2	Model 3
Any psychiatric disorder	During pregnancy	0-4	0.93(0.86 to 1.01)	0.93(0.86 to 1.01)	0.93(0.86 to 1.01)
Any psychiatric disorder	During pregnancy	5-9	0.84(0.77 to 0.90)	0.84(0.77 to 0.91)	0.84(0.77 to 0.91)
Any psychiatric disorder	During pregnancy	10-14	0.90(0.83 to 0.97)	0.90(0.83 to 0.97)	0.90(0.83 to 0.97)
Any psychiatric disorder	During pregnancy	15-19	0.86(0.80 to 0.93)	0.86(0.80 to 0.93)	0.86(0.80 to 0.93)
Any psychiatric disorder	During pregnancy	20-24	0.97(0.90 to 1.05)	0.97(0.90 to 1.06)	0.97(0.90 to 1.06)
Any psychiatric disorder	During pregnancy	25-29	0.89(0.82 to 0.97)	0.89(0.82 to 0.97)	0.89(0.82 to 0.97)
Any psychiatric disorder	During pregnancy	30-34	0.88(0.81 to 0.95)	0.88(0.81 to 0.95)	0.88(0.81 to 0.95)
Any psychiatric disorder	During pregnancy	35-39	0.78(0.72 to 0.85)	0.78(0.72 to 0.86)	0.78(0.72 to 0.86)
Any psychiatric disorder	After pregnancy	0-4	0.80(0.74 to 0.87)	0.80(0.74 to 0.87)	0.80(0.74 to 0.87)
Any psychiatric disorder	After pregnancy	5-9	0.86(0.79 to 0.93)	0.86(0.80 to 0.93)	0.86(0.80 to 0.93)
Any psychiatric disorder	After pregnancy	10-14	0.87(0.80 to 0.94)	0.87(0.80 to 0.94)	0.87(0.80 to 0.95)
Any psychiatric disorder	After pregnancy	15-19	0.89(0.82 to 0.96)	0.89(0.82 to 0.97)	0.89(0.83 to 0.97)
Any psychiatric disorder	After pregnancy	20-24	0.92(0.85 to 1.00)	0.93(0.85 to 1.01)	0.93(0.86 to 1.01)
Any psychiatric disorder	After pregnancy	25-29	0.95(0.87 to 1.03)	0.95(0.88 to 1.04)	0.96(0.88 to 1.04)
Any psychiatric disorder	After pregnancy	30-34	0.93(0.85 to 1.00)	0.93(0.86 to 1.01)	0.93(0.86 to 1.01)
Any psychiatric disorder	After pregnancy	35-39	0.91(0.84 to 0.99)	0.92(0.85 to 1.00)	0.92(0.85 to 1.00)
Any psychiatric disorder	After pregnancy	40-44	0.91(0.84 to 0.98)	0.91(0.84 to 0.99)	0.92(0.84 to 0.99)
Any psychiatric disorder	After pregnancy	45-49	1.01(0.93 to 1.10)	1.02(0.94 to 1.11)	1.02(0.94 to 1.11)
Any psychiatric disorder	After pregnancy	50-51	0.92(0.81 to 1.05)	0.93(0.81 to 1.06)	0.93(0.81 to 1.06)
Depression	During pregnancy	0-4	0.87(0.77 to 1.00)	0.88(0.77 to 1.00)	0.88(0.77 to 1.00)
Depression	During pregnancy	5-9	0.84(0.73 to 0.95)	0.84(0.73 to 0.95)	0.84(0.74 to 0.96)
Depression	During pregnancy	10-14	0.84(0.73 to 0.95)	0.84(0.73 to 0.95)	0.84(0.73 to 0.96)
Depression	During pregnancy	15-19	0.89(0.78 to 1.01)	0.89(0.78 to 1.01)	0.89(0.78 to 1.01)
Depression	During pregnancy	20-24	0.94(0.82 to 1.08)	0.94(0.82 to 1.08)	0.94(0.82 to 1.08)
Depression	During pregnancy	25-29	0.78(0.68 to 0.90)	0.78(0.68 to 0.90)	0.78(0.68 to 0.90)
Depression	During pregnancy	30-34	0.75(0.66 to 0.87)	0.76(0.66 to 0.87)	0.76(0.66 to 0.87)
Depression	During pregnancy	35-39	0.74(0.64 to 0.85)	0.74(0.64 to 0.86)	0.74(0.64 to 0.86)
Depression	After pregnancy	0-4	0.71(0.62 to 0.82)	0.72(0.62 to 0.82)	0.72(0.63 to 0.83)
Depression	After pregnancy	5-9	0.76(0.66 to 0.87)	0.76(0.67 to 0.87)	0.77(0.67 to 0.88)
Depression	After pregnancy	10-14	0.91(0.80 to 1.04)	0.91(0.80 to 1.04)	0.92(0.81 to 1.04)
Depression	After pregnancy	15-19	1.00(0.88 to 1.13)	1.00(0.88 to 1.14)	1.01(0.89 to 1.15)
Depression	After pregnancy	20-24	1.01(0.88 to 1.16)	1.01(0.89 to 1.16)	1.02(0.89 to 1.17)
Depression	After pregnancy	25-29	0.99(0.87 to 1.13)	0.99(0.87 to 1.13)	1.00(0.88 to 1.14)
Depression	After pregnancy	30-34	1.03(0.90 to 1.16)	1.03(0.91 to 1.17)	1.04(0.91 to 1.18)
Depression	After pregnancy	35-39	1.06(0.93 to 1.20)	1.07(0.94 to 1.21)	1.07(0.94 to 1.22)
Depression	After pregnancy	40-44	1.15(1.01 to 1.31)	1.16(1.02 to 1.32)	1.17(1.03 to 1.33)
Depression	After pregnancy	45-49	1.17(1.02 to 1.35)	1.18(1.03 to 1.35)	1.19(1.04 to 1.36)
Depression	After pregnancy	50-51	1.30(1.04 to 1.62)	1.30(1.04 to 1.63)	1.31(1.05 to 1.64)
Anxiety	During pregnancy	0-4	1.05(0.94 to 1.18)	1.05(0.94 to 1.18)	1.06(0.94 to 1.19)
Anxiety	During pregnancy	5-9	0.91(0.81 to 1.03)	0.91(0.81 to 1.03)	0.92(0.81 to 1.04)

© 2026 Xiang N et al. *JAMA Network Open*.

Anxiety	During pregnancy	10-14	0.95(0.84 to 1.07)	0.95(0.84 to 1.07)	0.95(0.85 to 1.07)
Anxiety	During pregnancy	15-19	0.89(0.78 to 1.00)	0.89(0.79 to 1.00)	0.89(0.79 to 1.00)
Anxiety	During pregnancy	20-24	1.09(0.97 to 1.23)	1.09(0.97 to 1.23)	1.10(0.97 to 1.24)
Anxiety	During pregnancy	25-29	1.05(0.93 to 1.18)	1.05(0.93 to 1.18)	1.05(0.93 to 1.18)
Anxiety	During pregnancy	30-34	1.07(0.95 to 1.21)	1.08(0.95 to 1.21)	1.08(0.96 to 1.22)
Anxiety	During pregnancy	35-39	0.85(0.75 to 0.97)	0.86(0.76 to 0.97)	0.86(0.76 to 0.97)
Anxiety	After pregnancy	0-4	0.89(0.79 to 1.01)	0.89(0.79 to 1.01)	0.90(0.80 to 1.02)
Anxiety	After pregnancy	5-9	0.98(0.87 to 1.10)	0.98(0.87 to 1.10)	0.99(0.88 to 1.11)
Anxiety	After pregnancy	10-14	0.95(0.84 to 1.07)	0.95(0.84 to 1.07)	0.96(0.85 to 1.08)
Anxiety	After pregnancy	15-19	1.02(0.91 to 1.15)	1.02(0.91 to 1.15)	1.03(0.92 to 1.16)
Anxiety	After pregnancy	20-24	1.09(0.96 to 1.23)	1.09(0.97 to 1.23)	1.10(0.97 to 1.24)
Anxiety	After pregnancy	25-29	1.03(0.91 to 1.16)	1.04(0.92 to 1.17)	1.04(0.93 to 1.18)
Anxiety	After pregnancy	30-34	1.03(0.91 to 1.16)	1.03(0.91 to 1.17)	1.04(0.92 to 1.17)
Anxiety	After pregnancy	35-39	0.85(0.75 to 0.95)	0.85(0.75 to 0.96)	0.86(0.76 to 0.97)
Anxiety	After pregnancy	40-44	0.85(0.75 to 0.96)	0.85(0.75 to 0.96)	0.86(0.76 to 0.97)
Anxiety	After pregnancy	45-49	1.00(0.89 to 1.14)	1.01(0.89 to 1.14)	1.02(0.90 to 1.15)
Anxiety	After pregnancy	50-51	1.00(0.83 to 1.21)	1.01(0.83 to 1.21)	1.01(0.84 to 1.21)
Stress-related disorder	During pregnancy	0-4	0.94(0.82 to 1.07)	0.94(0.82 to 1.07)	0.94(0.82 to 1.07)
Stress-related disorder	During pregnancy	5-9	0.85(0.74 to 0.97)	0.85(0.74 to 0.97)	0.85(0.74 to 0.97)
Stress-related disorder	During pregnancy	10-14	0.99(0.87 to 1.13)	0.99(0.87 to 1.13)	0.99(0.87 to 1.13)
Stress-related disorder	During pregnancy	15-19	0.92(0.80 to 1.05)	0.92(0.80 to 1.05)	0.92(0.81 to 1.05)
Stress-related disorder	During pregnancy	20-24	1.01(0.89 to 1.15)	1.01(0.89 to 1.15)	1.01(0.89 to 1.15)
Stress-related disorder	During pregnancy	25-29	0.93(0.81 to 1.07)	0.93(0.81 to 1.07)	0.94(0.82 to 1.07)
Stress-related disorder	During pregnancy	30-34	0.87(0.76 to 1.00)	0.87(0.76 to 1.00)	0.88(0.77 to 1.00)
Stress-related disorder	During pregnancy	35-39	0.95(0.82 to 1.09)	0.95(0.83 to 1.09)	0.96(0.83 to 1.10)
Stress-related disorder	After pregnancy	0-4	1.06(0.93 to 1.20)	1.06(0.93 to 1.20)	1.06(0.94 to 1.21)
Stress-related disorder	After pregnancy	5-9	1.20(1.06 to 1.37)	1.20(1.06 to 1.37)	1.21(1.07 to 1.37)
Stress-related disorder	After pregnancy	10-14	1.08(0.95 to 1.23)	1.08(0.95 to 1.24)	1.09(0.96 to 1.24)
Stress-related disorder	After pregnancy	15-19	1.05(0.92 to 1.19)	1.05(0.93 to 1.20)	1.06(0.93 to 1.20)
Stress-related disorder	After pregnancy	20-24	1.11(0.98 to 1.26)	1.11(0.98 to 1.26)	1.12(0.98 to 1.27)
Stress-related disorder	After pregnancy	25-29	1.18(1.04 to 1.35)	1.19(1.04 to 1.35)	1.19(1.05 to 1.36)
Stress-related disorder	After pregnancy	30-34	1.08(0.95 to 1.23)	1.08(0.95 to 1.23)	1.09(0.95 to 1.24)
Stress-related disorder	After pregnancy	35-39	1.19(1.05 to 1.35)	1.19(1.05 to 1.36)	1.20(1.06 to 1.37)
Stress-related disorder	After pregnancy	40-44	1.01(0.88 to 1.15)	1.01(0.89 to 1.16)	1.02(0.89 to 1.16)
Stress-related disorder	After pregnancy	45-49	1.10(0.96 to 1.25)	1.10(0.96 to 1.26)	1.11(0.97 to 1.27)
Stress-related disorder	After pregnancy	50-51	1.09(0.87 to 1.36)	1.09(0.87 to 1.37)	1.10(0.88 to 1.37)
Alcohol use disorder	During pregnancy	0-4	0.61(0.49 to 0.74)	0.61(0.50 to 0.74)	0.61(0.50 to 0.75)
Alcohol use disorder	During pregnancy	5-9	0.73(0.60 to 0.89)	0.73(0.60 to 0.89)	0.73(0.60 to 0.89)
Alcohol use disorder	During pregnancy	10-14	0.78(0.63 to 0.95)	0.78(0.63 to 0.96)	0.78(0.64 to 0.96)
Alcohol use disorder	During pregnancy	15-19	0.70(0.57 to 0.86)	0.70(0.57 to 0.86)	0.70(0.57 to 0.86)
Alcohol use disorder	During pregnancy	20-24	0.65(0.53 to 0.79)	0.65(0.53 to 0.79)	0.65(0.53 to 0.79)
Alcohol use disorder	During pregnancy	25-29	0.80(0.64 to 1.00)	0.80(0.64 to 1.00)	0.80(0.64 to 1.00)
Alcohol use disorder	During pregnancy	30-34	0.72(0.58 to 0.90)	0.72(0.58 to 0.90)	0.72(0.58 to 0.90)
Alcohol use disorder	During pregnancy	35-39	0.54(0.43 to 0.69)	0.55(0.43 to 0.70)	0.55(0.43 to 0.70)
Alcohol use disorder	After pregnancy	0-4	0.47(0.38 to 0.59)	0.47(0.38 to 0.59)	0.48(0.38 to 0.59)

© 2026 Xiang N et al. *JAMA Network Open*.

Alcohol use disorder	After pregnancy	5-9	0.59(0.48 to 0.73)	0.60(0.48 to 0.74)	0.60(0.48 to 0.74)
Alcohol use disorder	After pregnancy	10-14	0.67(0.54 to 0.83)	0.67(0.54 to 0.83)	0.68(0.55 to 0.84)
Alcohol use disorder	After pregnancy	15-19	0.70(0.57 to 0.86)	0.70(0.57 to 0.86)	0.71(0.57 to 0.86)
Alcohol use disorder	After pregnancy	20-24	0.56(0.45 to 0.70)	0.56(0.45 to 0.70)	0.57(0.46 to 0.70)
Alcohol use disorder	After pregnancy	25-29	0.95(0.77 to 1.18)	0.96(0.77 to 1.18)	0.96(0.78 to 1.19)
Alcohol use disorder	After pregnancy	30-34	0.83(0.68 to 1.03)	0.84(0.68 to 1.04)	0.84(0.68 to 1.04)
Alcohol use disorder	After pregnancy	35-39	0.82(0.67 to 1.01)	0.82(0.67 to 1.01)	0.83(0.67 to 1.02)
Alcohol use disorder	After pregnancy	40-44	0.78(0.63 to 0.95)	0.78(0.63 to 0.96)	0.78(0.64 to 0.96)
Alcohol use disorder	After pregnancy	45-49	0.88(0.72 to 1.09)	0.88(0.72 to 1.09)	0.89(0.72 to 1.09)
Alcohol use disorder	After pregnancy	50-51	0.85(0.62 to 1.17)	0.85(0.62 to 1.18)	0.86(0.62 to 1.19)
Tobacco use disorder	During pregnancy	0-4	1.14(0.76 to 1.73)	1.14(0.76 to 1.73)	1.14(0.76 to 1.73)
Tobacco use disorder	During pregnancy	5-9	1.00(0.66 to 1.52)	1.00(0.66 to 1.52)	1.00(0.66 to 1.52)
Tobacco use disorder	During pregnancy	10-14	1.04(0.71 to 1.52)	1.04(0.71 to 1.52)	1.04(0.71 to 1.52)
Tobacco use disorder	During pregnancy	15-19	0.91(0.60 to 1.39)	0.91(0.60 to 1.39)	0.91(0.60 to 1.39)
Tobacco use disorder	During pregnancy	20-24	1.00(0.66 to 1.52)	1.00(0.66 to 1.52)	1.00(0.66 to 1.52)
Tobacco use disorder	During pregnancy	25-29	1.00(0.66 to 1.53)	1.00(0.66 to 1.53)	1.00(0.66 to 1.53)
Tobacco use disorder	During pregnancy	30-34	1.27(0.84 to 1.92)	1.27(0.84 to 1.92)	1.27(0.84 to 1.92)
Tobacco use disorder	During pregnancy	35-39	1.19(0.80 to 1.75)	1.19(0.81 to 1.76)	1.20(0.81 to 1.76)
Tobacco use disorder	After pregnancy	0-4	1.07(0.70 to 1.63)	1.07(0.71 to 1.64)	1.08(0.71 to 1.64)
Tobacco use disorder	After pregnancy	5-9	1.09(0.73 to 1.65)	1.10(0.73 to 1.65)	1.10(0.73 to 1.65)
Tobacco use disorder	After pregnancy	10-14	0.72(0.47 to 1.09)	0.72(0.47 to 1.09)	0.72(0.47 to 1.09)
Tobacco use disorder	After pregnancy	15-19	0.79(0.51 to 1.22)	0.79(0.51 to 1.23)	0.79(0.51 to 1.23)
Tobacco use disorder	After pregnancy	20-24	1.11(0.74 to 1.67)	1.11(0.74 to 1.68)	1.11(0.74 to 1.68)
Tobacco use disorder	After pregnancy	25-29	0.95(0.62 to 1.46)	0.96(0.62 to 1.48)	0.96(0.63 to 1.48)
Tobacco use disorder	After pregnancy	30-34	1.32(0.87 to 1.99)	1.33(0.88 to 2.01)	1.33(0.88 to 2.01)
Tobacco use disorder	After pregnancy	35-39	1.17(0.80 to 1.70)	1.18(0.81 to 1.72)	1.18(0.81 to 1.73)
Tobacco use disorder	After pregnancy	40-44	1.09(0.76 to 1.57)	1.11(0.77 to 1.59)	1.12(0.78 to 1.60)
Tobacco use disorder	After pregnancy	45-49	1.09(0.74 to 1.62)	1.11(0.75 to 1.65)	1.11(0.75 to 1.65)
Tobacco use disorder	After pregnancy	50-51	1.29(0.64 to 2.59)	1.30(0.65 to 2.62)	1.31(0.65 to 2.63)
Drug use disorder	During pregnancy	0-4	0.70(0.56 to 0.88)	0.71(0.56 to 0.89)	0.71(0.57 to 0.89)
Drug use disorder	During pregnancy	5-9	0.78(0.62 to 0.98)	0.78(0.62 to 0.99)	0.79(0.62 to 0.99)
Drug use disorder	During pregnancy	10-14	0.77(0.62 to 0.97)	0.78(0.62 to 0.97)	0.78(0.62 to 0.97)
Drug use disorder	During pregnancy	15-19	0.78(0.63 to 0.97)	0.79(0.63 to 0.98)	0.79(0.64 to 0.98)
Drug use disorder	During pregnancy	20-24	0.80(0.64 to 1.00)	0.80(0.64 to 1.01)	0.80(0.64 to 1.01)
Drug use disorder	During pregnancy	25-29	0.77(0.62 to 0.95)	0.77(0.62 to 0.96)	0.77(0.62 to 0.96)
Drug use disorder	During pregnancy	30-34	0.84(0.66 to 1.05)	0.84(0.66 to 1.06)	0.84(0.67 to 1.06)
Drug use disorder	During pregnancy	35-39	0.77(0.60 to 1.00)	0.78(0.60 to 1.01)	0.79(0.61 to 1.02)
Drug use disorder	After pregnancy	0-4	0.55(0.43 to 0.70)	0.55(0.43 to 0.71)	0.56(0.44 to 0.71)
Drug use disorder	After pregnancy	5-9	0.81(0.64 to 1.02)	0.81(0.64 to 1.02)	0.82(0.65 to 1.04)
Drug use disorder	After pregnancy	10-14	0.83(0.67 to 1.03)	0.83(0.67 to 1.04)	0.84(0.68 to 1.05)
Drug use disorder	After pregnancy	15-19	0.70(0.56 to 0.88)	0.71(0.57 to 0.89)	0.72(0.57 to 0.90)
Drug use disorder	After pregnancy	20-24	0.83(0.66 to 1.04)	0.83(0.67 to 1.05)	0.84(0.67 to 1.06)
Drug use disorder	After pregnancy	25-29	0.57(0.45 to 0.73)	0.58(0.46 to 0.74)	0.59(0.46 to 0.75)
Drug use disorder	After pregnancy	30-34	0.89(0.70 to 1.12)	0.90(0.71 to 1.13)	0.91(0.72 to 1.14)
Drug use disorder	After pregnancy	35-39	0.85(0.66 to 1.08)	0.86(0.67 to 1.09)	0.87(0.68 to 1.10)

© 2026 Xiang N et al. *JAMA Network Open*.

Drug use disorder	After pregnancy	40-44	0.96(0.75 to 1.22)	0.97(0.76 to 1.23)	0.98(0.77 to 1.24)
Drug use disorder	After pregnancy	45-49	0.98(0.79 to 1.23)	0.99(0.79 to 1.24)	1.00(0.80 to 1.25)
Drug use disorder	After pregnancy	50-51	1.19(0.81 to 1.76)	1.21(0.82 to 1.78)	1.22(0.83 to 1.79)
ADHD	During pregnancy	0-4	1.07(0.87 to 1.32)	1.08(0.87 to 1.33)	1.08(0.88 to 1.34)
ADHD	During pregnancy	5-9	0.87(0.70 to 1.09)	0.87(0.70 to 1.09)	0.88(0.70 to 1.10)
ADHD	During pregnancy	10-14	0.98(0.79 to 1.21)	0.98(0.79 to 1.22)	0.99(0.80 to 1.22)
ADHD	During pregnancy	15-19	1.13(0.91 to 1.41)	1.14(0.91 to 1.41)	1.14(0.92 to 1.42)
ADHD	During pregnancy	20-24	1.08(0.87 to 1.34)	1.08(0.87 to 1.34)	1.09(0.88 to 1.35)
ADHD	During pregnancy	25-29	0.97(0.79 to 1.19)	0.98(0.80 to 1.20)	0.98(0.80 to 1.20)
ADHD	During pregnancy	30-34	1.09(0.88 to 1.36)	1.10(0.88 to 1.37)	1.10(0.89 to 1.37)
ADHD	During pregnancy	35-39	1.59(1.24 to 2.03)	1.60(1.25 to 2.05)	1.61(1.26 to 2.06)
ADHD	After pregnancy	0-4	0.94(0.75 to 1.16)	0.94(0.76 to 1.17)	0.95(0.77 to 1.18)
ADHD	After pregnancy	5-9	1.14(0.93 to 1.41)	1.15(0.94 to 1.42)	1.16(0.94 to 1.43)
ADHD	After pregnancy	10-14	1.06(0.86 to 1.31)	1.07(0.87 to 1.32)	1.08(0.88 to 1.34)
ADHD	After pregnancy	15-19	1.23(1.00 to 1.53)	1.24(1.00 to 1.54)	1.26(1.02 to 1.56)
ADHD	After pregnancy	20-24	1.08(0.87 to 1.35)	1.09(0.88 to 1.36)	1.11(0.89 to 1.37)
ADHD	After pregnancy	25-29	0.88(0.71 to 1.09)	0.89(0.72 to 1.10)	0.90(0.73 to 1.11)
ADHD	After pregnancy	30-34	1.12(0.90 to 1.39)	1.13(0.91 to 1.41)	1.14(0.92 to 1.42)
ADHD	After pregnancy	35-39	1.60(1.26 to 2.03)	1.61(1.27 to 2.05)	1.63(1.28 to 2.07)
ADHD	After pregnancy	40-44	1.04(0.84 to 1.28)	1.05(0.85 to 1.29)	1.06(0.86 to 1.31)
ADHD	After pregnancy	45-49	1.02(0.83 to 1.26)	1.03(0.84 to 1.26)	1.04(0.85 to 1.28)
ADHD	After pregnancy	50-51	1.31(0.94 to 1.82)	1.32(0.95 to 1.84)	1.33(0.96 to 1.86)
Bipolar disorder	During pregnancy	0-4	1.28(0.84 to 1.95)	1.28(0.84 to 1.95)	1.28(0.84 to 1.95)
Bipolar disorder	During pregnancy	5-9	0.77(0.50 to 1.18)	0.77(0.50 to 1.18)	0.77(0.50 to 1.18)
Bipolar disorder	During pregnancy	10-14	1.19(0.79 to 1.78)	1.19(0.79 to 1.78)	1.19(0.79 to 1.78)
Bipolar disorder	During pregnancy	15-19	0.84(0.55 to 1.27)	0.84(0.55 to 1.27)	0.84(0.55 to 1.27)
Bipolar disorder	During pregnancy	20-24	0.59(0.35 to 0.97)	0.59(0.35 to 0.97)	0.59(0.35 to 0.97)
Bipolar disorder	During pregnancy	25-29	0.76(0.49 to 1.16)	0.76(0.49 to 1.16)	0.76(0.50 to 1.16)
Bipolar disorder	During pregnancy	30-34	0.76(0.49 to 1.17)	0.76(0.49 to 1.17)	0.76(0.49 to 1.17)
Bipolar disorder	During pregnancy	35-39	1.11(0.69 to 1.77)	1.11(0.70 to 1.78)	1.11(0.70 to 1.78)
Bipolar disorder	After pregnancy	0-4	1.10(0.72 to 1.70)	1.10(0.72 to 1.70)	1.11(0.72 to 1.71)
Bipolar disorder	After pregnancy	5-9	1.15(0.78 to 1.70)	1.15(0.78 to 1.70)	1.16(0.78 to 1.71)
Bipolar disorder	After pregnancy	10-14	1.08(0.71 to 1.63)	1.08(0.71 to 1.63)	1.08(0.71 to 1.64)
Bipolar disorder	After pregnancy	15-19	1.11(0.76 to 1.64)	1.11(0.76 to 1.64)	1.12(0.76 to 1.65)
Bipolar disorder	After pregnancy	20-24	1.61(1.09 to 2.38)	1.61(1.09 to 2.38)	1.62(1.10 to 2.39)
Bipolar disorder	After pregnancy	25-29	1.13(0.77 to 1.66)	1.13(0.77 to 1.66)	1.13(0.77 to 1.67)
Bipolar disorder	After pregnancy	30-34	1.03(0.69 to 1.55)	1.03(0.69 to 1.55)	1.04(0.69 to 1.56)
Bipolar disorder	After pregnancy	35-39	2.09(1.40 to 3.12)	2.10(1.41 to 3.13)	2.11(1.42 to 3.15)
Bipolar disorder	After pregnancy	40-44	1.23(0.82 to 1.86)	1.23(0.82 to 1.85)	1.24(0.83 to 1.86)
Bipolar disorder	After pregnancy	45-49	1.80(1.17 to 2.77)	1.80(1.17 to 2.77)	1.81(1.17 to 2.78)
Bipolar disorder	After pregnancy	50-51	1.97(1.01 to 3.86)	1.97(1.01 to 3.85)	1.99(1.02 to 3.88)
Psychosis	During pregnancy	0-4	1.00(0.62 to 1.61)	1.00(0.62 to 1.61)	1.00(0.62 to 1.61)
Psychosis	During pregnancy	5-9	0.93(0.61 to 1.43)	0.93(0.61 to 1.43)	0.93(0.61 to 1.43)
Psychosis	During pregnancy	10-14	1.70(1.06 to 2.74)	1.71(1.06 to 2.74)	1.71(1.06 to 2.75)
Psychosis	During pregnancy	15-19	1.06(0.67 to 1.67)	1.06(0.67 to 1.67)	1.06(0.67 to 1.67)

© 2026 Xiang N et al. *JAMA Network Open*.

Psychosis	During pregnancy	20-24	1.18(0.75 to 1.86)	1.18(0.75 to 1.86)	1.18(0.75 to 1.86)
Psychosis	During pregnancy	25-29	0.92(0.58 to 1.47)	0.92(0.58 to 1.47)	0.92(0.58 to 1.47)
Psychosis	During pregnancy	30-34	1.10(0.69 to 1.76)	1.10(0.69 to 1.76)	1.11(0.69 to 1.76)
Psychosis	During pregnancy	35-39	0.90(0.55 to 1.49)	0.90(0.55 to 1.50)	0.91(0.55 to 1.50)
Psychosis	After pregnancy	0-4	1.06(0.66 to 1.69)	1.06(0.66 to 1.70)	1.07(0.67 to 1.70)
Psychosis	After pregnancy	5-9	0.91(0.59 to 1.40)	0.91(0.59 to 1.40)	0.92(0.60 to 1.41)
Psychosis	After pregnancy	10-14	0.97(0.57 to 1.66)	0.97(0.57 to 1.67)	0.98(0.57 to 1.67)
Psychosis	After pregnancy	15-19	1.21(0.78 to 1.88)	1.21(0.78 to 1.89)	1.22(0.78 to 1.90)
Psychosis	After pregnancy	20-24	0.99(0.61 to 1.59)	0.99(0.61 to 1.60)	1.00(0.62 to 1.61)
Psychosis	After pregnancy	25-29	0.86(0.53 to 1.38)	0.87(0.54 to 1.40)	0.87(0.54 to 1.40)
Psychosis	After pregnancy	30-34	1.55(1.01 to 2.39)	1.57(1.02 to 2.42)	1.58(1.03 to 2.44)
Psychosis	After pregnancy	35-39	1.08(0.68 to 1.72)	1.09(0.69 to 1.74)	1.10(0.69 to 1.75)
Psychosis	After pregnancy	40-44	1.12(0.70 to 1.80)	1.14(0.71 to 1.82)	1.14(0.71 to 1.82)
Psychosis	After pregnancy	45-49	1.30(0.80 to 2.13)	1.32(0.81 to 2.15)	1.33(0.81 to 2.17)
Psychosis	After pregnancy	50-51	2.01(0.86 to 4.70)	2.03(0.87 to 4.74)	2.05(0.88 to 4.78)

*ADHD, attention deficit hyperactivity disorder.

* The incidence rate ratio was estimated by every 5 weeks, by comparing the incidence in each interval during and after pregnancy with that before pregnancy. Model 1 was adjusted for age and calendar year at childbirth and week at follow-up. Model 2 was additionally adjusted for country of birth, region of residence and education before pregnancy, season at childbirth, and income before pregnancy. Model 3 was additionally adjusted for civil status during pregnancy, multiple gestation, number of children, and history of psychiatric disorders.

© 2026 Xiang N et al. *JAMA Network Open*.

eTable 8. Incidence Rate Ratios of Any Paternal Psychiatric Disorder and 9 Type-Specific Disorders During and After Pregnancy, Including First Childbirths Only

Psychiatric disorders	Phase	Weeks	Incidence rate differences per 1000 person-years		
			Model 1	Model 2	Model 3
Any psychiatric disorder	During pregnancy	0-4	0.89(0.81 to 0.98)	0.90(0.82 to 0.98)	0.90(0.82 to 0.98)
Any psychiatric disorder	During pregnancy	5-9	0.88(0.80 to 0.97)	0.88(0.80 to 0.96)	0.88(0.80 to 0.97)
Any psychiatric disorder	During pregnancy	10-14	0.93(0.85 to 1.02)	0.93(0.85 to 1.02)	0.93(0.85 to 1.02)
Any psychiatric disorder	During pregnancy	15-19	0.87(0.79 to 0.95)	0.87(0.79 to 0.95)	0.87(0.79 to 0.95)
Any psychiatric disorder	During pregnancy	20-24	0.97(0.89 to 1.07)	0.98(0.89 to 1.07)	0.98(0.89 to 1.07)
Any psychiatric disorder	During pregnancy	25-29	0.90(0.82 to 0.99)	0.91(0.82 to 1.00)	0.91(0.83 to 1.00)
Any psychiatric disorder	During pregnancy	30-34	0.86(0.78 to 0.94)	0.87(0.79 to 0.95)	0.87(0.79 to 0.95)
Any psychiatric disorder	During pregnancy	35-39	0.80(0.73 to 0.88)	0.80(0.73 to 0.89)	0.81(0.73 to 0.89)
Any psychiatric disorder	After pregnancy	0-4	0.80(0.73 to 0.88)	0.80(0.73 to 0.88)	0.81(0.74 to 0.89)
Any psychiatric disorder	After pregnancy	5-9	0.86(0.79 to 0.94)	0.86(0.78 to 0.94)	0.86(0.79 to 0.95)
Any psychiatric disorder	After pregnancy	10-14	0.88(0.80 to 0.96)	0.88(0.80 to 0.96)	0.88(0.80 to 0.97)
Any psychiatric disorder	After pregnancy	15-19	0.93(0.85 to 1.01)	0.93(0.85 to 1.02)	0.93(0.85 to 1.02)
Any psychiatric disorder	After pregnancy	20-24	0.96(0.87 to 1.05)	0.96(0.88 to 1.06)	0.97(0.88 to 1.06)
Any psychiatric disorder	After pregnancy	25-29	0.97(0.88 to 1.06)	0.98(0.89 to 1.07)	0.98(0.89 to 1.08)
Any psychiatric disorder	After pregnancy	30-34	0.89(0.81 to 0.97)	0.90(0.82 to 0.99)	0.90(0.82 to 0.99)
Any psychiatric disorder	After pregnancy	35-39	0.95(0.86 to 1.04)	0.95(0.87 to 1.05)	0.96(0.87 to 1.05)
Any psychiatric disorder	After pregnancy	40-44	0.88(0.80 to 0.96)	0.88(0.81 to 0.97)	0.89(0.81 to 0.97)
Any psychiatric disorder	After pregnancy	45-49	1.04(0.94 to 1.14)	1.04(0.95 to 1.15)	1.05(0.95 to 1.15)
Any psychiatric disorder	After pregnancy	50-51	0.94(0.81 to 1.09)	0.94(0.81 to 1.09)	0.95(0.81 to 1.10)
Depression	During pregnancy	0-4	0.81(0.70 to 0.95)	0.82(0.70 to 0.95)	0.83(0.71 to 0.96)
Depression	During pregnancy	5-9	0.82(0.71 to 0.96)	0.83(0.71 to 0.96)	0.83(0.72 to 0.97)
Depression	During pregnancy	10-14	0.82(0.71 to 0.96)	0.83(0.71 to 0.97)	0.83(0.71 to 0.97)
Depression	During pregnancy	15-19	0.93(0.79 to 1.08)	0.94(0.80 to 1.09)	0.94(0.81 to 1.10)
Depression	During pregnancy	20-24	1.03(0.87 to 1.20)	1.03(0.88 to 1.21)	1.04(0.88 to 1.22)
Depression	During pregnancy	25-29	0.76(0.65 to 0.90)	0.77(0.65 to 0.90)	0.77(0.65 to 0.91)
Depression	During pregnancy	30-34	0.73(0.62 to 0.85)	0.74(0.63 to 0.87)	0.74(0.63 to 0.87)
Depression	During pregnancy	35-39	0.68(0.57 to 0.80)	0.68(0.58 to 0.81)	0.69(0.58 to 0.81)
Depression	After pregnancy	0-4	0.66(0.56 to 0.77)	0.67(0.57 to 0.78)	0.68(0.57 to 0.79)
Depression	After pregnancy	5-9	0.75(0.64 to 0.87)	0.76(0.65 to 0.88)	0.76(0.65 to 0.89)
Depression	After pregnancy	10-14	0.93(0.80 to 1.08)	0.94(0.81 to 1.09)	0.95(0.82 to 1.10)
Depression	After pregnancy	15-19	1.11(0.95 to 1.28)	1.13(0.97 to 1.31)	1.13(0.98 to 1.32)
Depression	After pregnancy	20-24	1.07(0.91 to 1.26)	1.08(0.92 to 1.27)	1.09(0.93 to 1.28)
Depression	After pregnancy	25-29	1.05(0.90 to 1.22)	1.06(0.91 to 1.23)	1.07(0.92 to 1.24)
Depression	After pregnancy	30-34	1.02(0.88 to 1.18)	1.04(0.89 to 1.20)	1.04(0.90 to 1.21)
Depression	After pregnancy	35-39	1.01(0.87 to 1.17)	1.02(0.88 to 1.19)	1.03(0.89 to 1.19)
Depression	After pregnancy	40-44	1.09(0.94 to 1.27)	1.10(0.95 to 1.28)	1.11(0.96 to 1.28)
Depression	After pregnancy	45-49	1.19(1.02 to 1.40)	1.21(1.03 to 1.42)	1.22(1.04 to 1.43)
Depression	After pregnancy	50-51	1.58(1.22 to 2.05)	1.57(1.21 to 2.04)	1.58(1.22 to 2.05)
Anxiety	During pregnancy	0-4	1.03(0.89 to 1.18)	1.03(0.90 to 1.18)	1.04(0.91 to 1.19)
Anxiety	During pregnancy	5-9	0.92(0.80 to 1.06)	0.92(0.80 to 1.06)	0.93(0.81 to 1.07)

© 2026 Xiang N et al. *JAMA Network Open*.

Anxiety	During pregnancy	10-14	0.94(0.82 to 1.08)	0.95(0.82 to 1.09)	0.95(0.83 to 1.10)
Anxiety	During pregnancy	15-19	0.92(0.80 to 1.06)	0.92(0.80 to 1.06)	0.92(0.80 to 1.07)
Anxiety	During pregnancy	20-24	1.20(1.04 to 1.38)	1.20(1.05 to 1.38)	1.21(1.05 to 1.39)
Anxiety	During pregnancy	25-29	1.03(0.90 to 1.18)	1.04(0.90 to 1.19)	1.04(0.91 to 1.20)
Anxiety	During pregnancy	30-34	1.13(0.98 to 1.29)	1.14(0.99 to 1.31)	1.15(1.00 to 1.32)
Anxiety	During pregnancy	35-39	0.88(0.76 to 1.01)	0.89(0.77 to 1.02)	0.89(0.77 to 1.03)
Anxiety	After pregnancy	0-4	0.92(0.80 to 1.06)	0.94(0.81 to 1.08)	0.95(0.82 to 1.09)
Anxiety	After pregnancy	5-9	0.93(0.81 to 1.07)	0.94(0.82 to 1.08)	0.95(0.83 to 1.10)
Anxiety	After pregnancy	10-14	0.88(0.77 to 1.02)	0.90(0.78 to 1.03)	0.91(0.78 to 1.04)
Anxiety	After pregnancy	15-19	1.07(0.93 to 1.23)	1.07(0.94 to 1.23)	1.09(0.95 to 1.25)
Anxiety	After pregnancy	20-24	1.12(0.97 to 1.29)	1.13(0.98 to 1.30)	1.14(0.99 to 1.31)
Anxiety	After pregnancy	25-29	1.01(0.88 to 1.16)	1.02(0.89 to 1.18)	1.03(0.90 to 1.19)
Anxiety	After pregnancy	30-34	1.03(0.89 to 1.18)	1.04(0.90 to 1.20)	1.05(0.91 to 1.21)
Anxiety	After pregnancy	35-39	0.82(0.71 to 0.94)	0.83(0.72 to 0.95)	0.83(0.72 to 0.96)
Anxiety	After pregnancy	40-44	0.86(0.75 to 0.99)	0.88(0.76 to 1.01)	0.88(0.77 to 1.01)
Anxiety	After pregnancy	45-49	1.03(0.89 to 1.18)	1.04(0.90 to 1.20)	1.04(0.90 to 1.20)
Anxiety	After pregnancy	50-51	0.98(0.78 to 1.23)	0.99(0.79 to 1.24)	0.99(0.79 to 1.25)
Stress-related disorder	During pregnancy	0-4	0.99(0.84 to 1.16)	0.99(0.84 to 1.16)	0.99(0.85 to 1.17)
Stress-related disorder	During pregnancy	5-9	0.99(0.84 to 1.17)	0.99(0.84 to 1.17)	1.00(0.85 to 1.17)
Stress-related disorder	During pregnancy	10-14	1.11(0.94 to 1.30)	1.10(0.94 to 1.29)	1.11(0.95 to 1.30)
Stress-related disorder	During pregnancy	15-19	0.91(0.78 to 1.07)	0.92(0.79 to 1.08)	0.92(0.79 to 1.08)
Stress-related disorder	During pregnancy	20-24	0.97(0.83 to 1.13)	0.97(0.83 to 1.13)	0.98(0.84 to 1.14)
Stress-related disorder	During pregnancy	25-29	0.98(0.83 to 1.15)	0.98(0.84 to 1.15)	0.99(0.84 to 1.16)
Stress-related disorder	During pregnancy	30-34	0.86(0.73 to 1.00)	0.86(0.74 to 1.01)	0.87(0.74 to 1.01)
Stress-related disorder	During pregnancy	35-39	0.94(0.80 to 1.11)	0.94(0.80 to 1.11)	0.95(0.80 to 1.12)
Stress-related disorder	After pregnancy	0-4	1.24(1.07 to 1.44)	1.25(1.07 to 1.45)	1.26(1.08 to 1.47)
Stress-related disorder	After pregnancy	5-9	1.28(1.10 to 1.50)	1.29(1.11 to 1.50)	1.30(1.11 to 1.52)
Stress-related disorder	After pregnancy	10-14	1.19(1.02 to 1.40)	1.19(1.02 to 1.39)	1.20(1.03 to 1.41)
Stress-related disorder	After pregnancy	15-19	1.10(0.94 to 1.27)	1.11(0.96 to 1.29)	1.12(0.96 to 1.30)
Stress-related disorder	After pregnancy	20-24	1.15(0.99 to 1.33)	1.16(1.00 to 1.35)	1.17(1.01 to 1.35)
Stress-related disorder	After pregnancy	25-29	1.20(1.03 to 1.40)	1.22(1.04 to 1.42)	1.23(1.05 to 1.43)
Stress-related disorder	After pregnancy	30-34	1.02(0.87 to 1.19)	1.03(0.89 to 1.20)	1.04(0.89 to 1.21)
Stress-related disorder	After pregnancy	35-39	1.24(1.07 to 1.44)	1.25(1.08 to 1.45)	1.26(1.09 to 1.46)
Stress-related disorder	After pregnancy	40-44	1.00(0.86 to 1.17)	1.02(0.87 to 1.19)	1.02(0.87 to 1.19)
Stress-related disorder	After pregnancy	45-49	1.16(0.99 to 1.36)	1.18(1.01 to 1.38)	1.18(1.01 to 1.39)
Stress-related disorder	After pregnancy	50-51	1.02(0.79 to 1.32)	1.02(0.79 to 1.32)	1.02(0.79 to 1.33)
Alcohol use disorder	During pregnancy	0-4	0.60(0.48 to 0.76)	0.61(0.48 to 0.77)	0.61(0.49 to 0.77)
Alcohol use disorder	During pregnancy	5-9	0.74(0.59 to 0.93)	0.75(0.60 to 0.94)	0.75(0.60 to 0.94)
Alcohol use disorder	During pregnancy	10-14	0.78(0.61 to 0.98)	0.78(0.62 to 0.98)	0.78(0.62 to 0.99)
Alcohol use disorder	During pregnancy	15-19	0.64(0.51 to 0.81)	0.65(0.51 to 0.82)	0.65(0.52 to 0.83)
Alcohol use disorder	During pregnancy	20-24	0.59(0.46 to 0.74)	0.59(0.46 to 0.74)	0.59(0.47 to 0.75)
Alcohol use disorder	During pregnancy	25-29	0.75(0.58 to 0.96)	0.75(0.58 to 0.96)	0.75(0.59 to 0.97)
Alcohol use disorder	During pregnancy	30-34	0.68(0.53 to 0.87)	0.69(0.54 to 0.88)	0.69(0.54 to 0.89)
Alcohol use disorder	During pregnancy	35-39	0.57(0.44 to 0.75)	0.58(0.44 to 0.76)	0.58(0.45 to 0.76)
Alcohol use disorder	After pregnancy	0-4	0.43(0.34 to 0.56)	0.44(0.34 to 0.57)	0.45(0.35 to 0.58)

© 2026 Xiang N et al. *JAMA Network Open*.

Alcohol use disorder	After pregnancy	5-9	0.57(0.45 to 0.73)	0.58(0.45 to 0.74)	0.59(0.46 to 0.75)
Alcohol use disorder	After pregnancy	10-14	0.65(0.51 to 0.83)	0.66(0.52 to 0.84)	0.67(0.52 to 0.85)
Alcohol use disorder	After pregnancy	15-19	0.64(0.50 to 0.80)	0.65(0.51 to 0.82)	0.65(0.52 to 0.82)
Alcohol use disorder	After pregnancy	20-24	0.61(0.48 to 0.77)	0.61(0.48 to 0.77)	0.62(0.49 to 0.78)
Alcohol use disorder	After pregnancy	25-29	0.89(0.70 to 1.13)	0.90(0.71 to 1.14)	0.91(0.71 to 1.15)
Alcohol use disorder	After pregnancy	30-34	0.75(0.59 to 0.96)	0.76(0.60 to 0.97)	0.77(0.60 to 0.98)
Alcohol use disorder	After pregnancy	35-39	0.78(0.62 to 0.99)	0.79(0.63 to 1.00)	0.80(0.63 to 1.01)
Alcohol use disorder	After pregnancy	40-44	0.85(0.67 to 1.07)	0.86(0.68 to 1.08)	0.86(0.68 to 1.09)
Alcohol use disorder	After pregnancy	45-49	0.81(0.64 to 1.03)	0.82(0.65 to 1.04)	0.83(0.65 to 1.05)
Alcohol use disorder	After pregnancy	50-51	0.82(0.58 to 1.15)	0.82(0.58 to 1.16)	0.82(0.58 to 1.16)
Tobacco use disorder	During pregnancy	0-4	1.15(0.68 to 1.93)	1.14(0.67 to 1.92)	1.14(0.68 to 1.93)
Tobacco use disorder	During pregnancy	5-9	1.25(0.78 to 2.01)	1.22(0.76 to 1.97)	1.23(0.76 to 1.98)
Tobacco use disorder	During pregnancy	10-14	0.99(0.63 to 1.55)	0.98(0.62 to 1.54)	0.98(0.62 to 1.54)
Tobacco use disorder	During pregnancy	15-19	0.85(0.52 to 1.39)	0.85(0.52 to 1.40)	0.86(0.52 to 1.40)
Tobacco use disorder	During pregnancy	20-24	1.15(0.68 to 1.93)	1.15(0.68 to 1.94)	1.15(0.68 to 1.94)
Tobacco use disorder	During pregnancy	25-29	1.26(0.76 to 2.11)	1.24(0.74 to 2.07)	1.25(0.75 to 2.08)
Tobacco use disorder	During pregnancy	30-34	1.24(0.77 to 2.00)	1.23(0.76 to 1.98)	1.23(0.76 to 1.99)
Tobacco use disorder	During pregnancy	35-39	1.00(0.63 to 1.58)	1.01(0.63 to 1.60)	1.01(0.64 to 1.60)
Tobacco use disorder	After pregnancy	0-4	1.08(0.64 to 1.83)	1.08(0.64 to 1.83)	1.09(0.64 to 1.84)
Tobacco use disorder	After pregnancy	5-9	1.13(0.70 to 1.84)	1.10(0.68 to 1.79)	1.11(0.68 to 1.80)
Tobacco use disorder	After pregnancy	10-14	0.64(0.39 to 1.06)	0.64(0.38 to 1.06)	0.64(0.38 to 1.06)
Tobacco use disorder	After pregnancy	15-19	0.73(0.44 to 1.22)	0.74(0.44 to 1.24)	0.75(0.45 to 1.24)
Tobacco use disorder	After pregnancy	20-24	1.27(0.76 to 2.11)	1.28(0.77 to 2.14)	1.28(0.77 to 2.14)
Tobacco use disorder	After pregnancy	25-29	1.13(0.67 to 1.92)	1.12(0.66 to 1.90)	1.13(0.67 to 1.91)
Tobacco use disorder	After pregnancy	30-34	1.16(0.71 to 1.89)	1.16(0.71 to 1.90)	1.17(0.72 to 1.91)
Tobacco use disorder	After pregnancy	35-39	1.12(0.73 to 1.73)	1.14(0.74 to 1.76)	1.15(0.74 to 1.77)
Tobacco use disorder	After pregnancy	40-44	1.29(0.86 to 1.95)	1.32(0.87 to 1.99)	1.32(0.88 to 2.00)
Tobacco use disorder	After pregnancy	45-49	1.44(0.92 to 2.26)	1.46(0.93 to 2.28)	1.45(0.93 to 2.28)
Tobacco use disorder	After pregnancy	50-51	1.65(0.78 to 3.48)	1.64(0.77 to 3.48)	1.64(0.78 to 3.49)
Drug use disorder	During pregnancy	0-4	0.65(0.50 to 0.85)	0.66(0.51 to 0.85)	0.67(0.51 to 0.86)
Drug use disorder	During pregnancy	5-9	0.78(0.60 to 1.01)	0.77(0.59 to 1.00)	0.79(0.61 to 1.02)
Drug use disorder	During pregnancy	10-14	0.79(0.61 to 1.02)	0.79(0.61 to 1.03)	0.80(0.62 to 1.04)
Drug use disorder	During pregnancy	15-19	0.76(0.59 to 0.96)	0.76(0.60 to 0.97)	0.77(0.61 to 0.99)
Drug use disorder	During pregnancy	20-24	0.75(0.58 to 0.97)	0.75(0.58 to 0.97)	0.76(0.59 to 0.99)
Drug use disorder	During pregnancy	25-29	0.71(0.55 to 0.92)	0.71(0.55 to 0.92)	0.72(0.56 to 0.93)
Drug use disorder	During pregnancy	30-34	0.69(0.52 to 0.90)	0.70(0.54 to 0.92)	0.71(0.54 to 0.92)
Drug use disorder	During pregnancy	35-39	0.80(0.60 to 1.06)	0.81(0.61 to 1.08)	0.82(0.61 to 1.10)
Drug use disorder	After pregnancy	0-4	0.47(0.35 to 0.63)	0.48(0.36 to 0.64)	0.49(0.37 to 0.65)
Drug use disorder	After pregnancy	5-9	0.84(0.65 to 1.08)	0.84(0.65 to 1.08)	0.86(0.67 to 1.11)
Drug use disorder	After pregnancy	10-14	0.75(0.57 to 0.97)	0.76(0.58 to 0.98)	0.77(0.60 to 1.00)
Drug use disorder	After pregnancy	15-19	0.61(0.47 to 0.79)	0.62(0.48 to 0.81)	0.63(0.49 to 0.82)
Drug use disorder	After pregnancy	20-24	0.86(0.67 to 1.11)	0.87(0.68 to 1.12)	0.89(0.69 to 1.14)
Drug use disorder	After pregnancy	25-29	0.55(0.42 to 0.72)	0.55(0.42 to 0.73)	0.56(0.42 to 0.74)
Drug use disorder	After pregnancy	30-34	0.76(0.59 to 0.99)	0.79(0.60 to 1.02)	0.80(0.61 to 1.04)
Drug use disorder	After pregnancy	35-39	0.78(0.59 to 1.04)	0.80(0.61 to 1.06)	0.81(0.61 to 1.07)

© 2026 Xiang N et al. *JAMA Network Open*.

Drug use disorder	After pregnancy	40-44	0.95(0.73 to 1.23)	0.97(0.74 to 1.26)	0.98(0.75 to 1.27)
Drug use disorder	After pregnancy	45-49	0.98(0.76 to 1.25)	1.00(0.78 to 1.28)	1.01(0.78 to 1.29)
Drug use disorder	After pregnancy	50-51	1.05(0.70 to 1.60)	1.06(0.70 to 1.61)	1.07(0.70 to 1.62)
ADHD	During pregnancy	0-4	0.98(0.76 to 1.27)	0.99(0.76 to 1.28)	1.01(0.78 to 1.30)
ADHD	During pregnancy	5-9	0.79(0.60 to 1.05)	0.80(0.61 to 1.06)	0.81(0.62 to 1.07)
ADHD	During pregnancy	10-14	0.86(0.66 to 1.12)	0.87(0.67 to 1.13)	0.88(0.68 to 1.14)
ADHD	During pregnancy	15-19	1.20(0.91 to 1.56)	1.21(0.93 to 1.58)	1.23(0.94 to 1.60)
ADHD	During pregnancy	20-24	1.05(0.81 to 1.36)	1.07(0.82 to 1.38)	1.08(0.83 to 1.40)
ADHD	During pregnancy	25-29	0.97(0.75 to 1.26)	0.98(0.76 to 1.28)	0.99(0.76 to 1.29)
ADHD	During pregnancy	30-34	1.04(0.80 to 1.36)	1.06(0.81 to 1.38)	1.06(0.82 to 1.38)
ADHD	During pregnancy	35-39	1.63(1.21 to 2.21)	1.67(1.23 to 2.26)	1.68(1.24 to 2.27)
ADHD	After pregnancy	0-4	0.84(0.64 to 1.10)	0.85(0.65 to 1.11)	0.87(0.67 to 1.14)
ADHD	After pregnancy	5-9	1.08(0.84 to 1.39)	1.10(0.85 to 1.41)	1.11(0.86 to 1.43)
ADHD	After pregnancy	10-14	1.10(0.86 to 1.40)	1.12(0.87 to 1.43)	1.14(0.89 to 1.46)
ADHD	After pregnancy	15-19	1.24(0.95 to 1.61)	1.27(0.97 to 1.65)	1.29(0.99 to 1.68)
ADHD	After pregnancy	20-24	1.06(0.82 to 1.37)	1.09(0.84 to 1.41)	1.11(0.85 to 1.44)
ADHD	After pregnancy	25-29	0.92(0.71 to 1.20)	0.94(0.72 to 1.23)	0.96(0.73 to 1.25)
ADHD	After pregnancy	30-34	1.10(0.85 to 1.43)	1.12(0.86 to 1.46)	1.14(0.88 to 1.48)
ADHD	After pregnancy	35-39	1.64(1.22 to 2.21)	1.68(1.25 to 2.26)	1.70(1.27 to 2.29)
ADHD	After pregnancy	40-44	0.98(0.76 to 1.26)	1.00(0.78 to 1.29)	1.01(0.79 to 1.30)
ADHD	After pregnancy	45-49	1.09(0.84 to 1.41)	1.11(0.86 to 1.43)	1.12(0.86 to 1.44)
ADHD	After pregnancy	50-51	1.74(1.17 to 2.59)	1.73(1.16 to 2.58)	1.75(1.18 to 2.60)
Bipolar disorder	During pregnancy	0-4	1.07(0.64 to 1.78)	1.07(0.64 to 1.78)	1.08(0.65 to 1.80)
Bipolar disorder	During pregnancy	5-9	0.96(0.57 to 1.63)	0.97(0.57 to 1.64)	0.98(0.58 to 1.65)
Bipolar disorder	During pregnancy	10-14	1.18(0.71 to 1.96)	1.17(0.70 to 1.94)	1.17(0.71 to 1.95)
Bipolar disorder	During pregnancy	15-19	0.87(0.51 to 1.47)	0.86(0.51 to 1.47)	0.87(0.51 to 1.48)
Bipolar disorder	During pregnancy	20-24	0.56(0.29 to 1.09)	0.58(0.30 to 1.12)	0.58(0.30 to 1.12)
Bipolar disorder	During pregnancy	25-29	0.81(0.50 to 1.31)	0.82(0.51 to 1.32)	0.82(0.51 to 1.32)
Bipolar disorder	During pregnancy	30-34	0.80(0.48 to 1.33)	0.81(0.49 to 1.35)	0.81(0.49 to 1.35)
Bipolar disorder	During pregnancy	35-39	1.01(0.60 to 1.70)	1.03(0.61 to 1.73)	1.03(0.61 to 1.73)
Bipolar disorder	After pregnancy	0-4	1.11(0.67 to 1.84)	1.12(0.68 to 1.86)	1.14(0.69 to 1.88)
Bipolar disorder	After pregnancy	5-9	1.15(0.70 to 1.90)	1.16(0.70 to 1.92)	1.17(0.71 to 1.93)
Bipolar disorder	After pregnancy	10-14	0.99(0.58 to 1.68)	0.97(0.57 to 1.66)	0.98(0.58 to 1.67)
Bipolar disorder	After pregnancy	15-19	1.46(0.91 to 2.33)	1.46(0.91 to 2.34)	1.48(0.92 to 2.36)
Bipolar disorder	After pregnancy	20-24	1.87(1.14 to 3.06)	1.93(1.18 to 3.16)	1.94(1.18 to 3.17)
Bipolar disorder	After pregnancy	25-29	1.07(0.68 to 1.67)	1.08(0.69 to 1.69)	1.08(0.69 to 1.69)
Bipolar disorder	After pregnancy	30-34	1.03(0.64 to 1.66)	1.05(0.65 to 1.69)	1.05(0.65 to 1.70)
Bipolar disorder	After pregnancy	35-39	1.80(1.15 to 2.82)	1.83(1.17 to 2.87)	1.84(1.18 to 2.88)
Bipolar disorder	After pregnancy	40-44	1.08(0.65 to 1.79)	1.10(0.67 to 1.82)	1.11(0.67 to 1.83)
Bipolar disorder	After pregnancy	45-49	1.67(1.01 to 2.75)	1.66(1.01 to 2.75)	1.67(1.01 to 2.76)
Bipolar disorder	After pregnancy	50-51	2.31(1.00 to 5.32)	2.31(1.00 to 5.32)	2.32(1.01 to 5.35)
Psychosis	During pregnancy	0-4	0.88(0.50 to 1.58)	0.87(0.49 to 1.56)	0.89(0.50 to 1.59)
Psychosis	During pregnancy	5-9	1.25(0.73 to 2.13)	1.21(0.71 to 2.07)	1.23(0.72 to 2.09)
Psychosis	During pregnancy	10-14	1.71(1.02 to 2.89)	1.65(0.98 to 2.79)	1.68(0.99 to 2.83)
Psychosis	During pregnancy	15-19	1.08(0.64 to 1.83)	1.05(0.62 to 1.78)	1.06(0.63 to 1.80)

© 2026 Xiang N et al. *JAMA Network Open*.

Psychosis	During pregnancy	20-24	1.14(0.69 to 1.88)	1.09(0.66 to 1.81)	1.11(0.67 to 1.84)
Psychosis	During pregnancy	25-29	0.78(0.46 to 1.32)	0.79(0.47 to 1.34)	0.79(0.47 to 1.35)
Psychosis	During pregnancy	30-34	1.11(0.63 to 1.94)	1.11(0.63 to 1.94)	1.12(0.64 to 1.96)
Psychosis	During pregnancy	35-39	0.97(0.56 to 1.67)	0.93(0.54 to 1.62)	0.94(0.54 to 1.62)
Psychosis	After pregnancy	0-4	1.10(0.64 to 1.90)	1.10(0.64 to 1.90)	1.13(0.65 to 1.94)
Psychosis	After pregnancy	5-9	1.31(0.77 to 2.21)	1.25(0.74 to 2.12)	1.27(0.75 to 2.15)
Psychosis	After pregnancy	10-14	0.96(0.53 to 1.73)	0.92(0.51 to 1.66)	0.93(0.52 to 1.69)
Psychosis	After pregnancy	15-19	1.37(0.83 to 2.27)	1.34(0.81 to 2.21)	1.36(0.82 to 2.25)
Psychosis	After pregnancy	20-24	0.94(0.55 to 1.59)	0.89(0.52 to 1.52)	0.91(0.54 to 1.55)
Psychosis	After pregnancy	25-29	0.57(0.32 to 1.02)	0.59(0.33 to 1.05)	0.59(0.33 to 1.06)
Psychosis	After pregnancy	30-34	1.96(1.19 to 3.24)	1.98(1.20 to 3.28)	2.01(1.21 to 3.32)
Psychosis	After pregnancy	35-39	1.31(0.80 to 2.15)	1.28(0.78 to 2.11)	1.30(0.79 to 2.13)
Psychosis	After pregnancy	40-44	1.21(0.70 to 2.10)	1.20(0.70 to 2.08)	1.21(0.70 to 2.09)
Psychosis	After pregnancy	45-49	1.31(0.75 to 2.31)	1.33(0.76 to 2.34)	1.34(0.76 to 2.35)

*ADHD, attention deficit hyperactivity disorder.

* The incidence rate ratio was estimated by every 5 weeks, by comparing the incidence in each interval during and after pregnancy with that before pregnancy. Model 1 was adjusted for age and calendar year at childbirth and week at follow-up. Model 2 was additionally adjusted for country of birth, region of residence and education before pregnancy, season at childbirth, and income before pregnancy. Model 3 was additionally adjusted for civil status during pregnancy, multiple gestation, number of children, and history of psychiatric disorders.

© 2026 Xiang N et al. *JAMA Network Open*.

eTable 9. Standardized Incidence Rates of Any Paternal Psychiatric Disorder Before, During, and After Pregnancy by Weeks, Stratified by Year of Childbirth, Education Level, Country of Birth, and Number of Children

Stratified variables	Strata	Phase	Week	Standardized incidence rate per 1000 person-years (95%CI)
Year of childbirth	2003-2009	Before pregnancy	1	6.53(4.94 to 8.13)
Year of childbirth	2003-2009	Before pregnancy	2	6.44(4.86 to 8.02)
Year of childbirth	2003-2009	Before pregnancy	3	6.37(4.80 to 7.93)
Year of childbirth	2003-2009	Before pregnancy	4	6.31(4.75 to 7.87)
Year of childbirth	2003-2009	Before pregnancy	5	6.27(4.72 to 7.82)
Year of childbirth	2003-2009	Before pregnancy	6	6.24(4.69 to 7.79)
Year of childbirth	2003-2009	Before pregnancy	7	6.21(4.67 to 7.76)
Year of childbirth	2003-2009	Before pregnancy	8	6.19(4.65 to 7.73)
Year of childbirth	2003-2009	Before pregnancy	9	6.17(4.63 to 7.70)
Year of childbirth	2003-2009	Before pregnancy	10	6.14(4.62 to 7.67)
Year of childbirth	2003-2009	Before pregnancy	11	6.12(4.59 to 7.64)
Year of childbirth	2003-2009	Before pregnancy	12	6.09(4.57 to 7.61)
Year of childbirth	2003-2009	Before pregnancy	13	6.06(4.55 to 7.57)
Year of childbirth	2003-2009	Before pregnancy	14	6.03(4.52 to 7.53)
Year of childbirth	2003-2009	Before pregnancy	15	6.00(4.50 to 7.50)
Year of childbirth	2003-2009	Before pregnancy	16	5.97(4.47 to 7.46)
Year of childbirth	2003-2009	Before pregnancy	17	5.94(4.45 to 7.43)
Year of childbirth	2003-2009	Before pregnancy	18	5.91(4.43 to 7.39)
Year of childbirth	2003-2009	Before pregnancy	19	5.89(4.41 to 7.37)
Year of childbirth	2003-2009	Before pregnancy	20	5.87(4.40 to 7.35)
Year of childbirth	2003-2009	Before pregnancy	21	5.86(4.39 to 7.33)
Year of childbirth	2003-2009	Before pregnancy	22	5.85(4.38 to 7.31)
Year of childbirth	2003-2009	Before pregnancy	23	5.84(4.38 to 7.30)
Year of childbirth	2003-2009	Before pregnancy	24	5.84(4.37 to 7.30)
Year of childbirth	2003-2009	Before pregnancy	25	5.83(4.37 to 7.29)
Year of childbirth	2003-2009	Before pregnancy	26	5.83(4.38 to 7.29)
Year of childbirth	2003-2009	Before pregnancy	27	5.84(4.38 to 7.29)
Year of childbirth	2003-2009	Before pregnancy	28	5.84(4.38 to 7.29)
Year of childbirth	2003-2009	Before pregnancy	29	5.83(4.38 to 7.29)
Year of childbirth	2003-2009	Before pregnancy	30	5.83(4.38 to 7.28)
Year of childbirth	2003-2009	Before pregnancy	31	5.83(4.38 to 7.28)
Year of childbirth	2003-2009	Before pregnancy	32	5.82(4.38 to 7.27)
Year of childbirth	2003-2009	Before pregnancy	33	5.82(4.38 to 7.26)
Year of childbirth	2003-2009	Before pregnancy	34	5.81(4.37 to 7.25)
Year of childbirth	2003-2009	Before pregnancy	35	5.80(4.37 to 7.24)
Year of childbirth	2003-2009	Before pregnancy	36	5.80(4.37 to 7.23)
Year of childbirth	2003-2009	Before pregnancy	37	5.80(4.37 to 7.23)
Year of childbirth	2003-2009	Before pregnancy	38	5.80(4.37 to 7.22)

© 2026 Xiang N et al. *JAMA Network Open*.

Year of childbirth	2003-2009	Before pregnancy	39	5.79(4.37 to 7.22)
Year of childbirth	2003-2009	Before pregnancy	40	5.79(4.36 to 7.21)
Year of childbirth	2003-2009	Before pregnancy	41	5.78(4.36 to 7.20)
Year of childbirth	2003-2009	Before pregnancy	42	5.77(4.35 to 7.18)
Year of childbirth	2003-2009	Before pregnancy	43	5.75(4.34 to 7.16)
Year of childbirth	2003-2009	Before pregnancy	44	5.72(4.32 to 7.13)
Year of childbirth	2003-2009	Before pregnancy	45	5.68(4.29 to 7.08)
Year of childbirth	2003-2009	Before pregnancy	46	5.63(4.24 to 7.02)
Year of childbirth	2003-2009	Before pregnancy	47	5.57(4.19 to 6.95)
Year of childbirth	2003-2009	Before pregnancy	48	5.49(4.12 to 6.85)
Year of childbirth	2003-2009	Before pregnancy	49	5.39(4.04 to 6.74)
Year of childbirth	2003-2009	Before pregnancy	50	5.27(3.94 to 6.61)
Year of childbirth	2003-2009	Before pregnancy	51	5.14(3.82 to 6.45)
Year of childbirth	2003-2009	During pregnancy	1	5.35(4.05 to 6.66)
Year of childbirth	2003-2009	During pregnancy	2	5.37(4.06 to 6.67)
Year of childbirth	2003-2009	During pregnancy	3	5.39(4.08 to 6.69)
Year of childbirth	2003-2009	During pregnancy	4	5.40(4.09 to 6.71)
Year of childbirth	2003-2009	During pregnancy	5	5.41(4.10 to 6.72)
Year of childbirth	2003-2009	During pregnancy	6	5.42(4.11 to 6.73)
Year of childbirth	2003-2009	During pregnancy	7	5.43(4.12 to 6.74)
Year of childbirth	2003-2009	During pregnancy	8	5.44(4.13 to 6.76)
Year of childbirth	2003-2009	During pregnancy	9	5.46(4.14 to 6.77)
Year of childbirth	2003-2009	During pregnancy	10	5.47(4.15 to 6.78)
Year of childbirth	2003-2009	During pregnancy	11	5.48(4.16 to 6.79)
Year of childbirth	2003-2009	During pregnancy	12	5.48(4.17 to 6.80)
Year of childbirth	2003-2009	During pregnancy	13	5.48(4.17 to 6.80)
Year of childbirth	2003-2009	During pregnancy	14	5.48(4.16 to 6.79)
Year of childbirth	2003-2009	During pregnancy	15	5.47(4.15 to 6.78)
Year of childbirth	2003-2009	During pregnancy	16	5.45(4.14 to 6.77)
Year of childbirth	2003-2009	During pregnancy	17	5.44(4.13 to 6.75)
Year of childbirth	2003-2009	During pregnancy	18	5.42(4.11 to 6.73)
Year of childbirth	2003-2009	During pregnancy	19	5.40(4.09 to 6.70)
Year of childbirth	2003-2009	During pregnancy	20	5.38(4.07 to 6.68)
Year of childbirth	2003-2009	During pregnancy	21	5.35(4.05 to 6.65)
Year of childbirth	2003-2009	During pregnancy	22	5.32(4.03 to 6.62)
Year of childbirth	2003-2009	During pregnancy	23	5.30(4.00 to 6.59)
Year of childbirth	2003-2009	During pregnancy	24	5.27(3.98 to 6.56)
Year of childbirth	2003-2009	During pregnancy	25	5.23(3.95 to 6.52)
Year of childbirth	2003-2009	During pregnancy	26	5.19(3.91 to 6.48)
Year of childbirth	2003-2009	During pregnancy	27	5.15(3.86 to 6.44)
Year of childbirth	2003-2009	During pregnancy	28	5.09(3.79 to 6.39)
Year of childbirth	2003-2009	During pregnancy	29	5.03(3.72 to 6.34)
Year of childbirth	2003-2009	During pregnancy	30	4.96(3.63 to 6.29)
Year of childbirth	2003-2009	During pregnancy	31	4.89(3.54 to 6.25)

© 2026 Xiang N et al. *JAMA Network Open*.

Year of childbirth	2003-2009	During pregnancy	32	4.82(3.44 to 6.20)
Year of childbirth	2003-2009	During pregnancy	33	4.75(3.33 to 6.16)
Year of childbirth	2003-2009	During pregnancy	34	4.67(3.21 to 6.13)
Year of childbirth	2003-2009	During pregnancy	35	4.59(3.08 to 6.10)
Year of childbirth	2003-2009	During pregnancy	36	4.50(2.94 to 6.07)
Year of childbirth	2003-2009	During pregnancy	37	4.40(2.77 to 6.03)
Year of childbirth	2003-2009	During pregnancy	38	4.28(2.57 to 5.99)
Year of childbirth	2003-2009	During pregnancy	39	4.14(2.34 to 5.95)
Year of childbirth	2003-2009	During pregnancy	40	3.98(2.06 to 5.90)
Year of childbirth	2003-2009	During pregnancy	41	3.79(1.72 to 5.85)
Year of childbirth	2003-2009	After pregnancy	1	4.94(3.70 to 6.19)
Year of childbirth	2003-2009	After pregnancy	2	4.99(3.74 to 6.23)
Year of childbirth	2003-2009	After pregnancy	3	5.04(3.78 to 6.29)
Year of childbirth	2003-2009	After pregnancy	4	5.09(3.83 to 6.35)
Year of childbirth	2003-2009	After pregnancy	5	5.15(3.88 to 6.42)
Year of childbirth	2003-2009	After pregnancy	6	5.20(3.93 to 6.48)
Year of childbirth	2003-2009	After pregnancy	7	5.26(3.97 to 6.54)
Year of childbirth	2003-2009	After pregnancy	8	5.31(4.02 to 6.59)
Year of childbirth	2003-2009	After pregnancy	9	5.35(4.05 to 6.64)
Year of childbirth	2003-2009	After pregnancy	10	5.38(4.08 to 6.69)
Year of childbirth	2003-2009	After pregnancy	11	5.42(4.11 to 6.72)
Year of childbirth	2003-2009	After pregnancy	12	5.45(4.14 to 6.76)
Year of childbirth	2003-2009	After pregnancy	13	5.48(4.16 to 6.79)
Year of childbirth	2003-2009	After pregnancy	14	5.50(4.18 to 6.82)
Year of childbirth	2003-2009	After pregnancy	15	5.53(4.21 to 6.85)
Year of childbirth	2003-2009	After pregnancy	16	5.55(4.23 to 6.88)
Year of childbirth	2003-2009	After pregnancy	17	5.58(4.25 to 6.91)
Year of childbirth	2003-2009	After pregnancy	18	5.60(4.27 to 6.94)
Year of childbirth	2003-2009	After pregnancy	19	5.63(4.29 to 6.96)
Year of childbirth	2003-2009	After pregnancy	20	5.65(4.31 to 6.99)
Year of childbirth	2003-2009	After pregnancy	21	5.68(4.33 to 7.02)
Year of childbirth	2003-2009	After pregnancy	22	5.70(4.35 to 7.05)
Year of childbirth	2003-2009	After pregnancy	23	5.72(4.37 to 7.07)
Year of childbirth	2003-2009	After pregnancy	24	5.74(4.38 to 7.10)
Year of childbirth	2003-2009	After pregnancy	25	5.76(4.40 to 7.12)
Year of childbirth	2003-2009	After pregnancy	26	5.78(4.41 to 7.14)
Year of childbirth	2003-2009	After pregnancy	27	5.79(4.43 to 7.16)
Year of childbirth	2003-2009	After pregnancy	28	5.80(4.44 to 7.17)
Year of childbirth	2003-2009	After pregnancy	29	5.82(4.44 to 7.19)
Year of childbirth	2003-2009	After pregnancy	30	5.83(4.45 to 7.20)
Year of childbirth	2003-2009	After pregnancy	31	5.84(4.46 to 7.22)
Year of childbirth	2003-2009	After pregnancy	32	5.85(4.47 to 7.23)
Year of childbirth	2003-2009	After pregnancy	33	5.86(4.48 to 7.25)
Year of childbirth	2003-2009	After pregnancy	34	5.88(4.49 to 7.26)

© 2026 Xiang N et al. *JAMA Network Open*.

Year of childbirth	2003-2009	After pregnancy	35	5.89(4.50 to 7.28)
Year of childbirth	2003-2009	After pregnancy	36	5.91(4.51 to 7.30)
Year of childbirth	2003-2009	After pregnancy	37	5.93(4.53 to 7.32)
Year of childbirth	2003-2009	After pregnancy	38	5.94(4.54 to 7.34)
Year of childbirth	2003-2009	After pregnancy	39	5.96(4.55 to 7.36)
Year of childbirth	2003-2009	After pregnancy	40	5.97(4.56 to 7.38)
Year of childbirth	2003-2009	After pregnancy	41	5.98(4.57 to 7.39)
Year of childbirth	2003-2009	After pregnancy	42	5.99(4.58 to 7.40)
Year of childbirth	2003-2009	After pregnancy	43	5.99(4.58 to 7.41)
Year of childbirth	2003-2009	After pregnancy	44	5.99(4.58 to 7.41)
Year of childbirth	2003-2009	After pregnancy	45	5.99(4.58 to 7.41)
Year of childbirth	2003-2009	After pregnancy	46	5.99(4.57 to 7.41)
Year of childbirth	2003-2009	After pregnancy	47	5.98(4.57 to 7.40)
Year of childbirth	2003-2009	After pregnancy	48	5.98(4.56 to 7.39)
Year of childbirth	2003-2009	After pregnancy	49	5.96(4.55 to 7.38)
Year of childbirth	2003-2009	After pregnancy	50	5.95(4.54 to 7.36)
Year of childbirth	2003-2009	After pregnancy	51	5.94(4.53 to 7.35)
Year of childbirth	2010-2021	Before pregnancy	1	7.30(5.94 to 8.66)
Year of childbirth	2010-2021	Before pregnancy	2	7.31(5.95 to 8.66)
Year of childbirth	2010-2021	Before pregnancy	3	7.31(5.95 to 8.66)
Year of childbirth	2010-2021	Before pregnancy	4	7.29(5.94 to 8.65)
Year of childbirth	2010-2021	Before pregnancy	5	7.28(5.93 to 8.62)
Year of childbirth	2010-2021	Before pregnancy	6	7.25(5.91 to 8.59)
Year of childbirth	2010-2021	Before pregnancy	7	7.22(5.88 to 8.55)
Year of childbirth	2010-2021	Before pregnancy	8	7.18(5.85 to 8.51)
Year of childbirth	2010-2021	Before pregnancy	9	7.14(5.82 to 8.47)
Year of childbirth	2010-2021	Before pregnancy	10	7.10(5.79 to 8.42)
Year of childbirth	2010-2021	Before pregnancy	11	7.07(5.75 to 8.38)
Year of childbirth	2010-2021	Before pregnancy	12	7.03(5.72 to 8.33)
Year of childbirth	2010-2021	Before pregnancy	13	6.99(5.69 to 8.29)
Year of childbirth	2010-2021	Before pregnancy	14	6.96(5.66 to 8.25)
Year of childbirth	2010-2021	Before pregnancy	15	6.93(5.64 to 8.22)
Year of childbirth	2010-2021	Before pregnancy	16	6.90(5.61 to 8.18)
Year of childbirth	2010-2021	Before pregnancy	17	6.87(5.59 to 8.15)
Year of childbirth	2010-2021	Before pregnancy	18	6.84(5.56 to 8.11)
Year of childbirth	2010-2021	Before pregnancy	19	6.81(5.54 to 8.07)
Year of childbirth	2010-2021	Before pregnancy	20	6.78(5.51 to 8.04)
Year of childbirth	2010-2021	Before pregnancy	21	6.75(5.49 to 8.01)
Year of childbirth	2010-2021	Before pregnancy	22	6.72(5.47 to 7.97)
Year of childbirth	2010-2021	Before pregnancy	23	6.70(5.45 to 7.94)
Year of childbirth	2010-2021	Before pregnancy	24	6.67(5.43 to 7.92)
Year of childbirth	2010-2021	Before pregnancy	25	6.65(5.42 to 7.89)
Year of childbirth	2010-2021	Before pregnancy	26	6.64(5.40 to 7.87)
Year of childbirth	2010-2021	Before pregnancy	27	6.62(5.39 to 7.86)
Year of childbirth	2010-2021	Before pregnancy	28	6.61(5.39 to 7.84)

© 2026 Xiang N et al. *JAMA Network Open*.

Year of childbirth	2010-2021	Before pregnancy	29	6.61(5.38 to 7.83)
Year of childbirth	2010-2021	Before pregnancy	30	6.60(5.38 to 7.82)
Year of childbirth	2010-2021	Before pregnancy	31	6.60(5.38 to 7.82)
Year of childbirth	2010-2021	Before pregnancy	32	6.59(5.38 to 7.81)
Year of childbirth	2010-2021	Before pregnancy	33	6.59(5.37 to 7.80)
Year of childbirth	2010-2021	Before pregnancy	34	6.58(5.37 to 7.79)
Year of childbirth	2010-2021	Before pregnancy	35	6.58(5.37 to 7.79)
Year of childbirth	2010-2021	Before pregnancy	36	6.57(5.37 to 7.78)
Year of childbirth	2010-2021	Before pregnancy	37	6.57(5.37 to 7.77)
Year of childbirth	2010-2021	Before pregnancy	38	6.56(5.36 to 7.76)
Year of childbirth	2010-2021	Before pregnancy	39	6.55(5.36 to 7.75)
Year of childbirth	2010-2021	Before pregnancy	40	6.55(5.35 to 7.74)
Year of childbirth	2010-2021	Before pregnancy	41	6.54(5.35 to 7.73)
Year of childbirth	2010-2021	Before pregnancy	42	6.52(5.34 to 7.71)
Year of childbirth	2010-2021	Before pregnancy	43	6.51(5.33 to 7.69)
Year of childbirth	2010-2021	Before pregnancy	44	6.50(5.32 to 7.68)
Year of childbirth	2010-2021	Before pregnancy	45	6.48(5.30 to 7.65)
Year of childbirth	2010-2021	Before pregnancy	46	6.45(5.28 to 7.63)
Year of childbirth	2010-2021	Before pregnancy	47	6.43(5.26 to 7.59)
Year of childbirth	2010-2021	Before pregnancy	48	6.40(5.23 to 7.56)
Year of childbirth	2010-2021	Before pregnancy	49	6.36(5.20 to 7.51)
Year of childbirth	2010-2021	Before pregnancy	50	6.31(5.16 to 7.46)
Year of childbirth	2010-2021	Before pregnancy	51	6.27(5.13 to 7.41)
Year of childbirth	2010-2021	During pregnancy	1	5.59(4.56 to 6.62)
Year of childbirth	2010-2021	During pregnancy	2	5.57(4.54 to 6.59)
Year of childbirth	2010-2021	During pregnancy	3	5.56(4.53 to 6.58)
Year of childbirth	2010-2021	During pregnancy	4	5.56(4.53 to 6.58)
Year of childbirth	2010-2021	During pregnancy	5	5.56(4.54 to 6.59)
Year of childbirth	2010-2021	During pregnancy	6	5.58(4.55 to 6.60)
Year of childbirth	2010-2021	During pregnancy	7	5.59(4.56 to 6.62)
Year of childbirth	2010-2021	During pregnancy	8	5.61(4.58 to 6.64)
Year of childbirth	2010-2021	During pregnancy	9	5.63(4.59 to 6.66)
Year of childbirth	2010-2021	During pregnancy	10	5.64(4.61 to 6.67)
Year of childbirth	2010-2021	During pregnancy	11	5.65(4.62 to 6.69)
Year of childbirth	2010-2021	During pregnancy	12	5.66(4.63 to 6.70)
Year of childbirth	2010-2021	During pregnancy	13	5.67(4.63 to 6.70)
Year of childbirth	2010-2021	During pregnancy	14	5.67(4.64 to 6.71)
Year of childbirth	2010-2021	During pregnancy	15	5.67(4.64 to 6.71)
Year of childbirth	2010-2021	During pregnancy	16	5.67(4.63 to 6.71)
Year of childbirth	2010-2021	During pregnancy	17	5.66(4.63 to 6.69)
Year of childbirth	2010-2021	During pregnancy	18	5.65(4.61 to 6.68)
Year of childbirth	2010-2021	During pregnancy	19	5.63(4.60 to 6.66)
Year of childbirth	2010-2021	During pregnancy	20	5.61(4.58 to 6.64)
Year of childbirth	2010-2021	During pregnancy	21	5.58(4.56 to 6.61)
Year of childbirth	2010-2021	During pregnancy	22	5.56(4.53 to 6.58)

© 2026 Xiang N et al. *JAMA Network Open*.

Year of childbirth	2010-2021	During pregnancy	23	5.53(4.51 to 6.56)
Year of childbirth	2010-2021	During pregnancy	24	5.50(4.48 to 6.52)
Year of childbirth	2010-2021	During pregnancy	25	5.47(4.45 to 6.49)
Year of childbirth	2010-2021	During pregnancy	26	5.43(4.41 to 6.45)
Year of childbirth	2010-2021	During pregnancy	27	5.39(4.36 to 6.41)
Year of childbirth	2010-2021	During pregnancy	28	5.33(4.30 to 6.36)
Year of childbirth	2010-2021	During pregnancy	29	5.27(4.23 to 6.31)
Year of childbirth	2010-2021	During pregnancy	30	5.21(4.15 to 6.27)
Year of childbirth	2010-2021	During pregnancy	31	5.14(4.07 to 6.22)
Year of childbirth	2010-2021	During pregnancy	32	5.08(3.98 to 6.18)
Year of childbirth	2010-2021	During pregnancy	33	5.01(3.88 to 6.14)
Year of childbirth	2010-2021	During pregnancy	34	4.94(3.77 to 6.11)
Year of childbirth	2010-2021	During pregnancy	35	4.87(3.66 to 6.08)
Year of childbirth	2010-2021	During pregnancy	36	4.79(3.53 to 6.05)
Year of childbirth	2010-2021	During pregnancy	37	4.71(3.39 to 6.02)
Year of childbirth	2010-2021	During pregnancy	38	4.61(3.22 to 6.00)
Year of childbirth	2010-2021	During pregnancy	39	4.49(3.02 to 5.96)
Year of childbirth	2010-2021	During pregnancy	40	4.34(2.77 to 5.91)
Year of childbirth	2010-2021	During pregnancy	41	4.15(2.45 to 5.84)
Year of childbirth	2010-2021	After pregnancy	1	5.33(4.33 to 6.33)
Year of childbirth	2010-2021	After pregnancy	2	5.36(4.36 to 6.37)
Year of childbirth	2010-2021	After pregnancy	3	5.39(4.38 to 6.39)
Year of childbirth	2010-2021	After pregnancy	4	5.41(4.40 to 6.42)
Year of childbirth	2010-2021	After pregnancy	5	5.43(4.42 to 6.44)
Year of childbirth	2010-2021	After pregnancy	6	5.44(4.43 to 6.45)
Year of childbirth	2010-2021	After pregnancy	7	5.46(4.45 to 6.47)
Year of childbirth	2010-2021	After pregnancy	8	5.48(4.46 to 6.49)
Year of childbirth	2010-2021	After pregnancy	9	5.50(4.48 to 6.52)
Year of childbirth	2010-2021	After pregnancy	10	5.52(4.50 to 6.54)
Year of childbirth	2010-2021	After pregnancy	11	5.55(4.52 to 6.57)
Year of childbirth	2010-2021	After pregnancy	12	5.57(4.55 to 6.60)
Year of childbirth	2010-2021	After pregnancy	13	5.60(4.57 to 6.63)
Year of childbirth	2010-2021	After pregnancy	14	5.63(4.60 to 6.66)
Year of childbirth	2010-2021	After pregnancy	15	5.66(4.62 to 6.69)
Year of childbirth	2010-2021	After pregnancy	16	5.68(4.65 to 6.72)
Year of childbirth	2010-2021	After pregnancy	17	5.71(4.67 to 6.75)
Year of childbirth	2010-2021	After pregnancy	18	5.74(4.69 to 6.78)
Year of childbirth	2010-2021	After pregnancy	19	5.77(4.72 to 6.81)
Year of childbirth	2010-2021	After pregnancy	20	5.79(4.74 to 6.84)
Year of childbirth	2010-2021	After pregnancy	21	5.82(4.76 to 6.87)
Year of childbirth	2010-2021	After pregnancy	22	5.84(4.79 to 6.90)
Year of childbirth	2010-2021	After pregnancy	23	5.87(4.81 to 6.93)
Year of childbirth	2010-2021	After pregnancy	24	5.90(4.83 to 6.96)
Year of childbirth	2010-2021	After pregnancy	25	5.93(4.86 to 6.99)
Year of childbirth	2010-2021	After pregnancy	26	5.96(4.88 to 7.03)

© 2026 Xiang N et al. *JAMA Network Open*.

Year of childbirth	2010-2021	After pregnancy	27	5.98(4.91 to 7.06)
Year of childbirth	2010-2021	After pregnancy	28	6.01(4.93 to 7.09)
Year of childbirth	2010-2021	After pregnancy	29	6.04(4.96 to 7.12)
Year of childbirth	2010-2021	After pregnancy	30	6.06(4.98 to 7.15)
Year of childbirth	2010-2021	After pregnancy	31	6.09(5.00 to 7.18)
Year of childbirth	2010-2021	After pregnancy	32	6.11(5.02 to 7.21)
Year of childbirth	2010-2021	After pregnancy	33	6.14(5.04 to 7.23)
Year of childbirth	2010-2021	After pregnancy	34	6.16(5.06 to 7.26)
Year of childbirth	2010-2021	After pregnancy	35	6.18(5.07 to 7.28)
Year of childbirth	2010-2021	After pregnancy	36	6.19(5.09 to 7.30)
Year of childbirth	2010-2021	After pregnancy	37	6.21(5.10 to 7.32)
Year of childbirth	2010-2021	After pregnancy	38	6.22(5.11 to 7.33)
Year of childbirth	2010-2021	After pregnancy	39	6.24(5.13 to 7.35)
Year of childbirth	2010-2021	After pregnancy	40	6.25(5.14 to 7.36)
Year of childbirth	2010-2021	After pregnancy	41	6.26(5.15 to 7.38)
Year of childbirth	2010-2021	After pregnancy	42	6.27(5.15 to 7.39)
Year of childbirth	2010-2021	After pregnancy	43	6.28(5.16 to 7.40)
Year of childbirth	2010-2021	After pregnancy	44	6.28(5.16 to 7.40)
Year of childbirth	2010-2021	After pregnancy	45	6.29(5.17 to 7.41)
Year of childbirth	2010-2021	After pregnancy	46	6.29(5.17 to 7.41)
Year of childbirth	2010-2021	After pregnancy	47	6.28(5.16 to 7.40)
Year of childbirth	2010-2021	After pregnancy	48	6.27(5.15 to 7.39)
Year of childbirth	2010-2021	After pregnancy	49	6.27(5.15 to 7.38)
Year of childbirth	2010-2021	After pregnancy	50	6.26(5.14 to 7.38)
Year of childbirth	2010-2021	After pregnancy	51	6.25(5.14 to 7.37)
Educational level	< 10 years	Before pregnancy	1	17.83(12.73 to 22.93)
Educational level	< 10 years	Before pregnancy	2	17.68(12.60 to 22.75)
Educational level	< 10 years	Before pregnancy	3	17.55(12.50 to 22.60)
Educational level	< 10 years	Before pregnancy	4	17.44(12.41 to 22.47)
Educational level	< 10 years	Before pregnancy	5	17.34(12.33 to 22.35)
Educational level	< 10 years	Before pregnancy	6	17.24(12.25 to 22.23)
Educational level	< 10 years	Before pregnancy	7	17.14(12.16 to 22.11)
Educational level	< 10 years	Before pregnancy	8	17.03(12.08 to 21.98)
Educational level	< 10 years	Before pregnancy	9	16.91(11.98 to 21.84)
Educational level	< 10 years	Before pregnancy	10	16.79(11.89 to 21.70)
Educational level	< 10 years	Before pregnancy	11	16.68(11.79 to 21.56)
Educational level	< 10 years	Before pregnancy	12	16.56(11.70 to 21.42)
Educational level	< 10 years	Before pregnancy	13	16.44(11.60 to 21.28)
Educational level	< 10 years	Before pregnancy	14	16.32(11.51 to 21.14)
Educational level	< 10 years	Before pregnancy	15	16.21(11.42 to 21.00)
Educational level	< 10 years	Before pregnancy	16	16.10(11.33 to 20.87)
Educational level	< 10 years	Before pregnancy	17	15.99(11.25 to 20.74)
Educational level	< 10 years	Before pregnancy	18	15.89(11.16 to 20.62)
Educational level	< 10 years	Before pregnancy	19	15.79(11.08 to 20.49)
Educational level	< 10 years	Before pregnancy	20	15.69(11.00 to 20.37)

© 2026 Xiang N et al. *JAMA Network Open*.

Educational level	< 10 years	Before pregnancy	21	15.58(10.92 to 20.25)
Educational level	< 10 years	Before pregnancy	22	15.48(10.84 to 20.13)
Educational level	< 10 years	Before pregnancy	23	15.39(10.77 to 20.01)
Educational level	< 10 years	Before pregnancy	24	15.30(10.70 to 19.91)
Educational level	< 10 years	Before pregnancy	25	15.23(10.64 to 19.81)
Educational level	< 10 years	Before pregnancy	26	15.17(10.60 to 19.74)
Educational level	< 10 years	Before pregnancy	27	15.13(10.57 to 19.69)
Educational level	< 10 years	Before pregnancy	28	15.11(10.56 to 19.66)
Educational level	< 10 years	Before pregnancy	29	15.11(10.56 to 19.65)
Educational level	< 10 years	Before pregnancy	30	15.11(10.57 to 19.65)
Educational level	< 10 years	Before pregnancy	31	15.12(10.58 to 19.65)
Educational level	< 10 years	Before pregnancy	32	15.13(10.60 to 19.66)
Educational level	< 10 years	Before pregnancy	33	15.14(10.62 to 19.67)
Educational level	< 10 years	Before pregnancy	34	15.17(10.64 to 19.69)
Educational level	< 10 years	Before pregnancy	35	15.20(10.67 to 19.72)
Educational level	< 10 years	Before pregnancy	36	15.23(10.71 to 19.76)
Educational level	< 10 years	Before pregnancy	37	15.28(10.76 to 19.80)
Educational level	< 10 years	Before pregnancy	38	15.33(10.81 to 19.86)
Educational level	< 10 years	Before pregnancy	39	15.39(10.86 to 19.92)
Educational level	< 10 years	Before pregnancy	40	15.45(10.92 to 19.98)
Educational level	< 10 years	Before pregnancy	41	15.52(10.98 to 20.06)
Educational level	< 10 years	Before pregnancy	42	15.59(11.05 to 20.13)
Educational level	< 10 years	Before pregnancy	43	15.67(11.12 to 20.21)
Educational level	< 10 years	Before pregnancy	44	15.74(11.19 to 20.29)
Educational level	< 10 years	Before pregnancy	45	15.81(11.25 to 20.37)
Educational level	< 10 years	Before pregnancy	46	15.86(11.31 to 20.42)
Educational level	< 10 years	Before pregnancy	47	15.89(11.34 to 20.45)
Educational level	< 10 years	Before pregnancy	48	15.88(11.33 to 20.43)
Educational level	< 10 years	Before pregnancy	49	15.82(11.29 to 20.36)
Educational level	< 10 years	Before pregnancy	50	15.71(11.20 to 20.23)
Educational level	< 10 years	Before pregnancy	51	15.55(11.07 to 20.04)
Educational level	< 10 years	During pregnancy	1	13.29(9.39 to 17.18)
Educational level	< 10 years	During pregnancy	2	13.33(9.43 to 17.23)
Educational level	< 10 years	During pregnancy	3	13.34(9.44 to 17.24)
Educational level	< 10 years	During pregnancy	4	13.34(9.44 to 17.24)
Educational level	< 10 years	During pregnancy	5	13.34(9.44 to 17.24)
Educational level	< 10 years	During pregnancy	6	13.35(9.45 to 17.25)
Educational level	< 10 years	During pregnancy	7	13.36(9.46 to 17.27)
Educational level	< 10 years	During pregnancy	8	13.38(9.47 to 17.29)
Educational level	< 10 years	During pregnancy	9	13.40(9.49 to 17.31)
Educational level	< 10 years	During pregnancy	10	13.41(9.49 to 17.32)
Educational level	< 10 years	During pregnancy	11	13.41(9.49 to 17.32)
Educational level	< 10 years	During pregnancy	12	13.40(9.48 to 17.31)
Educational level	< 10 years	During pregnancy	13	13.37(9.46 to 17.28)
Educational level	< 10 years	During pregnancy	14	13.33(9.43 to 17.24)

© 2026 Xiang N et al. *JAMA Network Open*.

Educational level	< 10 years	During pregnancy	15	13.28(9.38 to 17.18)
Educational level	< 10 years	During pregnancy	16	13.21(9.32 to 17.10)
Educational level	< 10 years	During pregnancy	17	13.12(9.24 to 17.00)
Educational level	< 10 years	During pregnancy	18	13.04(9.17 to 16.91)
Educational level	< 10 years	During pregnancy	19	12.96(9.11 to 16.82)
Educational level	< 10 years	During pregnancy	20	12.89(9.04 to 16.74)
Educational level	< 10 years	During pregnancy	21	12.83(8.99 to 16.67)
Educational level	< 10 years	During pregnancy	22	12.77(8.93 to 16.60)
Educational level	< 10 years	During pregnancy	23	12.70(8.87 to 16.52)
Educational level	< 10 years	During pregnancy	24	12.62(8.81 to 16.44)
Educational level	< 10 years	During pregnancy	25	12.53(8.72 to 16.34)
Educational level	< 10 years	During pregnancy	26	12.41(8.61 to 16.22)
Educational level	< 10 years	During pregnancy	27	12.26(8.46 to 16.07)
Educational level	< 10 years	During pregnancy	28	12.08(8.27 to 15.89)
Educational level	< 10 years	During pregnancy	29	11.86(8.05 to 15.69)
Educational level	< 10 years	During pregnancy	30	11.62(7.80 to 15.48)
Educational level	< 10 years	During pregnancy	31	11.37(7.53 to 15.26)
Educational level	< 10 years	During pregnancy	32	11.10(7.23 to 15.04)
Educational level	< 10 years	During pregnancy	33	10.82(6.92 to 14.83)
Educational level	< 10 years	During pregnancy	34	10.52(6.58 to 14.60)
Educational level	< 10 years	During pregnancy	35	10.20(6.20 to 14.37)
Educational level	< 10 years	During pregnancy	36	9.84(5.79 to 14.13)
Educational level	< 10 years	During pregnancy	37	9.44(5.31 to 13.85)
Educational level	< 10 years	During pregnancy	38	8.96(4.75 to 13.53)
Educational level	< 10 years	During pregnancy	39	8.38(4.07 to 13.13)
Educational level	< 10 years	During pregnancy	40	7.66(3.24 to 12.62)
Educational level	< 10 years	During pregnancy	41	6.77(2.21 to 11.98)
Educational level	< 10 years	After pregnancy	1	10.85(7.33 to 14.38)
Educational level	< 10 years	After pregnancy	2	11.15(7.58 to 14.72)
Educational level	< 10 years	After pregnancy	3	11.40(7.79 to 15.01)
Educational level	< 10 years	After pregnancy	4	11.60(7.96 to 15.25)
Educational level	< 10 years	After pregnancy	5	11.76(8.09 to 15.43)
Educational level	< 10 years	After pregnancy	6	11.88(8.19 to 15.57)
Educational level	< 10 years	After pregnancy	7	11.97(8.26 to 15.68)
Educational level	< 10 years	After pregnancy	8	12.04(8.31 to 15.76)
Educational level	< 10 years	After pregnancy	9	12.09(8.35 to 15.83)
Educational level	< 10 years	After pregnancy	10	12.14(8.39 to 15.88)
Educational level	< 10 years	After pregnancy	11	12.18(8.42 to 15.93)
Educational level	< 10 years	After pregnancy	12	12.22(8.45 to 15.98)
Educational level	< 10 years	After pregnancy	13	12.25(8.48 to 16.03)
Educational level	< 10 years	After pregnancy	14	12.29(8.50 to 16.08)
Educational level	< 10 years	After pregnancy	15	12.33(8.53 to 16.13)
Educational level	< 10 years	After pregnancy	16	12.37(8.56 to 16.18)
Educational level	< 10 years	After pregnancy	17	12.41(8.59 to 16.23)
Educational level	< 10 years	After pregnancy	18	12.45(8.62 to 16.27)

© 2026 Xiang N et al. *JAMA Network Open*.

Educational level	< 10 years	After pregnancy	19	12.48(8.64 to 16.32)
Educational level	< 10 years	After pregnancy	20	12.51(8.66 to 16.36)
Educational level	< 10 years	After pregnancy	21	12.55(8.68 to 16.41)
Educational level	< 10 years	After pregnancy	22	12.58(8.71 to 16.45)
Educational level	< 10 years	After pregnancy	23	12.62(8.74 to 16.51)
Educational level	< 10 years	After pregnancy	24	12.68(8.78 to 16.58)
Educational level	< 10 years	After pregnancy	25	12.74(8.83 to 16.65)
Educational level	< 10 years	After pregnancy	26	12.81(8.88 to 16.74)
Educational level	< 10 years	After pregnancy	27	12.89(8.94 to 16.84)
Educational level	< 10 years	After pregnancy	28	12.98(9.01 to 16.94)
Educational level	< 10 years	After pregnancy	29	13.06(9.08 to 17.05)
Educational level	< 10 years	After pregnancy	30	13.15(9.15 to 17.16)
Educational level	< 10 years	After pregnancy	31	13.25(9.22 to 17.28)
Educational level	< 10 years	After pregnancy	32	13.34(9.29 to 17.39)
Educational level	< 10 years	After pregnancy	33	13.44(9.37 to 17.51)
Educational level	< 10 years	After pregnancy	34	13.54(9.44 to 17.63)
Educational level	< 10 years	After pregnancy	35	13.63(9.52 to 17.74)
Educational level	< 10 years	After pregnancy	36	13.72(9.59 to 17.85)
Educational level	< 10 years	After pregnancy	37	13.81(9.66 to 17.96)
Educational level	< 10 years	After pregnancy	38	13.88(9.72 to 18.05)
Educational level	< 10 years	After pregnancy	39	13.95(9.77 to 18.14)
Educational level	< 10 years	After pregnancy	40	14.01(9.82 to 18.21)
Educational level	< 10 years	After pregnancy	41	14.07(9.86 to 18.28)
Educational level	< 10 years	After pregnancy	42	14.11(9.89 to 18.33)
Educational level	< 10 years	After pregnancy	43	14.14(9.91 to 18.37)
Educational level	< 10 years	After pregnancy	44	14.15(9.92 to 18.39)
Educational level	< 10 years	After pregnancy	45	14.15(9.91 to 18.39)
Educational level	< 10 years	After pregnancy	46	14.13(9.89 to 18.37)
Educational level	< 10 years	After pregnancy	47	14.10(9.86 to 18.33)
Educational level	< 10 years	After pregnancy	48	14.05(9.82 to 18.28)
Educational level	< 10 years	After pregnancy	49	14.00(9.78 to 18.22)
Educational level	< 10 years	After pregnancy	50	13.95(9.73 to 18.16)
Educational level	< 10 years	After pregnancy	51	13.89(9.69 to 18.09)
Educational level	10-12 years	Before pregnancy	1	6.91(5.42 to 8.40)
Educational level	10-12 years	Before pregnancy	2	6.92(5.43 to 8.40)
Educational level	10-12 years	Before pregnancy	3	6.93(5.44 to 8.42)
Educational level	10-12 years	Before pregnancy	4	6.94(5.45 to 8.43)
Educational level	10-12 years	Before pregnancy	5	6.95(5.46 to 8.43)
Educational level	10-12 years	Before pregnancy	6	6.95(5.46 to 8.44)
Educational level	10-12 years	Before pregnancy	7	6.95(5.47 to 8.43)
Educational level	10-12 years	Before pregnancy	8	6.95(5.47 to 8.43)
Educational level	10-12 years	Before pregnancy	9	6.94(5.46 to 8.42)
Educational level	10-12 years	Before pregnancy	10	6.93(5.46 to 8.41)
Educational level	10-12 years	Before pregnancy	11	6.92(5.45 to 8.40)
Educational level	10-12 years	Before pregnancy	12	6.91(5.44 to 8.38)

© 2026 Xiang N et al. *JAMA Network Open*.

Educational level	10-12 years	Before pregnancy	13	6.90(5.43 to 8.36)
Educational level	10-12 years	Before pregnancy	14	6.88(5.42 to 8.35)
Educational level	10-12 years	Before pregnancy	15	6.87(5.41 to 8.33)
Educational level	10-12 years	Before pregnancy	16	6.86(5.40 to 8.31)
Educational level	10-12 years	Before pregnancy	17	6.84(5.39 to 8.30)
Educational level	10-12 years	Before pregnancy	18	6.83(5.38 to 8.28)
Educational level	10-12 years	Before pregnancy	19	6.82(5.37 to 8.26)
Educational level	10-12 years	Before pregnancy	20	6.80(5.36 to 8.24)
Educational level	10-12 years	Before pregnancy	21	6.79(5.35 to 8.23)
Educational level	10-12 years	Before pregnancy	22	6.78(5.34 to 8.21)
Educational level	10-12 years	Before pregnancy	23	6.77(5.34 to 8.20)
Educational level	10-12 years	Before pregnancy	24	6.76(5.33 to 8.18)
Educational level	10-12 years	Before pregnancy	25	6.75(5.32 to 8.17)
Educational level	10-12 years	Before pregnancy	26	6.73(5.31 to 8.15)
Educational level	10-12 years	Before pregnancy	27	6.72(5.30 to 8.14)
Educational level	10-12 years	Before pregnancy	28	6.70(5.29 to 8.12)
Educational level	10-12 years	Before pregnancy	29	6.69(5.28 to 8.10)
Educational level	10-12 years	Before pregnancy	30	6.67(5.27 to 8.07)
Educational level	10-12 years	Before pregnancy	31	6.65(5.25 to 8.05)
Educational level	10-12 years	Before pregnancy	32	6.63(5.23 to 8.02)
Educational level	10-12 years	Before pregnancy	33	6.61(5.22 to 8.00)
Educational level	10-12 years	Before pregnancy	34	6.59(5.20 to 7.97)
Educational level	10-12 years	Before pregnancy	35	6.57(5.18 to 7.95)
Educational level	10-12 years	Before pregnancy	36	6.54(5.17 to 7.92)
Educational level	10-12 years	Before pregnancy	37	6.52(5.15 to 7.89)
Educational level	10-12 years	Before pregnancy	38	6.49(5.13 to 7.86)
Educational level	10-12 years	Before pregnancy	39	6.46(5.10 to 7.83)
Educational level	10-12 years	Before pregnancy	40	6.43(5.08 to 7.79)
Educational level	10-12 years	Before pregnancy	41	6.40(5.05 to 7.75)
Educational level	10-12 years	Before pregnancy	42	6.36(5.02 to 7.71)
Educational level	10-12 years	Before pregnancy	43	6.32(4.98 to 7.66)
Educational level	10-12 years	Before pregnancy	44	6.27(4.94 to 7.60)
Educational level	10-12 years	Before pregnancy	45	6.22(4.89 to 7.54)
Educational level	10-12 years	Before pregnancy	46	6.15(4.84 to 7.47)
Educational level	10-12 years	Before pregnancy	47	6.09(4.78 to 7.39)
Educational level	10-12 years	Before pregnancy	48	6.01(4.72 to 7.31)
Educational level	10-12 years	Before pregnancy	49	5.94(4.65 to 7.22)
Educational level	10-12 years	Before pregnancy	50	5.86(4.58 to 7.13)
Educational level	10-12 years	Before pregnancy	51	5.78(4.52 to 7.05)
Educational level	10-12 years	During pregnancy	1	5.81(4.59 to 7.02)
Educational level	10-12 years	During pregnancy	2	5.75(4.54 to 6.96)
Educational level	10-12 years	During pregnancy	3	5.71(4.51 to 6.92)
Educational level	10-12 years	During pregnancy	4	5.69(4.49 to 6.90)
Educational level	10-12 years	During pregnancy	5	5.69(4.48 to 6.89)
Educational level	10-12 years	During pregnancy	6	5.69(4.48 to 6.89)

© 2026 Xiang N et al. *JAMA Network Open*.

Educational level	10-12 years	During pregnancy	7	5.69(4.48 to 6.89)
Educational level	10-12 years	During pregnancy	8	5.69(4.48 to 6.89)
Educational level	10-12 years	During pregnancy	9	5.68(4.48 to 6.89)
Educational level	10-12 years	During pregnancy	10	5.68(4.47 to 6.88)
Educational level	10-12 years	During pregnancy	11	5.66(4.46 to 6.86)
Educational level	10-12 years	During pregnancy	12	5.65(4.45 to 6.85)
Educational level	10-12 years	During pregnancy	13	5.63(4.43 to 6.83)
Educational level	10-12 years	During pregnancy	14	5.61(4.42 to 6.81)
Educational level	10-12 years	During pregnancy	15	5.59(4.40 to 6.79)
Educational level	10-12 years	During pregnancy	16	5.57(4.38 to 6.77)
Educational level	10-12 years	During pregnancy	17	5.55(4.36 to 6.74)
Educational level	10-12 years	During pregnancy	18	5.53(4.34 to 6.72)
Educational level	10-12 years	During pregnancy	19	5.50(4.31 to 6.69)
Educational level	10-12 years	During pregnancy	20	5.47(4.29 to 6.65)
Educational level	10-12 years	During pregnancy	21	5.44(4.26 to 6.62)
Educational level	10-12 years	During pregnancy	22	5.40(4.23 to 6.58)
Educational level	10-12 years	During pregnancy	23	5.37(4.20 to 6.54)
Educational level	10-12 years	During pregnancy	24	5.33(4.17 to 6.50)
Educational level	10-12 years	During pregnancy	25	5.30(4.13 to 6.47)
Educational level	10-12 years	During pregnancy	26	5.26(4.09 to 6.43)
Educational level	10-12 years	During pregnancy	27	5.22(4.05 to 6.39)
Educational level	10-12 years	During pregnancy	28	5.18(4.00 to 6.36)
Educational level	10-12 years	During pregnancy	29	5.13(3.94 to 6.33)
Educational level	10-12 years	During pregnancy	30	5.09(3.87 to 6.31)
Educational level	10-12 years	During pregnancy	31	5.04(3.80 to 6.29)
Educational level	10-12 years	During pregnancy	32	5.00(3.72 to 6.27)
Educational level	10-12 years	During pregnancy	33	4.95(3.64 to 6.26)
Educational level	10-12 years	During pregnancy	34	4.90(3.54 to 6.25)
Educational level	10-12 years	During pregnancy	35	4.85(3.44 to 6.25)
Educational level	10-12 years	During pregnancy	36	4.79(3.32 to 6.25)
Educational level	10-12 years	During pregnancy	37	4.72(3.18 to 6.26)
Educational level	10-12 years	During pregnancy	38	4.64(3.02 to 6.26)
Educational level	10-12 years	During pregnancy	39	4.54(2.82 to 6.26)
Educational level	10-12 years	During pregnancy	40	4.42(2.58 to 6.26)
Educational level	10-12 years	During pregnancy	41	4.25(2.27 to 6.24)
Educational level	10-12 years	After pregnancy	1	5.09(3.95 to 6.23)
Educational level	10-12 years	After pregnancy	2	5.13(3.98 to 6.27)
Educational level	10-12 years	After pregnancy	3	5.17(4.02 to 6.32)
Educational level	10-12 years	After pregnancy	4	5.22(4.06 to 6.37)
Educational level	10-12 years	After pregnancy	5	5.27(4.11 to 6.43)
Educational level	10-12 years	After pregnancy	6	5.32(4.15 to 6.49)
Educational level	10-12 years	After pregnancy	7	5.38(4.20 to 6.55)
Educational level	10-12 years	After pregnancy	8	5.43(4.25 to 6.61)
Educational level	10-12 years	After pregnancy	9	5.49(4.30 to 6.67)
Educational level	10-12 years	After pregnancy	10	5.54(4.35 to 6.74)

© 2026 Xiang N et al. *JAMA Network Open*.

Educational level	10-12 years	After pregnancy	11	5.60(4.40 to 6.80)
Educational level	10-12 years	After pregnancy	12	5.65(4.44 to 6.85)
Educational level	10-12 years	After pregnancy	13	5.70(4.49 to 6.91)
Educational level	10-12 years	After pregnancy	14	5.75(4.53 to 6.97)
Educational level	10-12 years	After pregnancy	15	5.80(4.58 to 7.02)
Educational level	10-12 years	After pregnancy	16	5.85(4.62 to 7.08)
Educational level	10-12 years	After pregnancy	17	5.89(4.66 to 7.13)
Educational level	10-12 years	After pregnancy	18	5.94(4.70 to 7.18)
Educational level	10-12 years	After pregnancy	19	5.99(4.74 to 7.24)
Educational level	10-12 years	After pregnancy	20	6.04(4.79 to 7.29)
Educational level	10-12 years	After pregnancy	21	6.09(4.83 to 7.35)
Educational level	10-12 years	After pregnancy	22	6.14(4.87 to 7.40)
Educational level	10-12 years	After pregnancy	23	6.18(4.91 to 7.45)
Educational level	10-12 years	After pregnancy	24	6.23(4.96 to 7.51)
Educational level	10-12 years	After pregnancy	25	6.28(4.99 to 7.56)
Educational level	10-12 years	After pregnancy	26	6.32(5.03 to 7.60)
Educational level	10-12 years	After pregnancy	27	6.36(5.07 to 7.65)
Educational level	10-12 years	After pregnancy	28	6.39(5.10 to 7.69)
Educational level	10-12 years	After pregnancy	29	6.43(5.12 to 7.73)
Educational level	10-12 years	After pregnancy	30	6.45(5.15 to 7.76)
Educational level	10-12 years	After pregnancy	31	6.48(5.17 to 7.79)
Educational level	10-12 years	After pregnancy	32	6.50(5.18 to 7.81)
Educational level	10-12 years	After pregnancy	33	6.51(5.20 to 7.83)
Educational level	10-12 years	After pregnancy	34	6.53(5.21 to 7.84)
Educational level	10-12 years	After pregnancy	35	6.54(5.22 to 7.86)
Educational level	10-12 years	After pregnancy	36	6.55(5.22 to 7.87)
Educational level	10-12 years	After pregnancy	37	6.56(5.23 to 7.88)
Educational level	10-12 years	After pregnancy	38	6.56(5.24 to 7.89)
Educational level	10-12 years	After pregnancy	39	6.57(5.24 to 7.90)
Educational level	10-12 years	After pregnancy	40	6.57(5.24 to 7.90)
Educational level	10-12 years	After pregnancy	41	6.57(5.24 to 7.90)
Educational level	10-12 years	After pregnancy	42	6.56(5.23 to 7.89)
Educational level	10-12 years	After pregnancy	43	6.54(5.21 to 7.87)
Educational level	10-12 years	After pregnancy	44	6.52(5.19 to 7.85)
Educational level	10-12 years	After pregnancy	45	6.49(5.17 to 7.82)
Educational level	10-12 years	After pregnancy	46	6.46(5.13 to 7.78)
Educational level	10-12 years	After pregnancy	47	6.41(5.09 to 7.73)
Educational level	10-12 years	After pregnancy	48	6.36(5.05 to 7.68)
Educational level	10-12 years	After pregnancy	49	6.31(5.00 to 7.62)
Educational level	10-12 years	After pregnancy	50	6.25(4.95 to 7.55)
Educational level	10-12 years	After pregnancy	51	6.19(4.90 to 7.49)
Educational level	> 13 years	Before pregnancy	1	4.37(3.07 to 5.68)
Educational level	> 13 years	Before pregnancy	2	4.31(3.02 to 5.60)
Educational level	> 13 years	Before pregnancy	3	4.24(2.97 to 5.52)
Educational level	> 13 years	Before pregnancy	4	4.18(2.91 to 5.44)

© 2026 Xiang N et al. *JAMA Network Open*.

Educational level	> 13 years	Before pregnancy	5	4.11(2.86 to 5.36)
Educational level	> 13 years	Before pregnancy	6	4.05(2.82 to 5.29)
Educational level	> 13 years	Before pregnancy	7	4.00(2.77 to 5.23)
Educational level	> 13 years	Before pregnancy	8	3.95(2.73 to 5.17)
Educational level	> 13 years	Before pregnancy	9	3.90(2.70 to 5.11)
Educational level	> 13 years	Before pregnancy	10	3.86(2.66 to 5.06)
Educational level	> 13 years	Before pregnancy	11	3.82(2.63 to 5.01)
Educational level	> 13 years	Before pregnancy	12	3.78(2.60 to 4.96)
Educational level	> 13 years	Before pregnancy	13	3.74(2.57 to 4.91)
Educational level	> 13 years	Before pregnancy	14	3.70(2.54 to 4.87)
Educational level	> 13 years	Before pregnancy	15	3.67(2.51 to 4.83)
Educational level	> 13 years	Before pregnancy	16	3.64(2.49 to 4.79)
Educational level	> 13 years	Before pregnancy	17	3.61(2.47 to 4.75)
Educational level	> 13 years	Before pregnancy	18	3.58(2.45 to 4.72)
Educational level	> 13 years	Before pregnancy	19	3.56(2.43 to 4.69)
Educational level	> 13 years	Before pregnancy	20	3.55(2.42 to 4.67)
Educational level	> 13 years	Before pregnancy	21	3.53(2.41 to 4.65)
Educational level	> 13 years	Before pregnancy	22	3.52(2.41 to 4.64)
Educational level	> 13 years	Before pregnancy	23	3.52(2.40 to 4.63)
Educational level	> 13 years	Before pregnancy	24	3.52(2.41 to 4.63)
Educational level	> 13 years	Before pregnancy	25	3.52(2.41 to 4.63)
Educational level	> 13 years	Before pregnancy	26	3.53(2.42 to 4.64)
Educational level	> 13 years	Before pregnancy	27	3.54(2.43 to 4.64)
Educational level	> 13 years	Before pregnancy	28	3.55(2.44 to 4.65)
Educational level	> 13 years	Before pregnancy	29	3.55(2.45 to 4.66)
Educational level	> 13 years	Before pregnancy	30	3.56(2.46 to 4.67)
Educational level	> 13 years	Before pregnancy	31	3.57(2.46 to 4.67)
Educational level	> 13 years	Before pregnancy	32	3.58(2.47 to 4.68)
Educational level	> 13 years	Before pregnancy	33	3.58(2.48 to 4.68)
Educational level	> 13 years	Before pregnancy	34	3.58(2.48 to 4.68)
Educational level	> 13 years	Before pregnancy	35	3.59(2.49 to 4.68)
Educational level	> 13 years	Before pregnancy	36	3.59(2.49 to 4.69)
Educational level	> 13 years	Before pregnancy	37	3.59(2.50 to 4.69)
Educational level	> 13 years	Before pregnancy	38	3.60(2.50 to 4.69)
Educational level	> 13 years	Before pregnancy	39	3.60(2.51 to 4.69)
Educational level	> 13 years	Before pregnancy	40	3.60(2.51 to 4.69)
Educational level	> 13 years	Before pregnancy	41	3.60(2.51 to 4.68)
Educational level	> 13 years	Before pregnancy	42	3.59(2.51 to 4.68)
Educational level	> 13 years	Before pregnancy	43	3.58(2.50 to 4.67)
Educational level	> 13 years	Before pregnancy	44	3.57(2.49 to 4.65)
Educational level	> 13 years	Before pregnancy	45	3.56(2.48 to 4.63)
Educational level	> 13 years	Before pregnancy	46	3.53(2.47 to 4.60)
Educational level	> 13 years	Before pregnancy	47	3.51(2.45 to 4.57)
Educational level	> 13 years	Before pregnancy	48	3.48(2.42 to 4.54)
Educational level	> 13 years	Before pregnancy	49	3.45(2.40 to 4.49)

© 2026 Xiang N et al. *JAMA Network Open*.

Educational level	> 13 years	Before pregnancy	50	3.41(2.37 to 4.45)
Educational level	> 13 years	Before pregnancy	51	3.37(2.33 to 4.40)
Educational level	> 13 years	During pregnancy	1	2.99(2.05 to 3.93)
Educational level	> 13 years	During pregnancy	2	3.02(2.08 to 3.97)
Educational level	> 13 years	During pregnancy	3	3.06(2.11 to 4.01)
Educational level	> 13 years	During pregnancy	4	3.09(2.13 to 4.04)
Educational level	> 13 years	During pregnancy	5	3.12(2.16 to 4.08)
Educational level	> 13 years	During pregnancy	6	3.15(2.18 to 4.11)
Educational level	> 13 years	During pregnancy	7	3.18(2.21 to 4.15)
Educational level	> 13 years	During pregnancy	8	3.21(2.24 to 4.19)
Educational level	> 13 years	During pregnancy	9	3.25(2.27 to 4.23)
Educational level	> 13 years	During pregnancy	10	3.29(2.30 to 4.28)
Educational level	> 13 years	During pregnancy	11	3.33(2.34 to 4.32)
Educational level	> 13 years	During pregnancy	12	3.37(2.37 to 4.37)
Educational level	> 13 years	During pregnancy	13	3.41(2.41 to 4.42)
Educational level	> 13 years	During pregnancy	14	3.45(2.44 to 4.46)
Educational level	> 13 years	During pregnancy	15	3.48(2.46 to 4.49)
Educational level	> 13 years	During pregnancy	16	3.50(2.48 to 4.52)
Educational level	> 13 years	During pregnancy	17	3.52(2.50 to 4.55)
Educational level	> 13 years	During pregnancy	18	3.54(2.51 to 4.56)
Educational level	> 13 years	During pregnancy	19	3.55(2.52 to 4.57)
Educational level	> 13 years	During pregnancy	20	3.55(2.52 to 4.58)
Educational level	> 13 years	During pregnancy	21	3.55(2.53 to 4.58)
Educational level	> 13 years	During pregnancy	22	3.55(2.52 to 4.58)
Educational level	> 13 years	During pregnancy	23	3.55(2.52 to 4.58)
Educational level	> 13 years	During pregnancy	24	3.54(2.52 to 4.57)
Educational level	> 13 years	During pregnancy	25	3.53(2.51 to 4.56)
Educational level	> 13 years	During pregnancy	26	3.52(2.49 to 4.55)
Educational level	> 13 years	During pregnancy	27	3.49(2.46 to 4.52)
Educational level	> 13 years	During pregnancy	28	3.46(2.42 to 4.50)
Educational level	> 13 years	During pregnancy	29	3.42(2.36 to 4.47)
Educational level	> 13 years	During pregnancy	30	3.37(2.30 to 4.43)
Educational level	> 13 years	During pregnancy	31	3.32(2.23 to 4.40)
Educational level	> 13 years	During pregnancy	32	3.26(2.16 to 4.37)
Educational level	> 13 years	During pregnancy	33	3.21(2.07 to 4.34)
Educational level	> 13 years	During pregnancy	34	3.15(1.99 to 4.32)
Educational level	> 13 years	During pregnancy	35	3.10(1.89 to 4.31)
Educational level	> 13 years	During pregnancy	36	3.05(1.79 to 4.30)
Educational level	> 13 years	During pregnancy	37	2.99(1.67 to 4.30)
Educational level	> 13 years	During pregnancy	38	2.92(1.54 to 4.29)
Educational level	> 13 years	During pregnancy	39	2.83(1.38 to 4.29)
Educational level	> 13 years	During pregnancy	40	2.74(1.18 to 4.29)
Educational level	> 13 years	During pregnancy	41	2.63(0.95 to 4.30)
Educational level	> 13 years	After pregnancy	1	3.79(2.73 to 4.84)
Educational level	> 13 years	After pregnancy	2	3.71(2.66 to 4.75)

© 2026 Xiang N et al. *JAMA Network Open*.

Educational level	> 13 years	After pregnancy	3	3.64(2.61 to 4.68)
Educational level	> 13 years	After pregnancy	4	3.59(2.56 to 4.62)
Educational level	> 13 years	After pregnancy	5	3.55(2.52 to 4.57)
Educational level	> 13 years	After pregnancy	6	3.51(2.49 to 4.53)
Educational level	> 13 years	After pregnancy	7	3.48(2.47 to 4.50)
Educational level	> 13 years	After pregnancy	8	3.46(2.45 to 4.47)
Educational level	> 13 years	After pregnancy	9	3.44(2.43 to 4.45)
Educational level	> 13 years	After pregnancy	10	3.42(2.41 to 4.43)
Educational level	> 13 years	After pregnancy	11	3.41(2.40 to 4.42)
Educational level	> 13 years	After pregnancy	12	3.40(2.39 to 4.41)
Educational level	> 13 years	After pregnancy	13	3.40(2.39 to 4.40)
Educational level	> 13 years	After pregnancy	14	3.40(2.39 to 4.40)
Educational level	> 13 years	After pregnancy	15	3.40(2.39 to 4.41)
Educational level	> 13 years	After pregnancy	16	3.41(2.40 to 4.42)
Educational level	> 13 years	After pregnancy	17	3.41(2.40 to 4.43)
Educational level	> 13 years	After pregnancy	18	3.42(2.41 to 4.44)
Educational level	> 13 years	After pregnancy	19	3.43(2.42 to 4.45)
Educational level	> 13 years	After pregnancy	20	3.45(2.43 to 4.46)
Educational level	> 13 years	After pregnancy	21	3.46(2.44 to 4.48)
Educational level	> 13 years	After pregnancy	22	3.47(2.45 to 4.49)
Educational level	> 13 years	After pregnancy	23	3.48(2.45 to 4.50)
Educational level	> 13 years	After pregnancy	24	3.49(2.46 to 4.51)
Educational level	> 13 years	After pregnancy	25	3.49(2.47 to 4.52)
Educational level	> 13 years	After pregnancy	26	3.50(2.47 to 4.53)
Educational level	> 13 years	After pregnancy	27	3.51(2.47 to 4.54)
Educational level	> 13 years	After pregnancy	28	3.51(2.48 to 4.54)
Educational level	> 13 years	After pregnancy	29	3.51(2.48 to 4.55)
Educational level	> 13 years	After pregnancy	30	3.52(2.48 to 4.55)
Educational level	> 13 years	After pregnancy	31	3.52(2.49 to 4.56)
Educational level	> 13 years	After pregnancy	32	3.53(2.49 to 4.56)
Educational level	> 13 years	After pregnancy	33	3.53(2.49 to 4.57)
Educational level	> 13 years	After pregnancy	34	3.53(2.49 to 4.57)
Educational level	> 13 years	After pregnancy	35	3.54(2.50 to 4.58)
Educational level	> 13 years	After pregnancy	36	3.54(2.50 to 4.58)
Educational level	> 13 years	After pregnancy	37	3.54(2.50 to 4.59)
Educational level	> 13 years	After pregnancy	38	3.55(2.50 to 4.59)
Educational level	> 13 years	After pregnancy	39	3.55(2.50 to 4.59)
Educational level	> 13 years	After pregnancy	40	3.55(2.50 to 4.59)
Educational level	> 13 years	After pregnancy	41	3.55(2.50 to 4.59)
Educational level	> 13 years	After pregnancy	42	3.55(2.50 to 4.59)
Educational level	> 13 years	After pregnancy	43	3.55(2.50 to 4.60)
Educational level	> 13 years	After pregnancy	44	3.55(2.50 to 4.60)
Educational level	> 13 years	After pregnancy	45	3.56(2.51 to 4.61)
Educational level	> 13 years	After pregnancy	46	3.58(2.52 to 4.63)
Educational level	> 13 years	After pregnancy	47	3.59(2.54 to 4.65)

© 2026 Xiang N et al. *JAMA Network Open*.

Educational level	> 13 years	After pregnancy	48	3.61(2.55 to 4.66)
Educational level	> 13 years	After pregnancy	49	3.62(2.57 to 4.68)
Educational level	> 13 years	After pregnancy	50	3.64(2.58 to 4.70)
Educational level	> 13 years	After pregnancy	51	3.66(2.60 to 4.73)
Country of birth	Sweden	Before pregnancy	1	6.50(5.38 to 7.61)
Country of birth	Sweden	Before pregnancy	2	6.46(5.34 to 7.57)
Country of birth	Sweden	Before pregnancy	3	6.43(5.32 to 7.54)
Country of birth	Sweden	Before pregnancy	4	6.40(5.29 to 7.51)
Country of birth	Sweden	Before pregnancy	5	6.38(5.27 to 7.48)
Country of birth	Sweden	Before pregnancy	6	6.35(5.25 to 7.45)
Country of birth	Sweden	Before pregnancy	7	6.33(5.23 to 7.42)
Country of birth	Sweden	Before pregnancy	8	6.30(5.21 to 7.39)
Country of birth	Sweden	Before pregnancy	9	6.27(5.18 to 7.36)
Country of birth	Sweden	Before pregnancy	10	6.24(5.16 to 7.32)
Country of birth	Sweden	Before pregnancy	11	6.20(5.13 to 7.28)
Country of birth	Sweden	Before pregnancy	12	6.17(5.10 to 7.25)
Country of birth	Sweden	Before pregnancy	13	6.14(5.07 to 7.21)
Country of birth	Sweden	Before pregnancy	14	6.11(5.04 to 7.17)
Country of birth	Sweden	Before pregnancy	15	6.08(5.02 to 7.14)
Country of birth	Sweden	Before pregnancy	16	6.06(5.00 to 7.11)
Country of birth	Sweden	Before pregnancy	17	6.03(4.98 to 7.08)
Country of birth	Sweden	Before pregnancy	18	6.01(4.96 to 7.06)
Country of birth	Sweden	Before pregnancy	19	5.99(4.94 to 7.03)
Country of birth	Sweden	Before pregnancy	20	5.97(4.93 to 7.01)
Country of birth	Sweden	Before pregnancy	21	5.95(4.91 to 6.99)
Country of birth	Sweden	Before pregnancy	22	5.94(4.90 to 6.97)
Country of birth	Sweden	Before pregnancy	23	5.93(4.89 to 6.96)
Country of birth	Sweden	Before pregnancy	24	5.91(4.88 to 6.94)
Country of birth	Sweden	Before pregnancy	25	5.90(4.88 to 6.93)
Country of birth	Sweden	Before pregnancy	26	5.90(4.87 to 6.92)
Country of birth	Sweden	Before pregnancy	27	5.89(4.86 to 6.91)
Country of birth	Sweden	Before pregnancy	28	5.88(4.86 to 6.90)
Country of birth	Sweden	Before pregnancy	29	5.88(4.86 to 6.89)
Country of birth	Sweden	Before pregnancy	30	5.87(4.85 to 6.89)
Country of birth	Sweden	Before pregnancy	31	5.87(4.85 to 6.88)
Country of birth	Sweden	Before pregnancy	32	5.86(4.85 to 6.87)
Country of birth	Sweden	Before pregnancy	33	5.86(4.85 to 6.87)
Country of birth	Sweden	Before pregnancy	34	5.85(4.84 to 6.86)
Country of birth	Sweden	Before pregnancy	35	5.85(4.84 to 6.86)
Country of birth	Sweden	Before pregnancy	36	5.85(4.84 to 6.85)
Country of birth	Sweden	Before pregnancy	37	5.85(4.84 to 6.85)
Country of birth	Sweden	Before pregnancy	38	5.84(4.84 to 6.85)
Country of birth	Sweden	Before pregnancy	39	5.84(4.84 to 6.84)
Country of birth	Sweden	Before pregnancy	40	5.83(4.83 to 6.83)
Country of birth	Sweden	Before pregnancy	41	5.82(4.83 to 6.82)

© 2026 Xiang N et al. *JAMA Network Open*.

Country of birth	Sweden	Before pregnancy	42	5.81(4.82 to 6.80)
Country of birth	Sweden	Before pregnancy	43	5.79(4.80 to 6.78)
Country of birth	Sweden	Before pregnancy	44	5.77(4.79 to 6.76)
Country of birth	Sweden	Before pregnancy	45	5.75(4.77 to 6.73)
Country of birth	Sweden	Before pregnancy	46	5.72(4.74 to 6.70)
Country of birth	Sweden	Before pregnancy	47	5.69(4.71 to 6.66)
Country of birth	Sweden	Before pregnancy	48	5.65(4.68 to 6.61)
Country of birth	Sweden	Before pregnancy	49	5.60(4.63 to 6.56)
Country of birth	Sweden	Before pregnancy	50	5.54(4.58 to 6.50)
Country of birth	Sweden	Before pregnancy	51	5.48(4.53 to 6.43)
Country of birth	Sweden	During pregnancy	1	5.20(4.31 to 6.09)
Country of birth	Sweden	During pregnancy	2	5.20(4.31 to 6.09)
Country of birth	Sweden	During pregnancy	3	5.21(4.32 to 6.10)
Country of birth	Sweden	During pregnancy	4	5.21(4.32 to 6.10)
Country of birth	Sweden	During pregnancy	5	5.22(4.33 to 6.11)
Country of birth	Sweden	During pregnancy	6	5.22(4.33 to 6.12)
Country of birth	Sweden	During pregnancy	7	5.23(4.34 to 6.12)
Country of birth	Sweden	During pregnancy	8	5.24(4.35 to 6.13)
Country of birth	Sweden	During pregnancy	9	5.25(4.35 to 6.14)
Country of birth	Sweden	During pregnancy	10	5.25(4.36 to 6.15)
Country of birth	Sweden	During pregnancy	11	5.25(4.36 to 6.15)
Country of birth	Sweden	During pregnancy	12	5.25(4.36 to 6.15)
Country of birth	Sweden	During pregnancy	13	5.25(4.35 to 6.14)
Country of birth	Sweden	During pregnancy	14	5.24(4.35 to 6.13)
Country of birth	Sweden	During pregnancy	15	5.23(4.34 to 6.12)
Country of birth	Sweden	During pregnancy	16	5.21(4.32 to 6.11)
Country of birth	Sweden	During pregnancy	17	5.20(4.31 to 6.09)
Country of birth	Sweden	During pregnancy	18	5.18(4.29 to 6.07)
Country of birth	Sweden	During pregnancy	19	5.15(4.27 to 6.04)
Country of birth	Sweden	During pregnancy	20	5.13(4.24 to 6.01)
Country of birth	Sweden	During pregnancy	21	5.10(4.22 to 5.99)
Country of birth	Sweden	During pregnancy	22	5.08(4.20 to 5.96)
Country of birth	Sweden	During pregnancy	23	5.05(4.17 to 5.93)
Country of birth	Sweden	During pregnancy	24	5.02(4.14 to 5.89)
Country of birth	Sweden	During pregnancy	25	4.98(4.11 to 5.86)
Country of birth	Sweden	During pregnancy	26	4.94(4.07 to 5.81)
Country of birth	Sweden	During pregnancy	27	4.89(4.01 to 5.77)
Country of birth	Sweden	During pregnancy	28	4.84(3.95 to 5.72)
Country of birth	Sweden	During pregnancy	29	4.77(3.88 to 5.66)
Country of birth	Sweden	During pregnancy	30	4.71(3.81 to 5.61)
Country of birth	Sweden	During pregnancy	31	4.64(3.72 to 5.56)
Country of birth	Sweden	During pregnancy	32	4.57(3.63 to 5.51)
Country of birth	Sweden	During pregnancy	33	4.50(3.54 to 5.46)
Country of birth	Sweden	During pregnancy	34	4.43(3.44 to 5.42)
Country of birth	Sweden	During pregnancy	35	4.35(3.32 to 5.37)

© 2026 Xiang N et al. *JAMA Network Open*.

Country of birth	Sweden	During pregnancy	36	4.26(3.20 to 5.32)
Country of birth	Sweden	During pregnancy	37	4.16(3.05 to 5.27)
Country of birth	Sweden	During pregnancy	38	4.05(2.89 to 5.22)
Country of birth	Sweden	During pregnancy	39	3.92(2.69 to 5.15)
Country of birth	Sweden	During pregnancy	40	3.77(2.46 to 5.08)
Country of birth	Sweden	During pregnancy	41	3.59(2.18 to 5.00)
Country of birth	Sweden	After pregnancy	1	5.01(4.14 to 5.88)
Country of birth	Sweden	After pregnancy	2	5.00(4.13 to 5.87)
Country of birth	Sweden	After pregnancy	3	5.00(4.13 to 5.87)
Country of birth	Sweden	After pregnancy	4	5.01(4.13 to 5.88)
Country of birth	Sweden	After pregnancy	5	5.01(4.14 to 5.89)
Country of birth	Sweden	After pregnancy	6	5.03(4.15 to 5.90)
Country of birth	Sweden	After pregnancy	7	5.04(4.17 to 5.92)
Country of birth	Sweden	After pregnancy	8	5.06(4.18 to 5.94)
Country of birth	Sweden	After pregnancy	9	5.08(4.20 to 5.96)
Country of birth	Sweden	After pregnancy	10	5.10(4.22 to 5.98)
Country of birth	Sweden	After pregnancy	11	5.12(4.24 to 6.01)
Country of birth	Sweden	After pregnancy	12	5.15(4.26 to 6.03)
Country of birth	Sweden	After pregnancy	13	5.17(4.28 to 6.06)
Country of birth	Sweden	After pregnancy	14	5.20(4.31 to 6.09)
Country of birth	Sweden	After pregnancy	15	5.23(4.33 to 6.12)
Country of birth	Sweden	After pregnancy	16	5.25(4.35 to 6.15)
Country of birth	Sweden	After pregnancy	17	5.28(4.38 to 6.18)
Country of birth	Sweden	After pregnancy	18	5.31(4.40 to 6.21)
Country of birth	Sweden	After pregnancy	19	5.34(4.43 to 6.24)
Country of birth	Sweden	After pregnancy	20	5.37(4.46 to 6.28)
Country of birth	Sweden	After pregnancy	21	5.39(4.48 to 6.31)
Country of birth	Sweden	After pregnancy	22	5.42(4.51 to 6.34)
Country of birth	Sweden	After pregnancy	23	5.45(4.53 to 6.37)
Country of birth	Sweden	After pregnancy	24	5.48(4.56 to 6.40)
Country of birth	Sweden	After pregnancy	25	5.51(4.58 to 6.43)
Country of birth	Sweden	After pregnancy	26	5.53(4.61 to 6.46)
Country of birth	Sweden	After pregnancy	27	5.56(4.63 to 6.49)
Country of birth	Sweden	After pregnancy	28	5.58(4.65 to 6.52)
Country of birth	Sweden	After pregnancy	29	5.61(4.67 to 6.54)
Country of birth	Sweden	After pregnancy	30	5.63(4.69 to 6.57)
Country of birth	Sweden	After pregnancy	31	5.65(4.71 to 6.59)
Country of birth	Sweden	After pregnancy	32	5.67(4.72 to 6.61)
Country of birth	Sweden	After pregnancy	33	5.68(4.74 to 6.63)
Country of birth	Sweden	After pregnancy	34	5.70(4.75 to 6.65)
Country of birth	Sweden	After pregnancy	35	5.71(4.76 to 6.66)
Country of birth	Sweden	After pregnancy	36	5.73(4.77 to 6.68)
Country of birth	Sweden	After pregnancy	37	5.74(4.78 to 6.69)
Country of birth	Sweden	After pregnancy	38	5.75(4.79 to 6.71)
Country of birth	Sweden	After pregnancy	39	5.76(4.80 to 6.72)

© 2026 Xiang N et al. *JAMA Network Open*.

Country of birth	Sweden	After pregnancy	40	5.77(4.81 to 6.73)
Country of birth	Sweden	After pregnancy	41	5.77(4.81 to 6.73)
Country of birth	Sweden	After pregnancy	42	5.77(4.81 to 6.74)
Country of birth	Sweden	After pregnancy	43	5.78(4.81 to 6.74)
Country of birth	Sweden	After pregnancy	44	5.77(4.81 to 6.74)
Country of birth	Sweden	After pregnancy	45	5.77(4.81 to 6.73)
Country of birth	Sweden	After pregnancy	46	5.76(4.80 to 6.72)
Country of birth	Sweden	After pregnancy	47	5.75(4.79 to 6.71)
Country of birth	Sweden	After pregnancy	48	5.73(4.77 to 6.69)
Country of birth	Sweden	After pregnancy	49	5.71(4.75 to 6.66)
Country of birth	Sweden	After pregnancy	50	5.68(4.72 to 6.63)
Country of birth	Sweden	After pregnancy	51	5.65(4.70 to 6.60)
Country of birth	Other	Before pregnancy	1	8.94(6.36 to 11.52)
Country of birth	Other	Before pregnancy	2	8.92(6.35 to 11.50)
Country of birth	Other	Before pregnancy	3	8.90(6.34 to 11.47)
Country of birth	Other	Before pregnancy	4	8.87(6.31 to 11.42)
Country of birth	Other	Before pregnancy	5	8.82(6.28 to 11.37)
Country of birth	Other	Before pregnancy	6	8.78(6.25 to 11.31)
Country of birth	Other	Before pregnancy	7	8.73(6.21 to 11.25)
Country of birth	Other	Before pregnancy	8	8.68(6.18 to 11.19)
Country of birth	Other	Before pregnancy	9	8.64(6.15 to 11.13)
Country of birth	Other	Before pregnancy	10	8.60(6.12 to 11.09)
Country of birth	Other	Before pregnancy	11	8.57(6.09 to 11.04)
Country of birth	Other	Before pregnancy	12	8.53(6.07 to 10.99)
Country of birth	Other	Before pregnancy	13	8.49(6.04 to 10.94)
Country of birth	Other	Before pregnancy	14	8.44(6.01 to 10.88)
Country of birth	Other	Before pregnancy	15	8.40(5.97 to 10.83)
Country of birth	Other	Before pregnancy	16	8.35(5.94 to 10.76)
Country of birth	Other	Before pregnancy	17	8.30(5.90 to 10.70)
Country of birth	Other	Before pregnancy	18	8.25(5.86 to 10.64)
Country of birth	Other	Before pregnancy	19	8.20(5.82 to 10.58)
Country of birth	Other	Before pregnancy	20	8.15(5.78 to 10.51)
Country of birth	Other	Before pregnancy	21	8.10(5.75 to 10.45)
Country of birth	Other	Before pregnancy	22	8.06(5.71 to 10.40)
Country of birth	Other	Before pregnancy	23	8.02(5.69 to 10.35)
Country of birth	Other	Before pregnancy	24	7.99(5.66 to 10.31)
Country of birth	Other	Before pregnancy	25	7.96(5.65 to 10.28)
Country of birth	Other	Before pregnancy	26	7.95(5.64 to 10.26)
Country of birth	Other	Before pregnancy	27	7.94(5.64 to 10.24)
Country of birth	Other	Before pregnancy	28	7.94(5.64 to 10.23)
Country of birth	Other	Before pregnancy	29	7.93(5.64 to 10.22)
Country of birth	Other	Before pregnancy	30	7.93(5.65 to 10.21)
Country of birth	Other	Before pregnancy	31	7.93(5.65 to 10.20)
Country of birth	Other	Before pregnancy	32	7.92(5.65 to 10.19)
Country of birth	Other	Before pregnancy	33	7.91(5.64 to 10.18)

© 2026 Xiang N et al. *JAMA Network Open*.

Country of birth	Other	Before pregnancy	34	7.90(5.64 to 10.16)
Country of birth	Other	Before pregnancy	35	7.88(5.63 to 10.13)
Country of birth	Other	Before pregnancy	36	7.87(5.62 to 10.11)
Country of birth	Other	Before pregnancy	37	7.85(5.62 to 10.09)
Country of birth	Other	Before pregnancy	38	7.84(5.61 to 10.07)
Country of birth	Other	Before pregnancy	39	7.83(5.60 to 10.05)
Country of birth	Other	Before pregnancy	40	7.82(5.60 to 10.04)
Country of birth	Other	Before pregnancy	41	7.81(5.60 to 10.03)
Country of birth	Other	Before pregnancy	42	7.80(5.60 to 10.01)
Country of birth	Other	Before pregnancy	43	7.79(5.59 to 9.99)
Country of birth	Other	Before pregnancy	44	7.77(5.58 to 9.96)
Country of birth	Other	Before pregnancy	45	7.73(5.55 to 9.92)
Country of birth	Other	Before pregnancy	46	7.68(5.51 to 9.85)
Country of birth	Other	Before pregnancy	47	7.61(5.46 to 9.76)
Country of birth	Other	Before pregnancy	48	7.52(5.39 to 9.66)
Country of birth	Other	Before pregnancy	49	7.42(5.30 to 9.53)
Country of birth	Other	Before pregnancy	50	7.29(5.20 to 9.38)
Country of birth	Other	Before pregnancy	51	7.14(5.08 to 9.21)
Country of birth	Other	During pregnancy	1	6.54(4.67 to 8.41)
Country of birth	Other	During pregnancy	2	6.50(4.64 to 8.36)
Country of birth	Other	During pregnancy	3	6.49(4.63 to 8.35)
Country of birth	Other	During pregnancy	4	6.50(4.64 to 8.35)
Country of birth	Other	During pregnancy	5	6.51(4.65 to 8.37)
Country of birth	Other	During pregnancy	6	6.54(4.68 to 8.40)
Country of birth	Other	During pregnancy	7	6.57(4.71 to 8.44)
Country of birth	Other	During pregnancy	8	6.61(4.74 to 8.48)
Country of birth	Other	During pregnancy	9	6.66(4.78 to 8.53)
Country of birth	Other	During pregnancy	10	6.70(4.82 to 8.58)
Country of birth	Other	During pregnancy	11	6.74(4.86 to 8.63)
Country of birth	Other	During pregnancy	12	6.78(4.89 to 8.67)
Country of birth	Other	During pregnancy	13	6.82(4.92 to 8.71)
Country of birth	Other	During pregnancy	14	6.84(4.95 to 8.74)
Country of birth	Other	During pregnancy	15	6.87(4.97 to 8.76)
Country of birth	Other	During pregnancy	16	6.88(4.98 to 8.78)
Country of birth	Other	During pregnancy	17	6.89(4.99 to 8.79)
Country of birth	Other	During pregnancy	18	6.88(4.99 to 8.78)
Country of birth	Other	During pregnancy	19	6.88(4.98 to 8.77)
Country of birth	Other	During pregnancy	20	6.87(4.97 to 8.76)
Country of birth	Other	During pregnancy	21	6.85(4.96 to 8.74)
Country of birth	Other	During pregnancy	22	6.83(4.94 to 8.72)
Country of birth	Other	During pregnancy	23	6.81(4.92 to 8.69)
Country of birth	Other	During pregnancy	24	6.78(4.90 to 8.66)
Country of birth	Other	During pregnancy	25	6.76(4.87 to 8.64)
Country of birth	Other	During pregnancy	26	6.73(4.84 to 8.61)
Country of birth	Other	During pregnancy	27	6.69(4.80 to 8.58)

© 2026 Xiang N et al. *JAMA Network Open*.

Country of birth	Other	During pregnancy	28	6.64(4.73 to 8.54)
Country of birth	Other	During pregnancy	29	6.58(4.65 to 8.51)
Country of birth	Other	During pregnancy	30	6.51(4.55 to 8.47)
Country of birth	Other	During pregnancy	31	6.44(4.44 to 8.44)
Country of birth	Other	During pregnancy	32	6.37(4.32 to 8.42)
Country of birth	Other	During pregnancy	33	6.31(4.20 to 8.41)
Country of birth	Other	During pregnancy	34	6.24(4.07 to 8.42)
Country of birth	Other	During pregnancy	35	6.18(3.92 to 8.45)
Country of birth	Other	During pregnancy	36	6.12(3.76 to 8.49)
Country of birth	Other	During pregnancy	37	6.06(3.58 to 8.54)
Country of birth	Other	During pregnancy	38	5.97(3.35 to 8.59)
Country of birth	Other	During pregnancy	39	5.85(3.07 to 8.63)
Country of birth	Other	During pregnancy	40	5.68(2.71 to 8.65)
Country of birth	Other	During pregnancy	41	5.45(2.25 to 8.66)
Country of birth	Other	After pregnancy	1	5.79(4.07 to 7.51)
Country of birth	Other	After pregnancy	2	5.97(4.22 to 7.71)
Country of birth	Other	After pregnancy	3	6.12(4.35 to 7.89)
Country of birth	Other	After pregnancy	4	6.25(4.46 to 8.04)
Country of birth	Other	After pregnancy	5	6.36(4.56 to 8.17)
Country of birth	Other	After pregnancy	6	6.46(4.64 to 8.28)
Country of birth	Other	After pregnancy	7	6.54(4.71 to 8.38)
Country of birth	Other	After pregnancy	8	6.61(4.77 to 8.46)
Country of birth	Other	After pregnancy	9	6.67(4.82 to 8.53)
Country of birth	Other	After pregnancy	10	6.73(4.86 to 8.59)
Country of birth	Other	After pregnancy	11	6.77(4.90 to 8.64)
Country of birth	Other	After pregnancy	12	6.81(4.93 to 8.69)
Country of birth	Other	After pregnancy	13	6.85(4.96 to 8.73)
Country of birth	Other	After pregnancy	14	6.88(4.99 to 8.78)
Country of birth	Other	After pregnancy	15	6.91(5.01 to 8.81)
Country of birth	Other	After pregnancy	16	6.94(5.03 to 8.85)
Country of birth	Other	After pregnancy	17	6.96(5.05 to 8.88)
Country of birth	Other	After pregnancy	18	6.99(5.07 to 8.90)
Country of birth	Other	After pregnancy	19	7.00(5.08 to 8.92)
Country of birth	Other	After pregnancy	20	7.02(5.09 to 8.94)
Country of birth	Other	After pregnancy	21	7.03(5.10 to 8.96)
Country of birth	Other	After pregnancy	22	7.05(5.11 to 8.98)
Country of birth	Other	After pregnancy	23	7.06(5.12 to 9.00)
Country of birth	Other	After pregnancy	24	7.08(5.14 to 9.02)
Country of birth	Other	After pregnancy	25	7.10(5.15 to 9.04)
Country of birth	Other	After pregnancy	26	7.11(5.16 to 9.07)
Country of birth	Other	After pregnancy	27	7.13(5.17 to 9.09)
Country of birth	Other	After pregnancy	28	7.15(5.18 to 9.11)
Country of birth	Other	After pregnancy	29	7.17(5.20 to 9.14)
Country of birth	Other	After pregnancy	30	7.19(5.21 to 9.16)
Country of birth	Other	After pregnancy	31	7.21(5.23 to 9.19)

© 2026 Xiang N et al. *JAMA Network Open*.

Country of birth	Other	After pregnancy	32	7.23(5.25 to 9.22)
Country of birth	Other	After pregnancy	33	7.26(5.26 to 9.25)
Country of birth	Other	After pregnancy	34	7.29(5.28 to 9.29)
Country of birth	Other	After pregnancy	35	7.31(5.31 to 9.32)
Country of birth	Other	After pregnancy	36	7.34(5.33 to 9.36)
Country of birth	Other	After pregnancy	37	7.37(5.35 to 9.40)
Country of birth	Other	After pregnancy	38	7.41(5.38 to 9.44)
Country of birth	Other	After pregnancy	39	7.44(5.41 to 9.48)
Country of birth	Other	After pregnancy	40	7.48(5.43 to 9.52)
Country of birth	Other	After pregnancy	41	7.51(5.46 to 9.56)
Country of birth	Other	After pregnancy	42	7.54(5.48 to 9.59)
Country of birth	Other	After pregnancy	43	7.57(5.50 to 9.63)
Country of birth	Other	After pregnancy	44	7.59(5.53 to 9.66)
Country of birth	Other	After pregnancy	45	7.62(5.54 to 9.69)
Country of birth	Other	After pregnancy	46	7.64(5.57 to 9.72)
Country of birth	Other	After pregnancy	47	7.67(5.59 to 9.75)
Country of birth	Other	After pregnancy	48	7.70(5.62 to 9.79)
Country of birth	Other	After pregnancy	49	7.74(5.65 to 9.83)
Country of birth	Other	After pregnancy	50	7.79(5.69 to 9.88)
Country of birth	Other	After pregnancy	51	7.85(5.75 to 9.95)
Number of children	0	Before pregnancy	1	6.88(5.79 to 7.98)
Number of children	0	Before pregnancy	2	6.85(5.77 to 7.94)
Number of children	0	Before pregnancy	3	6.82(5.74 to 7.91)
Number of children	0	Before pregnancy	4	6.79(5.72 to 7.87)
Number of children	0	Before pregnancy	5	6.76(5.69 to 7.84)
Number of children	0	Before pregnancy	6	6.73(5.66 to 7.80)
Number of children	0	Before pregnancy	7	6.70(5.63 to 7.76)
Number of children	0	Before pregnancy	8	6.66(5.60 to 7.72)
Number of children	0	Before pregnancy	9	6.62(5.56 to 7.68)
Number of children	0	Before pregnancy	10	6.58(5.53 to 7.63)
Number of children	0	Before pregnancy	11	6.54(5.49 to 7.59)
Number of children	0	Before pregnancy	12	6.50(5.45 to 7.54)
Number of children	0	Before pregnancy	13	6.46(5.42 to 7.50)
Number of children	0	Before pregnancy	14	6.42(5.39 to 7.45)
Number of children	0	Before pregnancy	15	6.39(5.36 to 7.42)
Number of children	0	Before pregnancy	16	6.35(5.33 to 7.38)
Number of children	0	Before pregnancy	17	6.32(5.30 to 7.34)
Number of children	0	Before pregnancy	18	6.29(5.28 to 7.31)
Number of children	0	Before pregnancy	19	6.27(5.25 to 7.28)
Number of children	0	Before pregnancy	20	6.24(5.23 to 7.25)
Number of children	0	Before pregnancy	21	6.22(5.21 to 7.22)
Number of children	0	Before pregnancy	22	6.19(5.19 to 7.19)
Number of children	0	Before pregnancy	23	6.17(5.17 to 7.17)
Number of children	0	Before pregnancy	24	6.15(5.16 to 7.15)
Number of children	0	Before pregnancy	25	6.14(5.14 to 7.13)

© 2026 Xiang N et al. *JAMA Network Open*.

Number of children	0	Before pregnancy	26	6.12(5.13 to 7.11)
Number of children	0	Before pregnancy	27	6.11(5.13 to 7.10)
Number of children	0	Before pregnancy	28	6.11(5.12 to 7.09)
Number of children	0	Before pregnancy	29	6.10(5.12 to 7.08)
Number of children	0	Before pregnancy	30	6.10(5.11 to 7.08)
Number of children	0	Before pregnancy	31	6.09(5.11 to 7.07)
Number of children	0	Before pregnancy	32	6.09(5.11 to 7.07)
Number of children	0	Before pregnancy	33	6.09(5.11 to 7.07)
Number of children	0	Before pregnancy	34	6.09(5.11 to 7.06)
Number of children	0	Before pregnancy	35	6.09(5.11 to 7.06)
Number of children	0	Before pregnancy	36	6.09(5.12 to 7.06)
Number of children	0	Before pregnancy	37	6.08(5.12 to 7.05)
Number of children	0	Before pregnancy	38	6.08(5.11 to 7.05)
Number of children	0	Before pregnancy	39	6.08(5.11 to 7.04)
Number of children	0	Before pregnancy	40	6.07(5.11 to 7.04)
Number of children	0	Before pregnancy	41	6.07(5.10 to 7.03)
Number of children	0	Before pregnancy	42	6.06(5.10 to 7.02)
Number of children	0	Before pregnancy	43	6.04(5.09 to 7.00)
Number of children	0	Before pregnancy	44	6.03(5.07 to 6.98)
Number of children	0	Before pregnancy	45	6.01(5.06 to 6.96)
Number of children	0	Before pregnancy	46	5.98(5.03 to 6.93)
Number of children	0	Before pregnancy	47	5.95(5.01 to 6.89)
Number of children	0	Before pregnancy	48	5.91(4.97 to 6.85)
Number of children	0	Before pregnancy	49	5.86(4.92 to 6.79)
Number of children	0	Before pregnancy	50	5.79(4.87 to 6.72)
Number of children	0	Before pregnancy	51	5.72(4.80 to 6.64)
Number of children	0	During pregnancy	1	5.34(4.49 to 6.20)
Number of children	0	During pregnancy	2	5.34(4.48 to 6.20)
Number of children	0	During pregnancy	3	5.34(4.49 to 6.20)
Number of children	0	During pregnancy	4	5.35(4.49 to 6.21)
Number of children	0	During pregnancy	5	5.36(4.50 to 6.21)
Number of children	0	During pregnancy	6	5.36(4.51 to 6.22)
Number of children	0	During pregnancy	7	5.37(4.51 to 6.23)
Number of children	0	During pregnancy	8	5.39(4.53 to 6.24)
Number of children	0	During pregnancy	9	5.40(4.54 to 6.26)
Number of children	0	During pregnancy	10	5.41(4.55 to 6.27)
Number of children	0	During pregnancy	11	5.42(4.56 to 6.29)
Number of children	0	During pregnancy	12	5.43(4.57 to 6.30)
Number of children	0	During pregnancy	13	5.44(4.58 to 6.31)
Number of children	0	During pregnancy	14	5.45(4.58 to 6.31)
Number of children	0	During pregnancy	15	5.45(4.58 to 6.31)
Number of children	0	During pregnancy	16	5.44(4.58 to 6.31)
Number of children	0	During pregnancy	17	5.43(4.57 to 6.30)
Number of children	0	During pregnancy	18	5.42(4.56 to 6.28)
Number of children	0	During pregnancy	19	5.40(4.54 to 6.26)

© 2026 Xiang N et al. *JAMA Network Open*.

Number of children	0	During pregnancy	20	5.38(4.52 to 6.24)
Number of children	0	During pregnancy	21	5.36(4.50 to 6.22)
Number of children	0	During pregnancy	22	5.34(4.48 to 6.19)
Number of children	0	During pregnancy	23	5.32(4.46 to 6.17)
Number of children	0	During pregnancy	24	5.29(4.44 to 6.14)
Number of children	0	During pregnancy	25	5.26(4.41 to 6.11)
Number of children	0	During pregnancy	26	5.22(4.37 to 6.07)
Number of children	0	During pregnancy	27	5.18(4.32 to 6.03)
Number of children	0	During pregnancy	28	5.12(4.27 to 5.98)
Number of children	0	During pregnancy	29	5.06(4.19 to 5.93)
Number of children	0	During pregnancy	30	4.99(4.11 to 5.87)
Number of children	0	During pregnancy	31	4.92(4.02 to 5.81)
Number of children	0	During pregnancy	32	4.84(3.93 to 5.75)
Number of children	0	During pregnancy	33	4.76(3.83 to 5.70)
Number of children	0	During pregnancy	34	4.68(3.72 to 5.64)
Number of children	0	During pregnancy	35	4.60(3.61 to 5.59)
Number of children	0	During pregnancy	36	4.51(3.48 to 5.54)
Number of children	0	During pregnancy	37	4.41(3.33 to 5.49)
Number of children	0	During pregnancy	38	4.30(3.17 to 5.43)
Number of children	0	During pregnancy	39	4.16(2.97 to 5.35)
Number of children	0	During pregnancy	40	3.99(2.72 to 5.26)
Number of children	0	During pregnancy	41	3.79(2.42 to 5.15)
Number of children	0	After pregnancy	1	4.99(4.17 to 5.81)
Number of children	0	After pregnancy	2	5.02(4.19 to 5.84)
Number of children	0	After pregnancy	3	5.05(4.22 to 5.87)
Number of children	0	After pregnancy	4	5.07(4.24 to 5.90)
Number of children	0	After pregnancy	5	5.10(4.27 to 5.93)
Number of children	0	After pregnancy	6	5.13(4.29 to 5.96)
Number of children	0	After pregnancy	7	5.15(4.32 to 5.99)
Number of children	0	After pregnancy	8	5.18(4.34 to 6.02)
Number of children	0	After pregnancy	9	5.21(4.36 to 6.05)
Number of children	0	After pregnancy	10	5.23(4.39 to 6.08)
Number of children	0	After pregnancy	11	5.26(4.41 to 6.11)
Number of children	0	After pregnancy	12	5.29(4.44 to 6.14)
Number of children	0	After pregnancy	13	5.31(4.46 to 6.17)
Number of children	0	After pregnancy	14	5.34(4.49 to 6.20)
Number of children	0	After pregnancy	15	5.37(4.51 to 6.23)
Number of children	0	After pregnancy	16	5.40(4.54 to 6.26)
Number of children	0	After pregnancy	17	5.43(4.56 to 6.29)
Number of children	0	After pregnancy	18	5.45(4.59 to 6.32)
Number of children	0	After pregnancy	19	5.48(4.61 to 6.35)
Number of children	0	After pregnancy	20	5.50(4.63 to 6.38)
Number of children	0	After pregnancy	21	5.53(4.66 to 6.40)
Number of children	0	After pregnancy	22	5.55(4.68 to 6.43)
Number of children	0	After pregnancy	23	5.58(4.70 to 6.46)

© 2026 Xiang N et al. *JAMA Network Open*.

Number of children	0	After pregnancy	24	5.60(4.72 to 6.49)
Number of children	0	After pregnancy	25	5.63(4.74 to 6.52)
Number of children	0	After pregnancy	26	5.66(4.77 to 6.54)
Number of children	0	After pregnancy	27	5.68(4.79 to 6.57)
Number of children	0	After pregnancy	28	5.70(4.81 to 6.60)
Number of children	0	After pregnancy	29	5.73(4.83 to 6.62)
Number of children	0	After pregnancy	30	5.75(4.85 to 6.65)
Number of children	0	After pregnancy	31	5.77(4.87 to 6.67)
Number of children	0	After pregnancy	32	5.79(4.88 to 6.69)
Number of children	0	After pregnancy	33	5.81(4.90 to 6.71)
Number of children	0	After pregnancy	34	5.82(4.91 to 6.73)
Number of children	0	After pregnancy	35	5.84(4.93 to 6.75)
Number of children	0	After pregnancy	36	5.86(4.94 to 6.77)
Number of children	0	After pregnancy	37	5.87(4.95 to 6.79)
Number of children	0	After pregnancy	38	5.89(4.97 to 6.80)
Number of children	0	After pregnancy	39	5.90(4.98 to 6.82)
Number of children	0	After pregnancy	40	5.91(4.99 to 6.83)
Number of children	0	After pregnancy	41	5.92(4.99 to 6.84)
Number of children	0	After pregnancy	42	5.93(5.00 to 6.85)
Number of children	0	After pregnancy	43	5.93(5.01 to 6.86)
Number of children	0	After pregnancy	44	5.94(5.01 to 6.86)
Number of children	0	After pregnancy	45	5.94(5.01 to 6.87)
Number of children	0	After pregnancy	46	5.94(5.01 to 6.86)
Number of children	0	After pregnancy	47	5.93(5.00 to 6.86)
Number of children	0	After pregnancy	48	5.92(4.99 to 6.85)
Number of children	0	After pregnancy	49	5.90(4.98 to 6.83)
Number of children	0	After pregnancy	50	5.89(4.96 to 6.81)
Number of children	0	After pregnancy	51	5.87(4.95 to 6.80)
Number of children	1	Before pregnancy	1	7.10(3.57 to 10.64)
Number of children	1	Before pregnancy	2	7.16(3.62 to 10.69)
Number of children	1	Before pregnancy	3	7.21(3.67 to 10.74)
Number of children	1	Before pregnancy	4	7.25(3.72 to 10.78)
Number of children	1	Before pregnancy	5	7.30(3.77 to 10.82)
Number of children	1	Before pregnancy	6	7.34(3.82 to 10.86)
Number of children	1	Before pregnancy	7	7.39(3.87 to 10.91)
Number of children	1	Before pregnancy	8	7.43(3.92 to 10.95)
Number of children	1	Before pregnancy	9	7.48(3.97 to 10.98)
Number of children	1	Before pregnancy	10	7.51(4.01 to 11.02)
Number of children	1	Before pregnancy	11	7.54(4.04 to 11.04)
Number of children	1	Before pregnancy	12	7.56(4.07 to 11.04)
Number of children	1	Before pregnancy	13	7.56(4.08 to 11.04)
Number of children	1	Before pregnancy	14	7.55(4.09 to 11.02)
Number of children	1	Before pregnancy	15	7.53(4.09 to 10.98)
Number of children	1	Before pregnancy	16	7.51(4.08 to 10.94)
Number of children	1	Before pregnancy	17	7.47(4.06 to 10.88)

© 2026 Xiang N et al. *JAMA Network Open*.

Number of children	1	Before pregnancy	18	7.43(4.04 to 10.83)
Number of children	1	Before pregnancy	19	7.39(4.02 to 10.76)
Number of children	1	Before pregnancy	20	7.35(4.00 to 10.70)
Number of children	1	Before pregnancy	21	7.31(3.98 to 10.65)
Number of children	1	Before pregnancy	22	7.28(3.96 to 10.60)
Number of children	1	Before pregnancy	23	7.26(3.96 to 10.56)
Number of children	1	Before pregnancy	24	7.24(3.95 to 10.53)
Number of children	1	Before pregnancy	25	7.23(3.96 to 10.51)
Number of children	1	Before pregnancy	26	7.23(3.96 to 10.49)
Number of children	1	Before pregnancy	27	7.22(3.97 to 10.48)
Number of children	1	Before pregnancy	28	7.21(3.97 to 10.45)
Number of children	1	Before pregnancy	29	7.19(3.96 to 10.42)
Number of children	1	Before pregnancy	30	7.16(3.95 to 10.38)
Number of children	1	Before pregnancy	31	7.13(3.94 to 10.33)
Number of children	1	Before pregnancy	32	7.10(3.92 to 10.28)
Number of children	1	Before pregnancy	33	7.06(3.90 to 10.23)
Number of children	1	Before pregnancy	34	7.03(3.88 to 10.17)
Number of children	1	Before pregnancy	35	7.00(3.86 to 10.13)
Number of children	1	Before pregnancy	36	6.97(3.85 to 10.09)
Number of children	1	Before pregnancy	37	6.95(3.85 to 10.06)
Number of children	1	Before pregnancy	38	6.94(3.85 to 10.04)
Number of children	1	Before pregnancy	39	6.94(3.85 to 10.02)
Number of children	1	Before pregnancy	40	6.93(3.86 to 10.01)
Number of children	1	Before pregnancy	41	6.93(3.87 to 10.00)
Number of children	1	Before pregnancy	42	6.93(3.88 to 9.99)
Number of children	1	Before pregnancy	43	6.93(3.88 to 9.97)
Number of children	1	Before pregnancy	44	6.91(3.88 to 9.94)
Number of children	1	Before pregnancy	45	6.88(3.87 to 9.90)
Number of children	1	Before pregnancy	46	6.83(3.84 to 9.83)
Number of children	1	Before pregnancy	47	6.77(3.80 to 9.74)
Number of children	1	Before pregnancy	48	6.69(3.75 to 9.64)
Number of children	1	Before pregnancy	49	6.61(3.69 to 9.53)
Number of children	1	Before pregnancy	50	6.52(3.63 to 9.41)
Number of children	1	Before pregnancy	51	6.44(3.58 to 9.31)
Number of children	1	During pregnancy	1	5.74(3.20 to 8.27)
Number of children	1	During pregnancy	2	5.64(3.13 to 8.16)
Number of children	1	During pregnancy	3	5.58(3.08 to 8.08)
Number of children	1	During pregnancy	4	5.55(3.05 to 8.05)
Number of children	1	During pregnancy	5	5.55(3.05 to 8.05)
Number of children	1	During pregnancy	6	5.57(3.07 to 8.08)
Number of children	1	During pregnancy	7	5.61(3.10 to 8.12)
Number of children	1	During pregnancy	8	5.66(3.14 to 8.18)
Number of children	1	During pregnancy	9	5.70(3.17 to 8.24)
Number of children	1	During pregnancy	10	5.74(3.20 to 8.29)
Number of children	1	During pregnancy	11	5.77(3.22 to 8.32)

© 2026 Xiang N et al. *JAMA Network Open*.

Number of children	1	During pregnancy	12	5.79(3.24 to 8.35)
Number of children	1	During pregnancy	13	5.80(3.24 to 8.36)
Number of children	1	During pregnancy	14	5.80(3.24 to 8.36)
Number of children	1	During pregnancy	15	5.80(3.24 to 8.36)
Number of children	1	During pregnancy	16	5.80(3.24 to 8.35)
Number of children	1	During pregnancy	17	5.79(3.23 to 8.34)
Number of children	1	During pregnancy	18	5.78(3.23 to 8.33)
Number of children	1	During pregnancy	19	5.77(3.22 to 8.32)
Number of children	1	During pregnancy	20	5.75(3.20 to 8.29)
Number of children	1	During pregnancy	21	5.72(3.18 to 8.27)
Number of children	1	During pregnancy	22	5.70(3.16 to 8.24)
Number of children	1	During pregnancy	23	5.67(3.14 to 8.20)
Number of children	1	During pregnancy	24	5.64(3.11 to 8.16)
Number of children	1	During pregnancy	25	5.59(3.07 to 8.11)
Number of children	1	During pregnancy	26	5.55(3.03 to 8.07)
Number of children	1	During pregnancy	27	5.50(2.97 to 8.04)
Number of children	1	During pregnancy	28	5.47(2.90 to 8.04)
Number of children	1	During pregnancy	29	5.44(2.82 to 8.07)
Number of children	1	During pregnancy	30	5.43(2.75 to 8.15)
Number of children	1	During pregnancy	31	5.45(2.67 to 8.27)
Number of children	1	During pregnancy	32	5.48(2.58 to 8.43)
Number of children	1	During pregnancy	33	5.53(2.50 to 8.63)
Number of children	1	During pregnancy	34	5.59(2.41 to 8.88)
Number of children	1	During pregnancy	35	5.68(2.30 to 9.18)
Number of children	1	During pregnancy	36	5.77(2.18 to 9.52)
Number of children	1	During pregnancy	37	5.88(2.03 to 9.92)
Number of children	1	During pregnancy	38	6.00(1.85 to 10.38)
Number of children	1	During pregnancy	39	6.13(1.63 to 10.92)
Number of children	1	During pregnancy	40	6.29(1.36 to 11.56)
Number of children	1	During pregnancy	41	6.48(1.03 to 12.37)
Number of children	1	After pregnancy	1	5.86(3.29 to 8.44)
Number of children	1	After pregnancy	2	6.00(3.40 to 8.60)
Number of children	1	After pregnancy	3	6.10(3.48 to 8.72)
Number of children	1	After pregnancy	4	6.17(3.54 to 8.81)
Number of children	1	After pregnancy	5	6.23(3.58 to 8.88)
Number of children	1	After pregnancy	6	6.28(3.62 to 8.94)
Number of children	1	After pregnancy	7	6.33(3.66 to 8.99)
Number of children	1	After pregnancy	8	6.37(3.69 to 9.05)
Number of children	1	After pregnancy	9	6.41(3.73 to 9.10)
Number of children	1	After pregnancy	10	6.46(3.76 to 9.15)
Number of children	1	After pregnancy	11	6.50(3.79 to 9.20)
Number of children	1	After pregnancy	12	6.54(3.82 to 9.26)
Number of children	1	After pregnancy	13	6.58(3.86 to 9.31)
Number of children	1	After pregnancy	14	6.62(3.89 to 9.36)
Number of children	1	After pregnancy	15	6.66(3.91 to 9.40)

© 2026 Xiang N et al. *JAMA Network Open*.

Number of children	1	After pregnancy	16	6.70(3.94 to 9.45)
Number of children	1	After pregnancy	17	6.73(3.97 to 9.50)
Number of children	1	After pregnancy	18	6.77(4.00 to 9.54)
Number of children	1	After pregnancy	19	6.81(4.03 to 9.59)
Number of children	1	After pregnancy	20	6.84(4.05 to 9.64)
Number of children	1	After pregnancy	21	6.88(4.08 to 9.68)
Number of children	1	After pregnancy	22	6.92(4.11 to 9.73)
Number of children	1	After pregnancy	23	6.97(4.15 to 9.79)
Number of children	1	After pregnancy	24	7.01(4.18 to 9.84)
Number of children	1	After pregnancy	25	7.06(4.22 to 9.90)
Number of children	1	After pregnancy	26	7.11(4.25 to 9.96)
Number of children	1	After pregnancy	27	7.16(4.29 to 10.02)
Number of children	1	After pregnancy	28	7.20(4.33 to 10.08)
Number of children	1	After pregnancy	29	7.25(4.36 to 10.14)
Number of children	1	After pregnancy	30	7.29(4.39 to 10.19)
Number of children	1	After pregnancy	31	7.33(4.42 to 10.24)
Number of children	1	After pregnancy	32	7.36(4.44 to 10.28)
Number of children	1	After pregnancy	33	7.39(4.46 to 10.32)
Number of children	1	After pregnancy	34	7.41(4.47 to 10.34)
Number of children	1	After pregnancy	35	7.42(4.48 to 10.36)
Number of children	1	After pregnancy	36	7.42(4.48 to 10.36)
Number of children	1	After pregnancy	37	7.42(4.48 to 10.36)
Number of children	1	After pregnancy	38	7.41(4.47 to 10.35)
Number of children	1	After pregnancy	39	7.40(4.46 to 10.33)
Number of children	1	After pregnancy	40	7.37(4.44 to 10.31)
Number of children	1	After pregnancy	41	7.34(4.42 to 10.27)
Number of children	1	After pregnancy	42	7.30(4.38 to 10.22)
Number of children	1	After pregnancy	43	7.24(4.34 to 10.15)
Number of children	1	After pregnancy	44	7.17(4.28 to 10.07)
Number of children	1	After pregnancy	45	7.09(4.21 to 9.96)
Number of children	1	After pregnancy	46	6.99(4.13 to 9.84)
Number of children	1	After pregnancy	47	6.87(4.04 to 9.70)
Number of children	1	After pregnancy	48	6.74(3.94 to 9.55)
Number of children	1	After pregnancy	49	6.60(3.83 to 9.38)
Number of children	1	After pregnancy	50	6.46(3.72 to 9.20)
Number of children	1	After pregnancy	51	6.30(3.59 to 9.00)
Number of children	2	Before pregnancy	1	14.47(3.17 to 25.91)
Number of children	2	Before pregnancy	2	13.79(2.90 to 24.90)
Number of children	2	Before pregnancy	3	13.17(2.65 to 23.97)
Number of children	2	Before pregnancy	4	12.63(2.44 to 23.15)
Number of children	2	Before pregnancy	5	12.17(2.26 to 22.46)
Number of children	2	Before pregnancy	6	11.79(2.11 to 21.88)
Number of children	2	Before pregnancy	7	11.48(1.98 to 21.39)
Number of children	2	Before pregnancy	8	11.21(1.88 to 20.97)
Number of children	2	Before pregnancy	9	10.98(1.80 to 20.60)

© 2026 Xiang N et al. *JAMA Network Open*.

Number of children	2	Before pregnancy	10	10.77(1.72 to 20.27)
Number of children	2	Before pregnancy	11	10.59(1.66 to 19.97)
Number of children	2	Before pregnancy	12	10.41(1.60 to 19.67)
Number of children	2	Before pregnancy	13	10.24(1.54 to 19.39)
Number of children	2	Before pregnancy	14	10.07(1.49 to 19.12)
Number of children	2	Before pregnancy	15	9.93(1.46 to 18.87)
Number of children	2	Before pregnancy	16	9.80(1.43 to 18.64)
Number of children	2	Before pregnancy	17	9.69(1.41 to 18.44)
Number of children	2	Before pregnancy	18	9.60(1.40 to 18.28)
Number of children	2	Before pregnancy	19	9.55(1.40 to 18.16)
Number of children	2	Before pregnancy	20	9.52(1.42 to 18.08)
Number of children	2	Before pregnancy	21	9.51(1.44 to 18.03)
Number of children	2	Before pregnancy	22	9.51(1.46 to 18.00)
Number of children	2	Before pregnancy	23	9.52(1.49 to 17.98)
Number of children	2	Before pregnancy	24	9.53(1.52 to 17.96)
Number of children	2	Before pregnancy	25	9.54(1.54 to 17.93)
Number of children	2	Before pregnancy	26	9.54(1.57 to 17.90)
Number of children	2	Before pregnancy	27	9.54(1.59 to 17.87)
Number of children	2	Before pregnancy	28	9.55(1.62 to 17.84)
Number of children	2	Before pregnancy	29	9.55(1.65 to 17.81)
Number of children	2	Before pregnancy	30	9.54(1.68 to 17.76)
Number of children	2	Before pregnancy	31	9.52(1.70 to 17.69)
Number of children	2	Before pregnancy	32	9.47(1.72 to 17.59)
Number of children	2	Before pregnancy	33	9.41(1.73 to 17.46)
Number of children	2	Before pregnancy	34	9.34(1.73 to 17.32)
Number of children	2	Before pregnancy	35	9.26(1.73 to 17.17)
Number of children	2	Before pregnancy	36	9.19(1.73 to 17.03)
Number of children	2	Before pregnancy	37	9.12(1.73 to 16.90)
Number of children	2	Before pregnancy	38	9.05(1.73 to 16.77)
Number of children	2	Before pregnancy	39	8.98(1.73 to 16.64)
Number of children	2	Before pregnancy	40	8.90(1.72 to 16.49)
Number of children	2	Before pregnancy	41	8.80(1.70 to 16.31)
Number of children	2	Before pregnancy	42	8.67(1.67 to 16.09)
Number of children	2	Before pregnancy	43	8.51(1.62 to 15.83)
Number of children	2	Before pregnancy	44	8.32(1.57 to 15.52)
Number of children	2	Before pregnancy	45	8.10(1.50 to 15.17)
Number of children	2	Before pregnancy	46	7.85(1.42 to 14.79)
Number of children	2	Before pregnancy	47	7.59(1.34 to 14.37)
Number of children	2	Before pregnancy	48	7.31(1.24 to 13.93)
Number of children	2	Before pregnancy	49	7.01(1.13 to 13.47)
Number of children	2	Before pregnancy	50	6.69(1.00 to 12.99)
Number of children	2	Before pregnancy	51	6.32(0.84 to 12.44)
Number of children	2	During pregnancy	1	7.60(1.33 to 13.86)
Number of children	2	During pregnancy	2	7.77(1.50 to 14.07)
Number of children	2	During pregnancy	3	7.98(1.68 to 14.34)

© 2026 Xiang N et al. *JAMA Network Open*.

Number of children	2	During pregnancy	4	8.20(1.85 to 14.64)
Number of children	2	During pregnancy	5	8.42(2.01 to 14.93)
Number of children	2	During pregnancy	6	8.62(2.15 to 15.19)
Number of children	2	During pregnancy	7	8.78(2.26 to 15.40)
Number of children	2	During pregnancy	8	8.89(2.34 to 15.55)
Number of children	2	During pregnancy	9	8.96(2.39 to 15.63)
Number of children	2	During pregnancy	10	8.98(2.41 to 15.66)
Number of children	2	During pregnancy	11	8.96(2.40 to 15.64)
Number of children	2	During pregnancy	12	8.93(2.38 to 15.59)
Number of children	2	During pregnancy	13	8.89(2.36 to 15.54)
Number of children	2	During pregnancy	14	8.85(2.32 to 15.48)
Number of children	2	During pregnancy	15	8.79(2.28 to 15.41)
Number of children	2	During pregnancy	16	8.73(2.23 to 15.32)
Number of children	2	During pregnancy	17	8.64(2.16 to 15.20)
Number of children	2	During pregnancy	18	8.53(2.09 to 15.06)
Number of children	2	During pregnancy	19	8.41(2.01 to 14.90)
Number of children	2	During pregnancy	20	8.27(1.92 to 14.72)
Number of children	2	During pregnancy	21	8.13(1.83 to 14.53)
Number of children	2	During pregnancy	22	7.99(1.74 to 14.33)
Number of children	2	During pregnancy	23	7.85(1.64 to 14.14)
Number of children	2	During pregnancy	24	7.71(1.56 to 13.96)
Number of children	2	During pregnancy	25	7.59(1.48 to 13.81)
Number of children	2	During pregnancy	26	7.50(1.43 to 13.70)
Number of children	2	During pregnancy	27	7.41(1.38 to 13.61)
Number of children	2	During pregnancy	28	7.33(1.34 to 13.54)
Number of children	2	During pregnancy	29	7.25(1.31 to 13.46)
Number of children	2	During pregnancy	30	7.16(1.28 to 13.37)
Number of children	2	During pregnancy	31	7.05(1.25 to 13.25)
Number of children	2	During pregnancy	32	6.93(1.23 to 13.12)
Number of children	2	During pregnancy	33	6.79(1.20 to 12.97)
Number of children	2	During pregnancy	34	6.62(1.16 to 12.79)
Number of children	2	During pregnancy	35	6.44(1.12 to 12.59)
Number of children	2	During pregnancy	36	6.25(1.08 to 12.37)
Number of children	2	During pregnancy	37	6.03(1.03 to 12.14)
Number of children	2	During pregnancy	38	5.79(0.98 to 11.87)
Number of children	2	During pregnancy	39	5.49(0.91 to 11.54)
Number of children	2	During pregnancy	40	5.08(0.80 to 11.05)
Number of children	2	During pregnancy	41	4.47(0.63 to 10.30)
Number of children	2	After pregnancy	1	6.72(1.11 to 12.54)
Number of children	2	After pregnancy	2	6.79(1.13 to 12.65)
Number of children	2	After pregnancy	3	6.92(1.18 to 12.84)
Number of children	2	After pregnancy	4	7.11(1.27 to 13.11)
Number of children	2	After pregnancy	5	7.33(1.38 to 13.43)
Number of children	2	After pregnancy	6	7.57(1.51 to 13.77)
Number of children	2	After pregnancy	7	7.81(1.64 to 14.09)

© 2026 Xiang N et al. *JAMA Network Open*.

Number of children	2	After pregnancy	8	8.03(1.76 to 14.40)
Number of children	2	After pregnancy	9	8.22(1.87 to 14.67)
Number of children	2	After pregnancy	10	8.40(1.97 to 14.92)
Number of children	2	After pregnancy	11	8.55(2.05 to 15.13)
Number of children	2	After pregnancy	12	8.67(2.12 to 15.30)
Number of children	2	After pregnancy	13	8.77(2.17 to 15.45)
Number of children	2	After pregnancy	14	8.85(2.22 to 15.57)
Number of children	2	After pregnancy	15	8.92(2.25 to 15.66)
Number of children	2	After pregnancy	16	8.96(2.26 to 15.73)
Number of children	2	After pregnancy	17	8.99(2.28 to 15.78)
Number of children	2	After pregnancy	18	9.01(2.28 to 15.81)
Number of children	2	After pregnancy	19	9.03(2.28 to 15.84)
Number of children	2	After pregnancy	20	9.04(2.28 to 15.86)
Number of children	2	After pregnancy	21	9.04(2.28 to 15.87)
Number of children	2	After pregnancy	22	9.03(2.26 to 15.86)
Number of children	2	After pregnancy	23	9.00(2.24 to 15.82)
Number of children	2	After pregnancy	24	8.95(2.21 to 15.76)
Number of children	2	After pregnancy	25	8.88(2.16 to 15.68)
Number of children	2	After pregnancy	26	8.80(2.11 to 15.57)
Number of children	2	After pregnancy	27	8.71(2.05 to 15.45)
Number of children	2	After pregnancy	28	8.61(1.99 to 15.33)
Number of children	2	After pregnancy	29	8.52(1.92 to 15.20)
Number of children	2	After pregnancy	30	8.42(1.85 to 15.08)
Number of children	2	After pregnancy	31	8.34(1.79 to 14.97)
Number of children	2	After pregnancy	32	8.26(1.74 to 14.88)
Number of children	2	After pregnancy	33	8.21(1.70 to 14.82)
Number of children	2	After pregnancy	34	8.20(1.67 to 14.80)
Number of children	2	After pregnancy	35	8.21(1.67 to 14.83)
Number of children	2	After pregnancy	36	8.26(1.70 to 14.90)
Number of children	2	After pregnancy	37	8.35(1.74 to 15.02)
Number of children	2	After pregnancy	38	8.46(1.81 to 15.18)
Number of children	2	After pregnancy	39	8.60(1.89 to 15.37)
Number of children	2	After pregnancy	40	8.76(1.99 to 15.59)
Number of children	2	After pregnancy	41	8.94(2.10 to 15.84)
Number of children	2	After pregnancy	42	9.16(2.23 to 16.13)
Number of children	2	After pregnancy	43	9.40(2.38 to 16.46)
Number of children	2	After pregnancy	44	9.68(2.56 to 16.84)
Number of children	2	After pregnancy	45	10.00(2.76 to 17.27)
Number of children	2	After pregnancy	46	10.36(2.99 to 17.75)
Number of children	2	After pregnancy	47	10.78(3.26 to 18.30)
Number of children	2	After pregnancy	48	11.24(3.56 to 18.93)
Number of children	2	After pregnancy	49	11.78(3.91 to 19.64)
Number of children	2	After pregnancy	50	12.40(4.32 to 20.47)
Number of children	2	After pregnancy	51	13.13(4.80 to 21.44)
Number of children	3+	Before pregnancy	1	6.44(0.00 to 15.80)

© 2026 Xiang N et al. *JAMA Network Open*.

Number of children	3+	Before pregnancy	2	6.76(0.00 to 16.74)
Number of children	3+	Before pregnancy	3	6.98(0.00 to 17.47)
Number of children	3+	Before pregnancy	4	7.15(-0.00 to 18.05)
Number of children	3+	Before pregnancy	5	7.29(-0.00 to 18.54)
Number of children	3+	Before pregnancy	6	7.42(-0.00 to 18.99)
Number of children	3+	Before pregnancy	7	7.56(-0.00 to 19.42)
Number of children	3+	Before pregnancy	8	7.73(0.00 to 19.87)
Number of children	3+	Before pregnancy	9	7.93(0.00 to 20.36)
Number of children	3+	Before pregnancy	10	8.17(0.00 to 20.89)
Number of children	3+	Before pregnancy	11	8.44(0.01 to 21.46)
Number of children	3+	Before pregnancy	12	8.73(0.01 to 22.06)
Number of children	3+	Before pregnancy	13	9.04(0.02 to 22.67)
Number of children	3+	Before pregnancy	14	9.35(0.02 to 23.27)
Number of children	3+	Before pregnancy	15	9.65(0.03 to 23.83)
Number of children	3+	Before pregnancy	16	9.92(0.03 to 24.35)
Number of children	3+	Before pregnancy	17	10.18(0.04 to 24.82)
Number of children	3+	Before pregnancy	18	10.41(0.04 to 25.24)
Number of children	3+	Before pregnancy	19	10.63(0.05 to 25.61)
Number of children	3+	Before pregnancy	20	10.83(0.05 to 25.93)
Number of children	3+	Before pregnancy	21	11.01(0.06 to 26.19)
Number of children	3+	Before pregnancy	22	11.17(0.06 to 26.42)
Number of children	3+	Before pregnancy	23	11.34(0.07 to 26.63)
Number of children	3+	Before pregnancy	24	11.51(0.07 to 26.84)
Number of children	3+	Before pregnancy	25	11.67(0.08 to 27.03)
Number of children	3+	Before pregnancy	26	11.83(0.08 to 27.21)
Number of children	3+	Before pregnancy	27	11.98(0.08 to 27.37)
Number of children	3+	Before pregnancy	28	12.12(0.08 to 27.52)
Number of children	3+	Before pregnancy	29	12.25(0.09 to 27.65)
Number of children	3+	Before pregnancy	30	12.35(0.09 to 27.73)
Number of children	3+	Before pregnancy	31	12.43(0.09 to 27.77)
Number of children	3+	Before pregnancy	32	12.47(0.09 to 27.75)
Number of children	3+	Before pregnancy	33	12.46(0.09 to 27.66)
Number of children	3+	Before pregnancy	34	12.41(0.08 to 27.49)
Number of children	3+	Before pregnancy	35	12.32(0.08 to 27.24)
Number of children	3+	Before pregnancy	36	12.20(0.08 to 26.94)
Number of children	3+	Before pregnancy	37	12.04(0.07 to 26.57)
Number of children	3+	Before pregnancy	38	11.85(0.07 to 26.17)
Number of children	3+	Before pregnancy	39	11.65(0.06 to 25.74)
Number of children	3+	Before pregnancy	40	11.43(0.05 to 25.28)
Number of children	3+	Before pregnancy	41	11.21(0.05 to 24.79)
Number of children	3+	Before pregnancy	42	10.97(0.04 to 24.29)
Number of children	3+	Before pregnancy	43	10.73(0.03 to 23.78)
Number of children	3+	Before pregnancy	44	10.47(0.03 to 23.24)
Number of children	3+	Before pregnancy	45	10.19(0.02 to 22.66)
Number of children	3+	Before pregnancy	46	9.88(0.01 to 22.02)

© 2026 Xiang N et al. *JAMA Network Open*.

Number of children	3+	Before pregnancy	47	9.53(0.00 to 21.32)
Number of children	3+	Before pregnancy	48	9.14(-0.00 to 20.56)
Number of children	3+	Before pregnancy	49	8.71(-0.01 to 19.74)
Number of children	3+	Before pregnancy	50	8.25(-0.02 to 18.86)
Number of children	3+	Before pregnancy	51	7.76(-0.02 to 17.94)
Number of children	3+	During pregnancy	1	12.19(1.62 to 23.73)
Number of children	3+	During pregnancy	2	12.11(1.65 to 23.53)
Number of children	3+	During pregnancy	3	11.99(1.62 to 23.30)
Number of children	3+	During pregnancy	4	11.82(1.55 to 23.04)
Number of children	3+	During pregnancy	5	11.62(1.46 to 22.77)
Number of children	3+	During pregnancy	6	11.42(1.36 to 22.50)
Number of children	3+	During pregnancy	7	11.21(1.27 to 22.21)
Number of children	3+	During pregnancy	8	10.99(1.19 to 21.88)
Number of children	3+	During pregnancy	9	10.75(1.11 to 21.52)
Number of children	3+	During pregnancy	10	10.50(1.03 to 21.12)
Number of children	3+	During pregnancy	11	10.25(0.95 to 20.71)
Number of children	3+	During pregnancy	12	9.99(0.88 to 20.29)
Number of children	3+	During pregnancy	13	9.74(0.80 to 19.90)
Number of children	3+	During pregnancy	14	9.51(0.72 to 19.54)
Number of children	3+	During pregnancy	15	9.31(0.64 to 19.25)
Number of children	3+	During pregnancy	16	9.14(0.56 to 19.02)
Number of children	3+	During pregnancy	17	9.01(0.48 to 18.85)
Number of children	3+	During pregnancy	18	8.93(0.41 to 18.76)
Number of children	3+	During pregnancy	19	8.89(0.36 to 18.74)
Number of children	3+	During pregnancy	20	8.88(0.33 to 18.76)
Number of children	3+	During pregnancy	21	8.90(0.32 to 18.81)
Number of children	3+	During pregnancy	22	8.92(0.33 to 18.86)
Number of children	3+	During pregnancy	23	8.93(0.33 to 18.88)
Number of children	3+	During pregnancy	24	8.93(0.33 to 18.91)
Number of children	3+	During pregnancy	25	8.94(0.33 to 18.95)
Number of children	3+	During pregnancy	26	8.94(0.33 to 18.99)
Number of children	3+	During pregnancy	27	8.92(0.32 to 19.00)
Number of children	3+	During pregnancy	28	8.89(0.31 to 18.97)
Number of children	3+	During pregnancy	29	8.83(0.30 to 18.91)
Number of children	3+	During pregnancy	30	8.77(0.29 to 18.83)
Number of children	3+	During pregnancy	31	8.68(0.27 to 18.69)
Number of children	3+	During pregnancy	32	8.54(0.26 to 18.48)
Number of children	3+	During pregnancy	33	8.36(0.24 to 18.15)
Number of children	3+	During pregnancy	34	8.08(0.23 to 17.67)
Number of children	3+	During pregnancy	35	7.70(0.20 to 16.98)
Number of children	3+	During pregnancy	36	7.21(0.16 to 16.06)
Number of children	3+	During pregnancy	37	6.59(0.11 to 14.94)
Number of children	3+	During pregnancy	38	5.87(0.05 to 13.60)
Number of children	3+	During pregnancy	39	5.04(-0.02 to 12.06)
Number of children	3+	During pregnancy	40	4.14(-0.09 to 10.32)

© 2026 Xiang N et al. *JAMA Network Open*.

Number of children	3+	During pregnancy	41	3.20(-0.15 to 8.41)
Number of children	3+	After pregnancy	1	11.73(0.68 to 23.58)
Number of children	3+	After pregnancy	2	11.38(0.61 to 23.06)
Number of children	3+	After pregnancy	3	11.11(0.55 to 22.66)
Number of children	3+	After pregnancy	4	10.88(0.50 to 22.31)
Number of children	3+	After pregnancy	5	10.66(0.45 to 21.96)
Number of children	3+	After pregnancy	6	10.43(0.41 to 21.59)
Number of children	3+	After pregnancy	7	10.17(0.37 to 21.15)
Number of children	3+	After pregnancy	8	9.88(0.33 to 20.66)
Number of children	3+	After pregnancy	9	9.57(0.30 to 20.12)
Number of children	3+	After pregnancy	10	9.25(0.27 to 19.55)
Number of children	3+	After pregnancy	11	8.91(0.24 to 18.97)
Number of children	3+	After pregnancy	12	8.59(0.21 to 18.40)
Number of children	3+	After pregnancy	13	8.30(0.19 to 17.88)
Number of children	3+	After pregnancy	14	8.04(0.18 to 17.42)
Number of children	3+	After pregnancy	15	7.82(0.17 to 17.03)
Number of children	3+	After pregnancy	16	7.64(0.17 to 16.69)
Number of children	3+	After pregnancy	17	7.49(0.17 to 16.40)
Number of children	3+	After pregnancy	18	7.37(0.17 to 16.16)
Number of children	3+	After pregnancy	19	7.27(0.18 to 15.95)
Number of children	3+	After pregnancy	20	7.19(0.18 to 15.77)
Number of children	3+	After pregnancy	21	7.13(0.19 to 15.62)
Number of children	3+	After pregnancy	22	7.07(0.20 to 15.49)
Number of children	3+	After pregnancy	23	7.01(0.20 to 15.36)
Number of children	3+	After pregnancy	24	6.96(0.21 to 15.23)
Number of children	3+	After pregnancy	25	6.90(0.21 to 15.12)
Number of children	3+	After pregnancy	26	6.84(0.21 to 15.02)
Number of children	3+	After pregnancy	27	6.78(0.21 to 14.92)
Number of children	3+	After pregnancy	28	6.72(0.21 to 14.83)
Number of children	3+	After pregnancy	29	6.68(0.21 to 14.77)
Number of children	3+	After pregnancy	30	6.65(0.21 to 14.75)
Number of children	3+	After pregnancy	31	6.66(0.21 to 14.79)
Number of children	3+	After pregnancy	32	6.72(0.21 to 14.91)
Number of children	3+	After pregnancy	33	6.82(0.22 to 15.11)
Number of children	3+	After pregnancy	34	6.95(0.22 to 15.38)
Number of children	3+	After pregnancy	35	7.12(0.23 to 15.70)
Number of children	3+	After pregnancy	36	7.32(0.23 to 16.06)
Number of children	3+	After pregnancy	37	7.52(0.24 to 16.44)
Number of children	3+	After pregnancy	38	7.72(0.24 to 16.83)
Number of children	3+	After pregnancy	39	7.92(0.24 to 17.21)
Number of children	3+	After pregnancy	40	8.12(0.24 to 17.59)
Number of children	3+	After pregnancy	41	8.30(0.24 to 17.96)
Number of children	3+	After pregnancy	42	8.49(0.24 to 18.34)
Number of children	3+	After pregnancy	43	8.69(0.24 to 18.73)
Number of children	3+	After pregnancy	44	8.92(0.24 to 19.17)

© 2026 Xiang N et al. *JAMA Network Open*.

Number of children	3+	After pregnancy	45	9.18(0.25 to 19.67)
Number of children	3+	After pregnancy	46	9.49(0.28 to 20.24)
Number of children	3+	After pregnancy	47	9.87(0.31 to 20.91)
Number of children	3+	After pregnancy	48	10.34(0.36 to 21.71)
Number of children	3+	After pregnancy	49	10.90(0.42 to 22.64)
Number of children	3+	After pregnancy	50	11.54(0.48 to 23.68)
Number of children	3+	After pregnancy	51	12.31(0.55 to 24.90)

*ADHD, attention deficit hyperactivity disorder.

*Incidence rate was standardized by age, calendar year at childbirth and week at follow-up.

© 2026 Xiang N et al. *JAMA Network Open*.

eTable 10. Incidence Rate Ratios of Any Paternal Psychiatric Disorder During and After Pregnancy, Stratified by Year of Childbirth, Education Level, Country of Birth, and Number of Children

Stratified variables	Strata	phase	Weeks	Incidence rate differences per 1000 person-years		
				Model 1	Model 2	Model 3
Year of childbirth	2003-2009	During pregnancy	0-4	0.84(0.72 to 0.98)	0.82(0.71 to 0.96)	0.83(0.71 to 0.97)
Year of childbirth	2003-2009	During pregnancy	5-9	0.87(0.75 to 1.02)	0.85(0.72 to 0.99)	0.86(0.73 to 1.00)
Year of childbirth	2003-2009	During pregnancy	10-14	0.99(0.84 to 1.16)	0.96(0.82 to 1.13)	0.97(0.83 to 1.14)
Year of childbirth	2003-2009	During pregnancy	15-19	0.83(0.71 to 0.97)	0.81(0.70 to 0.94)	0.82(0.70 to 0.95)
Year of childbirth	2003-2009	During pregnancy	20-24	0.98(0.84 to 1.14)	0.96(0.82 to 1.13)	0.97(0.83 to 1.14)
Year of childbirth	2003-2009	During pregnancy	25-29	0.90(0.76 to 1.06)	0.89(0.76 to 1.05)	0.89(0.76 to 1.05)
Year of childbirth	2003-2009	During pregnancy	30-34	0.90(0.77 to 1.06)	0.91(0.77 to 1.07)	0.91(0.78 to 1.07)
Year of childbirth	2003-2009	During pregnancy	35-39	0.77(0.65 to 0.91)	0.75(0.64 to 0.89)	0.75(0.64 to 0.89)
Year of childbirth	2003-2009	After pregnancy	0-4	0.76(0.65 to 0.89)	0.74(0.63 to 0.87)	0.75(0.64 to 0.88)
Year of childbirth	2003-2009	After pregnancy	5-9	0.85(0.72 to 0.99)	0.82(0.70 to 0.96)	0.83(0.71 to 0.97)
Year of childbirth	2003-2009	After pregnancy	10-14	0.98(0.84 to 1.15)	0.94(0.80 to 1.10)	0.95(0.81 to 1.11)
Year of childbirth	2003-2009	After pregnancy	15-19	0.93(0.80 to 1.08)	0.90(0.77 to 1.04)	0.90(0.78 to 1.05)
Year of childbirth	2003-2009	After pregnancy	20-24	0.96(0.82 to 1.13)	0.94(0.80 to 1.10)	0.94(0.80 to 1.11)
Year of childbirth	2003-2009	After pregnancy	25-29	1.03(0.88 to 1.21)	1.02(0.87 to 1.20)	1.03(0.88 to 1.21)
Year of childbirth	2003-2009	After pregnancy	30-34	1.06(0.90 to 1.23)	1.05(0.90 to 1.23)	1.06(0.90 to 1.24)
Year of childbirth	2003-2009	After pregnancy	35-39	1.03(0.89 to 1.20)	1.00(0.86 to 1.17)	1.01(0.87 to 1.17)
Year of childbirth	2003-2009	After pregnancy	40-44	0.94(0.81 to 1.09)	0.92(0.79 to 1.08)	0.93(0.80 to 1.08)
Year of childbirth	2003-2009	After pregnancy	45-49	1.14(0.97 to 1.33)	1.13(0.97 to 1.32)	1.14(0.97 to 1.33)
Year of childbirth	2003-2009	After pregnancy	50-51	1.08(0.84 to 1.38)	1.08(0.84 to 1.39)	1.09(0.84 to 1.40)
Year of childbirth	2010-2021	During pregnancy	0-4	0.78(0.70 to 0.88)	0.75(0.67 to 0.85)	0.76(0.67 to 0.85)
Year of childbirth	2010-2021	During pregnancy	5-9	0.75(0.66 to 0.84)	0.72(0.64 to 0.81)	0.73(0.65 to 0.82)
Year of childbirth	2010-2021	During pregnancy	10-14	0.79(0.71 to 0.89)	0.77(0.69 to 0.86)	0.78(0.69 to 0.87)
Year of childbirth	2010-2021	During pregnancy	15-19	0.87(0.78 to 0.98)	0.84(0.75 to 0.95)	0.85(0.75 to 0.95)
Year of childbirth	2010-2021	During pregnancy	20-24	0.88(0.79 to 1.00)	0.87(0.77 to 0.98)	0.87(0.78 to 0.98)

© 2026 Xiang N et al. *JAMA Network Open*.

Year of childbirth	2010-2021	During pregnancy	25-29	0.80(0.71 to 0.90)	0.77(0.68 to 0.87)	0.77(0.68 to 0.87)
Year of childbirth	2010-2021	During pregnancy	30-34	0.80(0.71 to 0.90)	0.78(0.69 to 0.88)	0.78(0.69 to 0.88)
Year of childbirth	2010-2021	During pregnancy	35-39	0.76(0.67 to 0.86)	0.75(0.66 to 0.84)	0.74(0.66 to 0.84)
Year of childbirth	2010-2021	After pregnancy	0-4	0.73(0.65 to 0.82)	0.71(0.63 to 0.80)	0.71(0.63 to 0.81)
Year of childbirth	2010-2021	After pregnancy	5-9	0.77(0.69 to 0.87)	0.73(0.65 to 0.82)	0.74(0.65 to 0.83)
Year of childbirth	2010-2021	After pregnancy	10-14	0.80(0.71 to 0.89)	0.77(0.68 to 0.86)	0.78(0.69 to 0.87)
Year of childbirth	2010-2021	After pregnancy	15-19	0.82(0.73 to 0.92)	0.79(0.70 to 0.89)	0.80(0.71 to 0.90)
Year of childbirth	2010-2021	After pregnancy	20-24	0.90(0.80 to 1.01)	0.87(0.77 to 0.98)	0.88(0.78 to 0.99)
Year of childbirth	2010-2021	After pregnancy	25-29	0.95(0.85 to 1.07)	0.94(0.83 to 1.05)	0.94(0.84 to 1.06)
Year of childbirth	2010-2021	After pregnancy	30-34	0.91(0.81 to 1.02)	0.90(0.80 to 1.01)	0.91(0.81 to 1.02)
Year of childbirth	2010-2021	After pregnancy	35-39	0.98(0.87 to 1.10)	0.97(0.87 to 1.09)	0.97(0.87 to 1.09)
Year of childbirth	2010-2021	After pregnancy	40-44	0.94(0.84 to 1.05)	0.93(0.83 to 1.05)	0.94(0.84 to 1.05)
Year of childbirth	2010-2021	After pregnancy	45-49	1.02(0.91 to 1.15)	1.00(0.89 to 1.12)	1.00(0.89 to 1.13)
Year of childbirth	2010-2021	After pregnancy	50-51	0.93(0.77 to 1.11)	0.91(0.75 to 1.09)	0.91(0.75 to 1.09)
Educational level	<10 years	During pregnancy	0-4	0.80(0.72 to 0.89)	0.79(0.71 to 0.88)	0.80(0.71 to 0.89)
Educational level	<10 years	During pregnancy	5-9	0.85(0.76 to 0.95)	0.83(0.75 to 0.93)	0.84(0.75 to 0.94)
Educational level	<10 years	During pregnancy	10-14	0.85(0.76 to 0.95)	0.84(0.75 to 0.93)	0.85(0.76 to 0.94)
Educational level	<10 years	During pregnancy	15-19	0.85(0.76 to 0.95)	0.84(0.75 to 0.93)	0.84(0.76 to 0.94)
Educational level	<10 years	During pregnancy	20-24	0.92(0.82 to 1.03)	0.91(0.82 to 1.02)	0.92(0.82 to 1.03)
Educational level	<10 years	During pregnancy	25-29	0.85(0.76 to 0.95)	0.83(0.74 to 0.93)	0.84(0.75 to 0.94)
Educational level	<10 years	During pregnancy	30-34	0.81(0.72 to 0.90)	0.80(0.71 to 0.89)	0.80(0.72 to 0.90)
Educational level	<10 years	During pregnancy	35-39	0.74(0.66 to 0.83)	0.73(0.65 to 0.83)	0.74(0.65 to 0.83)
Educational level	<10 years	After pregnancy	0-4	0.76(0.68 to 0.85)	0.74(0.67 to 0.83)	0.75(0.67 to 0.84)
Educational level	<10 years	After pregnancy	5-9	0.82(0.73 to 0.91)	0.80(0.72 to 0.89)	0.81(0.72 to 0.90)
Educational level	<10 years	After pregnancy	10-14	0.84(0.75 to 0.94)	0.83(0.74 to 0.92)	0.84(0.75 to 0.93)
Educational level	<10 years	After pregnancy	15-19	0.87(0.78 to 0.96)	0.86(0.77 to 0.95)	0.86(0.77 to 0.96)
Educational level	<10 years	After pregnancy	20-24	0.95(0.85 to 1.06)	0.94(0.84 to 1.05)	0.95(0.85 to 1.06)
Educational level	<10 years	After pregnancy	25-29	1.00(0.90 to 1.12)	0.99(0.89 to 1.11)	1.00(0.90 to 1.12)
Educational level	<10 years	After pregnancy	30-34	0.95(0.86 to 1.06)	0.95(0.85 to 1.06)	0.95(0.86 to 1.06)

© 2026 Xiang N et al. *JAMA Network Open*.

Educational level	<10 years	After pregnancy	35-39	1.00(0.90 to 1.11)	0.99(0.89 to 1.10)	1.00(0.90 to 1.11)
Educational level	<10 years	After pregnancy	40-44	0.96(0.87 to 1.07)	0.96(0.86 to 1.07)	0.96(0.87 to 1.07)
Educational level	<10 years	After pregnancy	45-49	1.08(0.97 to 1.21)	1.08(0.97 to 1.20)	1.08(0.97 to 1.20)
Educational level	<10 years	After pregnancy	50-51	0.92(0.78 to 1.09)	0.93(0.78 to 1.10)	0.93(0.78 to 1.10)
Educational level	10-12 years	During pregnancy	0-4	0.80(0.67 to 0.96)	0.75(0.62 to 0.90)	0.75(0.62 to 0.90)
Educational level	10-12 years	During pregnancy	5-9	0.63(0.52 to 0.75)	0.60(0.50 to 0.72)	0.60(0.50 to 0.72)
Educational level	10-12 years	During pregnancy	10-14	0.85(0.71 to 1.01)	0.81(0.68 to 0.97)	0.82(0.68 to 0.98)
Educational level	10-12 years	During pregnancy	15-19	0.86(0.72 to 1.03)	0.82(0.68 to 0.98)	0.81(0.68 to 0.98)
Educational level	10-12 years	During pregnancy	20-24	0.89(0.74 to 1.06)	0.87(0.73 to 1.04)	0.87(0.73 to 1.04)
Educational level	10-12 years	During pregnancy	25-29	0.78(0.65 to 0.95)	0.75(0.62 to 0.91)	0.75(0.62 to 0.91)
Educational level	10-12 years	During pregnancy	30-34	0.88(0.74 to 1.06)	0.89(0.74 to 1.07)	0.89(0.74 to 1.07)
Educational level	10-12 years	During pregnancy	35-39	0.81(0.67 to 0.97)	0.78(0.65 to 0.95)	0.78(0.64 to 0.94)
Educational level	10-12 years	After pregnancy	0-4	0.67(0.55 to 0.81)	0.66(0.54 to 0.80)	0.66(0.54 to 0.80)
Educational level	10-12 years	After pregnancy	5-9	0.73(0.61 to 0.87)	0.66(0.55 to 0.80)	0.66(0.55 to 0.80)
Educational level	10-12 years	After pregnancy	10-14	0.87(0.73 to 1.04)	0.81(0.68 to 0.97)	0.81(0.68 to 0.98)
Educational level	10-12 years	After pregnancy	15-19	0.83(0.69 to 1.00)	0.76(0.63 to 0.92)	0.76(0.63 to 0.92)
Educational level	10-12 years	After pregnancy	20-24	0.84(0.70 to 1.00)	0.78(0.65 to 0.94)	0.79(0.65 to 0.95)
Educational level	10-12 years	After pregnancy	25-29	0.90(0.75 to 1.08)	0.89(0.74 to 1.07)	0.89(0.74 to 1.08)
Educational level	10-12 years	After pregnancy	30-34	0.95(0.79 to 1.13)	0.96(0.80 to 1.15)	0.96(0.80 to 1.15)
Educational level	10-12 years	After pregnancy	35-39	0.99(0.83 to 1.17)	0.95(0.80 to 1.14)	0.95(0.80 to 1.14)
Educational level	10-12 years	After pregnancy	40-44	0.87(0.73 to 1.03)	0.85(0.71 to 1.02)	0.85(0.71 to 1.02)
Educational level	10-12 years	After pregnancy	45-49	0.99(0.83 to 1.19)	0.95(0.79 to 1.15)	0.96(0.80 to 1.15)
Educational level	10-12 years	After pregnancy	50-51	1.14(0.86 to 1.51)	1.08(0.81 to 1.45)	1.08(0.81 to 1.45)
Educational level	13+ years	During pregnancy	0-4	0.78(0.65 to 0.93)	0.76(0.63 to 0.91)	0.77(0.64 to 0.92)
Educational level	13+ years	During pregnancy	5-9	0.78(0.65 to 0.94)	0.77(0.64 to 0.92)	0.77(0.64 to 0.93)
Educational level	13+ years	During pregnancy	10-14	0.82(0.68 to 0.99)	0.80(0.66 to 0.96)	0.81(0.67 to 0.98)
Educational level	13+ years	During pregnancy	15-19	0.81(0.67 to 0.97)	0.79(0.66 to 0.95)	0.80(0.67 to 0.96)
Educational level	13+ years	During pregnancy	20-24	0.93(0.76 to 1.13)	0.91(0.75 to 1.11)	0.92(0.76 to 1.12)
Educational level	13+ years	During pregnancy	25-29	0.76(0.62 to 0.92)	0.75(0.61 to 0.91)	0.75(0.62 to 0.91)

© 2026 Xiang N et al. *JAMA Network Open*.

Educational level	13+ years	During pregnancy	30-34	0.89(0.73 to 1.09)	0.88(0.72 to 1.07)	0.89(0.73 to 1.08)
Educational level	13+ years	During pregnancy	35-39	0.72(0.58 to 0.89)	0.71(0.58 to 0.88)	0.71(0.58 to 0.88)
Educational level	13+ years	After pregnancy	0-4	0.59(0.48 to 0.72)	0.58(0.47 to 0.70)	0.59(0.48 to 0.71)
Educational level	13+ years	After pregnancy	5-9	0.78(0.65 to 0.94)	0.76(0.63 to 0.92)	0.77(0.64 to 0.93)
Educational level	13+ years	After pregnancy	10-14	0.82(0.68 to 0.99)	0.80(0.67 to 0.97)	0.81(0.68 to 0.98)
Educational level	13+ years	After pregnancy	15-19	0.72(0.60 to 0.87)	0.71(0.59 to 0.86)	0.71(0.59 to 0.86)
Educational level	13+ years	After pregnancy	20-24	0.82(0.67 to 1.00)	0.81(0.66 to 0.99)	0.82(0.67 to 1.00)
Educational level	13+ years	After pregnancy	25-29	0.85(0.70 to 1.03)	0.84(0.69 to 1.02)	0.85(0.70 to 1.03)
Educational level	13+ years	After pregnancy	30-34	1.00(0.82 to 1.22)	0.99(0.81 to 1.20)	1.00(0.82 to 1.21)
Educational level	13+ years	After pregnancy	35-39	0.98(0.82 to 1.19)	0.98(0.81 to 1.18)	0.98(0.81 to 1.18)
Educational level	13+ years	After pregnancy	40-44	0.82(0.68 to 0.98)	0.81(0.68 to 0.98)	0.82(0.68 to 0.98)
Educational level	13+ years	After pregnancy	45-49	1.03(0.86 to 1.25)	1.03(0.85 to 1.24)	1.04(0.86 to 1.25)
Educational level	13+ years	After pregnancy	50-51	0.75(0.56 to 1.01)	0.75(0.55 to 1.01)	0.75(0.56 to 1.01)
Country of birth	Sweden	During pregnancy	0-4	0.84(0.73 to 0.96)	0.82(0.72 to 0.94)	0.83(0.72 to 0.95)
Country of birth	Sweden	During pregnancy	5-9	0.79(0.69 to 0.90)	0.77(0.67 to 0.88)	0.78(0.68 to 0.89)
Country of birth	Sweden	During pregnancy	10-14	0.85(0.74 to 0.97)	0.83(0.72 to 0.95)	0.84(0.73 to 0.96)
Country of birth	Sweden	During pregnancy	15-19	0.81(0.71 to 0.92)	0.79(0.69 to 0.90)	0.80(0.70 to 0.91)
Country of birth	Sweden	During pregnancy	20-24	0.81(0.71 to 0.93)	0.80(0.70 to 0.92)	0.81(0.70 to 0.92)
Country of birth	Sweden	During pregnancy	25-29	0.77(0.67 to 0.89)	0.76(0.66 to 0.87)	0.76(0.66 to 0.88)
Country of birth	Sweden	During pregnancy	30-34	0.75(0.65 to 0.86)	0.74(0.64 to 0.85)	0.74(0.65 to 0.85)
Country of birth	Sweden	During pregnancy	35-39	0.76(0.66 to 0.88)	0.76(0.66 to 0.87)	0.76(0.66 to 0.87)
Country of birth	Sweden	After pregnancy	0-4	0.74(0.64 to 0.85)	0.72(0.63 to 0.83)	0.73(0.63 to 0.84)
Country of birth	Sweden	After pregnancy	5-9	0.78(0.68 to 0.89)	0.76(0.66 to 0.87)	0.77(0.67 to 0.88)
Country of birth	Sweden	After pregnancy	10-14	0.82(0.72 to 0.94)	0.81(0.71 to 0.93)	0.82(0.71 to 0.93)
Country of birth	Sweden	After pregnancy	15-19	0.86(0.75 to 0.98)	0.84(0.74 to 0.96)	0.85(0.74 to 0.97)
Country of birth	Sweden	After pregnancy	20-24	0.92(0.81 to 1.05)	0.91(0.80 to 1.04)	0.92(0.80 to 1.05)
Country of birth	Sweden	After pregnancy	25-29	0.95(0.84 to 1.09)	0.94(0.82 to 1.08)	0.95(0.83 to 1.08)
Country of birth	Sweden	After pregnancy	30-34	0.97(0.86 to 1.11)	0.96(0.85 to 1.10)	0.97(0.85 to 1.10)
Country of birth	Sweden	After pregnancy	35-39	1.02(0.90 to 1.15)	1.01(0.89 to 1.14)	1.01(0.89 to 1.15)

© 2026 Xiang N et al. *JAMA Network Open*.

Country of birth	Sweden	After pregnancy	40-44	1.07(0.94 to 1.22)	1.06(0.93 to 1.21)	1.06(0.93 to 1.21)
Country of birth	Sweden	After pregnancy	45-49	1.02(0.90 to 1.17)	1.02(0.89 to 1.16)	1.02(0.89 to 1.16)
Country of birth	Sweden	After pregnancy	50-51	1.06(0.85 to 1.31)	1.05(0.85 to 1.30)	1.05(0.85 to 1.31)
Country of birth	Other	During pregnancy	0-4	0.74(0.61 to 0.90)	0.72(0.60 to 0.88)	0.73(0.60 to 0.88)
Country of birth	Other	During pregnancy	5-9	0.75(0.62 to 0.91)	0.74(0.61 to 0.90)	0.74(0.61 to 0.90)
Country of birth	Other	During pregnancy	10-14	0.92(0.75 to 1.11)	0.90(0.74 to 1.10)	0.91(0.75 to 1.10)
Country of birth	Other	During pregnancy	15-19	1.01(0.83 to 1.23)	1.00(0.82 to 1.21)	1.00(0.82 to 1.22)
Country of birth	Other	During pregnancy	20-24	1.16(0.96 to 1.40)	1.15(0.95 to 1.38)	1.15(0.95 to 1.39)
Country of birth	Other	During pregnancy	25-29	1.00(0.82 to 1.22)	0.99(0.81 to 1.21)	0.99(0.81 to 1.21)
Country of birth	Other	During pregnancy	30-34	0.93(0.77 to 1.12)	0.92(0.76 to 1.11)	0.92(0.76 to 1.12)
Country of birth	Other	During pregnancy	35-39	0.79(0.65 to 0.97)	0.79(0.64 to 0.97)	0.79(0.64 to 0.97)
Country of birth	Other	After pregnancy	0-4	0.91(0.76 to 1.10)	0.90(0.74 to 1.08)	0.90(0.75 to 1.08)
Country of birth	Other	After pregnancy	5-9	0.78(0.64 to 0.94)	0.77(0.63 to 0.93)	0.77(0.64 to 0.93)
Country of birth	Other	After pregnancy	10-14	0.89(0.73 to 1.08)	0.88(0.72 to 1.07)	0.88(0.72 to 1.07)
Country of birth	Other	After pregnancy	15-19	1.00(0.82 to 1.22)	0.99(0.81 to 1.20)	0.99(0.82 to 1.21)
Country of birth	Other	After pregnancy	20-24	0.98(0.81 to 1.19)	0.97(0.80 to 1.18)	0.97(0.80 to 1.19)
Country of birth	Other	After pregnancy	25-29	1.16(0.96 to 1.41)	1.15(0.95 to 1.39)	1.15(0.95 to 1.40)
Country of birth	Other	After pregnancy	30-34	0.91(0.75 to 1.10)	0.91(0.75 to 1.10)	0.91(0.75 to 1.10)
Country of birth	Other	After pregnancy	35-39	0.98(0.81 to 1.18)	0.97(0.81 to 1.18)	0.98(0.81 to 1.18)
Country of birth	Other	After pregnancy	40-44	0.86(0.71 to 1.05)	0.86(0.71 to 1.04)	0.86(0.71 to 1.05)
Country of birth	Other	After pregnancy	45-49	1.12(0.93 to 1.35)	1.12(0.93 to 1.35)	1.12(0.93 to 1.35)
Country of birth	Other	After pregnancy	50-51	0.97(0.72 to 1.31)	0.97(0.72 to 1.31)	0.97(0.72 to 1.31)
Number of children	0	During pregnancy	0-4	0.79(0.72 to 0.87)	0.77(0.70 to 0.86)	0.78(0.71 to 0.87)
Number of children	0	During pregnancy	5-9	0.80(0.72 to 0.89)	0.78(0.71 to 0.87)	0.79(0.71 to 0.88)
Number of children	0	During pregnancy	10-14	0.83(0.75 to 0.92)	0.81(0.74 to 0.90)	0.82(0.74 to 0.91)
Number of children	0	During pregnancy	15-19	0.86(0.78 to 0.95)	0.84(0.76 to 0.93)	0.85(0.76 to 0.94)
Number of children	0	During pregnancy	20-24	0.95(0.85 to 1.05)	0.94(0.84 to 1.04)	0.94(0.85 to 1.05)
Number of children	0	During pregnancy	25-29	0.84(0.75 to 0.93)	0.82(0.74 to 0.92)	0.83(0.74 to 0.92)
Number of children	0	During pregnancy	30-34	0.84(0.76 to 0.93)	0.84(0.75 to 0.93)	0.84(0.76 to 0.93)

© 2026 Xiang N et al. *JAMA Network Open*.

Number of children	0	During pregnancy	35-39	0.76(0.68 to 0.85)	0.75(0.67 to 0.84)	0.75(0.67 to 0.84)
Number of children	0	After pregnancy	0-4	0.72(0.65 to 0.80)	0.71(0.64 to 0.79)	0.72(0.65 to 0.80)
Number of children	0	After pregnancy	5-9	0.79(0.72 to 0.88)	0.76(0.68 to 0.84)	0.77(0.69 to 0.85)
Number of children	0	After pregnancy	10-14	0.82(0.74 to 0.91)	0.80(0.72 to 0.89)	0.81(0.73 to 0.90)
Number of children	0	After pregnancy	15-19	0.85(0.77 to 0.95)	0.83(0.75 to 0.92)	0.84(0.76 to 0.93)
Number of children	0	After pregnancy	20-24	0.93(0.84 to 1.03)	0.91(0.82 to 1.01)	0.91(0.82 to 1.01)
Number of children	0	After pregnancy	25-29	0.97(0.88 to 1.08)	0.97(0.88 to 1.08)	0.98(0.88 to 1.08)
Number of children	0	After pregnancy	30-34	0.94(0.85 to 1.04)	0.94(0.85 to 1.04)	0.94(0.85 to 1.05)
Number of children	0	After pregnancy	35-39	1.00(0.91 to 1.10)	0.99(0.90 to 1.09)	0.99(0.90 to 1.10)
Number of children	0	After pregnancy	40-44	0.92(0.84 to 1.02)	0.92(0.83 to 1.02)	0.93(0.84 to 1.02)
Number of children	0	After pregnancy	45-49	1.05(0.95 to 1.16)	1.04(0.94 to 1.15)	1.04(0.94 to 1.15)
Number of children	0	After pregnancy	50-51	0.95(0.81 to 1.12)	0.94(0.80 to 1.11)	0.95(0.80 to 1.11)
Number of children	1	During pregnancy	0-4	0.90(0.67 to 1.23)	0.87(0.64 to 1.18)	0.88(0.65 to 1.19)
Number of children	1	During pregnancy	5-9	0.61(0.45 to 0.81)	0.59(0.44 to 0.79)	0.60(0.45 to 0.80)
Number of children	1	During pregnancy	10-14	0.81(0.60 to 1.09)	0.79(0.59 to 1.06)	0.79(0.59 to 1.07)
Number of children	1	During pregnancy	15-19	0.77(0.59 to 1.00)	0.75(0.58 to 0.98)	0.75(0.58 to 0.98)
Number of children	1	During pregnancy	20-24	0.76(0.57 to 1.01)	0.75(0.56 to 0.99)	0.75(0.56 to 1.00)
Number of children	1	During pregnancy	25-29	0.74(0.55 to 1.00)	0.73(0.54 to 0.98)	0.73(0.54 to 0.99)
Number of children	1	During pregnancy	30-34	0.80(0.59 to 1.07)	0.78(0.58 to 1.05)	0.78(0.58 to 1.06)
Number of children	1	During pregnancy	35-39	0.72(0.54 to 0.96)	0.71(0.53 to 0.95)	0.71(0.53 to 0.95)
Number of children	1	After pregnancy	0-4	0.88(0.65 to 1.20)	0.85(0.62 to 1.16)	0.86(0.63 to 1.17)
Number of children	1	After pregnancy	5-9	0.76(0.58 to 1.00)	0.73(0.56 to 0.97)	0.74(0.56 to 0.98)
Number of children	1	After pregnancy	10-14	1.05(0.79 to 1.38)	0.98(0.74 to 1.30)	0.99(0.75 to 1.31)
Number of children	1	After pregnancy	15-19	0.70(0.53 to 0.92)	0.69(0.52 to 0.90)	0.69(0.53 to 0.91)
Number of children	1	After pregnancy	20-24	0.95(0.72 to 1.25)	0.93(0.71 to 1.23)	0.94(0.71 to 1.23)
Number of children	1	After pregnancy	25-29	1.07(0.81 to 1.42)	1.05(0.79 to 1.39)	1.05(0.80 to 1.39)
Number of children	1	After pregnancy	30-34	1.11(0.84 to 1.46)	1.10(0.83 to 1.45)	1.10(0.84 to 1.46)
Number of children	1	After pregnancy	35-39	1.03(0.80 to 1.33)	1.03(0.79 to 1.32)	1.03(0.80 to 1.33)
Number of children	1	After pregnancy	40-44	1.05(0.81 to 1.37)	1.03(0.79 to 1.35)	1.04(0.79 to 1.36)

© 2026 Xiang N et al. *JAMA Network Open*.

Number of children	1	After pregnancy	45-49	1.11(0.85 to 1.46)	1.09(0.83 to 1.44)	1.10(0.83 to 1.44)
Number of children	1	After pregnancy	50-51	0.72(0.47 to 1.12)	0.70(0.45 to 1.09)	0.70(0.45 to 1.09)
Number of children	2	During pregnancy	0-4	0.53(0.32 to 0.88)	0.48(0.28 to 0.80)	0.48(0.29 to 0.81)
Number of children	2	During pregnancy	5-9	0.87(0.49 to 1.56)	0.85(0.47 to 1.51)	0.85(0.48 to 1.53)
Number of children	2	During pregnancy	10-14	1.34(0.79 to 2.28)	1.31(0.77 to 2.22)	1.32(0.78 to 2.24)
Number of children	2	During pregnancy	15-19	0.88(0.51 to 1.53)	0.84(0.48 to 1.45)	0.84(0.49 to 1.46)
Number of children	2	During pregnancy	20-24	0.70(0.41 to 1.21)	0.69(0.40 to 1.19)	0.70(0.40 to 1.20)
Number of children	2	During pregnancy	25-29	0.83(0.48 to 1.43)	0.78(0.45 to 1.37)	0.79(0.45 to 1.37)
Number of children	2	During pregnancy	30-34	0.62(0.35 to 1.12)	0.59(0.32 to 1.06)	0.59(0.32 to 1.06)
Number of children	2	During pregnancy	35-39	0.78(0.46 to 1.32)	0.77(0.45 to 1.31)	0.77(0.45 to 1.31)
Number of children	2	After pregnancy	0-4	0.49(0.29 to 0.83)	0.48(0.29 to 0.81)	0.49(0.29 to 0.82)
Number of children	2	After pregnancy	5-9	0.88(0.49 to 1.57)	0.86(0.48 to 1.53)	0.87(0.48 to 1.55)
Number of children	2	After pregnancy	10-14	0.83(0.47 to 1.49)	0.76(0.42 to 1.36)	0.76(0.42 to 1.38)
Number of children	2	After pregnancy	15-19	1.39(0.84 to 2.30)	1.31(0.79 to 2.18)	1.32(0.80 to 2.20)
Number of children	2	After pregnancy	20-24	0.72(0.42 to 1.24)	0.71(0.41 to 1.23)	0.72(0.42 to 1.24)
Number of children	2	After pregnancy	25-29	0.91(0.53 to 1.57)	0.84(0.48 to 1.46)	0.85(0.49 to 1.47)
Number of children	2	After pregnancy	30-34	0.80(0.46 to 1.39)	0.79(0.45 to 1.37)	0.79(0.46 to 1.38)
Number of children	2	After pregnancy	35-39	0.77(0.46 to 1.28)	0.76(0.46 to 1.28)	0.77(0.46 to 1.29)
Number of children	2	After pregnancy	40-44	0.88(0.49 to 1.56)	0.87(0.49 to 1.55)	0.88(0.49 to 1.56)
Number of children	2	After pregnancy	45-49	1.11(0.66 to 1.84)	1.10(0.66 to 1.84)	1.11(0.67 to 1.85)
Number of children	2	After pregnancy	50-51	2.81(1.21 to 6.52)	2.81(1.21 to 6.52)	2.82(1.22 to 6.55)
Number of children	3+	During pregnancy	0-4	1.96(0.66 to 5.82)	1.89(0.64 to 5.63)	1.91(0.64 to 5.69)
Number of children	3+	During pregnancy	5-9	1.16(0.51 to 2.64)	1.06(0.46 to 2.44)	1.07(0.47 to 2.47)
Number of children	3+	During pregnancy	10-14	1.36(0.53 to 3.46)	1.32(0.52 to 3.37)	1.33(0.52 to 3.40)
Number of children	3+	During pregnancy	15-19	1.14(0.43 to 3.00)	1.11(0.42 to 2.93)	1.12(0.43 to 2.95)
Number of children	3+	During pregnancy	20-24	0.60(0.27 to 1.37)	0.59(0.26 to 1.35)	0.60(0.26 to 1.35)
Number of children	3+	During pregnancy	25-29	0.73(0.34 to 1.55)	0.71(0.33 to 1.53)	0.72(0.33 to 1.53)
Number of children	3+	During pregnancy	30-34	0.78(0.36 to 1.66)	0.76(0.36 to 1.63)	0.76(0.36 to 1.63)
Number of children	3+	During pregnancy	35-39	0.65(0.29 to 1.44)	0.64(0.29 to 1.43)	0.64(0.29 to 1.43)

© 2026 Xiang N et al. *JAMA Network Open*.

Number of children	3+	After pregnancy	0-4	1.98(0.67 to 5.88)	1.92(0.65 to 5.72)	1.95(0.66 to 5.80)
Number of children	3+	After pregnancy	5-9	0.86(0.36 to 2.06)	0.84(0.35 to 2.01)	0.85(0.36 to 2.03)
Number of children	3+	After pregnancy	10-14	1.30(0.50 to 3.34)	1.27(0.49 to 3.26)	1.28(0.50 to 3.30)
Number of children	3+	After pregnancy	15-19	1.17(0.45 to 3.09)	1.06(0.40 to 2.83)	1.07(0.40 to 2.86)
Number of children	3+	After pregnancy	20-24	0.26(0.09 to 0.75)	0.26(0.09 to 0.74)	0.26(0.09 to 0.75)
Number of children	3+	After pregnancy	25-29	0.36(0.14 to 0.90)	0.35(0.14 to 0.89)	0.35(0.14 to 0.90)
Number of children	3+	After pregnancy	30-34	0.82(0.38 to 1.75)	0.81(0.38 to 1.73)	0.81(0.38 to 1.74)
Number of children	3+	After pregnancy	35-39	0.59(0.26 to 1.31)	0.58(0.26 to 1.30)	0.59(0.26 to 1.31)
Number of children	3+	After pregnancy	40-44	0.66(0.27 to 1.64)	0.66(0.27 to 1.63)	0.66(0.27 to 1.63)
Number of children	3+	After pregnancy	45-49	0.80(0.37 to 1.72)	0.86(0.39 to 1.89)	0.86(0.39 to 1.90)
Number of children	3+	After pregnancy	50-51	2.74(0.57 to 13.18)	2.74(0.57 to 13.18)	2.75(0.57 to 13.23)

*ADHD, attention deficit hyperactivity disorder.

* The incidence rate ratio was estimated by every 5 weeks, by comparing the incidence in each interval during and after pregnancy with that before pregnancy. Model 1 was adjusted for age and calendar year at childbirth and week at follow-up. Model 2 was additionally adjusted for country of birth, region of residence and education before pregnancy, season at childbirth, and income before pregnancy. Model 3 was additionally adjusted for civil status during pregnancy, multiple gestation, parity, and history of psychiatric disorders.

© 2026 Xiang N et al. *JAMA Network Open*.
