# Supplementary material for: Psychiatric Disorders Among Fathers in Sweden Before, During, and After Partner Pregnancy
Source: JAMA Netw Open. 2026 Mar 23;9(3):e262725. doi: 10.1001/jamanetworkopen.2026.2725 (PMC13010218; doi:10.1001/jamanetworkopen.2026.2725)
Supplement: Supplement 2. — Data Sharing Statement [file jamanetwopen-e262725-s002.pdf]

Data Sharing Statement

Xiang. Psychiatric Disorders Among Fathers in Sweden Before, During, and After Partner Pregnancy. *JAMA Netw Open*. Published March 23, 2026.
doi:10.1001/jamanetworkopen.2026.2725

Data
Data available: No

Additional Information
Explanation for why data not available: Swedish register data can only be accessed after granted ethical approval by appropriate authorities due to privacy protection governed by the General Data Protection Regulation. Information can be found at the Swedish National Board of Health and Welfare (https:// bestalladata.socialstyrelsen.se/, email: registerservice@socialstyrelsen.se) and/or Statistics Sweden (https://www.scb.se/vara-tjanster/bestall-data-och-statistik/, email: scb@scb.se). To access data from primary care registers in Sweden, separate applications to each region is needed (information can be found here: https://kliniskastudier.se/). Codes for data analysis can be shared upon request to the corresponding author.
